# Investigating Paracetamol’s Role as a Potential Treatment for Parkinson’s Disease: Ab Initio Analysis of Dopamine, l-DOPA, Paracetamol, and NAPQI Interactions with Enzymes Involved in Dopamine Metabolism

**DOI:** 10.1021/acsomega.3c03888

**Published:** 2023-10-03

**Authors:** Joshua Harle, Catherine Slater, Mauricio Cafiero

**Affiliations:** † School of Sciences, University of Wolverhampton, Wolverhampton WV1 1LY, U.K.; ‡ School of Chemistry Food and Pharmacy, University of Reading, Reading RG6 6AD, U.K.

## Abstract

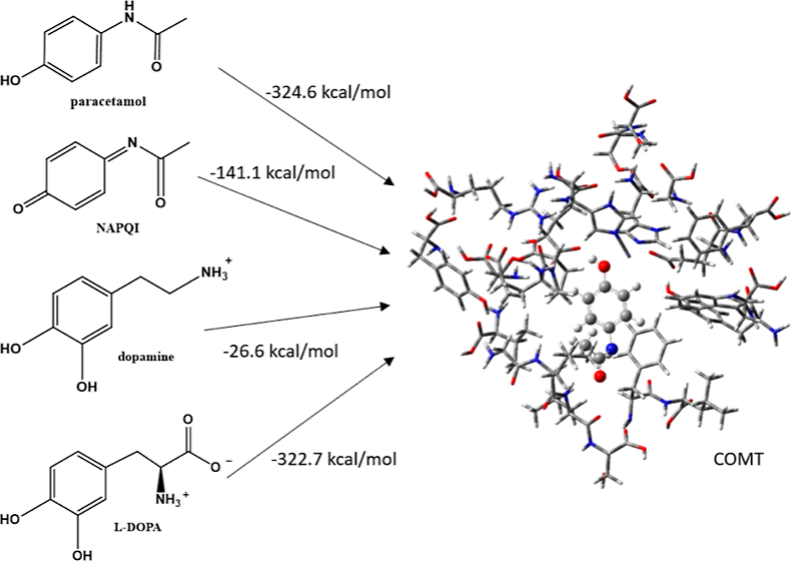

Recently, it was found that paracetamol can extend the therapeutic window of l-DOPA treatment for Parkinson’s disease [Golding (2019) BJPharm, 4(2), Article 619]. It has been posited that the effect could be due to paracetamol and its metabolite, NAPQI, inhibiting pain signals in the spinal column. In this work, we examine the possibility that the therapeutic effect of the paracetamol for the Parkinson’s disease patient may be due to an inhibition of the enzymes that metabolize dopamine and/or l-DOPA, thus effectively extending the lifetime of the l-DOPA treatment. In this work, we use the M062*X*/6-311+G* level of theory to calculate the electronic binding energies (including explicit desolvation) of several ligands (paracetamol, NAPQI, dopamine, and l-DOPA) with a series of enzymes important to the production and metabolism of dopamine and compare them to calculated binding energy values for the natural substrates for those enzymes in order to predict possible inhibition. Benchmark interaction energies for a subset of the systems studied are calculated using the more accurate second-order Møller–Plesset perturbation (MP2) method in order to calibrate the accuracy of the M062X method. If we assume that the interaction energies calculated here can serve as a proxy for in vivo inhibition, then we can predict that paracetamol and NAPQI should not inhibit the natural production of dopamine and may in fact inhibit the metabolism of l-DOPA and dopamine, thus extending the length of l-DOPA treatments.

## 1 Introduction

Parkinson’s disease (PD) is the second most common neurodegenerative disorder after Alzheimer’s disease. Those with PD suffer from reduced levels of dopamine in the brain, and this can be treated with levodopa (l-DOPA). l-DOPA can effectively cross the blood–brain barrier (BBB) and then be converted into dopamine. However, several enzymes are able to break down l-DOPA both before and after it crosses the BBB, meaning that only a small amount of l-DOPA is able to be converted into dopamine, resulting in a reduced therapeutic effect.^1^ There are three current methods used to increase the efficacy of l-DOPA treatment: inhibition of catechol-*o*-methyltransferase (COMT) and DOPA decarboxylase (DDC) in the periphery and inhibition of monoamine oxidase (MAO) in the brain.^2^ The first two approaches work by preventing the breakdown of l-DOPA before it can cross the BBB, while the third prevents the premature metabolism of dopamine in the brain. A fourth possibility is the inhibition of sulfotransferase (SULT), which is responsible for making dopamine more polar and thus more likely to be excreted. Finally, the enzyme tyrosinase, which normally acts on tyrosine, can catalyze the conversion of l-DOPA into Dopaquinone, thus reducing the therapeutic dose,^3^ and inhibition of this enzyme may also help to improve l-DOPA therapy.

**Figure 1:**
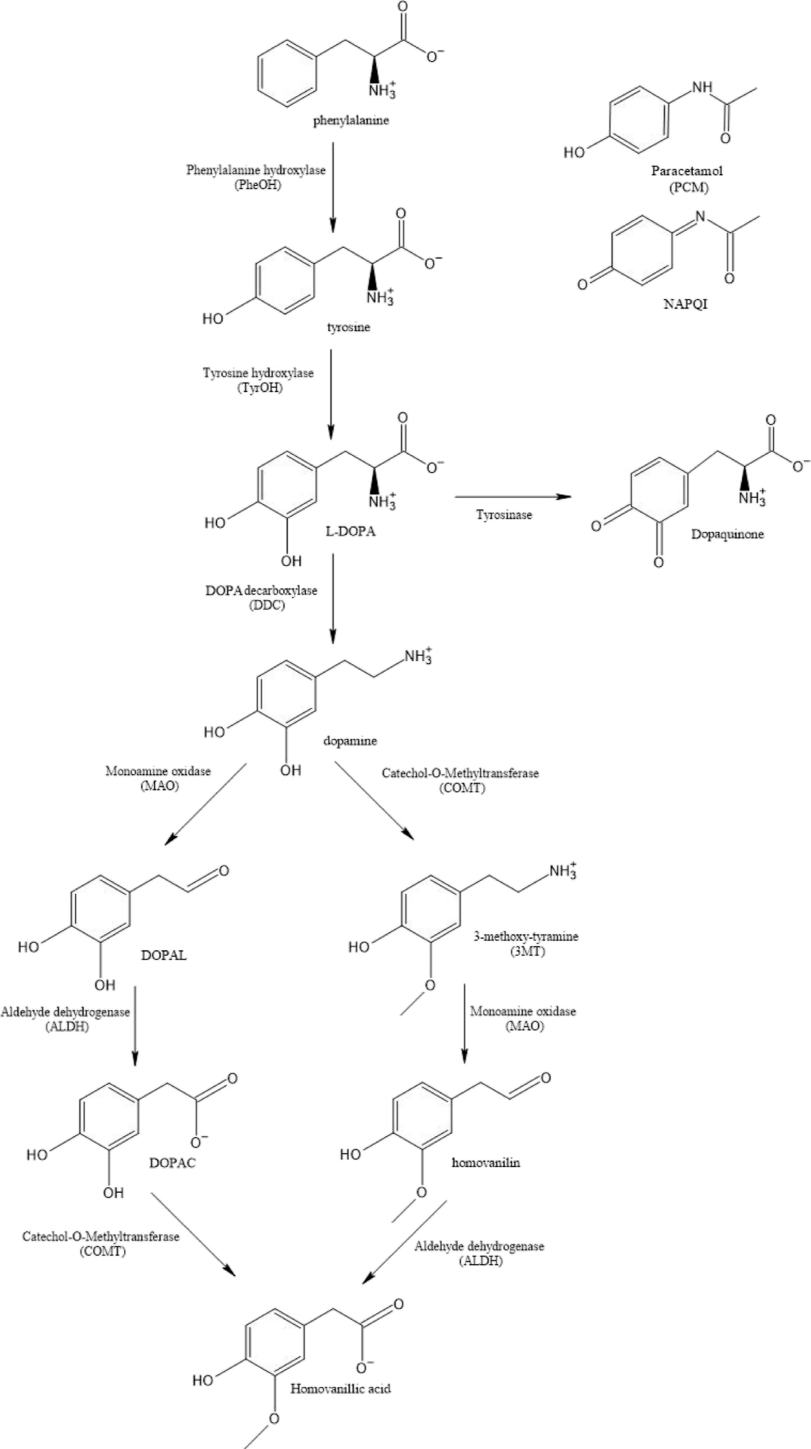
Dopamine pathway, illustrating all molecules and enzymes in this study.

A case study performed by Golding found that when patients with PD took paracetamol [*N*-(4-hydroxyphenyl) acetamide] in addition to the traditional Parkinson’s l-DOPA therapy, there was a reduction in the tremors experienced by the patients for a short period of time after the l-DOPA therapy, with the study concluding that paracetamol increased the efficacy of l-DOPA but did not hypothesize why this may be the case.^4^ Further, an experimental study by Labib et al. in 2021 found that paracetamol therapy normalized dopaminergic activity (as demonstrated by measured dopamine levels) in rats with a PD model,^5^ suggesting a therapeutic effect against PD. Work by Blecharz-Klin et al.^6^ suggests that paracetamol may influence the activity of MAO, COMT, and aldehyde dehydrogenase (ALDH), all of which are involved in the dopamine pathway and PD. This work investigates the hypothesis that paracetamol and its metabolite, NAPQI (*N*-acetyl-4-benzoquinone imine), are able to selectively inhibit the enzymes discussed above within the dopamine pathway (Figure 1) in order to increase the levels of l-DOPA in the periphery and levels of dopamine in the brain. At the same time as the desired inhibition, paracetamol and NAPQI should not inhibit phenylalanine hydroxylase (PheOH), tyrosine hydroxylase (TyrOH), and DDC in the periphery in order for the natural production of dopamine to proceed as normal. The overall dopamine level must be managed as too high a level of dopamine in the brain can cause people to become aggressive and have poor impulse control, and in cases where there is an extremely high level of dopamine, patients can experience psychosis.^7^

The selectivity of the enzymes studied here for paracetamol and NAPQI are analyzed via ab initio calculations of the electronic binding energies (EBEs, defined below) between the ligands and the enzyme active sites. First, the EBEs of the *natural substrates* of each ligand are calculated using density functional theory (DFT) at the M062X^8,9^/6-311+G*^10,11^ level (and in some cases MP2). Next, the calculated EBEs of the potential inhibitors, paracetamol, NAPQI, dopamine, and l-DOPA, are compared to these values in order to ascertain whether paracetamol and/or NAPQI can inhibit the natural activity of the enzyme. Inhibition would be shown via a comparable or stronger EBE. This study supposes that accurate calculations of relative interaction energies of ligands in different protein active sites can serve as a proxy for in vivo binding energies. In this work, we compare how sets of molecules bind noncovalently *only to a specific active site*, and so the differences between the electronic interaction energies calculated here and the in vivo binding energies should largely cancel when comparisons are made. Four cases are studied here: the ability of paracetamol and NAPQI to inhibit the natural production of dopamine in the periphery, the ability of paracetamol and NAPQI to prevent the metabolism of l-DOPA in the periphery, the ability of paracetamol and NAPQI to prevent the metabolism of dopamine in the brain, and the ability of paracetamol and NAPQI to inhibit the transport of dopamine.

## 2 Computational Methods

### 2.1 Desolvation

Explicit desolvation energies and free energies for paracetamol, NAPQI, dopamine, l-DOPA, and the other intermediate molecules studied here (phenylalanine, tyrosine, DOPAL, DOPAC, homovanilin, 3-methoxytyramine, and 3-hydroxyparacetamol; Figure 1) were calculated using M062X/cc-pVTZ.^10,12^ Our previous work explored the use of explicit, implicit, and hybrid implicit/explicit desolvation for catecholic molecules,^13^ and the model used here is based on that work. Each of the ligand molecules was surrounded by 11 explicit water molecules. The placement of the water molecules around each ligand was made as follows: two were placed near the phenolic hydroxyl group, three were placed near the end of the “tail” of the ligand, and three were placed on each “face” of the phenyl ring. This distribution of waters of solvation was based on the previous work^13^ and concentrates the solvent molecules at locations of highest positive or negative charge (hydroxyl group and tail) and areas of highest π electron density (ring). The structures of the solvated complexes were optimized to global minima, as evidenced by no imaginary vibrational frequencies. Each of the ligands was then optimized to a global minimum without the surrounding water molecules, as was a cluster of 11 water molecules. It was assumed that for all clusters, the 11 water molecules would return to the same bulk structure after desolvation. The 11 water molecules used in the solvation shell as well as the basis set used here were chosen based on our previous work.^13^ Energies and free energies were calculated for all molecules at 298.15 K
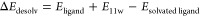

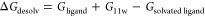
1

The total binding energies can be calculated as
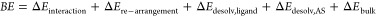
2

Of these terms, the interaction energy (first term), the rearrangement energy (second term), and the ligand desolvation energy (third term) are accounted for in this work. The desolvation of the active site (fourth term) is not needed if we are considering ligand selectivity only within each enzyme studied (i.e., not across enzymes). As the same 11 waters of solvation are used for each ligand, the bulk energy (fifth term, energy of waters returning to the bulk) is a constant and is excluded. Thus, the total electronic energy of binding used here is
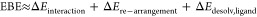
3

In these calculations, only explicit solvation was used, and free energies were calculated using zero-point, thermal, and vibrational corrections.

### 2.2 Electronic Binding Energies

Each of the ligands was then studied in up to eight enzyme active sites relevant to dopamine synthesis and metabolism. Paracetamol, NAPQI, dopamine, and l-DOPA were studied in all eight active sites, while the rest of the ligands were studied only in those enzymes for which they are a natural substrate (Figure 1). The isolation and preparation of active sites for ALDH,^14^ PheOH,^15^ TyrOH,^16^ sulfotransferase (SULT1A3),^17^ and catechol-*o*-methyltransferase (COMT)^18^ have been described in our previous work.^13,19−22^ In short, the crystal structures of each enzyme with a bound catecholic or near-catecholic ligand were identified and downloaded from the protein databank. The active site for the catecholic ligand in the crystal structure was chosen for this study. The active sites were identified as all amino acid residues with any atom within 3 Å of any atom of the catecholic ligand bound in the crystal structure (see below). For MAO,^23^ this resulted in an active site consisting of trp618, pro603, tyr934, tyr897, tyr825, tyr559, phe842, phe602, phe667, leu670, pro601, leu663, ile698, cys671, gln705, ile815, ile697, and the cofactor FAD. For tyrosinase,^24^ the resulting active site included ala221, asn205, gly216, his42, his60, his69, his204, his208, his231, met215, phe197, phe227, val217, and val218. For DDC,^25^ the active site included phe579, phe309, phe80, ile577, trp71, lys303, his192, his302, pro81, thr82, thr246, tyr79, and the cofactor pyridoxal phosphate (PLP). The previously studied active sites contained the following residues: Ala462, Cys302, Gly125, Gly294, Gly458, His293, Ile304, Phe171, Ser461, Thr129, Trp178, Tyr297, Val174, and Val460 (ALDH);^22^ Arg270, Glu280, Glu330, Gly346, His285, Phe331, Pro279, Pro281, Ser349, Ser350, Thr278, Trp326, Tyr138, and Tyr277 (PheOH);^21^ Fe501, Val291, Gly293, Leu294, Leu295, Ser296, Phe300, Leu301, Thr312, Tyr314, Arg316, Glu326, Pro327, His331, Glu332, Tyr336, Tyr371, Trp372, Glu376, Phe377, Gly392, Ser395, and Ser396 (TyrOH);^20^ Lys106, His108, His149, Glu146, Asp86, Ala148, Phe24, Phe81, Phe142, and Pro47 (SULT);^19^ and Mg, Asp169, Asn170, Asp141, Glu199, Trp38, Met40, Val42, Pro174, Trp253, Val388, Leu413, and Pro174 (COMT).^13^ In all cases, amino acid residues were capped with –H or –OH to maintain the charge found in the full protein structure. The ligands were placed in the active site by superimposing the positions of the phenyl ring and at least one hydroxyl group with those of the bound ligands from the crystal structure and allowing the entire molecule to move during the optimization. The use of enzyme active sites does offer a limitation compared with calculations using the entire protein; for example, long-range structural changes upon binding are lost in the current calculations. However, as the protein structures used here had a bound ligand, we can assume a model in which the ligand has already bound, and any structural changes have already happened. Further, the similar nature of all ligands studied suggests that the neglected long-range changes would be similar across the ligands.

All ligands were optimized in each active site using M062*X*/6-31G^11^ using implicit solvent (water) via the polarizable continuum model (PCM), specifically, the integral equation formalism variant of PCM.^26^ During the optimization, all nuclei in the ligands, all nuclei in the amino acid residue side chains, and all protons were allowed to move, while only the α-carbon and the attached carboxyl-carbon and amine-nitrogen of each amino acid were fixed. This maintains the overall structure of the active site from the crystal structure, where it had a bound phenolic or catecholic ligand, while also allowing significant relaxation to accommodate the new ligands. This model should represent a realistic active site conformation while also allowing flexibility. The counterpoise-corrected^27^ pairwise interaction energies between each optimized ligand and the *i*-th active site amino acid residue were calculated using M062*X*/6-311+G*
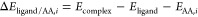
4where the energies of the separate ligand and amino acid residue calculations include all of the basis functions and DFT grid points of the ghost atoms from the opposite molecule. It should be noted that while the optimization step was performed using the implicit solvent, the interaction energies were calculated *in vacuo*. This results in interaction energies that are slightly higher than they would be within a model using the implicit solvent, as may be seen in the work of Riley et al.^28^ The pairwise interaction energies with all amino acid residues were summed to find the total electronic interaction energies per ligand in each active site
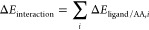
5

This reflects a model in which the active site adopts the optimal structure to bind the ligand immediately before binding to the ligand. Ucisik et al. showed that the use of pairwise interactions is in 99.9% agreement with the calculated total interactions for the M06L DFT method in the study of protein–ligand binding,^29^ and so, we do not calculate a total interaction but use a decomposition into *n*-body terms and neglect three-body and higher-order terms. Further, the residue/residue interactions [Δ^2^*E*(*i*,*j*) from eq 4 in that work] are the same with and without the ligand bound in our model, so those terms will cancel, leaving only the terms given in eq 5 here. The above interaction energy calculations were performed with second-order perturbation theory^30^ in addition to DFT for paracetamol and dopamine in the SULT active site in order to benchmark the DFT calculations. In order to better understand the interactions between ligands and the active sites, electrostatic potentials (ESPs) mapped to total electron densities were calculated at the same level of theory as that used for the interactions (M062*X*/6-311+G*).

The M06-2X functional^9^ was used in both the optimization and interaction energy calculations reported here. M06-2X is a meta-hybrid DFT method with 54% Hartree–Fock (HF) exchange that falls under the Minnesota functional umbrella and has been shown to effectively model systems with weak and noncovalent interactions.^8^ Previous work in our group studied the accuracy of M06, M062X, and M06L for the types of systems studied here,^20^ and while the M06-L functional was found to be preferable for modeling systems that contain a transition metal, in this work, only three of the eight systems modeled included a metal, and therefore, M062X was used for all systems studied for consistency. The choice of basis sets used here was based on benchmark studies in our previous work.^31^ In this work, we are concerned with *relative* energies for various molecules in a single active site, and previous work has shown that for these relative comparisons, EBEs follow the same trends as Gibbs free energies of binding.^19^ Thus, in this work, we use the EBEs to save computational expense. All calculations were carried out using Gaussian 16.^32^

## 3 Results and Discussion

### 3.1 Ligand Desolvation Energies and Free Energies

**Figure 2:**
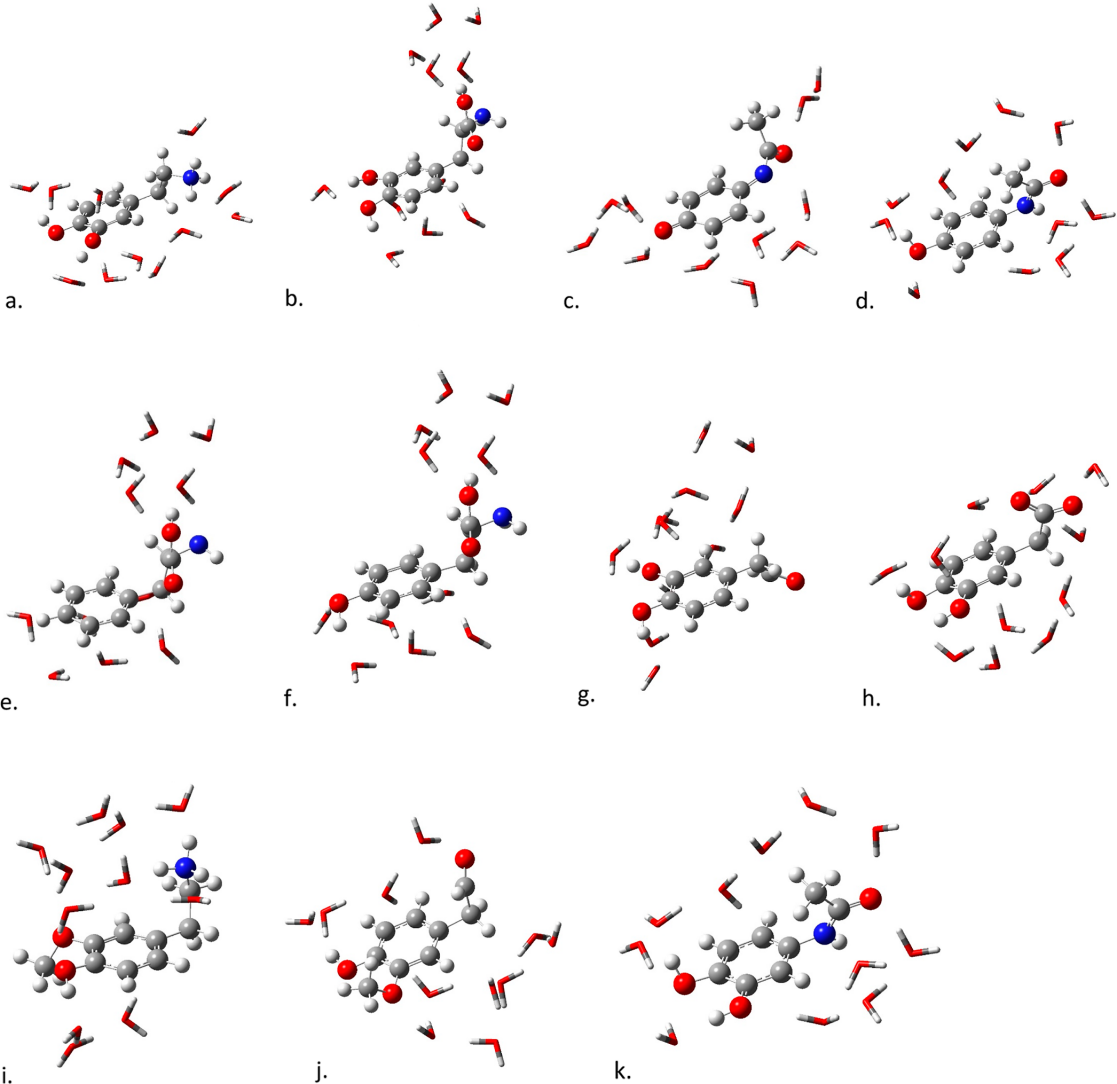
Structures for (a) dopamine, (b) l-DOPA, (c) paracetamol, (d) NAPQI, (e) phenylalnine, (f) tyrosine, (g) DOPAL, (h) DOPAC, (i) 3-methoxy tyramine, (j) homovanilin, and (k) 3-hydroxyparacetamol bound to 11 water molecules. Structures are optimized to a global minimum with M062X/cc-pVTZ.

**Figure 3:**
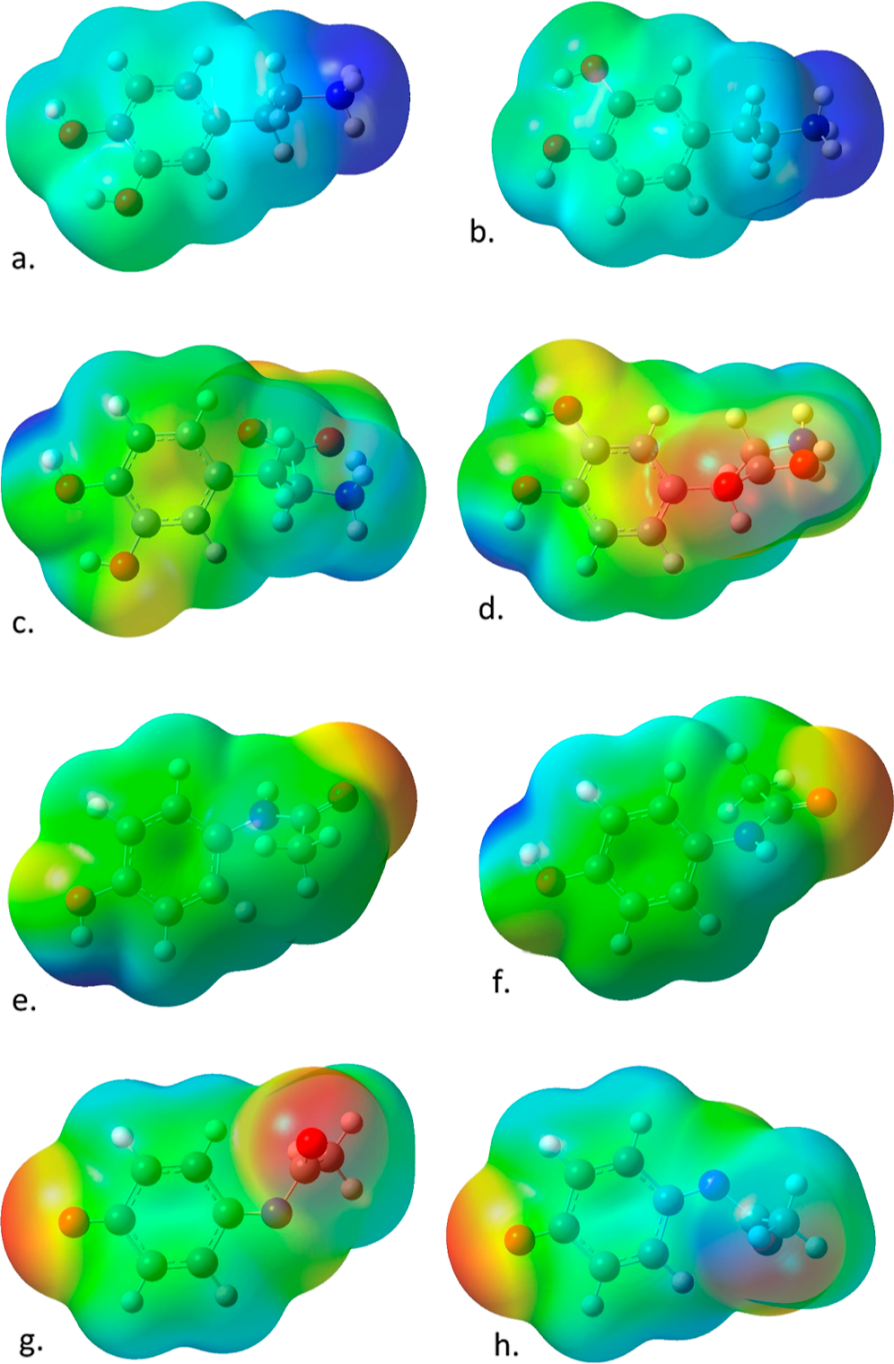
Electrostatic potentials mapped on to electron density for (a) dopamine, (b) dopamine (back view), (c) l-DOPA, (d) l-DOPA (back view), (e) paracetamol, (f) paracetamol (back view), (g) NAPQI, and (h) NAPQI (back view). Structures optimized with M062*X*/6-311+g* and implicit solvent; electron density calculated with the same method and basis set.

**Figure 4:**
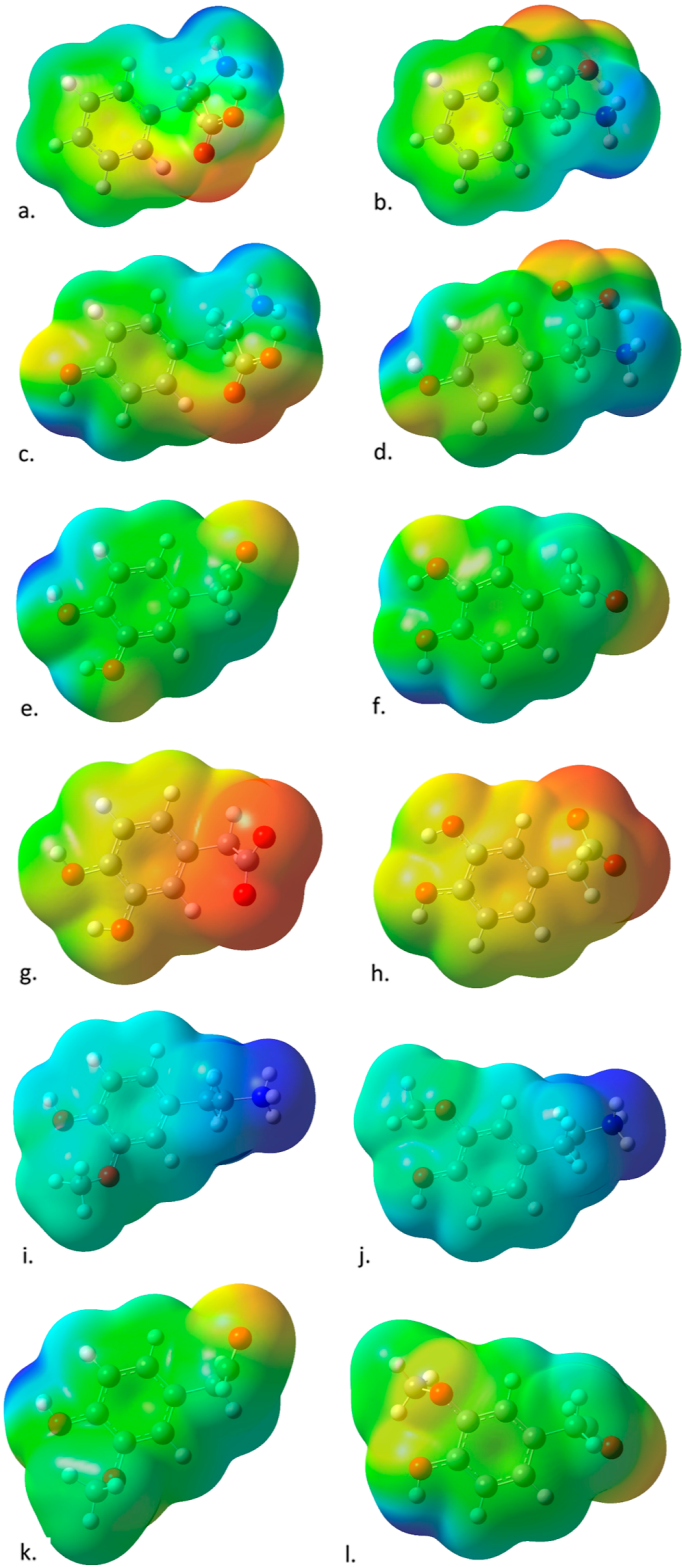
Electrostatic potentials mapped on to electron density for other substrates of the enzymes in the dopamine pathway: (a) phenylalanine, (b) phenylalanine (back view), (c) tyrosine, (d) tyrosine (back view), (e) DOPAL, (f) DOPAL (back view), (g) DOPAC, (h) DOPAC (back view), (i) 3-methoxy tyramine, (j) 3-methoxy tyramine (back view), (k) homovanilin, and (l) homovanilin (back view). Structures optimized with M062*X*/6-311+g* and implicit solvent; electron density calculated with the same method and basis set.

Figure 2 shows the optimized structures of each of the four ligands considered here with a solvation shell of 11 water molecules, while Table 1 shows the desolvation energies and free energies for all of the ligands considered here. The desolvation energies include only electronic contributions, while the free energies include zero-point, thermal, and entropic contributions. We include both in order to show consistent trends between the two values. As can be seen in Figure 2, despite similar starting configurations, optimization of the solvated complexes leads to a variety of structures. Despite starting with six water molecules near the faces of the phenyl ring before optimization, in the optimized complexes, only dopamine and DOPAC have significant solvation near the rings. In all other cases, the water molecules form a network around the more polar hydroxyl groups and tails, and in the cases of paracetamol, 3-hydroxyparacetamol, and 3-methoxy-tyramine, the water molecules form a chain from the hydroxyl group to the tail. Energy results are as expected: the charged molecules studied (dopamine, 3MT, and DOPAC) have larger, positive desolvation free energies, while the uncharged species have negative free energies. This indicates that desolvation of the charged molecules is unfavorable, while desolvation of the neutral species is favorable. This is easily understood by referencing the ESPs in Figures 3 and 4. Dopamine and 3MT have strong positive regions (shown in blue), and DOPAC has a strong negative region (shown in red). The neutral molecules have either mostly neutral coloring (green) or smaller, less intense blue and red regions that offset each other.

**Table 1:** Desolvation Energies, Free Energies (kcal/mol), and Dipoles (Debye) for the Ligands Studied Here Using an 11 Water Molecule Solvation Model Calculated with M062X/cc-pVTZ^a^

	Δ*Ε*_desolv_	Δ*G*_desolv_	Δ*G*_desolv_ – Δ*E*_desolv_	dipole
PCM	–0.8	–8.1	–7.3	4.2
NAPQI	–5.0	–13.4	–8.4	2.9
l-DOPA	6.0	–4.1	–10.1	2.6
dopamine	23.1	17.0	–6.1	14.8
phenylalanine	1.2	–8.1	–9.4	4.6
tyrosine	3.7	–4.8	–8.5	3.5
DOPAL	7.7	–4.8	–12.6	3.3
3-MT	50	37.6	–12.4	16.4
Homovanilin	8.8	–2.3	–11.1	2.4
DOPAC	53.7	38.9	–14.9	13.5
3-HP	4.00	–3.6	–7.6	3.6

a Structures are global minima. < Δ*G*_desolv_ – Δ*E*_desolv_> = −9.8 kcal/mol; σ(Δ*G*_desolv_ – Δ*E*_desolv_) = 2.5 kcal/mol.

Table 1 also shows that, on average, the free energies are about 10 kcal/mol *more negative* than the electronic energies, indicating that zero-point, thermal, and entropic contributions total about 10 kcal/mol across all molecules. The standard deviation across the molecules is 2.5 kcal/mol. Thus, in this work, we will use electronic desolvation energies in further calculations, understanding that results may be in error by around 2.5 kcal/mol when compared with free energies. Table 3 shows the electronic interaction energies for all ligands with the enzyme active sites, while Table 4 shows EBEs for the same. The values in Table 4 are obtained by adding the electronic desolvation energies (Table 1) to the appropriate values in Table 3, as shown in eq 3. Tables with all raw data from the calculations can be found in the Supporting Information.

**Table 2:** Interaction Energies (kcal/mol) between Dopamine (DA) and Paracetamol (PCM) and the Active Site for SULT1A3 Using M062X and MP2 with the 6-311+G* Basis Set^a^

	Ala148	Asp86	Glu146	His108	His149	Lys106	Phe142	Phe24	Phe81	Pro47	Total
DA/M062X	–0.46	–124.99	–88.53	–17.78	46.12	20.34	–10.53	–4.07	–1.10	0.15	–180.95
DA/MP2	–0.66	–119.99	–85.27	–15.47	–45.33	23.41	–8.96	–3.75	–1.82	–0.16	–167.34
MP2–M062X	–0.20	5.00	3.26	2.31	–0.79	3.06	1.57	0.32	–0.72	–0.32	13.61
<MP2–M062X>										1.35	
PCM/M062X	–0.59	39.98	49.16	–114.40	–1.29	–132.54	–6.83	–5.45	–1.90	–1.29	–175.25
PCM/MP2	–0.56	40.70	51.00	–110.12	–1.52	–125.97	–4.93	–4.96	–2.64	–1.28	–160.28
MP2–M062X	0.03	0.71	1.84	4.28	–0.24	6.57	1.90	0.49	–0.75	0.01	14.97
<MP2–M062X>										1.49	

a Also included are the differences between the two methods (MP2–M062X) and the average of the differences.

**Table 3:** Total Electronic Interaction Energies (kcal/mol) for the Ligands Studied Here in Various Enzyme Active Sites^a^

	PheOH	TyrOH	DDC	COMT	tyrosinase	MAOB	ALDH	SULT
PCM	–45.7	–152.6	–38.6	–323.8	*–243.2*	–22.7	*–18.0*	*–175.1*
NAPQI	–47.2	–148.4	–31.2	–136.1	*–230.5*	–25.3	*–26.0*	–44.6
l-DOPA	–111.4	–236.2	–93.5	–328.7	**–287.1**	–47.7	–50.0	–119.5
dopamine	–116.5	–124.2	–213.5	–49.7	–158.7	**–111.8**	–49.2	**–180.8**
phenylalanine	**–80.2**							
tyrosine		**–197.0**						
DOPAL							**–27.0**	
3-MT						**–107.4**		
homovanilin							**–24.8**	
DOPAC				**–410.1**				
3-HP				*–690.3*				

a Bold indicates the natural substrate for each enzyme in the dopamine pathway, and italics indicates whether PCM or NAPQI will be competitive inhibitors. Energies calculated with M062X/6-311+G* on structures optimized with M062X/6-31G.

**Table 4:** Total EBEs (Electronic Interactions with Electronic Ligand Desolvation; kcal/mol) for the Ligands Studied Here in Various Enzyme Active Sites^a^

	PheOH	TyrOH	DDC	COMT	tyrosinase	MAOB	ALDH	SULT
PCM	–46.5	–153.4	–39.4	*–324.6*	*–244.0*	–23.5	*–18.9*	*–176.0*
NAPQI	–52.2	–153.4	–36.2	–141.1	*–235.5*	–30.4	*–31.0*	–49.6
l-DOPA	–105.4	–230.2	**–87.4**	–322.7	**–281.0**	–41.7	–44.1	–113.5
dopamine	–93.4	–101.1	–190.4	**–26.6**	–135.6	**–88.7**	–26.1	**–157.7**
phenylalanine	**–79.0**							
tyrosine		**–193.3**						
DOPAL							**–19.3**	
3-MT						**–57.4**		
homovanilin							**–16.0**	
DOPAC				**–356.4**				
3-HP				*–686.3*				

a Bold indicates the natural substrate for each enzyme in the dopamine pathway, and italics indicates if PCM or NAPQI will be competitive inhibitors. Energies were calculated with M062*X*/6-311+G* on structures optimized with M062*X*/6-31G.

The Gibbs energy of desolvation can be roughly correlated with the polarity of the molecule. Table 1 shows the magnitudes of the total dipoles for each molecule studied here. Figure S1 (Supporting Information) shows these dipole magnitudes plotted against the Gibbs energy of desolvation. While the correlation is not linear (*R*^2^ = 0.85), it may be seen that a value of the dipole below 5 D leads to a slightly favorable desolvation, while a value of the dipole above 12 D leads to a larger, unfavorable desolvation.

### 3.2 Comparison of M062X and MP2 Interaction Energies

Table 2 (see the end of the paper) shows the interaction energies for dopamine and paracetamol with each amino acid in the active site of SULT1A3 calculated with both M062X and MP2 and the 6-311+G* basis set. The table also shows the difference between the M062X and MP2 values for each ligand/amino acid pair and the average difference for each molecule. The differences are small (average of 1.35–1.49 kcal/mol difference for each molecule pair), and M062X usually produces more negative values. In the cases where the differences are larger (such as a difference of 5 kcal/mol for Asp86 with dopamine), they are still less than 5% of the total interaction energies. Overall, for dopamine, M062X produces a value that is 14 kcal/mol more negative (more attractive) than MP2, while for paracetamol, M062X produces a value that is about 15 kcal/mol more negative than MP2. In both cases, the difference is less than 10% of the total interaction energy. Thus, in the further work, we will use the M062X method for calculations, with the understanding that it may be more attractive than an MP2 calculation by about 10%.

### 3.3 Potential Inhibition of the Biosynthesis of Dopamine

**Figure 5:**
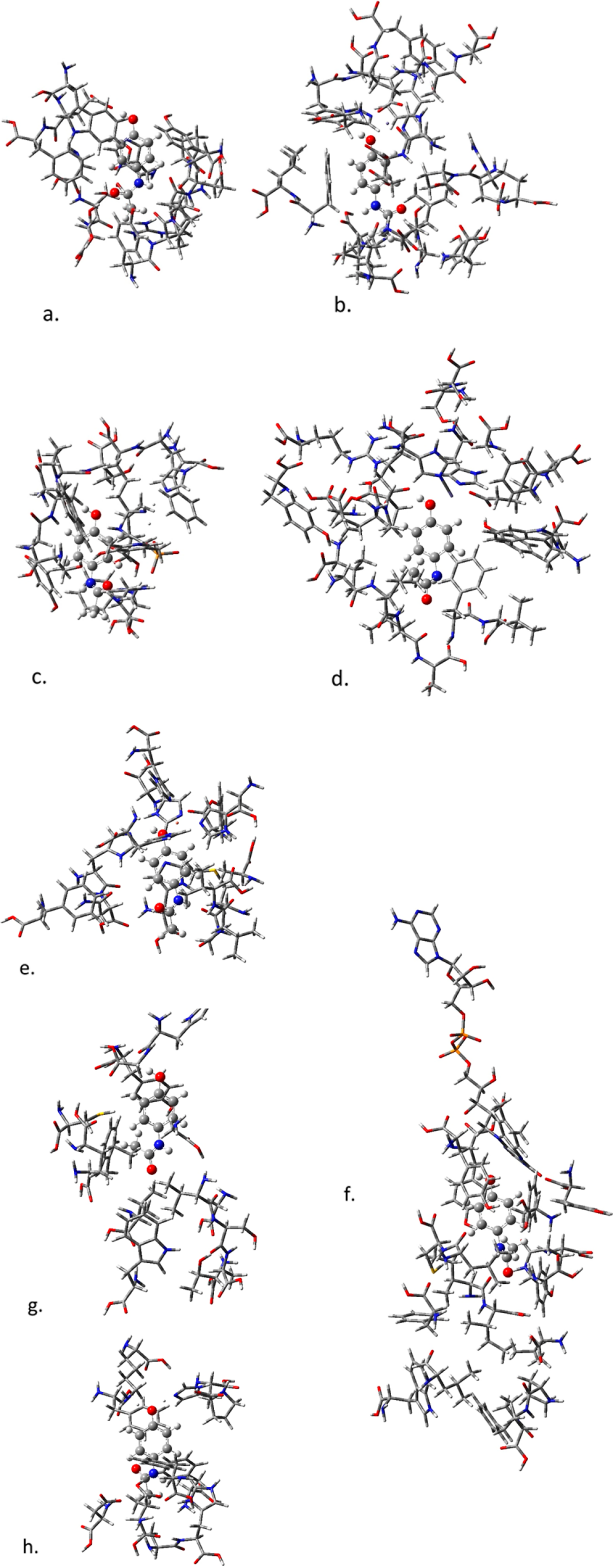
Optimized structures (M062*X*/6-31G) of paracetamol in the active sites for (a) PheOH, (b) TyrOH, (c). DDC, (d) COMT, (e) tyrosinase, (f) MAO, (g) ALDH, and (h) SULT.

Figure 5 shows the optimized structures of paracetamol in the active sites of each of the eight enzymes studied here (the optimized structures of all ligands in each active site are available in the Supporting Information and Figures S2–S6). As can be seen in Figure 1, PheOH, TyrOH, and DDC are needed for the synthesis of dopamine starting from dietary phenylalanine. DDC alone is needed for the conversion of l-DOPA to dopamine. Thus, in order to best extend the effects of l-DOPA therapy, PheOH and TyrOH should not be inhibited as this would lead to a decrease in naturally produced dopamine. At the same time, DDC should be inhibited in the periphery so that l-DOPA is not converted to dopamine before reaching the BBB. Carbidopa is a well-known DDC inhibitor^2^ and is commonly administered with l-DOPA to prevent early metabolism. This work will examine the ability of paracetamol and NAPQI to also inhibit DDC through the proxy value of the EBE, which is different from binding free energies but whose relative values are assumed to follow the same patterns as in vivo binding energies.

Table 4 shows the EBEs of the ligands studied here in the active sites of the three enzymes mentioned above. For PheOH, which should not be inhibited, we see that paracetamol and NAPQI have EBEs *half* that of the natural substrate, phenylalanine. Thus, within the error margins established for this work, paracetamol and NAPQI can be predicted to not inhibit PheOH. This is in agreement with clinical practice wherein patients with phenylketonuria, a condition wherein patients have reduced PheOH activity, are commonly prescribed paracetamol.^33^ For paracetamol and NAPQI in PheOH, approximately half of the total binding interactions come from the two deprotonated glutamate residues. For the other ligands, the glutamate residues are also attractive, and serine, threonine, and tryptophan also have strong attractive interactions, but arginine forms a strongly repulsive interaction with dopamine due to both molecules having a positive charge (see full table in the Supporting Information). For TyrOH, paracetamol and NAPQI have EBEs about three-fourths of the natural substrate, tyrosine, and so again, the enzyme should not be inhibited within the error margins established here. This result is supported by the experimental work of Courade et al.,^34^ wherein the activity of TyrOH was measured after administration of up to 1 mM paracetamol and no changes were detected. In TyrOH, the neutral paracetamol and NAPQI ligands are held strongly only by Fe^2+^, with all other residues contributing less than 5% of the total interaction energy, but for the ligands with charges, the glutamate and arginine residues contribute strongly along with Fe^2+^.

For DDC, Table 4 shows that paracetamol and NAPQI have EBEs about *half* that of the natural substrate, l-DOPA, meaning that they will likely not compete with l-DOPA, and thus, they will not prevent the metabolism of l-DOPA before the BBB. Thus, out of the three targets, none will be inhibited by paracetamol and NAPQI according to this work, and so, the biosynthesis of l-DOPA and dopamine will not be inhibited. For DDC, histidine and lysine form strong polar interactions with paracetamol and NAPQI, with phenylalanine and tyrosine also contributing significantly to the overall binding. For the other charged ligands, we see these same contributions, but the bulk of the interactions in those come from the PLP cofactor. A review of the literature does not reveal any phenolic DDC inhibitors; all known DDC inhibitors are catecholic,^2^ and thus the results here are expected. Recently, it has been shown that inhibition of DDC in the periphery can lead to an overall increase in the activity of DDC, which is detrimental to PD patients overall,^35^ so this avenue of treatment should be dealt with carefully.

### 3.4 Inhibition of l-DOPA Metabolism in the Periphery

In order for l-DOPA to reach the brain, it must not be metabolized in the periphery. l-DOPA is normally metabolized by DDC but, in some cases, may also be metabolized by COMT, such as the case when DDC is already inhibited in the periphery. We have discussed the inhibition of DDC in Section 3.3. Entacapone and tolcapone are well-known COMT inhibitors,^2^ and we will examine the ability of paracetamol and NAPQI to inhibit COMT as well. Further, tyrosinase can also metabolize l-DOPA and may be a target for inhibition as well. Thus, to improve the efficacy of l-DOPA therapy, DDC, COMT, and tyrosinase may be inhibited in the periphery. The work of Thareja et al.^36^ suggests that the paracetamol metabolite 3-hydroxy-paracetamol (see Figure 1) may also function as a substrate of COMT, so we have examined that molecule here as well. All inhibition in this section is examined through the proxy value of the EBE, which is different from binding free energy but whose relative values are assumed to follow the same patterns as in vivo binding energies.

Table 4 shows the EBEs of the ligands studied here in the active sites of the three enzymes mentioned above. As we have pointed about in Section 3.3, paracetamol and NAPQI are unlikely to inhibit DDC. For COMT, while there is a lack of evidence that paracetamol binds to the enzyme, it has been known that noncatechol/phenolic-type compounds (like paracetamol) can bind strongly,^37^ thus suggesting that paracetamol may also bind there. This work shows that paracetamol has an EBE much stronger than that of one natural substrate (dopamine) and of similar magnitude to those of the other substrates DOPAC and l-DOPA, so within the error margins of this work, paracetamol should inhibit COMT. NAPQI has a much weaker EBE than paracetamol, but it is stronger than the EBE of one substrate (dopamine), so it should provide some inhibition as well, despite not having a catecholic or phenolic structure. Finally, the paracetamol metabolite 3-hydroxy-paracetamol has an EBE *almost* twice that of the natural substrates, and so should serve as an inhibitor of COMT as well. This strong inhibition is due to 3-hydroxy-paracetamol being deprotonated by a glutamate residue in the active site, creating a divalent anion that binds strongly to Mg^2+^ in the active site. In general, Mg^2+^ forms the strongest interaction for all ligands in COMT, both attractive (for most ligands) and repulsive (for dopamine, due to its positive charge). Glutamate, aspartate, and lysine residues also form strong interactions with ligands, again based on charges. Paracetamol, 3-hydroxyparacetamol, l-DOPA, and DOPAC also form strong attractive interactions with asparagine. The strong attraction of 3-hydroxy-paracetamol for the COMT active site is in agreement with the experimental observations of Thareja et al.^36^ In their work they found that 3-methoxyparacetamol is produced in the location of the COMT enzyme; this molecule is made from 3-hydroxyparacetamol, suggesting that it is a substrate for COMT. For tyrosinase, both paracetamol and NAPQI have EBEs within 14% of that of the natural substrate l-DOPA, so they should both be competitive inhibitors for that active site. The experimental work of Valero et al.^38^ uses UV/vis spectroscopy, O_2_ consumption, and inhibition studies to show that paracetamol does in fact bind to tyrosinase to be converted to the oxidized form supporting this result. Further, tyrosinase oxidizes monophenols (like paracetamol) and diphenols (like l-DOPA and dopamine) from two different states: a monophenol-active state and a diphenol-active state. When the diphenol-active state binds to a monophenol, it locks the enzyme into a “dead-end” state, meaning that the diphenol cannot be oxidized until a large enough concentration of it accumulates. In this way, paracetamol acts as an inhibitor for the enzyme when compared to l-DOPA.

Thus, for the three enzymes that should be inhibited in the periphery to boost l-DOPA therapy efficacy, we predict that paracetamol, NAPQI, or both will inhibit two of them (COMT and tyrosinase) while leaving the third (DDC) uninhibited and 3-hydroxyparacetamol will inhibit COMT (in agreement with experimental studies^36^).

### 3.5 Inhibition of l-DOPA Metabolism in the Brain

After l-DOPA passes the BBB, it is converted to dopamine by DDC. Thus, after the BBB, the efforts to boost the efficacy of l-DOPA therapy switch from inhibiting the breakdown of l-DOPA to inhibiting the breakdown of dopamine. The enzymes that should be inhibited in this case are COMT and MAO, as well as ALDH (to a lesser degree as it is dependent on the action of MAO). We can study that inhibition through the proxy value of the EBE, which is different from the binding free energy but whose relative values are assumed to follow the same patterns as in vivo binding energies. We have seen in Section 3.4 that both paracetamol and NAPQI can inhibit COMT. Table 4 shows that with EBEs less than half of that of natural substrates dopamine and 3-methoxy-tyramine, paracetamol and NAPQI are unlikely to inhibit MAO strongly. The interactions between ligands and MAO active site residues are largely weak, though a leucine and a tyrosine residue do often form strong interactions with the charged ligands. For most ligands, the strongest interaction is with the negatively charged cofactor, FAD, especially for dopamine and 3-methoxytyramine (both positively charged). Cysteine and glutamine residues also contribute some attraction to the neutral paracetamol and NAPQI ligands. This result is consistent with the experimental work of Courade et al., who showed that paracetamol in concentration of up to 100 mM had no effect on MAO(A) activity.^34^ Kolawole showed experimentally and via docking simulations that paracetamol does bind to ALDH,^39^ and in fact, our work also shows that both paracetamol and NAPQI should be competitive inhibitors for ALDH, with EBEs stronger than natural substrates DOPAL and homovanilin. Inhibition of ALDH may thus cause a shift in equilibrium, which would indirectly inhibit MAO.

### 3.6 Transport of Dopamine

It is well-known that the polarity/transport ability in aqueous solution of both paracetamol^40^ and dopamine^17,41^ are enhanced by sulfation by the SULT enzyme, and that this polarity increase leads to excretion. Indeed, the results in Table 4 show that both paracetamol and dopamine bind strongly to SULT, with almost the same magnitude. These interactions are due largely to charged lysine, glutamate, and aspartate residues, with histidine and phenylalanine also making contributions. The work of Yamamoto et al.^40^ experimentally confirms the binding of paracetamol to the version of SULT used here (SULT1A3), and the^41^ work of Renskers, Feor, and Roth^41^ confirms the binding of dopamine to SULT1A3, supporting our results. NAPQI binds to SULT with a much weaker EBE. Thus, if we assume that our EBEs can serve as a proxy for in vivo binding energies, we can predict that paracetamol will compete with dopamine for SULT, meaning that dopamine may remain in the system longer before being excreted, thus increasing the l-DOPA therapy efficacy.

## 4 Conclusions

This work has shown that paracetamol and NAPQI will not inhibit the natural production of dopamine and may in fact improve the efficacy of l-DOPA therapy for PD patients by acting as inhibitors of COMT, tyrosinase, ALDH, and SULT. This has been demonstrated by the use of EBEs, which, when used for relative values as in this work, can mimic the pattern of in vivo binding energies. Further, we show that the paracetamol metabolite 3-hydroxyparacetamol may also inhibit COMT. Paracetamol and NAPQI will not inhibit MAO and DDC and thus are not broadly effective as adjuncts to l-DOPA therapy. Experimental observations, where they exist, and MP2 calculations support the findings here, serving as a benchmark for this work.

The model chemistry used here for electronic interaction energies, M062*X*/6-311+g*, was calibrated against MP2 with the same basis set and provided results in close agreement. Energies and free energies of desolvation yielded qualitatively correct results and offered clear criteria for when a ligand would have a favorable or unfavorable desolvation energy based on the ligand dipole moment.

## Supporting Information Available

All optimized structures for ligands in enzyme active sites, all pairwise interaction energies for each ligand in each enzyme, and *XYZ* coordinates for all optimized protein/ligand structures (PDF)

## Abbreviations

3-HP: 3-hydroxyparacetamol
3-MT: 3-methoxytyramine
DOPAL: 3,4-dihydroxyphenylacetaldehyde
DOPAC: 3,4-dihydroxyphenylacetic acid
NAPQI: *N*-acetyl-4-benzoquinone imine
ALDH: aldehyde dehydrogenase
COMT: catechol-*O*-methyltransferase
DDC: DOPA decarboxylase
MAO: monoamine oxidase
PCM: paracetamol
PheOH: phenylalanine hydroxylase
SULT: sulfotransferase
TyrOH: tyrosine hydroxylase
