# Supplementary material for: Investigating Paracetamol’s Role as a Potential Treatment for Parkinson’s Disease: Ab Initio Analysis of Dopamine, l-DOPA, Paracetamol, and NAPQI Interactions with Enzymes Involved in Dopamine Metabolism
Source: ACS Omega. 2023 Oct 3;8(41):38053–63. doi: 10.1021/acsomega.3c03888 (PMC10586264; doi:10.1021/acsomega.3c03888)
Supplement: Supplementary file 1 — ao3c03888_si_001.pdf [file ao3c03888_si_001.pdf]

Investigating Paracetamol's Role as a Potential Treatment for Parkinson's Disease: *ab initio* Analysis of Dopamine, L-DOPA, Paracetamol, and NAPQI Interactions with Enzymes involved in Dopamine Metabolism

Supporting Information

Joshua Harle[2], Catherine Slater[1] and Mauricio Cafiero[2*]

[1]School of Sciences, University of Wolverhampton, Wolverhampton, UK, WV1 1LY
[2]School of Chemistry, Food and Pharmacy, University of Reading, Reading, UK, RG6 6AD
*corresponding author

Figures of all optimized structures for ligands in enzyme active sites and tables of all pairwise interaction energies for each ligand in each enzyme.

Coordinates of all optimized protein/ligand complexes.

Figure S1. Relationship between Gibbs energy of solvation and total dipoles for the molecules studied here.

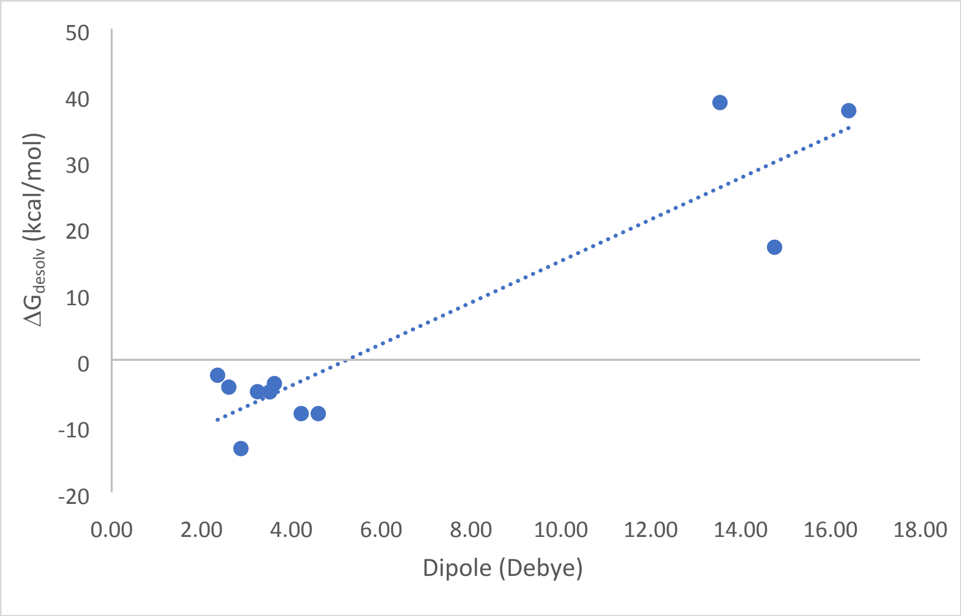

Figure S2. M062X/6-31G, implicit solvent (water) optimized structures of Paracetamol in each enzyme active site. Amino acid residue backbone atoms (alpha carbon and attached C and N) are fixed in the optimization and all other atoms are allowed to move during optimization.

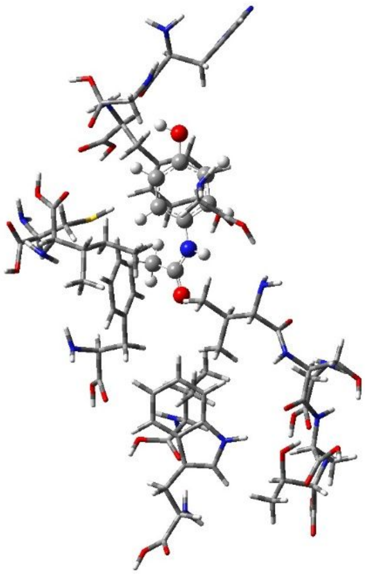

ALDH PCM

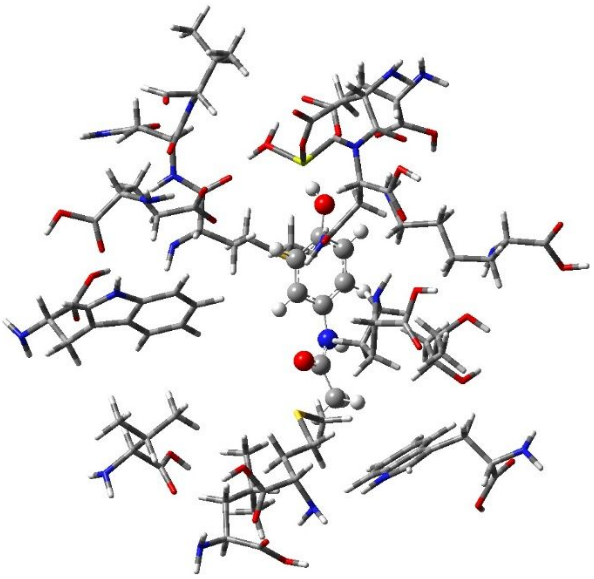

COMT PCM

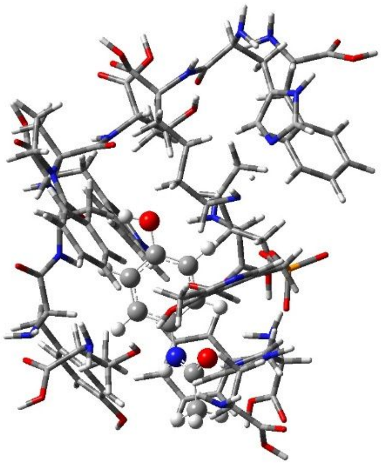

DCC PCM

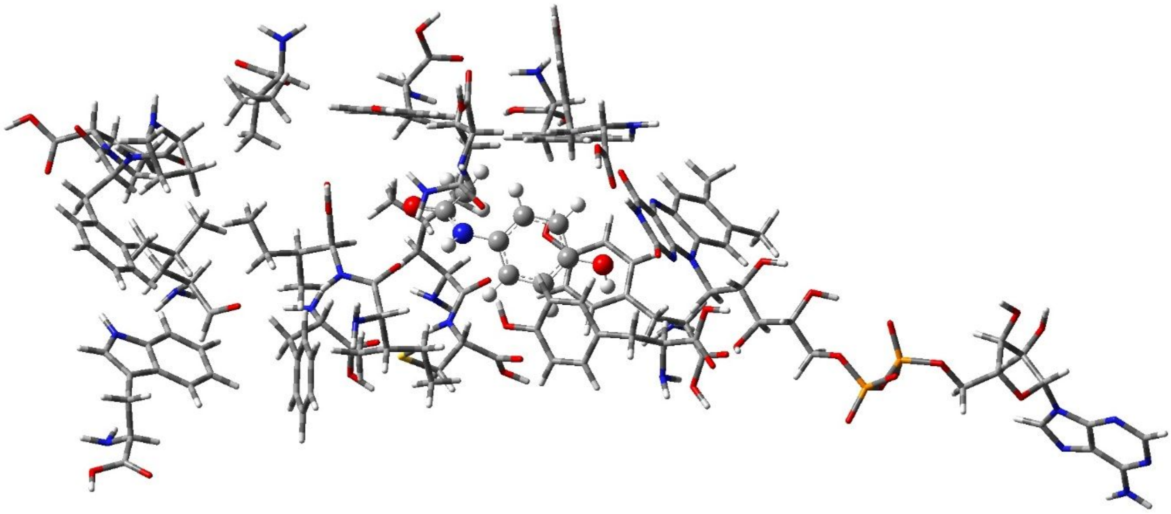

MAOB PCM

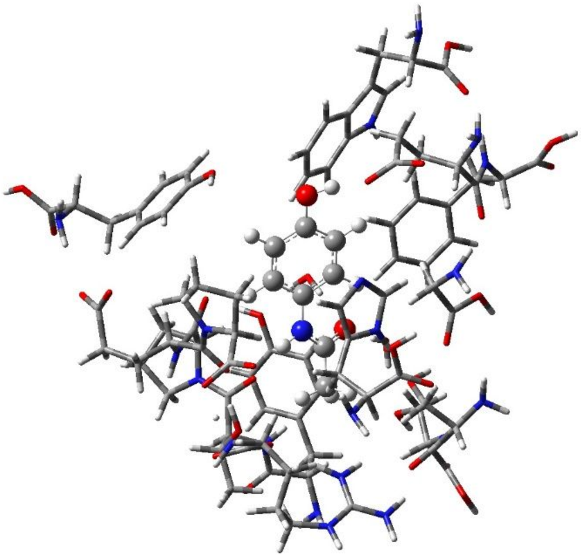

PheOH PCM

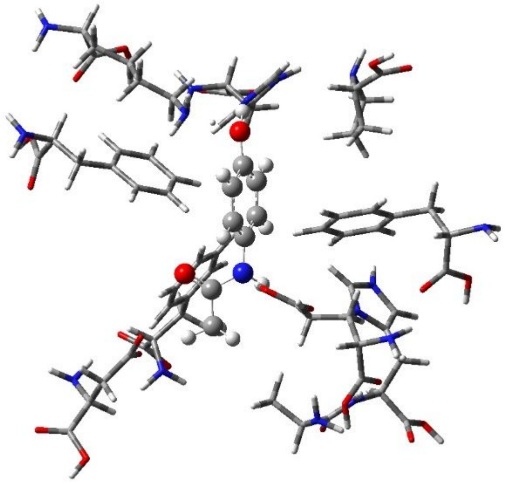

Sult1a3 PCM

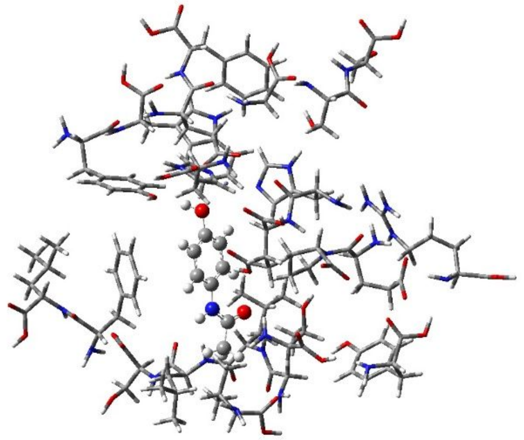

TyrOH PCM

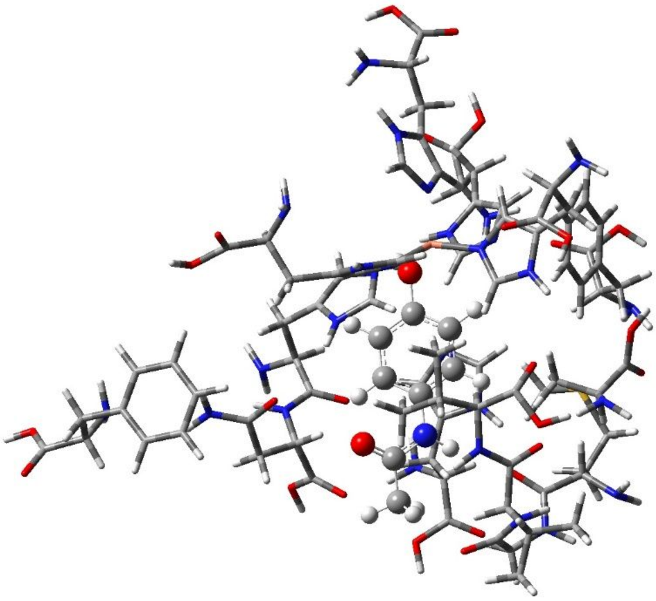

Tyrosinase PCM

Figure S3. M062X/6-31G, implicit solvent (water) optimized structures of NAPQI in each enzyme active site. Amino acid residue backbone atoms (alpha carbon and attached C and N) are fixed in the optimization and all other atoms are allowed to move during optimization.

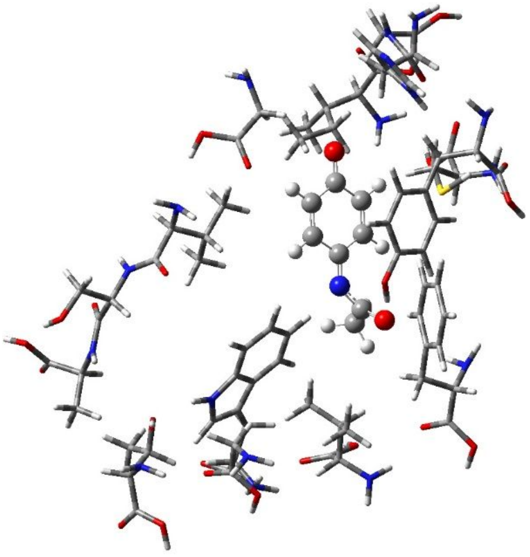

ALDH NAPQI

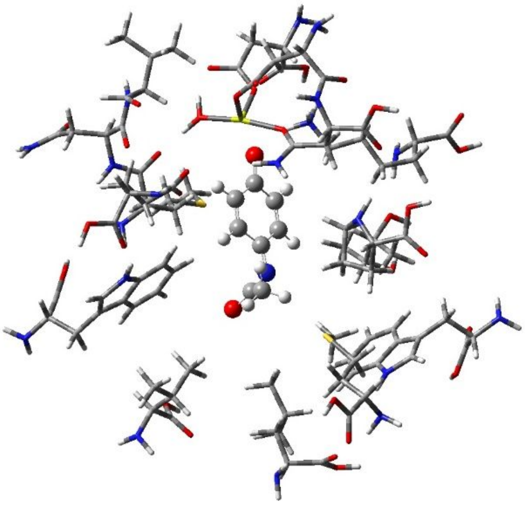

COMT NAPQI

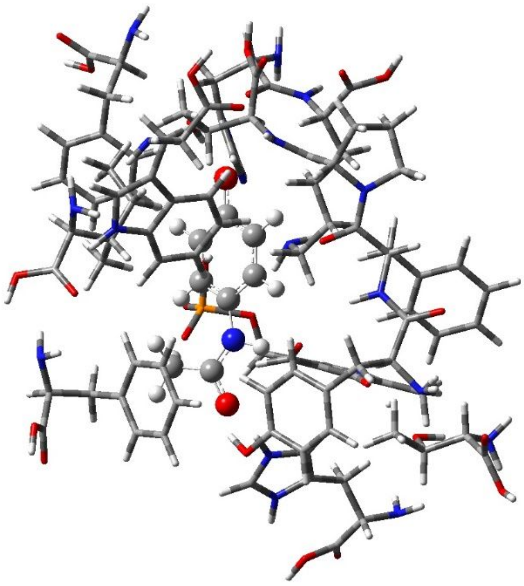

DCC NAPQI

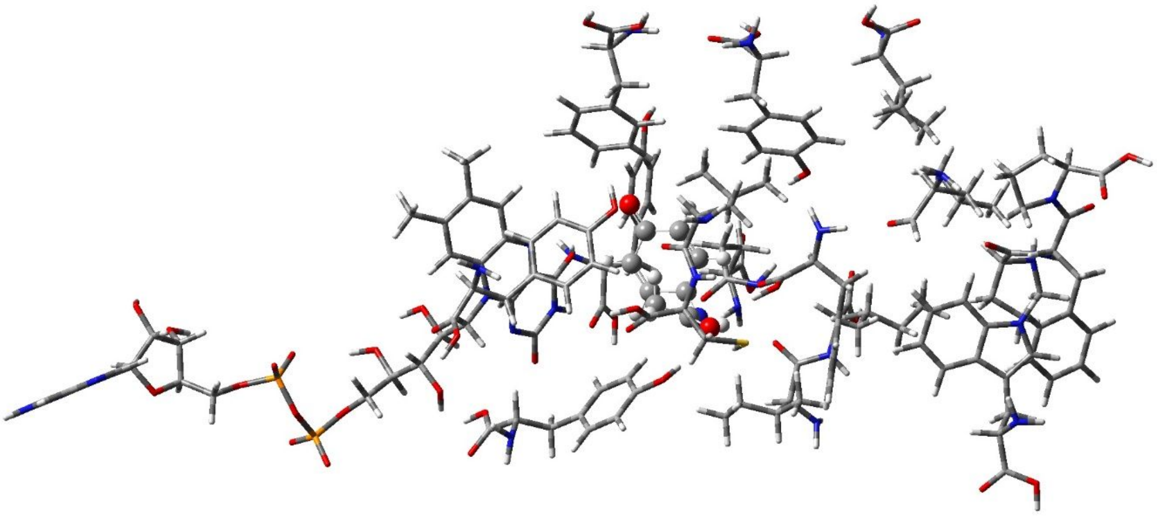

MAOB NAPQI

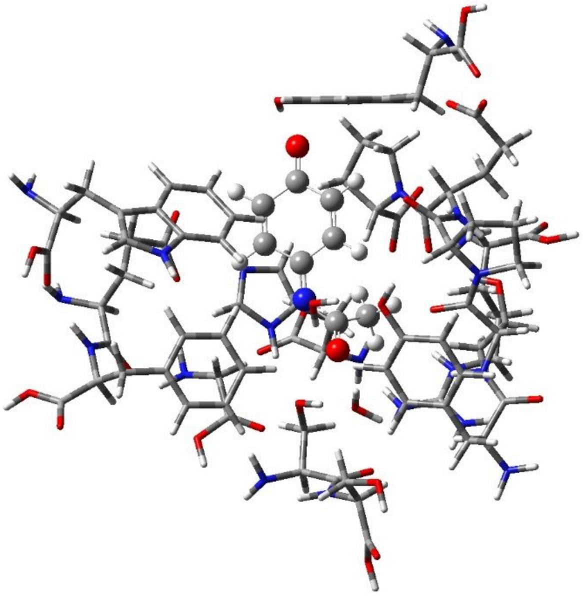

PheOH NAPQI

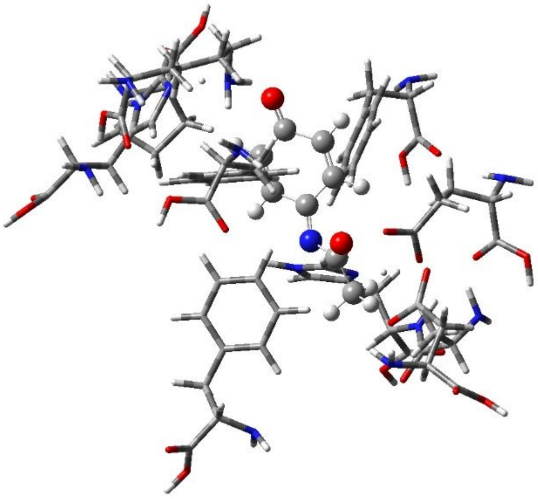

Sult1a3 NAPQI

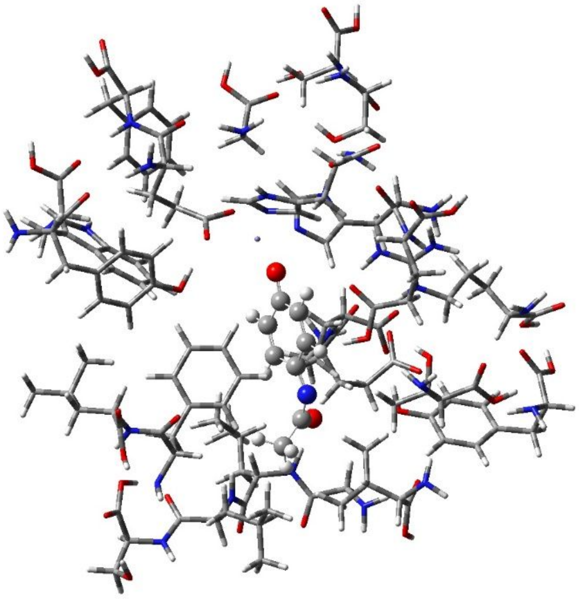

TyrOH NAPQI

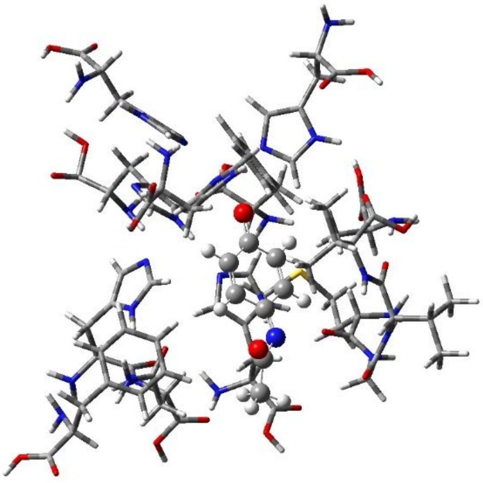

Tyrosinase NAPQI

Figure S4. M062X/6-31G, implicit solvent (water) optimized structures of Dopamine in each enzyme active site. Amino acid residue backbone atoms (alpha carbon and attached C and N) are fixed in the optimization and all other atoms are allowed to move during optimization.

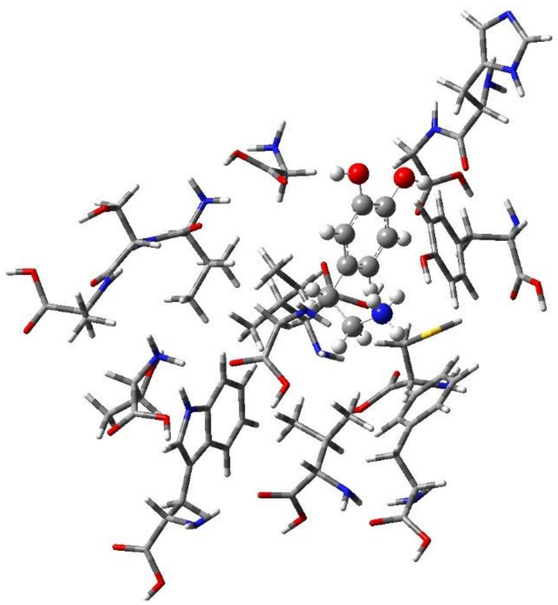

ALDH Dopamine

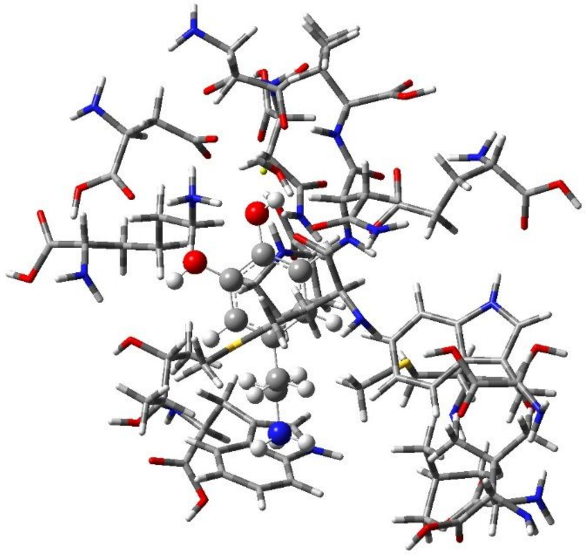

COMT Dopamine

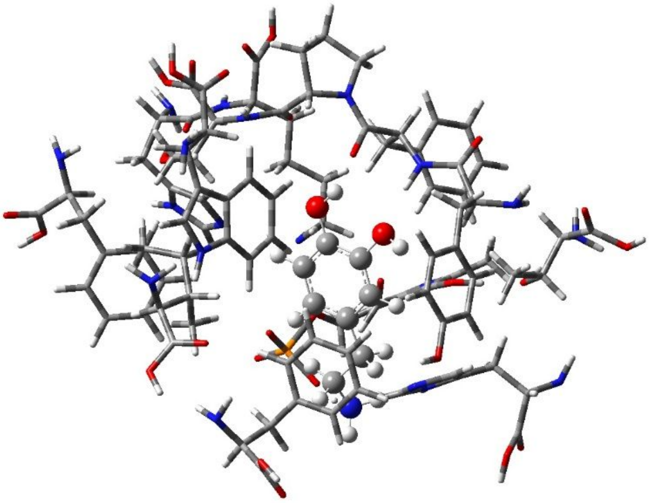

DCC Dopamine

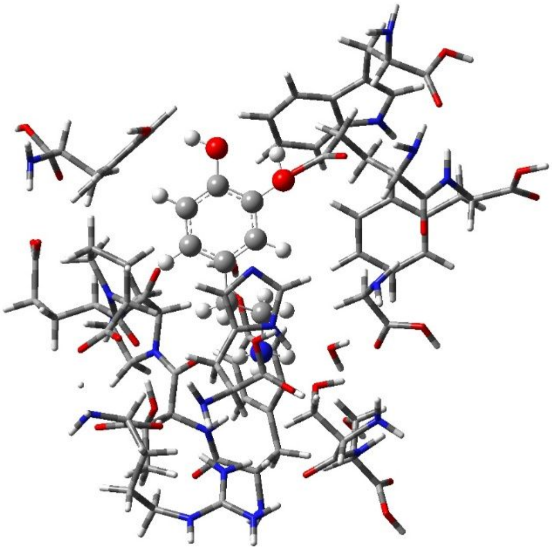

PheOH Dopamine

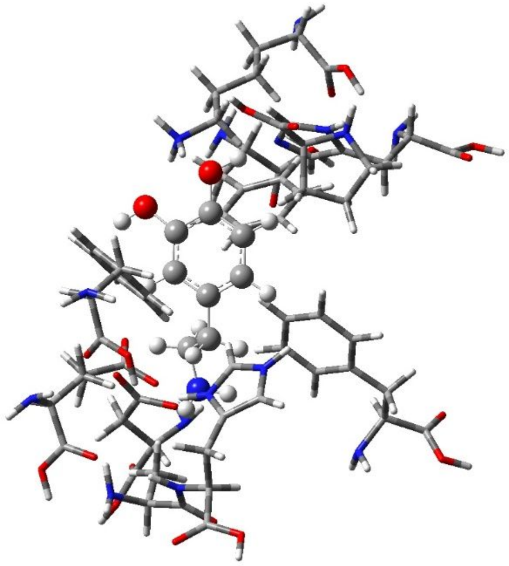

Sult1a3 Dopamine

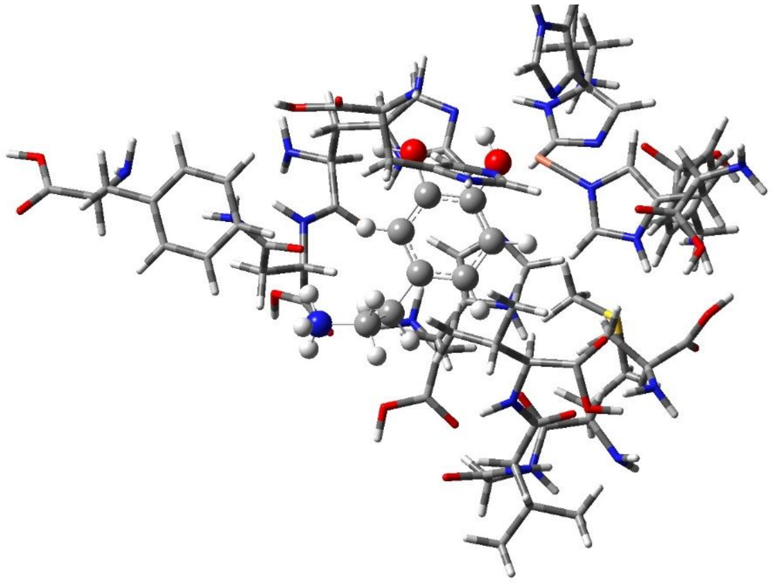

Tyrosinase Dopamine

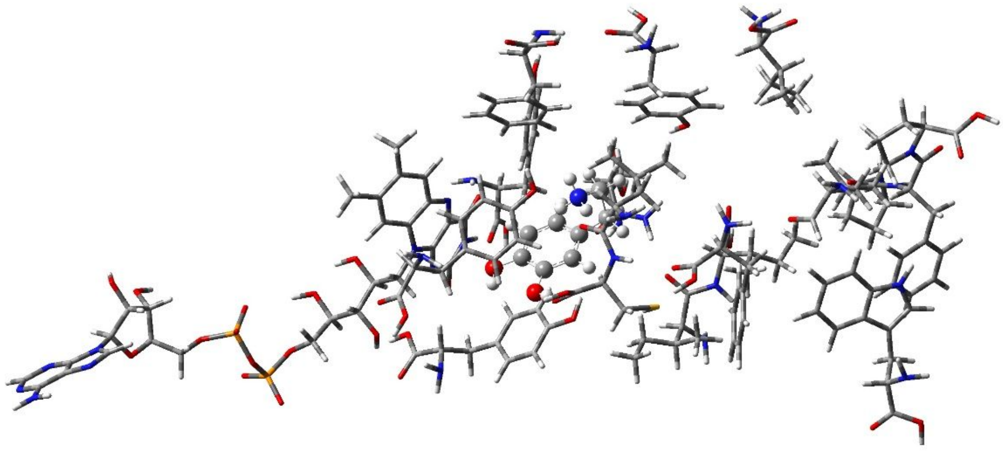

MAOB Dopamine

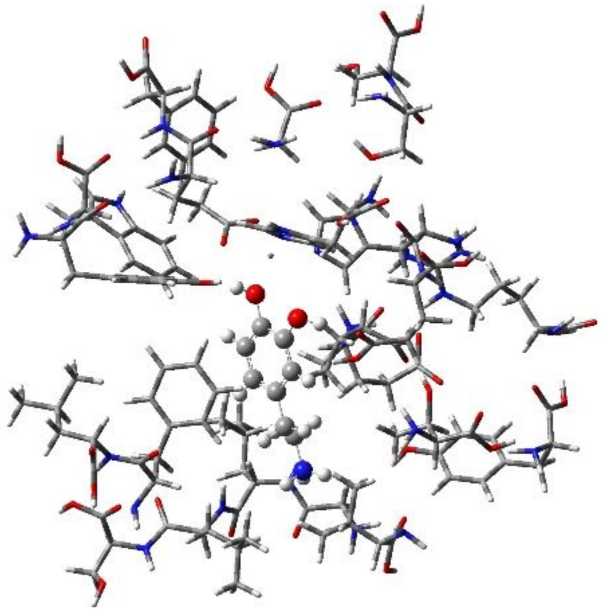

TyrOH Dopamine

Figure S5. M062X/6-31G, implicit solvent (water) optimized structures of LDOPA in each enzyme active site. Amino acid residue backbone atoms (alpha carbon and attached C and N) are fixed in the optimization and all other atoms are allowed to move during optimization.

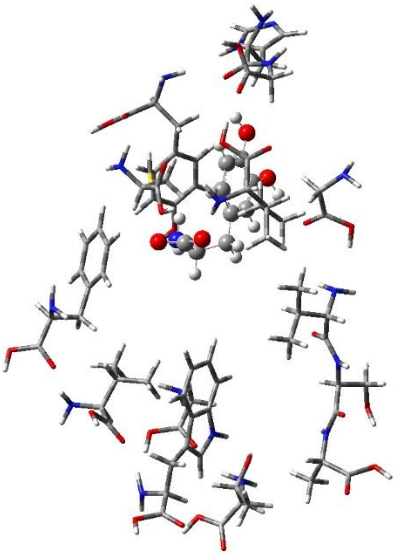

ALDH LD

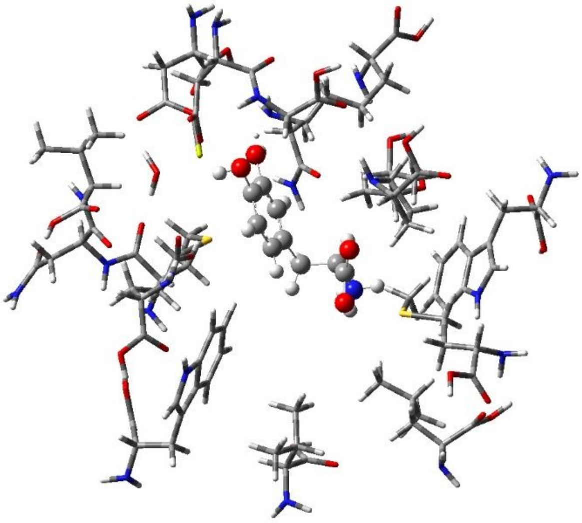

COMT LD

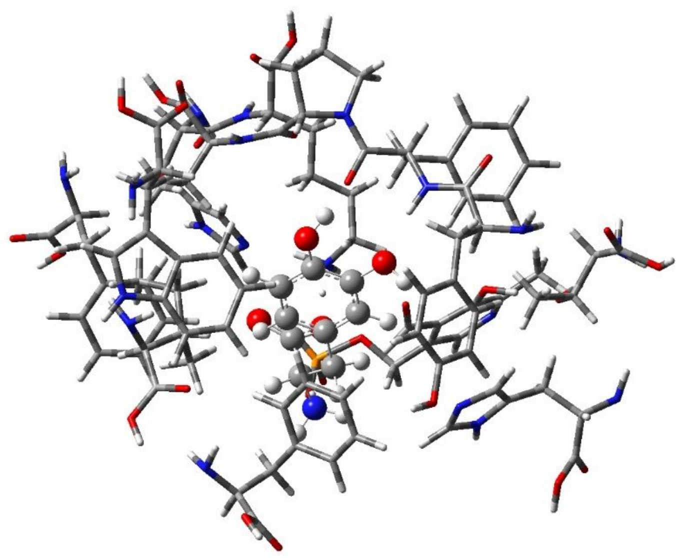

DCC LD

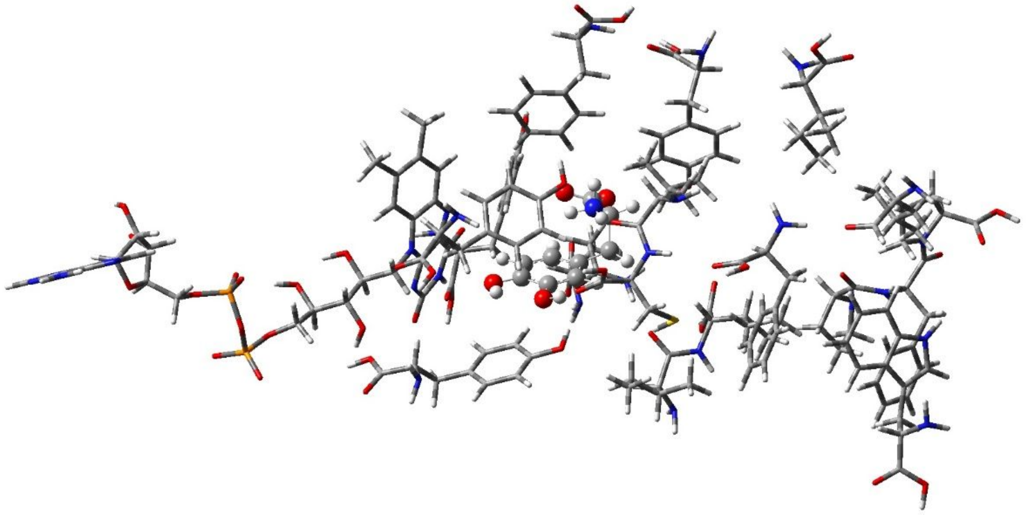

MAOB LD

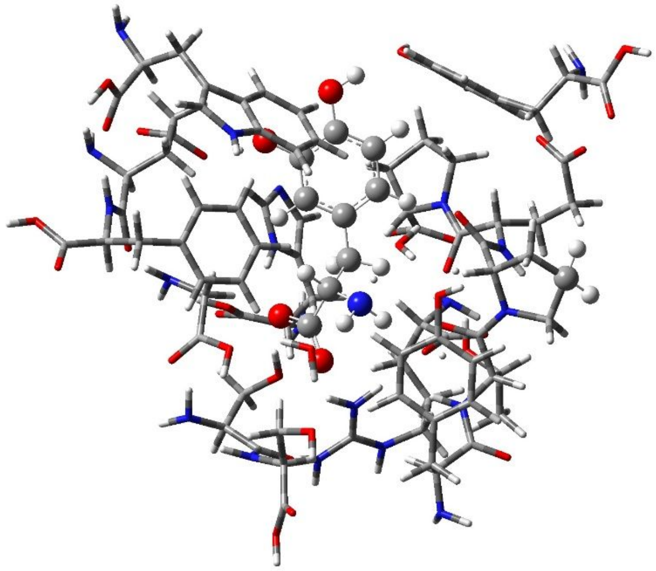

PheOH LD

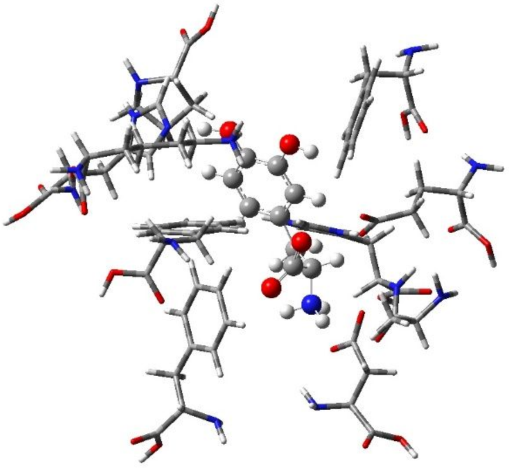

Sult1a3 LD

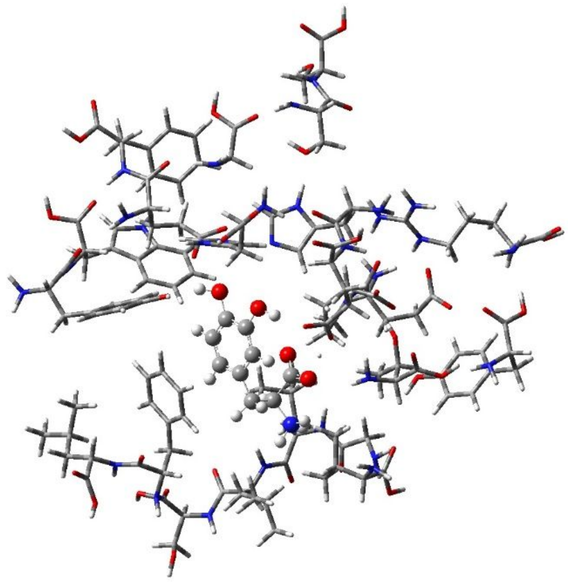

TyrOH LD

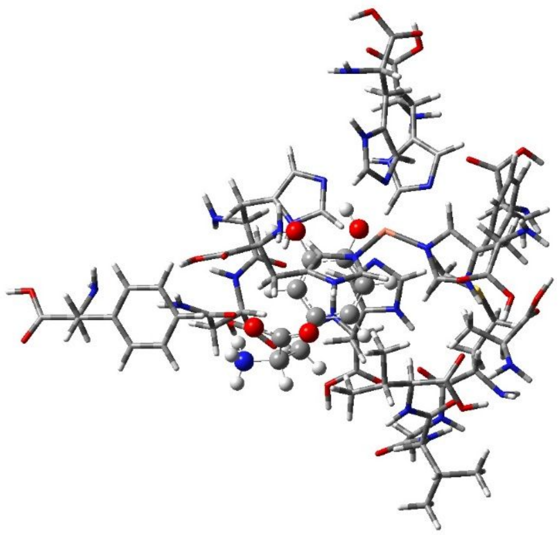

Tyrosinase LD

Figure S6. M062X/6-31G, implicit solvent (water) optimized structures of Natural substrates in their respective enzyme active sites. Amino acid residue backbone atoms (alpha carbon and attached C and N) are fixed in the optimization and all other atoms are allowed to move during optimization.

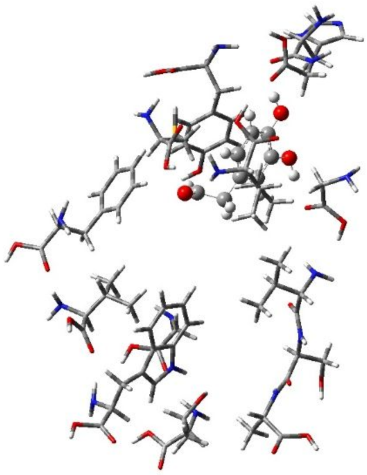

ALDH DOPAL

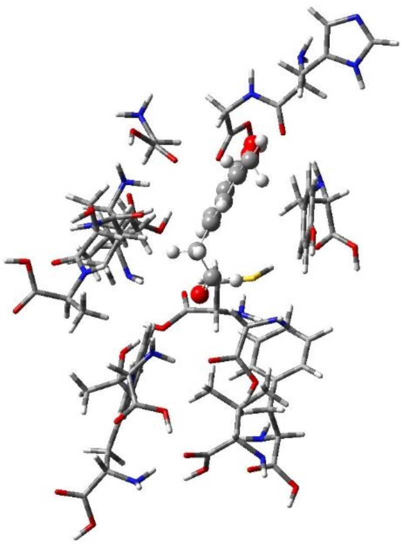

ALDH HomoVanilin

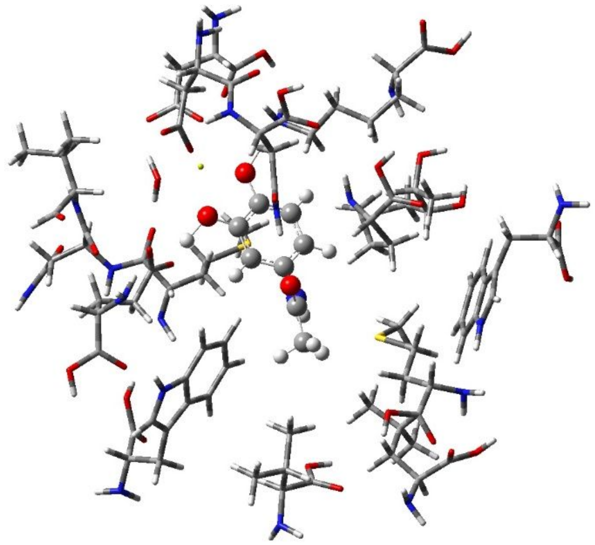

COMT 3HP

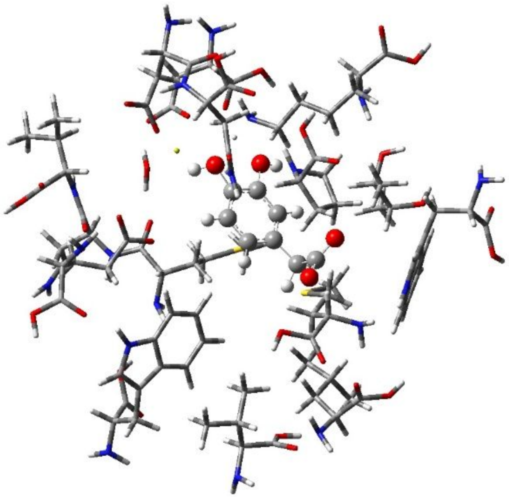

COMT DOPAC

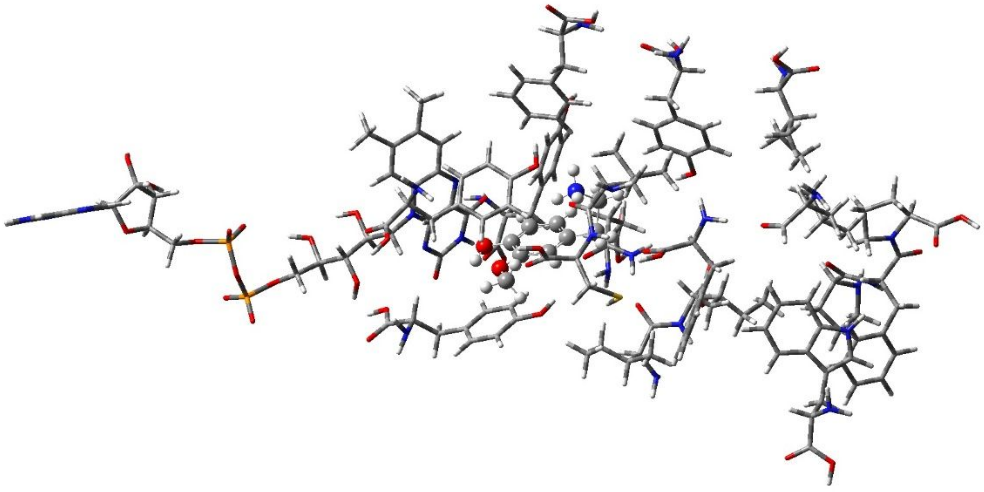

MAOB 3HT

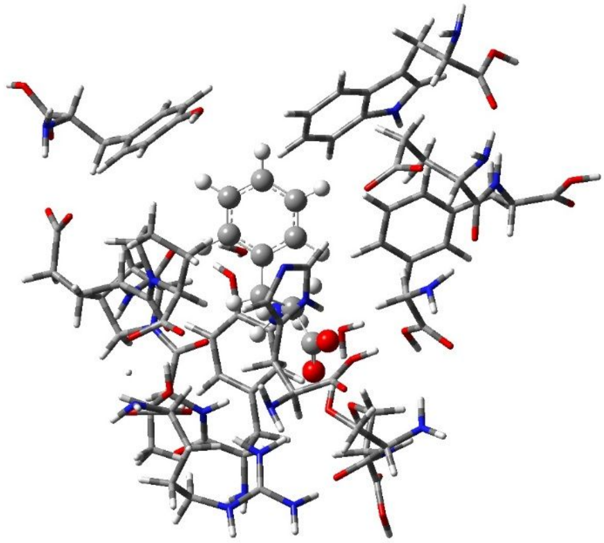

PheOH Phe

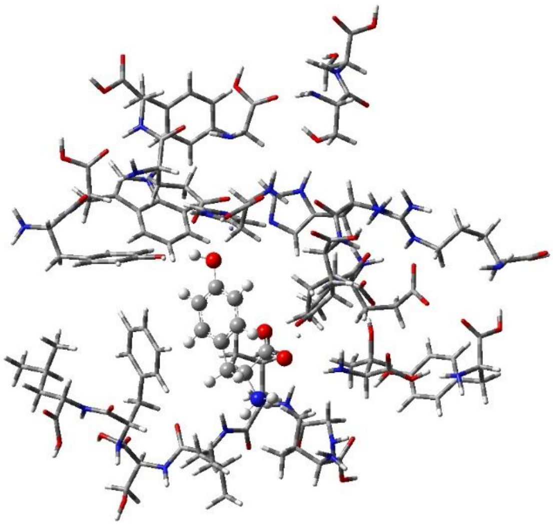

TyrOH Tyr

1. Optimized coordinates for Paracetamol in the active sites labelled below. Optimizations carried out with M062X/6-31G and implicit solvent using the PCM model in the Gaussian 16 software (see manuscript for references). Columns are: atom number, atom symbol, atom type (gaussian 16), and X, Y and Z coordinates in Angstroms.

Phenylalanine Hydroxylase: Overall Charge = -1, Total energy = -7287.26731629 Ha

1	N	0	-3.222601	9.806952	-0.080259
2	C	0	-2.316213	9.725655	-1.231843
3	C	0	-2.918419	10.438323	-2.441271
4	O	0	-3.387786	9.893416	-3.441216
5	C	0	-1.965208	8.267283	-1.599514
6	C	0	-1.076634	7.630148	-0.557758
7	C	0	-1.599840	7.168646	0.658819
8	C	0	0.306742	7.532460	-0.767566
9	C	0	-0.760319	6.645244	1.644374
10	C	0	1.151393	6.999213	0.204196
11	C	0	0.613502	6.565403	1.415436
12	O	0	1.503534	6.064044	2.357199
13	H	0	-2.760737	10.045302	0.786695
14	H	0	-3.850304	9.002592	0.015209
15	H	0	-1.397368	10.270871	-0.989193
16	H	0	-2.910101	7.717790	-1.689079
17	H	0	-1.473466	8.241457	-2.577264
18	H	0	-2.669375	7.210602	0.845995
19	H	0	0.728610	7.877388	-1.706796
20	H	0	-1.178462	6.310890	2.588925
21	H	0	2.219457	6.915139	0.042345
22	H	0	1.065484	5.806808	3.188757
23	N	0	-6.483006	-1.062703	4.910776

24	C	0	-5.689598	-1.889337	3.952045
25	C	0	-6.059672	-1.428854	2.559305
26	O	0	-7.005377	-0.664430	2.342554
27	C	0	-5.927237	-3.396625	4.141280
28	C	0	-7.305073	-3.883851	3.660259
29	C	0	-7.281135	-4.493208	2.251760
30	N	0	-6.471004	-5.715355	2.202282
31	C	0	-5.276881	-5.868583	1.605752
32	N	0	-4.580178	-4.825597	1.130204
33	N	0	-4.757136	-7.096050	1.480112
34	H	0	-6.348270	-1.385276	5.870615
35	H	0	-7.478556	-1.061926	4.675676
36	H	0	-4.635032	-1.669523	4.144771
37	H	0	-5.773767	-3.619207	5.201035
38	H	0	-5.121601	-3.932546	3.627464
39	H	0	-8.040428	-3.069024	3.663852
40	H	0	-7.689398	-4.639814	4.351483
41	H	0	-8.296202	-4.758537	1.946889
42	H	0	-6.901204	-3.787760	1.510020
43	H	0	-6.887551	-6.539642	2.614812
44	H	0	-4.836353	-3.855894	1.306002
45	H	0	-3.739539	-5.022452	0.595598
46	H	0	-3.839609	-7.199961	1.044715
47	H	0	-5.283405	-7.918473	1.729749
48	N	0	-5.548799	-4.434564	-5.240423
49	C	0	-4.706032	-3.317750	-4.848420
50	C	0	-5.503493	-2.102762	-4.374596
51	O	0	-6.599864	-1.796675	-4.905984
52	C	0	-3.857946	-2.850795	-6.066652
53	C	0	-3.073876	-1.592276	-5.772240

54	C	0	-1.885164	-1.662045	-5.031421
55	C	0	-3.563064	-0.330020	-6.129508
56	C	0	-1.202743	-0.509156	-4.651739
57	C	0	-2.892826	0.837463	-5.751532
58	C	0	-1.715341	0.733738	-5.013028
59	O	0	-0.993720	1.850679	-4.585527
60	H	0	-6.277135	-4.146950	-5.888847
61	H	0	-5.945443	-4.940289	-4.454978
62	H	0	-4.027447	-3.645972	-4.053891
63	H	0	-3.196677	-3.678410	-6.339499
64	H	0	-4.549136	-2.682294	-6.901309
65	H	0	-1.495042	-2.633656	-4.744484
66	H	0	-4.487539	-0.253059	-6.694219
67	H	0	-0.269615	-0.569753	-4.104245
68	H	0	-3.280617	1.809865	-6.039322
69	H	0	-1.238827	2.677312	-5.039657
70	N	0	-4.933246	-1.387858	-3.407268
71	C	0	-5.524313	-0.159587	-2.886623
72	C	0	-4.376346	0.786181	-2.546476
73	O	0	-3.341532	0.359980	-1.973037
74	C	0	-6.316592	-0.374637	-1.554173
75	O	0	-5.410604	-0.561871	-0.423141
76	C	0	-7.214270	-1.595007	-1.595459
77	H	0	-4.021819	-1.630436	-3.036063
78	H	0	-6.204957	0.233798	-3.642539
79	H	0	-6.907440	0.530686	-1.369840
80	H	0	-4.910282	0.228753	-0.095197
81	H	0	-6.601837	-2.497220	-1.680109
82	H	0	-7.881390	-1.546402	-2.458764
83	H	0	-7.807511	-1.651685	-0.679863

84	N	0	-4.518833	2.072865	-2.878241
85	C	0	-3.414887	2.997553	-2.563304
86	C	0	-3.291084	3.227867	-1.063542
87	O	0	-2.192865	3.533986	-0.549160
88	C	0	-3.790991	4.286356	-3.326453
89	C	0	-5.320180	4.205449	-3.476428
90	C	0	-5.598200	2.708344	-3.669760
91	H	0	-2.463299	2.572000	-2.889530
92	H	0	-3.310732	4.279081	-4.309128
93	H	0	-3.461881	5.182801	-2.797450
94	H	0	-5.811007	4.564167	-2.566098
95	H	0	-5.690596	4.798271	-4.313181
96	H	0	-5.511267	2.412026	-4.720904
97	H	0	-6.585027	2.421422	-3.301355
98	N	0	-4.437171	3.157828	-0.350280
99	C	0	-4.474647	3.452546	1.077615
100	C	0	-3.978790	2.279557	1.915585
101	O	0	-4.050096	1.088882	1.470387
102	C	0	-5.858902	3.974559	1.488492
103	C	0	-6.174330	5.300840	0.784512
104	C	0	-5.133541	6.406962	1.048583
105	O	0	-4.498238	6.369021	2.157782
106	O	0	-4.992009	7.302445	0.139644
107	H	0	-5.291044	2.863986	-0.803326
108	H	0	-3.770621	4.277985	1.239126
109	H	0	-5.860726	4.124288	2.571899
110	H	0	-6.621496	3.219274	1.259588
111	H	0	-6.254050	5.165249	-0.297791
112	H	0	-7.149674	5.667575	1.128423
113	N	0	-3.467293	2.569817	3.123433

114	C	0	-2.973578	1.512613	4.009470
115	C	0	-4.093626	0.876016	4.860975
116	O	0	-3.745291	0.030592	5.735404
117	C	0	-1.952172	2.244435	4.895258
118	C	0	-2.560499	3.652986	5.012652
119	C	0	-3.100798	3.925576	3.604124
120	H	0	-2.522566	0.706055	3.419397
121	H	0	-0.982221	2.283498	4.389118
122	H	0	-1.829958	1.739861	5.854254
123	H	0	-1.835976	4.407936	5.322179
124	H	0	-3.381729	3.648766	5.737527
125	H	0	-3.942151	4.619011	3.563964
126	H	0	-2.313155	4.328571	2.954273
127	N	0	-2.863996	-4.018264	5.490417
128	C	0	-1.883563	-3.976507	4.420020
129	C	0	-0.521188	-4.515298	4.874455
130	O	0	-0.065493	-5.632986	4.624017
131	C	0	-1.843955	-2.544191	3.797606
132	C	0	-0.472210	-2.001888	3.595128
133	N	0	0.500503	-2.606571	2.808572
134	C	0	0.190752	-1.051391	4.333046
135	C	0	1.703047	-2.015377	3.114345
136	N	0	1.547648	-1.071160	4.037879
137	H	0	-2.655162	-3.412791	6.277252
138	H	0	-3.163798	-4.943587	5.772195
139	H	0	-2.222976	-4.685659	3.664475
140	H	0	-2.359419	-1.868388	4.490549
141	H	0	-2.432664	-2.562779	2.871590
142	H	0	0.333393	-3.294710	2.065045
143	H	0	-0.222461	-0.410175	5.095300

144	H	0	2.641960	-2.302582	2.660600
145	N	0	10.573429	3.153949	2.209319
146	C	0	9.683533	2.795722	1.124882
147	C	0	10.281176	1.730264	0.210250
148	O	0	10.044344	0.520111	0.280110
149	C	0	9.203018	3.989628	0.266676
150	C	0	8.169530	3.595186	-0.745800
151	C	0	8.374441	3.110855	-2.017669
152	C	0	6.736507	3.620578	-0.543394
153	N	0	7.154232	2.847500	-2.621716
154	C	0	6.132370	3.153283	-1.741456
155	C	0	5.914818	4.005689	0.531618
156	C	0	4.742047	3.063311	-1.887298
157	C	0	4.535726	3.898892	0.400079
158	C	0	3.956570	3.429836	-0.801477
159	H	0	11.301276	3.816852	1.972447
160	H	0	10.112811	3.359549	3.084546
161	H	0	8.805597	2.319309	1.565687
162	H	0	10.076116	4.441025	-0.216255
163	H	0	8.788919	4.732777	0.957002
164	H	0	9.300305	2.948378	-2.546421
165	H	0	7.035501	2.470134	-3.547656
166	H	0	6.352540	4.378150	1.452907
167	H	0	4.295696	2.694671	-2.803888
168	H	0	3.891244	4.152599	1.233124
169	H	0	2.877712	3.341725	-0.871624
170	N	0	8.655477	-1.155773	2.743503
171	C	0	7.426573	-1.120550	1.948807
172	C	0	7.451814	-1.964246	0.671838
173	O	0	6.633460	-2.897154	0.472485

174	C	0	6.925004	0.298992	1.569343
175	C	0	6.841176	1.243543	2.780359
176	C	0	5.925485	0.675889	3.854274
177	O	0	6.411775	-0.193304	4.646519
178	O	0	4.695917	1.068025	3.868485
179	H	0	8.429405	-0.911866	3.705871
180	H	0	9.396971	-0.568763	2.365811
181	H	0	6.652138	-1.584853	2.564971
182	H	0	7.592206	0.734155	0.813636
183	H	0	5.933938	0.209920	1.101999
184	H	0	6.469511	2.217299	2.451762
185	H	0	7.838475	1.370194	3.214567
186	N	0	8.406901	-1.703655	-0.216569
187	C	0	8.449458	-2.451116	-1.467108
188	C	0	9.789519	-3.100206	-1.760141
189	O	0	10.104087	-3.546080	-2.863410
190	C	0	7.995323	-1.578124	-2.664811
191	C	0	6.500536	-1.353906	-2.657616
192	C	0	5.918391	-0.340031	-1.882535
193	C	0	5.663666	-2.195938	-3.402749
194	C	0	4.528988	-0.187410	-1.841037
195	C	0	4.276629	-2.034929	-3.377138
196	C	0	3.704511	-1.030689	-2.593492
197	H	0	9.084815	-0.962108	-0.056548
198	H	0	7.733557	-3.274550	-1.353580
199	H	0	8.293201	-2.084322	-3.586954
200	H	0	8.533063	-0.623266	-2.615057
201	H	0	6.555738	0.336358	-1.318579
202	H	0	6.106003	-2.985491	-4.003919
203	H	0	4.090537	0.595774	-1.231442

204	H	0	3.624991	-2.685331	-3.949793
205	H	0	2.628788	-0.894301	-2.590233
206	N	0	4.232007	-3.477340	1.375342
207	C	0	3.406078	-3.058541	0.261212
208	C	0	3.229573	-4.128062	-0.795668
209	O	0	2.142071	-4.676641	-1.048146
210	H	0	5.231308	-3.398378	1.205496
211	H	0	3.920405	-4.315176	1.851331
212	H	0	3.872927	-2.186965	-0.207508
213	H	0	2.395557	-2.779663	0.577788
214	N	0	1.012299	-7.276004	0.852161
215	C	0	-0.419204	-7.085640	1.107771
216	C	0	-1.272151	-6.864652	-0.151098
217	O	0	-2.507364	-6.620784	-0.045888
218	C	0	-0.496514	-5.690419	1.757558
219	O	0	-0.067133	-4.646630	0.837453
220	H	0	1.291237	-8.220877	0.609613
221	H	0	1.613712	-6.888938	1.570404
222	H	0	-0.900638	-7.832954	1.745817
223	H	0	-1.527708	-5.457832	2.014557
224	H	0	0.122241	-5.684150	2.649292
225	H	0	0.723765	-4.891418	0.296658
226	N	0	-0.623447	-6.852140	-1.312557
227	C	0	-1.186542	-6.343945	-2.544009
228	C	0	-1.386928	-7.431245	-3.580264
229	O	0	-1.176506	-7.267651	-4.786008
230	C	0	-0.264892	-5.239342	-3.069360
231	O	0	-0.806841	-4.595443	-4.240274
232	H	0	0.386650	-6.981339	-1.249369
233	H	0	-2.175106	-5.925454	-2.316278

234	H	0	0.731913	-5.645443	-3.274235
235	H	0	-0.161745	-4.474040	-2.299530
236	H	0	-0.881309	-5.246032	-4.969641
237	O	0	1.092171	-2.574914	-3.807503
238	H	0	0.763805	-2.219331	-2.950880
239	H	0	0.438887	-3.191027	-4.197933
240	O	0	1.094778	1.184400	-2.746579
241	H	0	0.836101	0.398124	-2.222775
242	H	0	0.353538	1.493650	-3.306975
243	O	0	-5.312697	-1.984222	1.591351
244	H	0	-5.430957	-1.489993	0.664696
245	O	0	-2.901478	11.792319	-2.298717
246	H	0	-3.321201	12.250291	-3.056543
247	O	0	0.062786	-3.708493	5.799618
248	H	0	0.886403	-4.090919	6.168400
249	O	0	-1.852784	-8.581367	-3.059315
250	H	0	-2.011403	-9.275151	-3.733876
251	O	0	11.174566	2.231925	-0.671480
252	H	0	11.594647	1.545179	-1.231834
253	O	0	-5.325293	1.146310	4.552787
254	H	0	-6.082811	0.013859	4.835352
255	O	0	10.605484	-3.178338	-0.679468
256	H	0	11.451172	-3.629287	-0.885047
257	O	0	4.375142	-4.523185	-1.388419
258	H	0	4.235068	-5.235164	-2.047566
259	C	0	0.093617	0.626118	0.649206
260	C	0	-0.239955	1.794944	1.346480
261	H	0	-1.256447	2.175655	1.303575
262	C	0	2.046127	2.003246	2.138546
263	C	0	-0.831638	-1.111402	-0.863692

264	O	0	2.952031	2.650364	2.935662
265	H	0	3.752058	2.066986	3.201538
266	O	0	0.243021	-1.261519	-1.527047
267	C	0	-1.963735	-2.081655	-1.030810
268	H	0	-2.022340	-2.365059	-2.085095
269	H	0	-1.720286	-2.979753	-0.448438
270	H	0	-2.920532	-1.671563	-0.706675
271	C	0	2.391130	0.879058	1.378582
272	H	0	3.411566	0.510724	1.410776
273	C	0	0.730501	2.482287	2.073773
274	H	0	0.474682	3.383173	2.619532
275	C	0	1.427495	0.193407	0.644173
276	H	0	1.711603	-0.682946	0.079097
277	N	0	-0.961381	-0.143618	0.068315
278	H	0	-1.894969	0.032910	0.430803

Tyrosine Hydroxylase: Overall Charge = 0, Total energy = -11830.4614897 Ha

1	C	0	3.474385	-9.227740	2.996989
2	O	0	5.104201	-7.925848	1.679675
3	C	0	2.120042	-8.558296	2.726513
4	C	0	2.202272	-7.034329	2.827900
5	C	0	1.574395	-9.078932	1.393683
6	H	0	3.288080	-10.310674	2.950358
7	H	0	1.457106	-8.916840	3.527388
8	H	0	2.895359	-6.620095	2.086665
9	H	0	2.553813	-6.755437	3.824161
10	H	0	1.219171	-6.578857	2.685789
11	H	0	0.548859	-8.744805	1.249710

12	H	0	1.568149	-10.174867	1.386771
13	H	0	2.182920	-8.739148	0.545280
14	C	0	3.807639	-7.102652	-2.171776
15	C	0	2.517883	-7.323671	-2.923597
16	O	0	2.335474	-8.433946	-3.474488
17	H	0	4.598394	-7.027042	-2.937328
18	H	0	3.779113	-6.146176	-1.639786
19	C	0	0.349448	-6.371754	-3.568959
20	C	0	-0.384868	-7.711140	-3.592254
21	O	0	-0.212631	-8.596535	-4.456126
22	C	0	0.386559	-5.761370	-4.981365
23	C	0	0.363945	-4.227385	-4.872951
24	C	0	0.656729	-3.557057	-6.216594
25	C	0	-1.004364	-3.816146	-4.312018
26	H	0	-0.244066	-5.718008	-2.931450
27	H	0	-0.485539	-6.090451	-5.561052
28	H	0	1.281941	-6.125631	-5.497051
29	H	0	1.141857	-3.926290	-4.155013
30	H	0	0.608073	-2.466925	-6.128211
31	H	0	-0.080646	-3.869217	-6.965475
32	H	0	1.651906	-3.827169	-6.583880
33	H	0	-1.770436	-3.890068	-5.092395
34	H	0	-1.002715	-2.795655	-3.919530
35	H	0	-1.274128	-4.510197	-3.504076
36	C	0	-2.329173	-8.903994	-2.522089
37	C	0	-3.639378	-8.708560	-3.341638
38	O	0	-3.913686	-7.602014	-3.858313
39	C	0	-2.704270	-9.010201	-1.032448

40	C	0	-1.629282	-9.650612	-0.145812
41	C	0	-1.985168	-9.411161	1.327703
42	C	0	-1.486304	-11.152037	-0.422813
43	H	0	-1.828744	-9.810549	-2.876412
44	H	0	-3.635090	-9.584136	-0.929690
45	H	0	-2.929942	-7.997109	-0.669603
46	H	0	-0.670437	-9.163149	-0.372914
47	H	0	-2.985294	-9.809091	1.539349
48	H	0	-1.990397	-8.341839	1.569461
49	H	0	-1.276755	-9.912302	1.994457
50	H	0	-2.423930	-11.669504	-0.187532
51	H	0	-0.695747	-11.581606	0.200264
52	H	0	-1.234834	-11.359118	-1.467333
53	C	0	-5.755683	-9.582988	-4.168649
54	O	0	-6.374121	-8.343408	-2.149864
55	C	0	-6.409588	-10.943137	-4.359691
56	H	0	-5.581199	-9.099806	-5.135259
57	H	0	-5.791295	-11.545579	-5.030923
58	H	0	-7.404428	-10.832760	-4.802014
59	C	0	-6.369397	-7.548301	1.567514
60	C	0	-5.910670	-6.446620	0.579401
61	C	0	-5.697704	-5.096959	1.219801
62	C	0	-6.573272	-4.038765	0.944718
63	C	0	-6.398851	-2.787821	1.544813
64	C	0	-4.646065	-4.885060	2.122646
65	C	0	-5.348159	-2.588854	2.441935
66	C	0	-7.464218	-7.000810	2.458138
67	O	0	-7.249652	-6.689567	3.658954

68	H	0	-5.538090	-7.812480	2.233602
69	H	0	-6.676823	-6.372106	-0.200588
70	H	0	-4.994052	-6.798748	0.091922
71	H	0	-7.394461	-4.197841	0.251400
72	H	0	-7.080211	-1.973795	1.314141
73	H	0	-3.960729	-5.697824	2.347037
74	H	0	-5.201168	-1.619351	2.903806
75	C	0	-9.755678	-6.348982	2.666062
76	O	0	-10.668205	-7.055195	4.795525
77	C	0	-10.968270	-6.047102	1.773974
78	C	0	-10.691519	-4.963917	0.715123
79	C	0	-11.950373	-4.752556	-0.135558
80	C	0	-10.241945	-3.643478	1.353499
81	H	0	-9.455791	-5.447289	3.207338
82	H	0	-11.289341	-6.974297	1.281052
83	H	0	-11.792330	-5.717544	2.419157
84	H	0	-9.886548	-5.321132	0.057269
85	H	0	-11.765440	-4.018830	-0.925533
86	H	0	-12.771315	-4.380251	0.488356
87	H	0	-12.274688	-5.687085	-0.603861
88	H	0	-10.104041	-2.875478	0.584782
89	H	0	-9.292557	-3.747296	1.888057
90	H	0	-10.999809	-3.284391	2.060520
91	C	0	5.669741	-4.671308	3.216961
92	O	0	6.802725	-3.580509	5.128143
93	C	0	5.798337	-3.869698	1.893644
94	C	0	4.812445	-4.351538	0.836400
95	H	0	5.763001	-5.736262	2.976881

96	H	0	6.824177	-3.987982	1.520044
97	H	0	4.981826	-3.814279	-0.103300
98	H	0	3.784639	-4.164795	1.164835
99	H	0	4.933979	-5.425666	0.657283
100	C	0	11.490731	-4.018047	1.812306
101	O	0	10.535794	-1.734182	1.888191
102	C	0	11.587499	-4.791020	0.459563
103	C	0	10.302709	-4.709526	-0.332022
104	C	0	10.071665	-3.660321	-1.237207
105	C	0	9.286275	-5.656883	-0.147699
106	C	0	8.873782	-3.555252	-1.941871
107	C	0	8.075305	-5.563507	-0.837336
108	C	0	7.877528	-4.511805	-1.732227
109	H	0	12.430995	-4.169407	2.353429
110	H	0	11.811496	-5.835007	0.702713
111	H	0	12.424722	-4.394349	-0.119364
112	H	0	10.850226	-2.920289	-1.400760
113	H	0	9.445024	-6.486866	0.534342
114	H	0	8.708873	-2.753741	-2.659043
115	H	0	7.301547	-6.310574	-0.685349
116	C	0	12.251170	1.322620	-2.232956
117	O	0	14.645088	1.854572	-1.937722
118	C	0	11.473728	2.587830	-2.701543
119	C	0	10.223052	2.908028	-1.888095
120	C	0	9.136059	1.807533	-1.899726
121	H	0	11.871571	0.441118	-2.756005
122	H	0	12.135238	3.460858	-2.640219
123	H	0	11.215895	2.453068	-3.757005

124	H	0	9.799260	3.829074	-2.301677
125	H	0	10.536815	3.135749	-0.861221
126	H	0	9.344599	1.049646	-2.661569
127	H	0	9.080407	1.293769	-0.930842
128	C	0	4.725849	0.034761	-5.222229
129	C	0	4.810492	0.502045	-3.753492
130	O	0	5.511471	1.500397	-3.426867
131	C	0	5.494619	-1.328159	-5.317485
132	C	0	6.975359	-1.234478	-5.756873
133	O	0	7.893788	0.807859	-4.840347
134	H	0	3.670568	-0.127946	-5.459337
135	H	0	5.457368	-1.838082	-4.347671
136	H	0	4.980586	-1.966399	-6.045393
137	H	0	7.014199	-0.756869	-6.740091
138	H	0	7.352417	-2.256046	-5.855934
139	N	0	3.887863	-0.000175	-2.927356
140	C	0	3.900158	0.367582	-1.521252
141	O	0	6.053290	-0.768339	-1.676761
142	C	0	2.668111	-0.349041	-0.947034
143	C	0	2.589948	-1.617971	-1.811831
144	C	0	2.953109	-1.109574	-3.212369
145	H	0	3.881390	1.452737	-1.382117
146	H	0	2.797963	-0.561671	0.114501
147	H	0	1.769475	0.264474	-1.083194
148	H	0	1.620986	-2.115034	-1.783530
149	H	0	3.342071	-2.340733	-1.475936
150	H	0	3.416718	-1.880050	-3.829529
151	H	0	2.069193	-0.727955	-3.738992

152	C	0	4.690734	4.128784	2.629745
153	C	0	3.834804	3.499204	3.735982
154	O	0	2.846801	4.117146	4.205901
155	C	0	4.232663	3.465378	1.297347
156	C	0	2.750009	3.470293	1.123410
157	C	0	1.802162	2.468067	1.145818
158	C	0	0.724988	4.364858	0.930961
159	N	0	0.536677	3.039501	1.013216
160	H	0	4.450614	5.194241	2.628873
161	H	0	4.595363	2.430091	1.281040
162	H	0	4.718112	3.991100	0.467750
163	H	0	1.944768	1.404753	1.245733
164	H	0	-0.061231	5.098308	0.822490
165	C	0	3.365520	1.553438	5.179469
166	O	0	2.722005	2.556712	7.304094
167	C	0	3.785511	0.079122	5.223839
168	C	0	3.554328	-0.633441	3.894741
169	O	0	1.155243	-0.447561	4.286703
170	H	0	2.299768	1.651151	4.965098
171	H	0	4.846436	0.021953	5.487602
172	H	0	3.220145	-0.420502	6.016183
173	H	0	4.051548	-1.608372	3.896127
174	H	0	4.007918	-0.100909	3.049069
175	C	0	-0.693442	3.980624	7.141266
176	O	0	0.066162	4.300617	9.394812
177	C	0	-0.945984	2.682467	6.339537
178	C	0	-1.449551	2.963663	4.957702
179	C	0	-0.829647	2.949201	3.734463

180	C	0	-2.925138	3.467573	3.344133
181	N	0	-1.759048	3.255244	2.737649
182	H	0	-1.567141	4.624314	6.936080
183	H	0	-1.663200	2.055843	6.877101
184	H	0	-0.005387	2.124987	6.274978
185	H	0	0.202531	2.747295	3.505166
186	H	0	-3.858580	3.693494	2.856681
187	C	0	-10.778662	2.317053	0.295080
188	O	0	-8.964586	3.983757	0.325582
189	C	0	-10.146865	0.896070	0.279803
190	C	0	-8.730389	0.856722	0.833375
191	C	0	-8.487768	0.664147	2.199315
192	C	0	-7.618951	1.054063	-0.003710
193	C	0	-7.187814	0.672367	2.714840
194	C	0	-6.313482	1.077477	0.493412
195	C	0	-6.106907	0.885874	1.858708
196	H	0	-10.867048	2.648167	1.338441
197	H	0	-10.153879	0.542832	-0.757856
198	H	0	-10.795368	0.230685	0.860408
199	H	0	-9.321643	0.502973	2.875056
200	H	0	-7.784737	1.200201	-1.066912
201	H	0	-7.024511	0.516215	3.776237
202	H	0	-5.462166	1.263156	-0.152247
203	C	0	-8.751927	4.045066	-2.408501
204	O	0	-7.976728	6.327888	-2.143043
205	C	0	-8.785067	3.762797	-3.930834
206	C	0	-7.466303	4.056798	-4.570376
207	C	0	-7.161939	5.024078	-5.494961

208	C	0	-6.244405	3.323049	-4.319603
209	C	0	-5.237665	3.892971	-5.143847
210	C	0	-5.919937	2.228725	-3.497723
211	C	0	-3.928276	3.392871	-5.168629
212	C	0	-4.620821	1.735892	-3.511372
213	C	0	-3.634756	2.314145	-4.342232
214	H	0	-7.747557	3.802486	-2.038019
215	H	0	-9.022463	2.697676	-4.047273
216	H	0	-9.594709	4.328803	-4.402271
217	H	0	-7.799238	5.772373	-5.938399
218	H	0	-6.677580	1.761627	-2.876674
219	H	0	-3.172900	3.827177	-5.813937
220	H	0	-4.357908	0.890484	-2.885598
221	H	0	-2.631151	1.904984	-4.338449
222	C	0	-5.251681	5.586773	0.478692
223	C	0	-5.153765	7.044087	0.088810
224	O	0	-4.076747	7.685830	0.151611
225	C	0	-5.283081	4.737539	-0.809059
226	C	0	-3.902567	4.663495	-1.450149
227	O	0	-3.385370	2.941616	0.190949
228	H	0	-4.334946	5.368515	1.040235
229	H	0	-5.615137	3.729695	-0.537188
230	H	0	-6.006613	5.164653	-1.512375
231	H	0	-3.484909	5.658789	-1.628558
232	H	0	-3.958372	4.156024	-2.422522
233	C	0	-6.191305	9.064362	-0.767730
234	O	0	-7.291690	11.209159	-0.470188
235	C	0	-6.216133	9.190667	-2.320439

236	C	0	-4.957932	8.657081	-2.957307
237	C	0	-4.889881	7.350463	-3.457140
238	C	0	-3.822204	9.474951	-3.036529
239	C	0	-3.701279	6.877216	-4.022934
240	C	0	-2.637848	9.003252	-3.600087
241	C	0	-2.574378	7.698162	-4.095382
242	H	0	-5.232126	9.448033	-0.407508
243	H	0	-6.338057	10.249347	-2.567908
244	H	0	-7.092663	8.646053	-2.685710
245	H	0	-5.767266	6.708362	-3.401042
246	H	0	-3.865725	10.485608	-2.641045
247	H	0	-3.651700	5.859936	-4.392544
248	H	0	-1.766758	9.646804	-3.640903
249	H	0	-1.655780	7.324173	-4.533479
250	C	0	-0.936835	7.537023	-0.359652
251	O	0	0.591260	9.379910	0.001374
252	H	0	-0.030657	6.935605	-0.461800
253	H	0	-1.610284	7.313430	-1.189460
254	C	0	3.752577	9.122577	2.100917
255	C	0	4.269710	9.675793	0.768751
256	O	0	5.260427	9.125809	0.227611
257	C	0	3.710745	7.600919	1.963933
258	H	0	4.467234	9.401821	2.880067
259	H	0	4.690015	7.206689	1.695821
260	H	0	3.358486	7.156238	2.900550
261	C	0	4.135896	11.259357	-0.999979
262	O	0	3.457999	13.521067	-0.483290
263	C	0	3.130048	10.931131	-2.110122

264	H	0	5.094358	10.793380	-1.219028
265	H	0	3.084747	9.843980	-2.231317
266	H	0	2.140727	11.295671	-1.810217
267	Fe	0	-1.464009	2.649852	0.850821
268	C	0	-0.746365	-3.013710	0.985811
269	C	0	-1.172274	-2.612271	2.261966
270	C	0	-0.749684	-2.097319	-0.075448
271	C	0	-1.632970	-1.316255	2.475521
272	C	0	-1.203816	-0.799100	0.146320
273	C	0	-1.658131	-0.426046	1.406578
274	O	0	-2.136517	0.872037	1.594586
275	H	0	-1.147833	-3.319354	3.084062
276	H	0	-0.427475	-2.405885	-1.057403
277	H	0	-1.964493	-0.996843	3.455805
278	H	0	-1.231643	-0.081717	-0.665464
279	H	0	-3.116532	0.931369	1.781231
280	N	0	12.282380	1.047832	-0.788870
281	H	0	11.362461	1.000289	-0.363181
282	H	0	12.905624	1.667437	-0.278728
283	C	0	13.713097	1.578403	-2.689171
284	O	0	13.824797	1.536563	-4.039447
285	H	0	14.725040	1.758752	-4.356920
286	C	0	4.308108	12.755167	-0.937983
287	O	0	5.490869	13.169873	-1.444427
288	H	0	5.583591	14.145816	-1.443397
289	N	0	2.431987	9.665326	2.450336
290	H	0	1.664925	9.514022	1.803492
291	H	0	2.179598	9.610508	3.426099

292	C	0	-0.529538	8.997801	-0.393222
293	O	0	-1.491745	9.866241	-0.753794
294	H	0	-1.212705	10.803160	-0.670336
295	N	0	-1.650003	7.269851	0.897626
296	H	0	-2.649555	7.435447	0.852743
297	H	0	-1.156363	7.543463	1.737786
298	N	0	-8.637910	-6.825571	1.888717
299	H	0	-8.789801	-7.280292	0.994384
300	N	0	-6.877173	-8.677237	0.773810
301	H	0	-6.513332	-8.733535	-0.171666
302	H	0	-6.899686	-9.566187	1.257131
303	C	0	-10.119030	-7.354445	3.735311
304	O	0	-9.849614	-8.638764	3.386165
305	H	0	-10.118263	-9.276215	4.079864
306	O	0	-6.477175	-11.521146	-3.040558
307	H	0	-6.828680	-12.429692	-3.059230
308	N	0	-4.462486	-9.761236	-3.474163
309	H	0	-4.338305	-10.629954	-2.968635
310	N	0	-1.408517	-7.774601	-2.734615
311	H	0	-1.635487	-6.956809	-2.183869
312	N	0	1.667134	-6.307135	-2.943291
313	H	0	1.807649	-5.480308	-2.367496
314	N	0	4.062564	-8.160112	-1.183008
315	H	0	4.126589	-9.081011	-1.603141
316	H	0	4.820392	-7.936752	-0.547571
317	C	0	-6.625630	-8.656494	-3.305232
318	O	0	-7.759794	-8.188923	-3.891625
319	H	0	-7.883048	-8.419675	-4.832753

320	O	0	-4.787051	0.893873	2.343849
321	H	0	-4.754420	0.876145	3.319356
322	N	0	-12.048742	2.347038	-0.445576
323	H	0	-12.601176	1.502587	-0.348484
324	H	0	-12.601203	3.181579	-0.286305
325	N	0	-5.824037	4.930473	-5.845780
326	H	0	-5.355959	5.533559	-6.502709
327	N	0	-9.698591	3.240374	-1.694061
328	H	0	-10.473584	2.787012	-2.168028
329	O	0	3.603919	11.594485	-3.295879
330	H	0	2.987823	11.479250	-4.041375
331	O	0	2.815222	7.232988	0.875253
332	H	0	2.063258	7.854826	0.760893
333	N	0	3.647553	10.729215	0.252551
334	H	0	2.857901	11.139474	0.738159
335	N	0	2.041584	4.651905	0.984357
336	H	0	2.424152	5.613701	0.912287
337	N	0	6.115296	3.827119	2.831341
338	H	0	6.642574	3.647722	1.985855
339	H	0	6.600815	4.433313	3.479766
340	N	0	4.131694	2.245513	4.129124
341	H	0	5.042559	1.887649	3.857428
342	C	0	3.613234	2.222285	6.520924
343	O	0	4.930751	2.386393	6.783761
344	H	0	5.100980	2.804201	7.653755
345	C	0	2.100486	-0.819588	3.586282
346	O	0	1.908906	-1.463392	2.399765
347	N	0	-2.773319	3.303547	4.686432

348	H	0	-3.509980	3.402016	5.369855
349	N	0	0.473218	4.682937	6.587901
350	H	0	0.653858	5.576828	7.031676
351	H	0	1.309380	4.103966	6.544180
352	C	0	-0.742504	3.829894	8.594046
353	O	0	-1.854004	3.153056	9.006088
354	H	0	-1.915953	3.098306	9.981774
355	O	0	6.702324	-4.373020	-2.460311
356	H	0	6.064634	-5.086462	-2.279516
357	N	0	10.388984	-4.514099	2.650451
358	H	0	10.607393	-5.436776	3.019773
359	H	0	9.513374	-4.540373	2.132308
360	C	0	11.417590	-2.499743	1.505255
361	O	0	12.441181	-2.099517	0.701680
362	H	0	12.383121	-1.157909	0.402797
363	C	0	4.475759	-8.967404	1.885182
364	O	0	4.632769	-10.071020	1.104368
365	H	0	5.295601	-9.949842	0.393816
366	N	0	4.030192	-8.836383	4.299337
367	H	0	3.321102	-8.700947	5.010813
368	H	0	4.784308	-9.428885	4.625915
369	N	0	7.811014	2.319967	-2.256695
370	H	0	7.332734	1.848414	-3.038750
371	C	0	7.178953	3.297857	-1.611591
372	N	0	5.952510	3.677456	-2.005004
373	H	0	5.512642	4.506518	-1.640703
374	H	0	5.456119	3.058563	-2.648439
375	N	0	7.753349	3.933708	-0.565497

376	H	0	7.309680	4.733313	-0.141306
377	H	0	8.685003	3.712557	-0.256012
378	N	0	5.298312	0.963870	-6.207530
379	H	0	6.260666	1.185489	-5.926307
380	H	0	4.745096	1.814310	-6.287702
381	C	0	7.929708	-0.479541	-4.816440
382	O	0	8.722397	-1.164824	-4.085957
383	C	0	5.205979	-0.219013	-0.920948
384	O	0	5.265838	-0.190112	0.375864
385	C	0	6.836272	-4.287982	4.125464
386	O	0	7.991758	-4.812420	3.637220
387	H	0	8.826368	-4.425790	3.996232
388	N	0	4.395372	-4.399674	3.897601
389	H	0	3.925822	-3.578599	3.534585
390	H	0	3.786016	-5.201259	3.976832
391	O	0	5.549654	-2.486967	2.249214
392	H	0	5.537352	-1.854240	1.484340
393	C	0	-2.966083	3.910635	-0.566994
394	N	0	-6.434651	5.269996	1.291963
395	H	0	-7.240668	4.887715	0.804467
396	H	0	-6.660097	5.923410	2.029580
397	N	0	-6.261375	7.664192	-0.358163
398	H	0	-7.116115	7.147612	-0.526457
399	C	0	-7.266747	10.005487	-0.221627
400	O	0	-8.201591	9.383750	0.539539
401	H	0	-8.887251	9.999583	0.873681
402	O	0	-1.704206	4.189848	-0.507486
403	C	0	-9.746322	3.274752	-0.351870

404	C	0	-8.911715	5.522394	-2.141596
405	O	0	-10.198567	5.897026	-1.974104
406	H	0	-10.301840	6.861236	-1.830400
407	C	0	-4.477444	-3.643114	2.734926
408	H	0	-3.668100	-3.494180	3.440119
409	H	0	0.956571	-1.609521	2.202779
410	N	0	-0.365171	-4.362330	0.838941
411	H	0	-0.628076	-4.968232	1.605183
412	C	0	0.537873	-6.414722	-0.037348
413	H	0	1.609162	-6.614035	0.062832
414	H	0	0.019353	-6.818504	0.829840
415	H	0	0.194957	-6.953134	-0.922364
416	C	0	0.305796	-4.940929	-0.190254
417	O	0	0.716289	-4.303987	-1.194711

Tyrosinase: Overall Charge = +2, Total energy = -8953.00642101 Ha

1	C	0	6.170561	2.853043	5.886630
2	O	0	4.270297	2.098922	7.143446
3	C	0	6.356139	2.073614	4.561066
4	C	0	5.026604	1.768166	3.953088
5	N	0	4.347441	0.558211	4.058637
6	C	0	4.150831	2.620925	3.327857
7	C	0	3.116378	0.691968	3.518008
8	N	0	2.966926	1.943327	3.065274
9	H	0	6.933244	1.162244	4.744891
10	H	0	6.938601	2.709566	3.890237
11	H	0	4.690576	-0.309303	4.455166

12	H	0	4.282488	3.657005	3.065033
13	H	0	2.397630	-0.107308	3.463683
14	H	0	5.501226	3.695286	5.694967
15	C	0	-3.623847	4.639462	5.110696
16	O	0	-5.001745	5.267319	6.922992
17	C	0	-3.330975	3.107621	5.139240
18	C	0	-1.940354	2.798294	4.696715
19	N	0	-0.898386	2.626212	5.598605
20	C	0	-1.365194	2.679763	3.456682
21	C	0	0.253867	2.424174	4.925889
22	N	0	0.003952	2.452940	3.612009
23	H	0	-3.677843	4.956251	4.066812
24	H	0	-3.471985	2.744444	6.163436
25	H	0	-4.058048	2.589032	4.507853
26	H	0	-0.984546	2.680976	6.603592
27	H	0	-1.822290	2.755277	2.485594
28	H	0	1.215467	2.277363	5.387429
29	C	0	2.627383	8.885134	-0.116257
30	O	0	4.401043	9.498158	-1.570167
31	C	0	2.509434	7.471822	-0.716904
32	C	0	2.188651	6.426795	0.308785
33	N	0	1.578462	6.734097	1.518637
34	C	0	2.372837	5.064027	0.328554
35	C	0	1.408963	5.604221	2.233990
36	N	0	1.878987	4.564682	1.535071
37	H	0	3.290920	8.844774	0.756023
38	H	0	1.740131	7.486929	-1.500012
39	H	0	3.454698	7.219402	-1.203287

40	H	0	1.279673	7.657984	1.798464
41	H	0	2.808956	4.417380	-0.416815
42	H	0	0.957158	5.568144	3.210372
43	C	0	-12.104542	0.847426	-0.066456
44	O	0	-14.458483	0.420002	0.171117
45	C	0	-11.885541	1.403237	1.352655
46	C	0	-10.473304	1.171165	1.841232
47	C	0	-10.055907	-0.112431	2.221001
48	C	0	-9.558704	2.228332	1.925833
49	C	0	-8.755470	-0.334183	2.676186
50	C	0	-8.255993	2.012187	2.387774
51	C	0	-7.850928	0.728369	2.763243
52	H	0	-11.925256	-0.234212	-0.049510
53	H	0	-12.606954	0.920452	2.019101
54	H	0	-12.108538	2.476951	1.349705
55	H	0	-10.757245	-0.940137	2.166461
56	H	0	-9.871531	3.228458	1.641881
57	H	0	-8.448741	-1.332509	2.966620
58	H	0	-7.565373	2.844691	2.465053
59	H	0	-6.841972	0.558160	3.121060
60	C	0	-4.799166	1.205221	-5.166643
61	C	0	-4.650378	-0.097223	-4.378113
62	O	0	-3.647297	-0.782697	-4.517603
63	C	0	-4.709229	2.424916	-4.214441
64	C	0	-3.342595	2.553645	-3.648928
65	N	0	-2.863150	1.746155	-2.629353
66	C	0	-2.255733	3.269821	-4.089495
67	C	0	-1.522818	1.981045	-2.496350

68	N	0	-1.123246	2.903026	-3.369487
69	H	0	-3.913098	1.222347	-5.816080
70	H	0	-4.932062	3.324226	-4.796632
71	H	0	-5.475557	2.340936	-3.436356
72	H	0	-3.415247	1.099598	-2.080302
73	H	0	-2.217231	4.000367	-4.879189
74	H	0	-0.889063	1.391409	-1.855027
75	N	0	-5.641733	-0.424913	-3.550977
76	C	0	-5.628516	-1.680299	-2.791719
77	O	0	-4.754866	-3.822659	-3.490216
78	C	0	-6.860533	-1.672558	-1.873468
79	C	0	-6.756515	-0.502746	-0.892914
80	O	0	-5.649061	-0.206198	-0.380702
81	H	0	-6.509470	0.095660	-3.624941
82	H	0	-4.721914	-1.718945	-2.182987
83	H	0	-6.894689	-2.600230	-1.292783
84	H	0	-7.777229	-1.620294	-2.468654
85	C	0	-1.381759	-4.395679	-3.987742
86	O	0	-0.833145	-6.699887	-4.013335
87	C	0	-1.476879	-3.825958	-2.537578
88	C	0	-0.642111	-2.601374	-2.397442
89	N	0	0.710136	-2.667413	-2.091633
90	C	0	-0.907192	-1.265719	-2.598539
91	C	0	1.207151	-1.397480	-2.102547
92	N	0	0.253982	-0.516986	-2.402107
93	H	0	-0.335867	-4.361198	-4.302675
94	H	0	-1.126048	-4.580947	-1.825016
95	H	0	-2.530263	-3.610984	-2.332696

96	H	0	1.192254	-3.541807	-1.867754
97	H	0	-1.844472	-0.814183	-2.883380
98	H	0	2.234311	-1.155074	-1.882172
99	C	0	3.232900	-6.119895	-1.978085
100	C	0	1.817446	-6.261079	-1.451036
101	O	0	1.064696	-5.288438	-1.382974
102	C	0	3.239321	-5.951900	-3.505359
103	C	0	4.375629	-5.029099	-3.944132
104	S	0	4.053956	-3.232148	-3.479096
105	C	0	2.807826	-2.804749	-4.807110
106	H	0	3.566230	-5.157274	-1.560649
107	H	0	2.276566	-5.547845	-3.838615
108	H	0	3.347309	-6.936260	-3.978390
109	H	0	5.309432	-5.289089	-3.439383
110	H	0	4.535968	-5.062953	-5.023170
111	H	0	3.280858	-2.865220	-5.787331
112	H	0	2.473748	-1.785404	-4.613008
113	H	0	1.948541	-3.473777	-4.752982
114	N	0	1.464442	-7.477159	-1.070537
115	C	0	0.144516	-7.773375	-0.568450
116	C	0	-0.084795	-7.380557	0.885852
117	O	0	-1.233460	-7.392741	1.331075
118	H	0	2.191458	-8.185973	-1.127457
119	H	0	-0.046998	-8.844128	-0.661686
120	H	0	-0.606481	-7.242880	-1.160545
121	N	0	0.955358	-7.052476	1.625905
122	C	0	0.766055	-6.642151	3.004005
123	C	0	1.402704	-5.308332	3.372298

124	O	0	2.532373	-5.039535	3.038854
125	C	0	1.265799	-7.735481	3.996845
126	C	0	0.415621	-9.001500	3.846020
127	C	0	2.754320	-8.055680	3.814368
128	H	0	1.888064	-7.033210	1.233873
129	H	0	-0.315566	-6.561000	3.138526
130	H	0	1.115774	-7.325648	5.005674
131	H	0	0.539696	-9.423848	2.842485
132	H	0	-0.646581	-8.791423	4.000379
133	H	0	0.730119	-9.757925	4.570893
134	H	0	3.379769	-7.166679	3.922951
135	H	0	2.934933	-8.490527	2.824509
136	H	0	3.067080	-8.793375	4.559188
137	N	0	0.643083	-4.499930	4.096574
138	C	0	1.099896	-3.176519	4.516945
139	O	0	3.387154	-2.466880	4.485366
140	C	0	0.052028	-2.500189	5.427643
141	C	0	0.508488	-1.074076	5.763367
142	C	0	-1.322032	-2.445328	4.748695
143	H	0	-0.265841	-4.799623	4.418112
144	H	0	1.188497	-2.551661	3.616799
145	H	0	-0.016509	-3.084637	6.354680
146	H	0	0.496310	-0.458767	4.854440
147	H	0	1.514249	-1.040120	6.195268
148	H	0	-0.180369	-0.617894	6.479053
149	H	0	-1.249848	-1.943841	3.776428
150	H	0	-2.018897	-1.877063	5.371327
151	H	0	-1.771470	-3.428151	4.579962

152	C	0	4.869887	-3.936332	0.805849
153	O	0	6.374212	-3.645852	-1.027846
154	C	0	3.661892	-3.002437	0.694789
155	H	0	4.677374	-4.810581	0.165766
156	H	0	3.486951	-2.700310	-0.343569
157	H	0	3.811424	-2.116933	1.321707
158	H	0	2.785580	-3.551107	1.050960
159	C	0	5.771451	-0.301942	-4.741600
160	O	0	4.813014	1.815723	-5.277467
161	C	0	6.515524	-0.485203	-3.385138
162	C	0	6.207077	0.417294	-2.216341
163	C	0	6.754172	1.705906	-2.144007
164	C	0	5.496870	-0.075180	-1.114178
165	C	0	6.600328	2.479114	-0.992855
166	C	0	5.355004	0.689412	0.045500
167	C	0	5.907853	1.969356	0.107966
168	H	0	4.714706	-0.565703	-4.636962
169	H	0	6.340450	-1.525056	-3.087755
170	H	0	7.583791	-0.393606	-3.621183
171	H	0	7.328281	2.090602	-2.983192
172	H	0	5.076278	-1.076799	-1.169191
173	H	0	7.037303	3.470892	-0.947432
174	H	0	4.827810	0.281526	0.903109
175	H	0	5.808685	2.562463	1.010435
176	C	0	2.504826	4.759581	-6.041168
177	O	0	1.755520	6.938211	-6.734579
178	C	0	3.139722	5.021171	-4.653523
179	C	0	2.890410	3.942079	-3.652974

180	N	0	1.696334	3.276483	-3.425230
181	C	0	3.777560	3.415202	-2.739432
182	C	0	1.904757	2.377242	-2.413842
183	N	0	3.158050	2.439438	-1.966046
184	H	0	1.421619	4.660964	-5.898573
185	H	0	2.754769	5.983757	-4.292254
186	H	0	4.219423	5.132906	-4.765598
187	H	0	0.753425	3.409930	-3.799347
188	H	0	4.819017	3.656467	-2.617010
189	H	0	1.164850	1.652019	-2.111112
190	Cu	0	1.385479	2.756658	2.225833
191	C	0	-0.797913	-1.826953	0.980877
192	C	0	-1.447508	0.481903	0.612395
193	C	0	0.538339	-1.429111	1.066978
194	C	0	-0.110702	0.848721	0.698685
195	O	0	0.272012	2.213131	0.658185
196	H	0	-2.216216	1.229999	0.452745
197	H	0	1.307313	-2.171378	1.237789
198	H	0	-0.050913	2.749509	-0.089837
199	N	0	1.342844	9.462488	0.268549
200	H	0	1.250763	9.881585	1.183436
201	H	0	0.497104	9.056314	-0.109300
202	C	0	3.326295	9.826559	-1.111927
203	O	0	2.681565	10.970184	-1.389163
204	H	0	1.822021	10.946772	-0.880904
205	C	0	2.609703	6.065298	-6.848081
206	O	0	3.866617	6.659260	-6.516477
207	H	0	3.827851	7.620932	-6.699644

208	N	0	2.907999	3.549804	-6.772151
209	H	0	2.498400	2.695431	-6.415839
210	H	0	3.918195	3.424081	-6.779754
211	N	0	6.541491	-1.145120	-5.667613
212	H	0	6.883665	-1.999228	-5.240171
213	H	0	6.079504	-1.337248	-6.551033
214	C	0	5.833304	1.129171	-5.218038
215	O	0	7.045433	1.558810	-5.642137
216	H	0	7.638507	0.776380	-5.757489
217	C	0	2.533106	-3.134625	5.058600
218	O	0	2.935560	-4.504463	5.121400
219	H	0	3.910190	-4.560745	5.028132
220	N	0	4.104573	-7.213752	-1.526167
221	H	0	4.666602	-7.652047	-2.244060
222	H	0	4.617857	-7.043706	-0.671026
223	N	0	7.425761	3.369174	6.381687
224	H	0	8.091676	2.655633	6.657882
225	H	0	7.335916	4.098813	7.077826
226	C	0	5.426947	1.958519	6.865062
227	O	0	6.221243	0.964781	7.359181
228	H	0	5.757632	0.375377	7.989577
229	C	0	-4.956849	4.859346	5.791857
230	O	0	-6.318521	4.723818	5.378158
231	H	0	-6.847138	4.971091	6.163797
232	N	0	-2.552088	5.392432	5.777911
233	H	0	-2.396678	6.312164	5.380987
234	H	0	-2.643837	5.413374	6.788097
235	N	0	5.055672	-4.318454	2.212861

236	H	0	4.167104	-4.444774	2.681349
237	H	0	5.683069	-5.104263	2.341352
238	C	0	6.070210	-3.286360	0.095288
239	O	0	6.676961	-2.284837	0.775087
240	H	0	7.360066	-1.822654	0.244232
241	N	0	-11.180614	1.474791	-1.021998
242	H	0	-11.432596	1.326258	-1.993189
243	H	0	-11.028822	2.462422	-0.840396
244	C	0	-13.579035	1.002051	-0.466402
245	O	0	-13.815427	1.785749	-1.542302
246	H	0	-14.768200	1.843074	-1.765105
247	N	0	-6.059324	1.321545	-5.915549
248	H	0	-6.285033	2.254059	-6.232720
249	H	0	-6.188052	0.627980	-6.642351
250	C	0	-5.561330	-2.910415	-3.715861
251	O	0	-6.434717	-2.880858	-4.738761
252	H	0	-6.385251	-3.667997	-5.321316
253	N	0	-7.889823	0.174330	-0.610515
254	H	0	-7.862124	0.908217	0.090067
255	H	0	-8.791550	-0.018498	-1.025808
256	N	0	-2.210202	-3.703058	-4.966006
257	H	0	-1.998301	-2.708751	-5.018404
258	H	0	-3.197704	-3.837901	-4.761594
259	C	0	-1.720776	-5.874729	-3.940078
260	O	0	-3.030480	-6.172720	-3.785468
261	H	0	-3.195671	-7.138346	-3.745018
262	N	0	-1.136980	-3.201941	1.110179
263	C	0	-2.376807	-3.602258	1.566676

264	C	0	-2.752169	-5.032055	1.351298
265	H	0	-3.839975	-5.100633	1.339933
266	H	0	-2.347154	-5.433469	0.422789
267	H	0	-2.372012	-5.661148	2.157897
268	H	0	-0.505695	-3.889986	0.716109
269	O	0	-3.156975	-2.810453	2.137354
270	C	0	-1.788107	-0.860441	0.726494
271	H	0	-2.824233	-1.149787	0.637819
272	C	0	0.890251	-0.087890	0.907764
273	H	0	1.933350	0.213617	0.909817

DOPA decarboxylase: Overall Charge = 0, Total energy = -7657.88962625 Ha

1	N	0	8.051862	5.305088	5.626244
2	C	0	7.248658	4.093679	5.615331
3	C	0	7.826013	2.993472	6.489268
4	O	0	7.117005	2.085009	6.916936
5	C	0	7.212128	3.503718	4.176700
6	C	0	6.292853	4.212276	3.233305
7	C	0	6.538376	5.351348	2.502569
8	C	0	4.973638	3.754430	2.852338
9	N	0	5.446206	5.636384	1.698365
10	C	0	4.479947	4.661460	1.877840
11	C	0	4.190533	2.638320	3.204192
12	C	0	3.258877	4.460844	1.219504
13	C	0	2.978837	2.431104	2.555650
14	C	0	2.524214	3.332253	1.564606
15	H	0	8.154163	5.734423	6.539211
16	H	0	7.742321	5.980765	4.934730
17	H	0	6.212713	4.221403	5.962365
18	H	0	6.899776	2.455392	4.242824
19	H	0	8.243700	3.517241	3.808738
20	H	0	7.423423	5.967053	2.464464
21	H	0	5.397346	6.391768	1.033411
22	H	0	4.532192	1.947758	3.969840
23	H	0	2.930331	5.136442	0.436363
24	H	0	2.374973	1.561937	2.798844
25	H	0	1.583744	3.132219	1.060074
26	N	0	-5.333105	0.693931	5.504109

27	C	0	-4.005894	0.660072	4.934374
28	C	0	-3.154149	-0.558846	5.336998
29	O	0	-3.504581	-1.367418	6.221114
30	C	0	-3.257858	1.968272	5.263057
31	C	0	-3.576225	3.138686	4.351743
32	C	0	-2.815346	4.310407	4.468071
33	C	0	-4.560067	3.087589	3.356622
34	C	0	-2.999581	5.386792	3.599778
35	C	0	-4.750047	4.151219	2.475732
36	C	0	-3.961027	5.292492	2.592624
37	O	0	-4.156225	6.294843	1.645862
38	H	0	-5.336730	0.867535	6.504870
39	H	0	-5.928697	-0.097824	5.280411
40	H	0	-4.101700	0.579471	3.841903
41	H	0	-3.485071	2.230380	6.305458
42	H	0	-2.175406	1.798581	5.229114
43	H	0	-2.051091	4.375961	5.236397
44	H	0	-5.192085	2.213117	3.266630
45	H	0	-2.390996	6.279700	3.700484
46	H	0	-5.503754	4.107477	1.697764
47	H	0	-3.747666	7.142456	1.900959
48	N	0	-2.000641	-0.688849	4.624016
49	C	0	-1.106530	-1.829812	4.716817
50	C	0	0.327661	-1.338217	4.596377
51	O	0	0.574412	-0.328913	3.864291
52	C	0	-1.360817	-2.803287	3.529134
53	C	0	-2.777497	-3.319604	3.525862
54	C	0	-3.738053	-2.771328	2.667255

55	C	0	-3.160330	-4.331721	4.417623
56	C	0	-5.060425	-3.225661	2.705413
57	C	0	-4.477470	-4.786324	4.456389
58	C	0	-5.432872	-4.231322	3.599547
59	H	0	-1.766560	-0.010025	3.907403
60	H	0	-1.291690	-2.329985	5.670866
61	H	0	-1.157988	-2.240981	2.609855
62	H	0	-0.640468	-3.629074	3.572176
63	H	0	-3.454246	-2.001538	1.954930
64	H	0	-2.419932	-4.764427	5.084571
65	H	0	-5.785257	-2.797754	2.021066
66	H	0	-4.758838	-5.571954	5.148118
67	H	0	-6.454872	-4.594628	3.622038
68	N	0	1.314301	-2.013583	5.192497
69	C	0	2.701469	-1.619303	4.947146
70	C	0	3.074976	-1.963046	3.510609
71	O	0	2.421067	-2.779097	2.827931
72	C	0	3.510085	-2.511971	5.921620
73	C	0	2.665532	-3.796720	5.969909
74	C	0	1.216751	-3.284406	5.949396
75	H	0	2.836519	-0.549950	5.128496
76	H	0	3.549501	-2.029736	6.901673
77	H	0	4.531091	-2.679051	5.572001
78	H	0	2.861667	-4.405561	5.082861
79	H	0	2.867235	-4.399370	6.855880
80	H	0	0.844529	-3.085653	6.958879
81	H	0	0.539113	-3.981029	5.452224
82	N	0	4.231609	-1.389433	3.085350

83	C	0	4.986012	-1.966172	1.991354
84	C	0	6.219285	-2.603075	2.558296
85	O	0	6.776630	-2.405393	3.636966
86	C	0	5.325611	-0.958252	0.859240
87	O	0	5.940291	-1.640990	-0.251653
88	C	0	6.274621	0.137491	1.314141
89	H	0	4.723110	-0.759306	3.707746
90	H	0	4.385645	-2.762882	1.546589
91	H	0	4.362469	-0.523491	0.557348
92	H	0	5.656607	-2.584008	-0.324737
93	H	0	5.816816	0.756584	2.091517
94	H	0	6.518503	0.784633	0.468217
95	H	0	7.207981	-0.291354	1.693116
96	N	0	-11.123720	-1.817172	-2.535965
97	C	0	-10.122424	-0.842795	-2.924648
98	C	0	-10.583085	-0.206103	-4.204069
99	O	0	-11.173391	-0.756016	-5.132793
100	C	0	-8.710765	-1.469514	-3.150155
101	C	0	-7.573579	-0.504831	-3.215655
102	N	0	-6.672937	-0.441722	-4.278481
103	C	0	-7.110250	0.391694	-2.289325
104	C	0	-5.688017	0.436800	-4.008867
105	N	0	-5.953297	0.954183	-2.804006
106	H	0	-10.998504	-2.203762	-1.609119
107	H	0	-11.320022	-2.520016	-3.239658
108	H	0	-10.051219	-0.067739	-2.158645
109	H	0	-8.737680	-2.094747	-4.049179
110	H	0	-8.547405	-2.144103	-2.304409

111	H	0	-6.734708	-0.993679	-5.123069
112	H	0	-7.487151	0.637612	-1.312886
113	H	0	-4.769975	0.616552	-4.575318
114	N	0	-9.149723	-2.740868	2.924811
115	C	0	-8.341838	-1.634408	3.403958
116	C	0	-8.887579	-0.952912	4.640973
117	O	0	-8.139669	-0.626806	5.563520
118	C	0	-8.141452	-0.631632	2.228534
119	O	0	-7.992906	-1.471550	1.054657
120	C	0	-6.912706	0.235755	2.408167
121	H	0	-8.929748	-3.641816	3.331835
122	H	0	-10.151153	-2.564568	2.928307
123	H	0	-7.359725	-2.015601	3.696239
124	H	0	-9.045644	-0.024131	2.107748
125	H	0	-8.520543	-2.296100	1.276448
126	H	0	-6.978457	0.802684	3.343363
127	H	0	-6.809017	0.939354	1.578674
128	H	0	-6.015573	-0.393539	2.444471
129	N	0	4.708530	-7.446658	-2.033089
130	C	0	5.305098	-6.149971	-1.749888
131	C	0	4.600592	-5.356832	-0.667556
132	O	0	4.936486	-4.205972	-0.388722
133	C	0	5.339964	-5.217930	-2.990493
134	C	0	3.956391	-4.896121	-3.457942
135	N	0	3.278880	-5.583536	-4.458941
136	C	0	3.033376	-4.011482	-2.950663
137	C	0	2.001689	-5.106622	-4.526280
138	N	0	1.819343	-4.153443	-3.614997

139	H	0	5.353345	-8.218002	-2.121940
140	H	0	3.973292	-7.454126	-2.729709
141	H	0	6.333746	-6.300820	-1.406050
142	H	0	5.935427	-5.685079	-3.779862
143	H	0	5.833703	-4.295852	-2.680079
144	H	0	3.673844	-6.285323	-5.068287
145	H	0	3.171872	-3.296838	-2.158315
146	H	0	1.267988	-5.468493	-5.223756
147	N	0	3.617222	-5.980459	-0.041240
148	C	0	2.988110	-5.342554	1.092487
149	C	0	3.515044	-6.034538	2.338365
150	O	0	4.347032	-5.495302	3.060503
151	C	0	1.469115	-5.231363	0.994176
152	C	0	1.131510	-4.340951	-0.212833
153	C	0	-0.077405	-3.429498	0.000362
154	C	0	-0.327315	-2.512383	-1.191389
155	N	0	-0.612232	-3.299947	-2.442564
156	H	0	3.411257	-6.934919	-0.323136
157	H	0	3.385869	-4.335489	1.170867
158	H	0	1.119875	-4.758106	1.918594
159	H	0	1.002440	-6.218772	0.918621
160	H	0	1.993258	-3.689482	-0.409818
161	H	0	1.008663	-4.961281	-1.108795
162	H	0	0.120350	-2.789835	0.867605
163	H	0	-0.980405	-4.015600	0.222939
164	H	0	-1.170969	-1.845073	-1.010109
165	H	0	0.544761	-1.887823	-1.398106
166	H	0	0.282355	-3.591229	-2.932841

167	H	0	-1.207084	-2.719307	-3.104904
168	H	0	-1.149032	-4.143463	-2.221854
169	N	0	8.616242	-3.401002	-3.095206
170	C	0	7.695510	-3.006556	-4.122343
171	C	0	8.237606	-3.082551	-5.494534
172	O	0	9.360203	-2.663151	-5.799144
173	C	0	7.261965	-1.533836	-3.866613
174	C	0	6.025321	-1.156404	-4.642254
175	C	0	6.093862	-0.592753	-5.920975
176	C	0	4.767645	-1.382411	-4.066487
177	C	0	4.923363	-0.260760	-6.610350
178	C	0	3.597190	-1.054137	-4.749148
179	C	0	3.674866	-0.490381	-6.026873
180	H	0	8.392961	-3.045673	-2.175116
181	H	0	8.900437	-4.372125	-3.091303
182	H	0	6.769055	-3.592127	-4.141974
183	H	0	8.108861	-0.878674	-4.095617
184	H	0	7.049611	-1.453730	-2.793262
185	H	0	7.063591	-0.403354	-6.373187
186	H	0	4.721949	-1.785305	-3.058081
187	H	0	4.987210	0.180479	-7.598538
188	H	0	2.631045	-1.225239	-4.286060
189	H	0	2.766613	-0.228240	-6.557996
190	N	0	5.856604	4.312356	-1.205463
191	C	0	4.933695	4.039032	-2.310607
192	C	0	4.247827	5.396146	-2.528210
193	O	0	3.364808	5.776124	-1.757567
194	C	0	3.851759	2.970775	-2.059013

195	C	0	4.450083	1.698256	-1.437627
196	C	0	3.084760	2.685172	-3.357028
197	C	0	5.693512	1.170419	-2.159722
198	H	0	5.441736	4.219914	-0.285195
199	H	0	6.759528	3.861922	-1.274107
200	H	0	5.516630	3.789346	-3.201463
201	H	0	3.147969	3.392175	-1.329236
202	H	0	4.691196	1.899224	-0.384510
203	H	0	3.668818	0.925550	-1.432513
204	H	0	3.734144	2.224536	-4.110303
205	H	0	2.673130	3.606837	-3.788369
206	H	0	2.246724	2.006234	-3.171453
207	H	0	6.558980	1.819097	-1.988594
208	H	0	5.530204	1.103586	-3.239643
209	H	0	5.944650	0.170113	-1.795108
210	N	0	1.774423	6.845611	-3.900659
211	C	0	0.435357	6.855106	-4.473678
212	C	0	-0.300924	8.161136	-4.191038
213	O	0	-1.370527	8.403433	-4.725849
214	C	0	-0.494867	5.718103	-3.926773
215	C	0	-0.758420	5.909296	-2.450112
216	C	0	-1.836298	6.705443	-2.027042
217	C	0	0.118472	5.397424	-1.482545
218	C	0	-2.011501	7.015808	-0.678402
219	C	0	-0.063404	5.695763	-0.128675
220	C	0	-1.119735	6.514733	0.276509
221	H	0	1.790531	6.888652	-2.884517
222	H	0	2.301067	6.042439	-4.232312

223	H	0	0.504745	6.769177	-5.560549
224	H	0	-1.432500	5.733000	-4.489857
225	H	0	0.003167	4.762599	-4.119339
226	H	0	-2.529325	7.098009	-2.764801
227	H	0	0.953148	4.771613	-1.787088
228	H	0	-2.844390	7.641196	-0.377480
229	H	0	0.617374	5.290703	0.611869
230	H	0	-1.243792	6.756238	1.326746
231	N	0	-6.231816	-4.215601	-2.679596
232	C	0	-6.371521	-3.532757	-1.530535
233	C	0	-7.391830	-4.035478	-0.548580
234	C	0	-5.563722	-2.407698	-1.252172
235	O	0	-5.738878	-1.659074	-0.127787
236	C	0	-4.560684	-2.036184	-2.165999
237	C	0	-4.453429	-2.747152	-3.376027
238	C	0	-5.310038	-3.827009	-3.580675
239	C	0	-3.502178	-2.363761	-4.483327
240	O	0	-2.246690	-1.890683	-3.972213
241	15	0	-1.564045	-0.366370	-4.649884
242	O	0	-0.994115	-0.762721	-6.103925
243	O	0	-2.886770	0.580723	-4.687469
244	O	0	-0.465330	-0.070547	-3.503166
245	H	0	-8.307227	-3.431451	-0.554732
246	H	0	-6.997989	-4.042064	0.473270
247	H	0	-7.668357	-5.053088	-0.824305
248	H	0	-6.664555	-1.645649	0.286984
249	H	0	-5.246739	-4.414171	-4.489862
250	H	0	-3.339274	-3.226439	-5.136654

251	H	0	-3.948457	-1.561351	-5.086230
252	C	0	-1.879190	2.818875	0.047739
253	C	0	-1.283698	1.997009	-0.912710
254	C	0	-1.524524	2.684660	1.391439
255	C	0	-0.608069	1.707692	1.782164
256	C	0	-0.046915	0.857746	0.819189
257	O	0	0.838694	-0.150895	1.162426
258	H	0	-5.326894	1.644889	-2.323020
259	H	0	-1.553254	2.083259	-1.957494
260	H	0	-1.974103	3.337476	2.132488
261	H	0	-0.323722	1.603629	2.824018
262	O	0	9.161912	3.053908	6.703410
263	H	0	9.481298	2.296869	7.238549
264	O	0	6.776056	-3.544662	1.637267
265	H	0	7.597267	-3.925578	2.015185
266	O	0	7.454805	-3.661464	-6.541731
267	H	0	7.952534	-3.572932	-7.381058
268	O	0	3.041087	-7.337776	2.686842
269	H	0	3.503806	-7.639098	3.496838
270	O	0	-10.286957	-0.680123	4.748569
271	H	0	-10.465434	-0.242516	5.607452
272	O	0	-10.209876	1.169002	-4.324589
273	H	0	-10.479893	1.510978	-5.202825
274	O	0	0.277238	9.106330	-3.287265
275	H	0	-0.337029	9.864316	-3.188731
276	O	0	4.671485	6.126527	-3.584725
277	H	0	4.184033	6.979091	-3.673691
278	C	0	-3.659383	-0.888264	-1.872671

279	H	0	-3.430258	-0.218153	-2.706445
280	O	0	-3.185641	-0.662686	-0.744405
281	C	0	-3.920965	3.593343	-1.094601
282	C	0	-4.827658	4.771657	-1.320837
283	H	0	-4.701638	5.537727	-0.552922
284	H	0	-4.609998	5.209279	-2.300220
285	H	0	-5.862781	4.425389	-1.329403
286	C	0	-0.369993	1.019556	-0.530388
287	H	0	0.038256	0.367499	-1.294827
288	O	0	-4.147472	2.470465	-1.627866
289	H	0	0.871681	-0.289579	2.141029
290	N	0	-2.847486	3.805543	-0.309143
291	H	0	-2.750693	4.725713	0.109642

Monoamine Oxidase: Overall Charge = -1, Total energy = -12613.6441783 Ha

1	N	0	5.094221	0.890483	7.571368
2	C	0	4.034551	0.073258	8.103921
3	C	0	4.363812	-1.416193	8.048898
4	O	0	4.835182	-1.985168	7.056218
5	C	0	2.741996	0.280400	7.271263
6	C	0	2.303762	1.727974	7.193652
7	C	0	1.926703	2.418940	8.348571
8	C	0	2.350452	2.424288	5.994376
9	C	0	1.584380	3.757703	8.302475
10	C	0	2.002495	3.766063	5.940404
11	C	0	1.623782	4.420628	7.096979

12	O	0	1.299702	5.747928	7.054809
13	H	0	5.054268	1.090223	6.578347
14	H	0	6.013866	0.792062	7.974668
15	H	0	3.835987	0.320062	9.151990
16	H	0	1.966887	-0.347033	7.722048
17	H	0	2.926491	-0.098827	6.258920
18	H	0	1.894069	1.892679	9.298599
19	H	0	2.684548	1.918190	5.096048
20	H	0	1.291431	4.280499	9.207122
21	H	0	2.032608	4.317617	5.008014
22	H	0	1.045727	6.104435	7.924206
23	C	0	-12.286783	-1.847492	5.376894
24	C	0	-12.850999	-1.423723	4.016038
25	O	0	-12.109713	-0.884642	3.194483
26	C	0	-11.609503	-3.217789	5.258157
27	C	0	-12.651171	-4.189848	5.694181
28	C	0	-13.372260	-3.488677	6.809624
29	H	0	-11.583147	-1.064904	5.672426
30	H	0	-11.251718	-3.405864	4.249144
31	H	0	-10.741516	-3.247333	5.926691
32	H	0	-12.235228	-5.150837	6.006140
33	H	0	-13.347547	-4.390172	4.868430
34	H	0	-12.840253	-3.620452	7.762437
35	H	0	-14.397172	-3.842703	6.945309
36	N	0	-14.146816	-1.656693	3.797240
37	C	0	-14.776494	-1.469040	2.482722
38	C	0	-15.594761	-0.193052	2.389760
39	O	0	-16.519628	0.002991	3.183146

40	C	0	-15.695662	-2.654641	2.143734
41	C	0	-15.044284	-4.008593	2.288899
42	C	0	-13.872585	-4.319149	1.596429
43	C	0	-15.617074	-4.977576	3.114405
44	C	0	-13.271600	-5.578112	1.734332
45	C	0	-15.032988	-6.240857	3.251009
46	C	0	-13.855659	-6.541305	2.560268
47	H	0	-14.684261	-1.991450	4.591722
48	H	0	-13.952388	-1.449175	1.769938
49	H	0	-16.054491	-2.509272	1.115841
50	H	0	-16.576587	-2.598649	2.793765
51	H	0	-13.416681	-3.579135	0.944591
52	H	0	-16.528222	-4.743382	3.656362
53	H	0	-12.359080	-5.802320	1.193600
54	H	0	-15.492176	-6.982226	3.894849
55	H	0	-13.396610	-7.517553	2.666461
56	N	0	-15.265260	0.683755	1.417562
57	C	0	-16.088946	1.873133	1.184255
58	C	0	-17.477003	1.437833	0.723459
59	O	0	-17.578551	0.618809	-0.180678
60	C	0	-15.353532	2.598844	0.044438
61	C	0	-13.971743	2.028265	0.043478
62	C	0	-14.139911	0.605586	0.472464
63	H	0	-16.167003	2.469674	2.096228
64	H	0	-15.375800	3.680371	0.182158
65	H	0	-15.853625	2.373033	-0.903106
66	H	0	-13.484270	2.116724	-0.929643
67	H	0	-13.343758	2.554703	0.770495

68	H	0	-13.251329	0.215249	0.971711
69	H	0	-14.397125	-0.049994	-0.369009
70	N	0	-13.487013	-7.238684	-6.498481
71	C	0	-12.553679	-7.700278	-5.451131
72	C	0	-12.540650	-9.210935	-5.335471
73	O	0	-11.474091	-9.820879	-5.176102
74	C	0	-12.855327	-7.060106	-4.083010
75	C	0	-13.022501	-5.592901	-4.209845
76	C	0	-14.152798	-4.869669	-3.964689
77	C	0	-12.047482	-4.662984	-4.701299
78	N	0	-13.935301	-3.540700	-4.247184
79	C	0	-12.653896	-3.387402	-4.707519
80	C	0	-10.712006	-4.782768	-5.117036
81	C	0	-11.974493	-2.232037	-5.122676
82	C	0	-10.037332	-3.640391	-5.532716
83	C	0	-10.673126	-2.379382	-5.535275
84	H	0	-14.459443	-7.416074	-6.260699
85	H	0	-13.340477	-6.260263	-6.723804
86	H	0	-11.542873	-7.439160	-5.767243
87	H	0	-12.049395	-7.315754	-3.382280
88	H	0	-13.777189	-7.503088	-3.685427
89	H	0	-15.109062	-5.209132	-3.598922
90	H	0	-14.614340	-2.804873	-4.140815
91	H	0	-10.203178	-5.741082	-5.102948
92	H	0	-12.466926	-1.265719	-5.122797
93	H	0	-9.009360	-3.717581	-5.857514
94	H	0	-10.123992	-1.509939	-5.881835
95	N	0	-12.409986	3.198844	-8.432197

96	C	0	-11.835281	2.601872	-7.215953
97	C	0	-10.796005	1.505642	-7.507252
98	O	0	-9.766531	1.425235	-6.817317
99	C	0	-12.915500	2.106465	-6.241700
100	C	0	-12.424165	1.741080	-4.824664
101	C	0	-11.687164	2.904602	-4.112854
102	C	0	-13.575144	1.237145	-3.949573
103	H	0	-11.734386	3.655853	-9.032709
104	H	0	-13.038501	2.593015	-8.949425
105	H	0	-11.269437	3.394284	-6.713112
106	H	0	-10.991667	0.807331	-8.333962
107	H	0	-13.430558	1.237579	-6.676862
108	H	0	-13.657343	2.913153	-6.171404
109	H	0	-11.697342	0.919530	-4.925471
110	H	0	-11.454653	2.619634	-3.082007
111	H	0	-12.320658	3.799553	-4.087042
112	H	0	-10.747479	3.154611	-4.612646
113	H	0	-14.143003	0.442495	-4.446568
114	H	0	-14.271388	2.053761	-3.721858
115	H	0	-13.191767	0.840157	-3.004457
116	N	0	-6.891252	1.537025	-6.389297
117	C	0	-6.339900	0.318513	-5.768586
118	C	0	-5.025038	-0.110894	-6.418572
119	O	0	-4.029222	-0.296947	-5.721336
120	C	0	-7.389389	-0.808604	-5.813378
121	C	0	-6.878212	-2.169146	-5.364253
122	C	0	-6.642059	-2.437333	-4.016736
123	C	0	-6.697015	-3.200329	-6.296862

124	C	0	-6.183756	-3.711526	-3.599650
125	C	0	-6.243208	-4.485233	-5.882283
126	C	0	-5.984053	-4.726867	-4.539134
127	H	0	-7.890255	1.511069	-6.554567
128	H	0	-6.561282	2.402313	-5.983833
129	H	0	-6.051687	0.480538	-4.722457
130	H	0	-7.768529	-0.875639	-6.839933
131	H	0	-8.220914	-0.484648	-5.177910
132	H	0	-6.800705	-1.659308	-3.274665
133	H	0	-6.898739	-3.015759	-7.347698
134	H	0	-5.983874	-3.898118	-2.552475
135	H	0	-6.105933	-5.265188	-6.622225
136	H	0	-5.633650	-5.701209	-4.216773
137	N	0	-2.271387	2.591775	-5.820957
138	C	0	-1.837491	2.675447	-4.423004
139	C	0	-1.010831	1.452836	-4.001196
140	O	0	-0.090643	1.581706	-3.206664
141	C	0	-3.060219	2.824416	-3.517960
142	C	0	-2.908934	3.017617	-2.009144
143	C	0	-1.929754	4.144287	-1.667641
144	C	0	-4.293460	3.328023	-1.455779
145	H	0	-1.557179	2.809619	-6.504177
146	H	0	-3.152219	3.050617	-6.016873
147	H	0	-1.161512	3.524617	-4.246713
148	H	0	-3.708311	1.954761	-3.704123
149	H	0	-3.610632	3.701554	-3.896489
150	H	0	-2.542949	2.082138	-1.561389
151	H	0	-1.980423	4.389415	-0.601726

152	H	0	-2.189785	5.051626	-2.230443
153	H	0	-0.898082	3.867396	-1.908868
154	H	0	-5.027848	2.583072	-1.782178
155	H	0	-4.630674	4.310656	-1.804884
156	H	0	-4.290260	3.340746	-0.360523
157	N	0	-1.342072	0.279829	-4.532214
158	C	0	-0.601798	-0.952462	-4.199051
159	C	0	0.799943	-0.962526	-4.775092
160	O	0	1.747904	-1.433690	-4.123335
161	C	0	-1.335389	-2.201048	-4.713342
162	S	0	-2.795539	-2.705893	-3.752169
163	H	0	-2.120796	0.238989	-5.189467
164	H	0	-0.452278	-1.004854	-3.117381
165	H	0	-1.695778	-2.018393	-5.728236
166	H	0	-0.635307	-3.037769	-4.751322
167	H	0	-2.124007	-3.126206	-2.630954
168	N	0	-4.259027	-6.817913	2.172164
169	C	0	-3.395038	-5.767927	1.614065
170	C	0	-3.934236	-4.344821	1.860621
171	O	0	-3.150620	-3.418161	2.074126
172	C	0	-3.031471	-6.029838	0.108089
173	C	0	-1.673403	-5.408298	-0.243704
174	C	0	-4.158034	-5.595854	-0.862112
175	C	0	-1.199658	-5.689601	-1.702919
176	H	0	-4.162113	-7.720044	1.724248
177	H	0	-4.248931	-6.880241	3.182449
178	H	0	-2.428672	-5.745708	2.142594
179	H	0	-2.922092	-7.122797	0.029383

180	H	0	-1.726829	-4.321042	-0.086323
181	H	0	-0.912458	-5.782041	0.450070
182	H	0	-3.967791	-5.970483	-1.871357
183	H	0	-4.220622	-4.500540	-0.907424
184	H	0	-5.126518	-5.984767	-0.530529
185	H	0	-1.108547	-6.767765	-1.874091
186	H	0	-0.221529	-5.228874	-1.864473
187	H	0	-1.898349	-5.291184	-2.442389
188	N	0	-5.255719	-4.179749	1.855490
189	C	0	-5.850761	-2.832289	1.965922
190	C	0	-6.134058	-2.418043	3.407603
191	O	0	-6.430150	-1.255007	3.671791
192	C	0	-7.156655	-2.675353	1.114038
193	C	0	-8.308951	-3.539450	1.655684
194	C	0	-6.892790	-3.052266	-0.342431
195	C	0	-9.153827	-2.889337	2.716768
196	H	0	-5.831712	-5.012495	1.795830
197	H	0	-5.100529	-2.121027	1.604737
198	H	0	-7.431826	-1.616773	1.182538
199	H	0	-7.921199	-4.503239	2.014772
200	H	0	-8.955803	-3.787272	0.805353
201	H	0	-6.008882	-2.539371	-0.739839
202	H	0	-7.754568	-2.784611	-0.960765
203	H	0	-6.731848	-4.131420	-0.433626
204	H	0	-10.068292	-3.469998	2.844418
205	H	0	-9.453384	-1.875843	2.433648
206	H	0	-8.663687	-2.837164	3.693629
207	N	0	-1.188864	-2.203918	7.005571

208	C	0	-0.948207	-0.866103	7.555301
209	C	0	-1.288351	-0.741555	9.044718
210	O	0	-1.452498	0.371788	9.539986
211	C	0	-1.726512	0.192281	6.750999
212	C	0	-1.173066	0.442586	5.344218
213	C	0	-1.294082	-0.781123	4.420037
214	O	0	-0.284594	-1.393416	4.037497
215	N	0	-2.527513	-1.163643	4.087407
216	H	0	-0.685714	-2.370019	6.139764
217	H	0	-1.030421	-2.949746	7.676153
218	H	0	0.115976	-0.579452	7.514205
219	H	0	-1.691912	1.134901	7.303974
220	H	0	-2.773260	-0.127838	6.703405
221	H	0	-1.710198	1.284479	4.891869
222	H	0	-0.110559	0.700947	5.393276
223	H	0	-3.337852	-0.621175	4.344765
224	H	0	-2.676769	-1.960219	3.470383
225	N	0	-9.637598	8.271544	1.243008
226	C	0	-9.514799	7.591674	-0.045362
227	C	0	-10.168918	8.538958	-1.049917
228	O	0	-11.374834	8.776359	-0.989181
229	C	0	-10.231161	6.216102	-0.021340
230	C	0	-9.599925	5.291700	1.031329
231	C	0	-10.212895	5.546137	-1.403801
232	C	0	-10.591089	4.333759	1.669528
233	H	0	-10.589501	8.531835	1.475341
234	H	0	-8.969583	9.013053	1.411321
235	H	0	-8.456625	7.462181	-0.289093

236	H	0	-11.273483	6.422841	0.264012
237	H	0	-9.144128	5.911799	1.812091
238	H	0	-8.788928	4.722232	0.554539
239	H	0	-9.180574	5.420134	-1.749578
240	H	0	-10.759921	6.130558	-2.151457
241	H	0	-10.675546	4.556157	-1.350606
242	H	0	-11.085694	3.710781	0.915770
243	H	0	-11.368255	4.884530	2.209593
244	H	0	-10.096238	3.663937	2.378925
245	N	0	-4.569830	8.959946	-1.315068
246	C	0	-4.697415	8.216227	-0.064786
247	C	0	-3.889875	8.824162	1.066955
248	O	0	-2.731937	9.188134	0.877439
249	C	0	-4.236609	6.775053	-0.288221
250	C	0	-4.521855	5.802603	0.845200
251	C	0	-5.823842	5.383118	1.122855
252	C	0	-3.480633	5.251651	1.590731
253	C	0	-6.090026	4.452804	2.132623
254	C	0	-3.737076	4.307707	2.607970
255	C	0	-5.039265	3.920522	2.868104
256	O	0	-5.299395	2.998298	3.866967
257	H	0	-3.605620	9.051446	-1.623516
258	H	0	-5.045333	9.855802	-1.313332
259	H	0	-5.751650	8.213253	0.228343
260	H	0	-3.160666	6.786445	-0.505632
261	H	0	-4.743532	6.444414	-1.200500
262	H	0	-6.652251	5.777850	0.541494
263	H	0	-2.455214	5.553979	1.399873

264	H	0	-7.103761	4.131804	2.343280
265	H	0	-2.913715	3.893001	3.180863
266	H	0	-4.485204	2.652255	4.274489
267	N	0	0.226072	10.096730	-0.115505
268	C	0	1.063258	9.623134	-1.232451
269	O	0	-0.560033	10.428515	-2.790720
270	C	0	0.864787	8.116906	-1.497651
271	C	0	1.473154	7.193232	-0.460156
272	C	0	2.844780	7.205337	-0.203273
273	C	0	0.675269	6.280261	0.222090
274	C	0	3.403336	6.349439	0.758633
275	C	0	1.223869	5.409039	1.169361
276	C	0	2.585689	5.443801	1.434897
277	H	0	-0.766600	9.912198	-0.201136
278	H	0	0.622599	9.952427	0.801154
279	H	0	2.110359	9.852260	-1.024701
280	H	0	-0.214351	7.938862	-1.564448
281	H	0	1.287685	7.855113	-2.478571
282	H	0	3.491841	7.892548	-0.740522
283	H	0	-0.391931	6.258727	0.023593
284	H	0	4.465777	6.393581	0.965047
285	H	0	0.580899	4.709467	1.691216
286	H	0	3.014617	4.769897	2.168414
287	N	0	7.263703	1.917330	-4.315501
288	C	0	7.800742	1.081164	-3.253644
289	C	0	8.730114	-0.006184	-3.752871
290	O	0	9.675819	-0.386512	-3.053676
291	C	0	6.688157	0.369466	-2.472162

292	C	0	5.398864	1.143200	-2.316264
293	C	0	5.391205	2.435027	-1.795758
294	C	0	4.173613	0.563422	-2.670750
295	C	0	4.191085	3.142569	-1.645542
296	C	0	2.981357	1.259678	-2.527257
297	C	0	2.996477	2.547614	-2.012357
298	O	0	1.802972	3.234005	-1.878096
299	H	0	7.957203	2.368153	-4.904493
300	H	0	6.524547	1.483214	-4.856841
301	H	0	8.407060	1.696942	-2.582795
302	H	0	7.095265	0.091341	-1.490625
303	H	0	6.450416	-0.573736	-2.980779
304	H	0	6.329438	2.912892	-1.531291
305	H	0	4.146859	-0.437630	-3.092805
306	H	0	4.203311	4.150988	-1.240375
307	H	0	2.026878	0.823459	-2.797170
308	H	0	1.921350	4.084994	-1.413008
309	N	0	7.777338	-5.383046	0.386833
310	C	0	7.642663	-4.855133	1.732827
311	C	0	8.912699	-5.158482	2.531440
312	O	0	9.755471	-5.956129	2.104995
313	C	0	6.444203	-5.460053	2.482441
314	C	0	5.093499	-5.093210	1.906282
315	C	0	4.672946	-5.588901	0.657022
316	C	0	4.232015	-4.259103	2.606294
317	C	0	3.419163	-5.232610	0.127861
318	C	0	2.983587	-3.913058	2.085878
319	C	0	2.586712	-4.402327	0.855575

320	O	0	1.347101	-4.040740	0.365068
321	H	0	8.115079	-6.346736	0.386201
322	H	0	8.351965	-4.798806	-0.214102
323	H	0	7.525265	-3.764515	1.707512
324	H	0	6.498300	-5.115408	3.519961
325	H	0	6.568194	-6.552041	2.480520
326	H	0	5.333478	-6.233374	0.092293
327	H	0	4.541623	-3.863582	3.569600
328	H	0	3.093354	-5.607545	-0.835271
329	H	0	2.324571	-3.254288	2.645043
330	H	0	0.899611	-3.387628	0.933129
331	O	0	20.778192	0.916292	0.618971
332	C	0	21.100041	1.003136	-1.790784
333	O	0	21.650772	2.270813	-1.476215
334	C	0	22.144882	-0.084861	-2.003759
335	N	0	22.779283	-0.133479	-3.340529
336	C	0	22.198062	0.091927	-4.571617
337	N	0	23.137059	-0.076731	-5.530468
338	C	0	24.317238	-0.393210	-4.934736
339	C	0	25.597042	-0.662007	-5.432374
340	N	0	25.850101	-0.674290	-6.751239
341	N	0	26.610894	-0.979239	-4.538240
342	C	0	26.377259	-1.009607	-3.180107
343	N	0	25.113413	-0.738372	-2.699793
344	C	0	24.100086	-0.436085	-3.558891
345	N	0	7.388872	-1.400059	2.903501
346	C	0	6.701389	-1.795051	4.030325
347	O	0	6.925338	-2.892026	4.540712

348	N	0	5.808891	-0.922803	4.622433
349	C	0	5.561412	0.320602	4.064453
350	O	0	4.768036	1.084375	4.622271
351	C	0	6.241872	0.720339	2.919686
352	N	0	6.022912	1.970041	2.346671
353	C	0	6.842702	2.440337	1.342874
354	C	0	6.797597	3.793203	0.989475
355	C	0	7.843644	4.377843	0.271582
356	C	0	7.722745	5.835369	-0.074149
357	C	0	8.957216	3.610064	-0.107831
358	C	0	10.123250	4.181889	-0.882380
359	C	0	8.995900	2.251304	0.248138
360	C	0	7.939132	1.658179	0.967781
361	N	0	7.912606	0.291612	1.269581
362	C	0	7.165917	-0.147228	2.345625
363	C	0	8.887176	-0.644365	0.595495
364	C	0	10.057610	-1.040117	1.514374
365	O	0	10.705287	0.133331	1.964354
366	C	0	11.012630	-1.953521	0.740226
367	O	0	10.321778	-3.133884	0.364832
368	C	0	12.286438	-2.373339	1.492075
369	O	0	12.869227	-1.263458	2.156190
370	C	0	13.292565	-2.924214	0.486795
371	O	0	14.408203	-3.458533	1.183270
372	15	0	15.629472	-4.171147	0.406827
373	O	0	16.747554	-4.374584	1.529520
374	O	0	15.185940	-5.278697	-0.435458
375	O	0	16.095738	-3.065017	-0.622591

376	15	0	16.607562	-1.513734	-0.527842
377	O	0	16.442082	-0.914230	-1.999755
378	O	0	16.011923	-0.800750	0.665484
379	O	0	18.234830	-1.643955	-0.221623
380	C	0	19.170788	-1.752669	-1.256525
381	C	0	20.444773	-1.069781	-0.789931
382	O	0	21.441387	-1.289026	-1.762252
383	C	0	20.281438	0.442966	-0.645063
384	H	0	20.153196	0.611725	1.317114
385	H	0	20.472057	1.110909	-2.677359
386	H	0	21.741596	2.339962	-0.501151
387	H	0	22.969751	0.041126	-1.292300
388	H	0	21.163797	0.338837	-4.729407
389	H	0	26.777690	-0.887085	-7.081704
390	H	0	25.123729	-0.459335	-7.415601
391	H	0	27.174239	-1.250261	-2.496248
392	H	0	5.352578	-1.193733	5.502580
393	H	0	5.957291	4.382493	1.335433
394	H	0	6.805634	6.259381	0.335116
395	H	0	7.710508	5.983161	-1.159923
396	H	0	8.567565	6.412349	0.317091
397	H	0	10.568291	5.031315	-0.353833
398	H	0	9.789829	4.549907	-1.859163
399	H	0	10.892304	3.426101	-1.040494
400	H	0	9.884246	1.676472	0.025351
401	H	0	9.229533	-0.175576	-0.326721
402	H	0	8.326568	-1.549858	0.349494
403	H	0	9.646676	-1.610270	2.355243

404	H	0	11.620813	-0.083352	2.261461
405	H	0	11.307209	-1.442038	-0.185911
406	H	0	10.482030	-3.887746	0.966765
407	H	0	12.031953	-3.145761	2.232608
408	H	0	13.842824	-1.226754	2.037675
409	H	0	12.821761	-3.691842	-0.137016
410	H	0	13.619053	-2.095620	-0.157000
411	H	0	19.383112	-2.803196	-1.482083
412	H	0	18.803423	-1.270579	-2.169638
413	H	0	20.753420	-1.492325	0.173330
414	H	0	19.227975	0.725290	-0.742237
415	O	0	-13.716287	-9.898917	-5.401880
416	H	0	-13.572326	-10.888187	-5.342439
417	O	0	-9.401420	9.143487	-2.006000
418	H	0	-9.940968	9.746999	-2.592975
419	O	0	-1.379344	-1.861428	9.817359
420	H	0	-1.590301	-1.640968	10.772493
421	O	0	-4.959270	-0.301317	-7.766663
422	H	0	-4.042939	-0.571178	-8.063941
423	O	0	-6.059442	-3.342063	4.409456
424	H	0	-6.241076	-2.927783	5.304709
425	O	0	-18.594632	1.945846	1.315454
426	H	0	-19.429970	1.540045	0.933408
427	N	0	-13.359805	-2.064591	6.373238
428	H	0	-13.437931	-1.361572	7.108418
429	O	0	-4.441199	8.944824	2.308962
430	H	0	-3.803387	9.350756	2.967756
431	O	0	1.015949	-0.460876	-6.020297

432	H	0	1.982028	-0.513170	-6.280390
433	O	0	1.553912	10.931987	-3.291824
434	H	0	1.161870	11.353735	-4.109674
435	O	0	8.511998	-0.578525	-4.970492
436	H	0	9.195671	-1.280385	-5.172630
437	O	0	9.136830	-4.543794	3.722597
438	H	0	10.016204	-4.806842	4.112846
439	O	0	4.080259	-2.084976	9.190485
440	H	0	4.297512	-3.044375	9.157228
441	C	0	0.373205	-0.319992	1.109824
442	C	0	1.190340	0.393893	1.995942
443	C	0	2.341591	-0.985314	-0.141452
444	C	0	3.139431	-0.229598	0.718844
445	H	0	2.784959	-1.533978	-0.965263
446	O	0	4.515337	-0.141183	0.564783
447	H	0	4.797814	-0.387535	-0.337273
448	H	0	0.745767	0.885011	2.853192
449	C	0	0.688456	10.386385	-2.493055
450	H	0	-0.937197	10.909764	-3.561217
451	C	0	2.566890	0.455390	1.793506
452	H	0	3.210196	1.022128	2.458736
453	N	0	-1.045556	-0.374035	1.277360
454	H	0	-1.471580	-1.281921	1.437996
455	C	0	-1.910443	0.679591	1.242688
456	O	0	-3.144521	0.506497	1.384166
457	C	0	-1.329622	2.053329	1.023687
458	H	0	-0.822063	2.406828	1.926353
459	H	0	-0.605791	2.066894	0.203212

460	H	0	-2.153604	2.731002	0.803700
461	C	0	0.959251	-1.008371	0.044387
462	H	0	0.330290	-1.568481	-0.639678

Catechol-O-methyltransferase: Overall Charge = -1, Total energy = -8469.18776953 Ha

1	C	0	3.546446	9.672130	-1.552373
2	C	0	3.902466	8.669882	-2.642039
3	O	0	3.632032	8.888820	-3.824791
4	C	0	2.186388	9.294328	-0.913993
5	C	0	2.217218	7.977853	-0.200283
6	C	0	2.664652	7.752784	1.080999
7	C	0	1.872678	6.685651	-0.743596
8	N	0	2.622435	6.400567	1.369265
9	C	0	2.140427	5.719003	0.267764
10	C	0	1.386217	6.260089	-1.993022
11	C	0	1.922244	4.350131	0.061666
12	C	0	1.152803	4.904025	-2.195754
13	C	0	1.416451	3.962726	-1.172992
14	H	0	4.309017	9.613191	-0.771001
15	H	0	1.417736	9.296004	-1.698499
16	H	0	1.944734	10.109752	-0.226239
17	H	0	3.016079	8.459684	1.816734
18	H	0	2.917955	6.004192	2.254682
19	H	0	1.192510	6.978841	-2.784662
20	H	0	2.171327	3.603955	0.807380
21	H	0	0.766325	4.558417	-3.148579
22	H	0	1.230570	2.909662	-1.355078

23	N	0	4.483575	4.037491	-2.957948
24	C	0	4.416598	2.713202	-2.323387
25	C	0	5.759501	1.998971	-2.096955
26	O	0	5.833715	0.758145	-2.141545
27	C	0	3.443017	1.748908	-3.041045
28	C	0	3.896476	1.394793	-4.453252
29	S	0	2.620875	0.272832	-5.258441
30	C	0	3.416328	0.097629	-6.941271
31	H	0	4.675489	4.022366	-3.953632
32	H	0	3.671650	4.604741	-2.730950
33	H	0	4.026550	2.852700	-1.302121
34	H	0	2.466837	2.243526	-3.085064
35	H	0	3.330041	0.835586	-2.448171
36	H	0	3.983800	2.282989	-5.084919
37	H	0	4.848469	0.861780	-4.436829
38	H	0	3.496719	1.075229	-7.415907
39	H	0	2.773013	-0.549474	-7.535386
40	H	0	4.401818	-0.355446	-6.837991
41	N	0	6.807024	2.754513	-1.796217
42	C	0	8.071593	2.182755	-1.355474
43	C	0	7.919952	1.644655	0.047483
44	O	0	7.132861	2.206800	0.824599
45	C	0	9.164679	3.252398	-1.387705
46	C	0	9.508254	3.628330	-2.819200
47	O	0	9.430506	2.793633	-3.747398
48	N	0	9.921964	4.898268	-3.010313
49	H	0	6.668066	3.759089	-1.781629
50	H	0	8.347050	1.382810	-2.050782

51	H	0	10.071778	2.856929	-0.915727
52	H	0	8.852990	4.125898	-0.805450
53	H	0	9.969533	5.569953	-2.260469
54	H	0	10.206810	5.194176	-3.932589
55	N	0	8.685291	0.628588	0.445496
56	C	0	8.567813	0.111448	1.820046
57	C	0	8.851104	1.188808	2.856358
58	O	0	8.300800	1.174143	3.968627
59	C	0	9.497192	-1.113197	2.002636
60	C	0	10.981481	-0.722075	1.956437
61	C	0	9.165880	-1.859105	3.299683
62	H	0	9.280720	0.136839	-0.207427
63	H	0	7.526959	-0.203296	1.977546
64	H	0	9.578229	1.968171	2.584622
65	H	0	9.274814	-1.780311	1.157821
66	H	0	11.231354	-0.136038	1.066430
67	H	0	11.604498	-1.620255	1.954075
68	H	0	11.253161	-0.132193	2.839304
69	H	0	9.355445	-1.227700	4.173592
70	H	0	9.795230	-2.749456	3.383687
71	H	0	8.118238	-2.174091	3.327938
72	N	0	6.235535	-8.130633	-1.766562
73	C	0	5.731361	-6.736372	-1.761353
74	C	0	5.069878	-6.398729	-3.086330
75	O	0	5.199419	-5.258108	-3.575601
76	C	0	6.843110	-5.748108	-1.450563
77	C	0	6.382154	-4.355186	-1.067363
78	O	0	5.173095	-4.181400	-0.625041

79	O	0	7.244096	-3.419976	-1.137053
80	H	0	5.552906	-8.793732	-2.119965
81	H	0	6.593491	-8.412825	-0.860061
82	H	0	4.941387	-6.587153	-1.003247
83	H	0	7.538745	-5.666017	-2.287611
84	H	0	7.409537	-6.138609	-0.594794
85	C	0	-1.414750	-8.578532	-1.988731
86	C	0	-2.136778	-9.902972	-2.220591
87	O	0	-1.872806	-10.863765	-1.484263
88	C	0	-2.120826	-7.808366	-0.857151
89	C	0	-1.467570	-6.446176	-0.592311
90	C	0	0.057460	-6.502524	-0.440162
91	C	0	0.621073	-5.090066	-0.453524
92	N	0	2.125377	-5.062646	-0.442567
93	H	0	-0.430182	-8.902190	-1.632488
94	H	0	-2.101800	-8.431049	0.043640
95	H	0	-3.170542	-7.649772	-1.132475
96	H	0	-1.712717	-5.780120	-1.427867
97	H	0	-1.912850	-6.010526	0.311443
98	H	0	0.482802	-7.046619	-1.290768
99	H	0	0.341011	-7.028940	0.479873
100	H	0	0.278253	-4.497147	0.400067
101	H	0	0.302582	-4.570176	-1.358865
102	H	0	2.511775	-5.540479	-1.260902
103	H	0	2.505256	-5.525444	0.390005
104	H	0	2.456265	-4.016986	-0.460982
105	N	0	5.640495	-7.592651	3.292127
106	C	0	5.187471	-6.241490	3.565445

107	C	0	3.670807	-6.180584	3.680596
108	O	0	2.952103	-6.958616	3.070744
109	C	0	5.613867	-5.221553	2.490597
110	C	0	5.311601	-3.799344	2.967968
111	O	0	5.593726	-3.488768	4.155781
112	O	0	4.736684	-2.983474	2.133482
113	H	0	5.661026	-8.180834	4.117953
114	H	0	5.105394	-8.033816	2.549305
115	H	0	5.633541	-5.910314	4.507576
116	H	0	5.116267	-5.410580	1.534601
117	H	0	6.694116	-5.323909	2.338290
118	N	0	3.187107	-5.184648	4.430187
119	C	0	1.775035	-4.776951	4.350429
120	C	0	0.798563	-5.777809	4.939626
121	O	0	-0.361528	-5.847851	4.562693
122	C	0	1.352552	-4.267737	2.978342
123	C	0	0.735394	-2.882582	3.109049
124	O	0	0.248593	-2.486336	4.189807
125	N	0	0.777417	-2.139510	1.986561
126	H	0	3.833786	-4.556338	4.893970
127	H	0	1.685785	-3.922020	5.035726
128	H	0	0.617338	-4.939810	2.523727
129	H	0	2.237377	-4.211235	2.334046
130	H	0	1.376578	-2.383218	1.204309
131	C	0	-3.500064	-2.947983	3.324267
132	C	0	-4.177729	-4.124741	3.981405
133	O	0	-5.379708	-4.306665	4.171726
134	C	0	-4.440661	-2.232447	2.323794

135	C	0	-3.520765	-1.882254	1.139122
136	C	0	-2.540525	-3.063130	1.103131
137	H	0	-3.209087	-2.272105	4.142445
138	H	0	-5.227929	-2.922483	2.001442
139	H	0	-4.917364	-1.355540	2.766310
140	H	0	-2.978886	-0.950114	1.328398
141	H	0	-4.073133	-1.760211	0.203821
142	H	0	-2.990849	-3.911130	0.563752
143	H	0	-1.596569	-2.805126	0.610128
144	C	0	5.531854	4.260847	4.450872
145	C	0	4.984306	5.690787	4.425972
146	O	0	3.890466	5.943788	3.929035
147	C	0	5.869236	3.855144	2.992004
148	C	0	4.658103	3.539844	2.095767
149	C	0	4.339885	2.046776	1.939831
150	O	0	4.596620	1.270895	2.930860
151	O	0	3.770381	1.681676	0.849679
152	H	0	6.484925	4.315235	4.994812
153	H	0	6.470690	4.651947	2.539950
154	H	0	6.502368	2.967851	3.042783
155	H	0	4.802200	3.947860	1.092119
156	H	0	3.756811	4.010180	2.509085
157	C	0	-9.754226	-4.528690	-0.952919
158	C	0	-9.929966	-4.275560	-2.442341
159	O	0	-9.481537	-5.050753	-3.266506
160	C	0	-8.357866	-3.999603	-0.521427
161	C	0	-8.217302	-2.527769	-0.769685
162	C	0	-8.655162	-1.518624	0.055002

163	C	0	-7.756824	-1.894218	-1.986766
164	N	0	-8.500873	-0.295054	-0.576805
165	C	0	-7.954216	-0.494077	-1.830834
166	C	0	-7.226749	-2.378826	-3.197417
167	C	0	-7.645543	0.421357	-2.845441
168	C	0	-6.921018	-1.472998	-4.205912
169	C	0	-7.134051	-0.086502	-4.033138
170	H	0	-10.521056	-3.985078	-0.397571
171	H	0	-8.247391	-4.242657	0.538728
172	H	0	-7.594091	-4.564774	-1.070420
173	H	0	-9.079910	-1.579241	1.044877
174	H	0	-8.752227	0.596531	-0.181700
175	H	0	-7.057400	-3.439513	-3.358455
176	H	0	-7.805561	1.485168	-2.708588
177	H	0	-6.509769	-1.843047	-5.138952
178	H	0	-6.885416	0.594123	-4.839721
179	N	0	-3.148391	10.097133	-1.865387
180	C	0	-2.725376	8.724311	-2.044391
181	C	0	-3.566923	8.088930	-3.154572
182	O	0	-4.771013	7.961591	-2.982084
183	C	0	-2.833156	7.875835	-0.765092
184	C	0	-2.398633	6.432587	-1.053925
185	C	0	-1.968407	8.485029	0.341313
186	H	0	-2.835118	10.745589	-2.576108
187	H	0	-4.130568	10.204601	-1.637284
188	H	0	-1.675210	8.727339	-2.358897
189	H	0	-3.888126	7.882587	-0.453485
190	H	0	-2.435867	5.832458	-0.140022

191	H	0	-1.366622	6.414929	-1.424586
192	H	0	-3.039892	5.947543	-1.798221
193	H	0	-2.231021	9.530295	0.512690
194	H	0	-0.907804	8.433975	0.061251
195	H	0	-2.096802	7.926893	1.273521
196	C	0	-8.489528	6.254833	1.341596
197	C	0	-9.466594	5.114549	1.597037
198	O	0	-9.654875	4.687523	2.744819
199	C	0	-7.055998	5.714758	1.240160
200	C	0	-6.829623	4.641511	0.161209
201	C	0	-7.342364	5.089468	-1.214374
202	C	0	-5.331892	4.321207	0.082829
203	H	0	-8.763416	6.747110	0.405899
204	H	0	-6.762120	5.318201	2.221894
205	H	0	-6.413613	6.580103	1.034792
206	H	0	-7.370427	3.727752	0.458232
207	H	0	-6.878656	6.043410	-1.496085
208	H	0	-8.428975	5.208728	-1.229203
209	H	0	-7.075576	4.349746	-1.976172
210	H	0	-4.784980	5.212014	-0.247233
211	H	0	-5.136335	3.518819	-0.636843
212	H	0	-4.937271	4.009379	1.054308
213	N	0	-9.624197	2.174377	4.382416
214	C	0	-8.428957	2.326616	5.220766
215	C	0	-8.522493	3.563414	6.100416
216	O	0	-9.442619	4.365046	5.991466
217	C	0	-7.161672	2.477398	4.352945
218	C	0	-6.986175	1.327528	3.369765

219	S	0	-5.293621	1.489831	2.557814
220	C	0	-5.644378	0.828109	0.843868
221	H	0	-9.790054	2.990957	3.796632
222	H	0	-10.451050	1.925084	4.914773
223	H	0	-8.320576	1.447231	5.864121
224	H	0	-6.293197	2.554471	5.016314
225	H	0	-7.230779	3.419279	3.793333
226	H	0	-7.044473	0.357140	3.868346
227	H	0	-7.744688	1.367427	2.586609
228	H	0	-6.351963	1.484122	0.333915
229	H	0	-6.046224	-0.185366	0.903953
230	H	0	-4.688786	0.823552	0.313581
231	Mg	0	4.494525	-2.512754	0.297322
232	C	0	-0.559775	-0.660459	-2.187353
233	C	0	0.082488	-0.037737	-1.108404
234	C	0	1.236687	-0.602196	-0.561820
235	C	0	0.046317	-1.770512	-2.789289
236	C	0	1.197446	-2.329947	-2.244334
237	H	0	1.735406	-0.120080	0.273823
238	H	0	0.304104	-1.248050	1.966317
239	O	0	-5.216534	-4.312649	-4.889633
240	C	0	-4.077919	-3.639892	-4.316548
241	C	0	-2.989618	-3.539093	-5.392279
242	C	0	-3.553764	-4.339411	-3.061351
243	O	0	-3.225043	-5.706251	-3.352490
244	C	0	-4.539626	-4.339599	-1.891454
245	H	0	-4.932838	-4.985396	-5.537861
246	H	0	-4.436674	-2.645760	-4.032495

247	H	0	-3.399552	-3.089964	-6.299810
248	H	0	-2.609096	-4.539597	-5.622634
249	H	0	-2.149415	-2.927616	-5.054983
250	H	0	-2.612142	-3.863812	-2.767645
251	H	0	-4.043211	-6.211937	-3.530922
252	H	0	-4.785003	-3.322679	-1.568973
253	H	0	-4.117876	-4.894758	-1.050209
254	H	0	-5.469690	-4.827451	-2.205165
255	N	0	4.624323	3.269862	5.046578
256	H	0	4.554344	2.422713	4.477230
257	H	0	4.748901	3.119345	6.038019
258	O	0	5.750427	6.759505	4.987182
259	H	0	5.270715	7.603608	4.849701
260	O	0	-3.101605	-10.047460	-3.265254
261	H	0	-3.454337	-10.961938	-3.242670
262	N	0	-1.265951	-7.740371	-3.187057
263	H	0	-0.987387	-8.227400	-4.027226
264	H	0	-1.953995	-7.004109	-3.314955
265	N	0	-9.879600	-5.967557	-0.679269
266	H	0	-10.835377	-6.305363	-0.703205
267	H	0	-9.270570	-6.527907	-1.267659
268	O	0	-10.630786	-3.113295	-2.892248
269	H	0	-10.593974	-3.079288	-3.871429
270	O	0	-10.177527	4.524929	0.505464
271	H	0	-10.745813	3.798114	0.839363
272	N	0	-8.605025	7.229611	2.435859
273	H	0	-8.470516	6.804970	3.348523
274	H	0	-9.454129	7.782943	2.402359

275	N	0	3.510572	11.030883	-2.080735
276	H	0	2.880382	11.128363	-2.871158
277	H	0	4.426528	11.409580	-2.295854
278	O	0	4.574602	7.462781	-2.273970
279	H	0	4.699624	6.888286	-3.058751
280	O	0	-2.942822	7.655538	-4.365821
281	H	0	-3.621630	7.287540	-4.970827
282	O	0	-7.519359	3.815949	7.087668
283	H	0	-7.730521	4.657926	7.542275
284	O	0	4.299236	-7.388329	-3.772815
285	H	0	3.943648	-7.002242	-4.600784
286	O	0	1.228526	-6.676113	5.966667
287	H	0	0.458166	-7.217766	6.237645
288	O	0	-3.233904	-5.122992	4.377376
289	H	0	-3.689602	-5.874263	4.809846
290	N	0	-2.326228	-3.326812	2.528845
291	H	0	-1.832100	-4.158915	2.827955
292	H	0	6.650001	-2.017907	-0.672730
293	O	0	5.965042	-1.366010	-0.299612
294	H	0	5.933272	-0.513751	-0.791448
295	H	0	-0.399380	-2.220787	-3.670161
296	H	0	1.653830	-3.197024	-2.710296
297	H	0	-0.344173	0.853141	-0.671338
298	C	0	1.750017	-1.805868	-1.067172
299	O	0	2.692158	-2.528257	-0.372800
300	N	0	-1.818027	-0.238250	-2.689538
301	H	0	-2.046307	-0.546519	-3.625397
302	C	0	-4.154842	0.429826	-2.664947

303	H	0	-4.642252	1.375966	-2.424905
304	H	0	-4.088015	0.325713	-3.749440
305	H	0	-4.797046	-0.375664	-2.286508
306	C	0	-2.817524	0.361439	-1.976942
307	O	0	-2.663350	0.783856	-0.807050

Aldehyde Dehydrogenase: Overall Charge = 0, Total energy = -6684.31117988 Ha

1	N	0	5.721201	0.855491	-6.964376
2	C	0	6.067918	0.983073	-5.551739
3	C	0	7.333301	0.253920	-5.154475
4	O	0	8.160696	0.805251	-4.398809
5	H	0	6.390283	1.267719	-7.601994
6	H	0	5.439398	-0.074703	-7.246170
7	H	0	6.233475	2.026733	-5.288084
8	H	0	5.244683	0.602059	-4.939330
9	N	0	9.706309	2.701880	-2.821293
10	C	0	10.644285	2.362630	-1.766505
11	C	0	11.788783	1.461667	-2.169036
12	O	0	12.879581	1.423710	-1.599314
13	C	0	9.857930	1.662668	-0.607069
14	O	0	8.536938	2.249932	-0.552516
15	C	0	10.538348	1.827103	0.737719
16	H	0	9.414948	1.925710	-3.413614
17	H	0	9.951581	3.526227	-3.355983
18	H	0	11.091355	3.278664	-1.374573
19	H	0	9.754450	0.600023	-0.872407
20	H	0	8.379609	2.611736	-1.468780

21	H	0	10.633863	2.890669	0.976356
22	H	0	11.535297	1.378453	0.723444
23	H	0	9.950378	1.354014	1.527810
24	N	0	1.134122	-8.083571	0.057093
25	C	0	1.697730	-7.738960	-1.246765
26	C	0	3.093989	-8.340333	-1.251805
27	O	0	4.014535	-7.788788	-0.643421
28	C	0	1.766602	-6.225523	-1.541004
29	C	0	0.379315	-5.634618	-1.539738
30	C	0	-0.166383	-5.100230	-0.364465
31	C	0	-0.417697	-5.692277	-2.691298
32	C	0	-1.486162	-4.644384	-0.335150
33	C	0	-1.736557	-5.236041	-2.666811
34	C	0	-2.274363	-4.717470	-1.485346
35	H	0	1.582407	-7.607974	0.834003
36	H	0	0.124100	-8.023652	0.100721
37	H	0	1.097683	-8.234246	-2.014648
38	H	0	2.258565	-6.063335	-2.507612
39	H	0	2.387179	-5.762414	-0.766457
40	H	0	0.446506	-5.046348	0.530077
41	H	0	-0.002286	-6.103155	-3.606786
42	H	0	-1.907114	-4.239866	0.579147
43	H	0	-2.344830	-5.292099	-3.563148
44	H	0	-3.304692	-4.380315	-1.452181
45	N	0	6.030768	-6.002775	-2.980952
46	C	0	6.122360	-4.727342	-2.254755
47	C	0	6.697553	-4.954590	-0.864373
48	O	0	7.603576	-4.231249	-0.441710

49	C	0	4.753223	-4.024583	-2.170274
50	C	0	4.818182	-2.775947	-1.282688
51	C	0	4.264293	-3.674335	-3.577833
52	H	0	5.463594	-6.687284	-2.485684
53	H	0	6.927104	-6.385172	-3.259950
54	H	0	6.838681	-4.071958	-2.757124
55	H	0	4.061318	-4.746728	-1.718006
56	H	0	5.035584	-3.011030	-0.235352
57	H	0	3.862506	-2.244606	-1.306266
58	H	0	5.595073	-2.087818	-1.639381
59	H	0	3.255396	-3.252542	-3.534308
60	H	0	4.253345	-4.559453	-4.216413
61	H	0	4.926854	-2.926799	-4.032458
62	N	0	10.009131	-3.689633	0.960272
63	C	0	9.917086	-2.880263	2.198522
64	C	0	10.810952	-3.433064	3.291555
65	O	0	11.307534	-2.686986	4.116496
66	C	0	8.451789	-2.816925	2.671220
67	C	0	7.671026	-1.790900	1.899463
68	C	0	8.180453	-0.807690	1.088085
69	C	0	6.240116	-1.595699	1.916856
70	N	0	7.164100	0.002356	0.607274
71	C	0	5.959833	-0.461203	1.100913
72	C	0	5.175450	-2.278054	2.532331
73	C	0	4.651018	-0.002616	0.897038
74	C	0	3.877849	-1.828457	2.323019
75	C	0	3.618166	-0.699560	1.512526
76	H	0	9.141805	-3.749682	0.440694

77	H	0	10.817351	-3.483063	0.386942
78	H	0	10.289301	-1.854439	2.082708
79	H	0	8.014636	-3.816484	2.553214
80	H	0	8.421460	-2.577393	3.742004
81	H	0	9.201765	-0.628606	0.797318
82	H	0	7.339820	0.854719	0.075834
83	H	0	5.365251	-3.145369	3.156741
84	H	0	4.451368	0.860250	0.271382
85	H	0	3.046882	-2.348674	2.785959
86	H	0	2.592730	-0.376940	1.362720
87	N	0	-12.515366	2.855991	-1.828698
88	C	0	-11.244055	2.254094	-2.203404
89	C	0	-10.506948	1.790617	-0.949972
90	O	0	-9.884832	0.730845	-0.943254
91	C	0	-10.361494	3.288507	-2.941069
92	C	0	-10.957017	3.710671	-4.239990
93	N	0	-10.976666	2.905896	-5.369541
94	C	0	-11.619803	4.860382	-4.608216
95	C	0	-11.631305	3.583922	-6.363227
96	N	0	-12.035538	4.774703	-5.936016
97	H	0	-13.220623	2.198879	-1.515393
98	H	0	-12.880848	3.503080	-2.520065
99	H	0	-11.341350	1.357740	-2.832413
100	H	0	-9.361536	2.861981	-3.077879
101	H	0	-10.257564	4.168602	-2.298183
102	H	0	-10.566921	1.987429	-5.451844
103	H	0	-11.808287	5.734773	-4.008019
104	H	0	-11.779580	3.179669	-7.348883

105	N	0	-10.618570	2.575579	0.113463
106	C	0	-10.014717	2.243731	1.395604
107	C	0	-10.328857	0.857403	1.929298
108	O	0	-9.469284	0.230735	2.527808
109	H	0	-11.287733	3.335294	0.038580
110	H	0	-10.351896	2.979560	2.129027
111	H	0	-8.925166	2.304050	1.338540
112	N	0	-9.795740	-2.201301	-1.590699
113	C	0	-8.538801	-2.358569	-2.307414
114	C	0	-7.988418	-3.776793	-2.153864
115	O	0	-7.977885	-4.330805	-1.050182
116	C	0	-7.432175	-1.385678	-1.798859
117	C	0	-6.201175	-1.288757	-2.663423
118	C	0	-6.104777	-0.273943	-3.627567
119	C	0	-5.134256	-2.187357	-2.543631
120	C	0	-4.983041	-0.154184	-4.442446
121	C	0	-3.998475	-2.075770	-3.347563
122	C	0	-3.928037	-1.055507	-4.296977
123	O	0	-2.831905	-0.881895	-5.126998
124	H	0	-10.079177	-1.224879	-1.536801
125	H	0	-9.761001	-2.622979	-0.665599
126	H	0	-8.697153	-2.167304	-3.372892
127	H	0	-7.911367	-0.406858	-1.760131
128	H	0	-7.180322	-1.680810	-0.771614
129	H	0	-6.913121	0.445987	-3.717335
130	H	0	-5.194196	-2.994216	-1.817147
131	H	0	-4.902455	0.634227	-5.180839
132	H	0	-3.173401	-2.771889	-3.233656

133	H	0	-2.132796	-1.542100	-4.969120
134	N	0	-5.885607	-5.808061	2.833908
135	C	0	-4.917223	-4.807912	3.259456
136	C	0	-5.047679	-4.598393	4.746635
137	O	0	-6.153265	-4.402231	5.254710
138	C	0	-5.098933	-3.386574	2.677110
139	S	0	-4.832895	-3.422589	0.821963
140	H	0	-6.852894	-5.529010	2.952847
141	H	0	-5.693885	-6.217076	1.928777
142	H	0	-3.911932	-5.168746	3.029185
143	H	0	-6.108728	-3.031143	2.890494
144	H	0	-4.370538	-2.699221	3.113981
145	H	0	-5.059126	-2.088844	0.586657
146	N	0	-4.469943	-1.971844	6.318702
147	C	0	-5.065280	-0.658482	6.566669
148	C	0	-6.500849	-0.499877	6.076689
149	O	0	-7.009598	0.618343	6.068263
150	C	0	-4.171480	0.492915	6.051988
151	C	0	-4.051921	0.521364	4.516002
152	C	0	-2.801459	0.409664	6.738733
153	C	0	-3.166795	1.673728	4.023854
154	H	0	-5.189977	-2.688038	6.297050
155	H	0	-3.866800	-2.018117	5.504898
156	H	0	-5.151523	-0.519670	7.655561
157	H	0	-4.664224	1.422549	6.359894
158	H	0	-5.055707	0.618101	4.081296
159	H	0	-3.633469	-0.426664	4.148914
160	H	0	-2.226695	1.322935	6.569222

161	H	0	-2.220687	-0.436254	6.356815
162	H	0	-2.917061	0.278660	7.818906
163	H	0	-3.495336	2.624557	4.459648
164	H	0	-3.203518	1.767458	2.933592
165	H	0	-2.120768	1.519590	4.303076
166	N	0	-5.422753	5.699114	2.416933
167	C	0	-4.585873	4.696199	1.767393
168	C	0	-3.403373	5.262046	1.004010
169	O	0	-2.912245	4.618152	0.083796
170	H	0	-4.945347	6.228700	3.135991
171	H	0	-5.920892	6.301804	1.773713
172	H	0	-5.162334	4.107373	1.049648
173	H	0	-4.183418	4.003286	2.514318
174	N	0	0.268596	5.928020	0.834424
175	C	0	1.448132	5.344254	1.449435
176	C	0	2.607029	5.631264	0.500346
177	O	0	2.481845	5.450952	-0.694392
178	C	0	1.254344	3.830411	1.659040
179	C	0	2.532825	3.184007	2.205149
180	C	0	0.063524	3.561311	2.584531
181	H	0	0.053316	5.463051	-0.046340
182	H	0	0.379396	6.926683	0.667217
183	H	0	1.624482	5.821157	2.420964
184	H	0	1.034157	3.391781	0.674290
185	H	0	2.378213	2.112930	2.362947
186	H	0	2.807496	3.631662	3.168281
187	H	0	3.379145	3.303499	1.520355
188	H	0	0.265178	3.950556	3.590098

189	H	0	-0.114369	2.484586	2.648570
190	H	0	-0.840795	4.047460	2.206337
191	N	0	3.715279	6.125810	1.024807
192	C	0	4.855288	6.444681	0.177314
193	C	0	6.141835	6.093324	0.914194
194	O	0	6.167259	6.023743	2.142841
195	C	0	4.866785	7.952343	-0.160960
196	O	0	6.052834	8.313447	-0.899131
197	H	0	3.852271	6.218250	2.023794
198	H	0	4.772497	5.869866	-0.747357
199	H	0	4.781643	8.525756	0.768154
200	H	0	4.010685	8.177907	-0.797379
201	H	0	6.810865	8.459265	-0.298153
202	N	0	7.218132	5.897796	0.152357
203	C	0	8.511573	5.508618	0.720767
204	C	0	9.080814	6.531548	1.722582
205	O	0	9.869726	6.173135	2.621412
206	C	0	9.527410	5.380958	-0.420894
207	H	0	7.158825	6.035072	-0.848121
208	H	0	8.417630	4.570209	1.273063
209	H	0	9.639597	6.339389	-0.935621
210	H	0	9.184548	4.626810	-1.130539
211	H	0	10.500444	5.083308	-0.025468
212	C	0	-3.596113	1.565844	-0.123582
213	C	0	-3.941137	2.294036	-1.268739
214	C	0	-6.261447	1.764530	-0.917514
215	O	0	11.073922	-4.837011	3.357714
216	H	0	11.646461	-5.004755	4.135815

217	O	0	7.581240	-1.066368	-5.644080
218	H	0	8.441619	-1.383455	-5.296706
219	O	0	-7.471791	-4.312807	-3.280235
220	H	0	-7.061489	-5.191216	-3.131065
221	O	0	-2.861334	6.538673	1.352163
222	H	0	-1.905872	6.542350	1.082088
223	O	0	8.695317	7.904228	1.614257
224	H	0	9.149414	8.424751	2.309905
225	O	0	3.335581	-9.553919	-1.968572
226	H	0	4.270871	-9.819930	-1.841084
227	O	0	-3.884232	-4.626022	5.577507
228	H	0	-4.115788	-4.415137	6.506998
229	O	0	-7.236733	-1.643836	5.635662
230	H	0	-8.136381	-1.363660	5.363460
231	O	0	6.184704	-6.010197	-0.047344
232	H	0	6.661465	-6.009122	0.810033
233	O	0	11.462231	0.631391	-3.192710
234	H	0	12.186009	0.014755	-3.429043
235	O	0	-11.595244	0.442821	1.713604
236	H	0	-11.774670	-0.452658	2.069028
237	C	0	-5.931092	1.006689	0.207845
238	O	0	-7.560227	1.925521	-1.353794
239	H	0	-8.211143	1.305616	-0.951802
240	H	0	-6.707545	0.512848	0.784893
241	H	0	-3.161527	2.795250	-1.827799
242	C	0	-5.266957	2.405616	-1.657835
243	H	0	-5.551964	2.969073	-2.537523
244	C	0	-4.594888	0.919885	0.609406

245	H	0	-4.340678	0.371534	1.510724
246	N	0	-2.233610	1.584560	0.296355
247	H	0	-1.807175	2.506179	0.354742
248	C	0	-1.973165	-0.865776	0.398669
249	H	0	-2.699519	-0.938990	-0.414174
250	H	0	-1.145419	-1.550602	0.215180
251	H	0	-2.468110	-1.174929	1.325962
252	C	0	-1.410464	0.525155	0.544670
253	O	0	-0.217755	0.712499	0.886782

Sult1a3: Overall Charge = +1, Total energy = -5483.52654897 Ha

1	N	0	5.721201	0.855491	-6.964376
2	C	0	6.067918	0.983073	-5.551739
3	C	0	7.333301	0.253920	-5.154475
4	O	0	8.160696	0.805251	-4.398809
5	H	0	6.390283	1.267719	-7.601994
6	H	0	5.439398	-0.074703	-7.246170
7	H	0	6.233475	2.026733	-5.288084
8	H	0	5.244683	0.602059	-4.939330
9	N	0	9.706309	2.701880	-2.821293
10	C	0	10.644285	2.362630	-1.766505
11	C	0	11.788783	1.461667	-2.169036
12	O	0	12.879581	1.423710	-1.599314
13	C	0	9.857930	1.662668	-0.607069
14	O	0	8.536938	2.249932	-0.552516
15	C	0	10.538348	1.827103	0.737719
16	H	0	9.414948	1.925710	-3.413614

17	H	0	9.951581	3.526227	-3.355983
18	H	0	11.091355	3.278664	-1.374573
19	H	0	9.754450	0.600023	-0.872407
20	H	0	8.379609	2.611736	-1.468780
21	H	0	10.633863	2.890669	0.976356
22	H	0	11.535297	1.378453	0.723444
23	H	0	9.950378	1.354014	1.527810
24	N	0	1.134122	-8.083571	0.057093
25	C	0	1.697730	-7.738960	-1.246765
26	C	0	3.093989	-8.340333	-1.251805
27	O	0	4.014535	-7.788788	-0.643421
28	C	0	1.766602	-6.225523	-1.541004
29	C	0	0.379315	-5.634618	-1.539738
30	C	0	-0.166383	-5.100230	-0.364465
31	C	0	-0.417697	-5.692277	-2.691298
32	C	0	-1.486162	-4.644384	-0.335150
33	C	0	-1.736557	-5.236041	-2.666811
34	C	0	-2.274363	-4.717470	-1.485346
35	H	0	1.582407	-7.607974	0.834003
36	H	0	0.124100	-8.023652	0.100721
37	H	0	1.097683	-8.234246	-2.014648
38	H	0	2.258565	-6.063335	-2.507612
39	H	0	2.387179	-5.762414	-0.766457
40	H	0	0.446506	-5.046348	0.530077
41	H	0	-0.002286	-6.103155	-3.606786
42	H	0	-1.907114	-4.239866	0.579147
43	H	0	-2.344830	-5.292099	-3.563148
44	H	0	-3.304692	-4.380315	-1.452181

45	N	0	6.030768	-6.002775	-2.980952
46	C	0	6.122360	-4.727342	-2.254755
47	C	0	6.697553	-4.954590	-0.864373
48	O	0	7.603576	-4.231249	-0.441710
49	C	0	4.753223	-4.024583	-2.170274
50	C	0	4.818182	-2.775947	-1.282688
51	C	0	4.264293	-3.674335	-3.577833
52	H	0	5.463594	-6.687284	-2.485684
53	H	0	6.927104	-6.385172	-3.259950
54	H	0	6.838681	-4.071958	-2.757124
55	H	0	4.061318	-4.746728	-1.718006
56	H	0	5.035584	-3.011030	-0.235352
57	H	0	3.862506	-2.244606	-1.306266
58	H	0	5.595073	-2.087818	-1.639381
59	H	0	3.255396	-3.252542	-3.534308
60	H	0	4.253345	-4.559453	-4.216413
61	H	0	4.926854	-2.926799	-4.032458
62	N	0	10.009131	-3.689633	0.960272
63	C	0	9.917086	-2.880263	2.198522
64	C	0	10.810952	-3.433064	3.291555
65	O	0	11.307534	-2.686986	4.116496
66	C	0	8.451789	-2.816925	2.671220
67	C	0	7.671026	-1.790900	1.899463
68	C	0	8.180453	-0.807690	1.088085
69	C	0	6.240116	-1.595699	1.916856
70	N	0	7.164100	0.002356	0.607274
71	C	0	5.959833	-0.461203	1.100913
72	C	0	5.175450	-2.278054	2.532331

73	C	0	4.651018	-0.002616	0.897038
74	C	0	3.877849	-1.828457	2.323019
75	C	0	3.618166	-0.699560	1.512526
76	H	0	9.141805	-3.749682	0.440694
77	H	0	10.817351	-3.483063	0.386942
78	H	0	10.289301	-1.854439	2.082708
79	H	0	8.014636	-3.816484	2.553214
80	H	0	8.421460	-2.577393	3.742004
81	H	0	9.201765	-0.628606	0.797318
82	H	0	7.339820	0.854719	0.075834
83	H	0	5.365251	-3.145369	3.156741
84	H	0	4.451368	0.860250	0.271382
85	H	0	3.046882	-2.348674	2.785959
86	H	0	2.592730	-0.376940	1.362720
87	N	0	-12.515366	2.855991	-1.828698
88	C	0	-11.244055	2.254094	-2.203404
89	C	0	-10.506948	1.790617	-0.949972
90	O	0	-9.884832	0.730845	-0.943254
91	C	0	-10.361494	3.288507	-2.941069
92	C	0	-10.957017	3.710671	-4.239990
93	N	0	-10.976666	2.905896	-5.369541
94	C	0	-11.619803	4.860382	-4.608216
95	C	0	-11.631305	3.583922	-6.363227
96	N	0	-12.035538	4.774703	-5.936016
97	H	0	-13.220623	2.198879	-1.515393
98	H	0	-12.880848	3.503080	-2.520065
99	H	0	-11.341350	1.357740	-2.832413
100	H	0	-9.361536	2.861981	-3.077879

101	H	0	-10.257564	4.168602	-2.298183
102	H	0	-10.566921	1.987429	-5.451844
103	H	0	-11.808287	5.734773	-4.008019
104	H	0	-11.779580	3.179669	-7.348883
105	N	0	-10.618570	2.575579	0.113463
106	C	0	-10.014717	2.243731	1.395604
107	C	0	-10.328857	0.857403	1.929298
108	O	0	-9.469284	0.230735	2.527808
109	H	0	-11.287733	3.335294	0.038580
110	H	0	-10.351896	2.979560	2.129027
111	H	0	-8.925166	2.304050	1.338540
112	N	0	-9.795740	-2.201301	-1.590699
113	C	0	-8.538801	-2.358569	-2.307414
114	C	0	-7.988418	-3.776793	-2.153864
115	O	0	-7.977885	-4.330805	-1.050182
116	C	0	-7.432175	-1.385678	-1.798859
117	C	0	-6.201175	-1.288757	-2.663423
118	C	0	-6.104777	-0.273943	-3.627567
119	C	0	-5.134256	-2.187357	-2.543631
120	C	0	-4.983041	-0.154184	-4.442446
121	C	0	-3.998475	-2.075770	-3.347563
122	C	0	-3.928037	-1.055507	-4.296977
123	O	0	-2.831905	-0.881895	-5.126998
124	H	0	-10.079177	-1.224879	-1.536801
125	H	0	-9.761001	-2.622979	-0.665599
126	H	0	-8.697153	-2.167304	-3.372892
127	H	0	-7.911367	-0.406858	-1.760131
128	H	0	-7.180322	-1.680810	-0.771614

129	H	0	-6.913121	0.445987	-3.717335
130	H	0	-5.194196	-2.994216	-1.817147
131	H	0	-4.902455	0.634227	-5.180839
132	H	0	-3.173401	-2.771889	-3.233656
133	H	0	-2.132796	-1.542100	-4.969120
134	N	0	-5.885607	-5.808061	2.833908
135	C	0	-4.917223	-4.807912	3.259456
136	C	0	-5.047679	-4.598393	4.746635
137	O	0	-6.153265	-4.402231	5.254710
138	C	0	-5.098933	-3.386574	2.677110
139	S	0	-4.832895	-3.422589	0.821963
140	H	0	-6.852894	-5.529010	2.952847
141	H	0	-5.693885	-6.217076	1.928777
142	H	0	-3.911932	-5.168746	3.029185
143	H	0	-6.108728	-3.031143	2.890494
144	H	0	-4.370538	-2.699221	3.113981
145	H	0	-5.059126	-2.088844	0.586657
146	N	0	-4.469943	-1.971844	6.318702
147	C	0	-5.065280	-0.658482	6.566669
148	C	0	-6.500849	-0.499877	6.076689
149	O	0	-7.009598	0.618343	6.068263
150	C	0	-4.171480	0.492915	6.051988
151	C	0	-4.051921	0.521364	4.516002
152	C	0	-2.801459	0.409664	6.738733
153	C	0	-3.166795	1.673728	4.023854
154	H	0	-5.189977	-2.688038	6.297050
155	H	0	-3.866800	-2.018117	5.504898
156	H	0	-5.151523	-0.519670	7.655561

157	H	0	-4.664224	1.422549	6.359894
158	H	0	-5.055707	0.618101	4.081296
159	H	0	-3.633469	-0.426664	4.148914
160	H	0	-2.226695	1.322935	6.569222
161	H	0	-2.220687	-0.436254	6.356815
162	H	0	-2.917061	0.278660	7.818906
163	H	0	-3.495336	2.624557	4.459648
164	H	0	-3.203518	1.767458	2.933592
165	H	0	-2.120768	1.519590	4.303076
166	N	0	-5.422753	5.699114	2.416933
167	C	0	-4.585873	4.696199	1.767393
168	C	0	-3.403373	5.262046	1.004010
169	O	0	-2.912245	4.618152	0.083796
170	H	0	-4.945347	6.228700	3.135991
171	H	0	-5.920892	6.301804	1.773713
172	H	0	-5.162334	4.107373	1.049648
173	H	0	-4.183418	4.003286	2.514318
174	N	0	0.268596	5.928020	0.834424
175	C	0	1.448132	5.344254	1.449435
176	C	0	2.607029	5.631264	0.500346
177	O	0	2.481845	5.450952	-0.694392
178	C	0	1.254344	3.830411	1.659040
179	C	0	2.532825	3.184007	2.205149
180	C	0	0.063524	3.561311	2.584531
181	H	0	0.053316	5.463051	-0.046340
182	H	0	0.379396	6.926683	0.667217
183	H	0	1.624482	5.821157	2.420964
184	H	0	1.034157	3.391781	0.674290

185	H	0	2.378213	2.112930	2.362947
186	H	0	2.807496	3.631662	3.168281
187	H	0	3.379145	3.303499	1.520355
188	H	0	0.265178	3.950556	3.590098
189	H	0	-0.114369	2.484586	2.648570
190	H	0	-0.840795	4.047460	2.206337
191	N	0	3.715279	6.125810	1.024807
192	C	0	4.855288	6.444681	0.177314
193	C	0	6.141835	6.093324	0.914194
194	O	0	6.167259	6.023743	2.142841
195	C	0	4.866785	7.952343	-0.160960
196	O	0	6.052834	8.313447	-0.899131
197	H	0	3.852271	6.218250	2.023794
198	H	0	4.772497	5.869866	-0.747357
199	H	0	4.781643	8.525756	0.768154
200	H	0	4.010685	8.177907	-0.797379
201	H	0	6.810865	8.459265	-0.298153
202	N	0	7.218132	5.897796	0.152357
203	C	0	8.511573	5.508618	0.720767
204	C	0	9.080814	6.531548	1.722582
205	O	0	9.869726	6.173135	2.621412
206	C	0	9.527410	5.380958	-0.420894
207	H	0	7.158825	6.035072	-0.848121
208	H	0	8.417630	4.570209	1.273063
209	H	0	9.639597	6.339389	-0.935621
210	H	0	9.184548	4.626810	-1.130539
211	H	0	10.500444	5.083308	-0.025468
212	C	0	-3.596113	1.565844	-0.123582

213	C	0	-3.941137	2.294036	-1.268739
214	C	0	-6.261447	1.764530	-0.917514
215	O	0	11.073922	-4.837011	3.357714
216	H	0	11.646461	-5.004755	4.135815
217	O	0	7.581240	-1.066368	-5.644080
218	H	0	8.441619	-1.383455	-5.296706
219	O	0	-7.471791	-4.312807	-3.280235
220	H	0	-7.061489	-5.191216	-3.131065
221	O	0	-2.861334	6.538673	1.352163
222	H	0	-1.905872	6.542350	1.082088
223	O	0	8.695317	7.904228	1.614257
224	H	0	9.149414	8.424751	2.309905
225	O	0	3.335581	-9.553919	-1.968572
226	H	0	4.270871	-9.819930	-1.841084
227	O	0	-3.884232	-4.626022	5.577507
228	H	0	-4.115788	-4.415137	6.506998
229	O	0	-7.236733	-1.643836	5.635662
230	H	0	-8.136381	-1.363660	5.363460
231	O	0	6.184704	-6.010197	-0.047344
232	H	0	6.661465	-6.009122	0.810033
233	O	0	11.462231	0.631391	-3.192710
234	H	0	12.186009	0.014755	-3.429043
235	O	0	-11.595244	0.442821	1.713604
236	H	0	-11.774670	-0.452658	2.069028
237	C	0	-5.931092	1.006689	0.207845
238	O	0	-7.560227	1.925521	-1.353794
239	H	0	-8.211143	1.305616	-0.951802
240	H	0	-6.707545	0.512848	0.784893

241	H	0	-3.161527	2.795250	-1.827799
242	C	0	-5.266957	2.405616	-1.657835
243	H	0	-5.551964	2.969073	-2.537523
244	C	0	-4.594888	0.919885	0.609406
245	H	0	-4.340678	0.371534	1.510724
246	N	0	-2.233610	1.584560	0.296355
247	H	0	-1.807175	2.506179	0.354742
248	C	0	-1.973165	-0.865776	0.398669
249	H	0	-2.699519	-0.938990	-0.414174
250	H	0	-1.145419	-1.550602	0.215180
251	H	0	-2.468110	-1.174929	1.325962
252	C	0	-1.410464	0.525155	0.544670
253	O	0	-0.217755	0.712499	0.886782

2. Optimized coordinates for NAPQI in the active sites labelled below. Optimizations carried out with M062X/6-31G and implicit solvent using the PCM model in the Gaussian 16 software (see manuscript for references). Columns are: atom number, atom symbol, atom type (gaussian 16), and X, Y and Z coordinates in Angstroms.

Phenylalanine Hydroxylase: Overall Charge = 0, Total energy = -7285.97977557 Ha

1	N	0	-4.092695	9.209673	-0.855673
2	C	0	-3.134120	9.171225	-1.966333
3	C	0	-3.758984	9.747584	-3.235718
4	O	0	-4.040767	9.114415	-4.254291
5	C	0	-2.572702	7.759705	-2.229056
6	C	0	-1.606027	7.368380	-1.139048
7	C	0	-2.056910	6.827290	0.072731
8	C	0	-0.234607	7.618009	-1.291327
9	C	0	-1.160308	6.548428	1.106397
10	C	0	0.669261	7.335784	-0.269540
11	C	0	0.203936	6.802980	0.933304
12	O	0	1.137946	6.568542	1.930594
13	H	0	-3.685200	9.519014	0.016761
14	H	0	-4.626574	8.340604	-0.747441
15	H	0	-2.303024	9.843977	-1.722815
16	H	0	-3.418023	7.062412	-2.268547
17	H	0	-2.073919	7.737300	-3.203248
18	H	0	-3.115425	6.621896	0.217148
19	H	0	0.129548	8.036434	-2.225260
20	H	0	-1.515409	6.131269	2.042545
21	H	0	1.731206	7.516538	-0.386868
22	H	0	0.787430	5.959669	2.615616
23	N	0	-6.850089	-0.640761	4.749928
24	C	0	-7.020797	-0.485606	3.272679
25	C	0	-8.483352	-0.219250	3.006780

26	O	0	-9.362331	-0.265504	3.866037
27	C	0	-6.510231	-1.690613	2.470207
28	C	0	-7.264029	-2.990348	2.781468
29	C	0	-6.799119	-4.177393	1.933536
30	N	0	-5.384098	-4.466531	2.190251
31	C	0	-4.587765	-5.227327	1.416724
32	N	0	-5.037840	-5.736224	0.259264
33	N	0	-3.337926	-5.466864	1.815694
34	H	0	-6.195578	-1.398071	4.977290
35	H	0	-7.752011	-0.764582	5.221895
36	H	0	-6.448926	0.399029	2.969769
37	H	0	-5.435897	-1.782642	2.667460
38	H	0	-6.622650	-1.422805	1.414799
39	H	0	-8.335205	-2.858938	2.589606
40	H	0	-7.168037	-3.254030	3.842675
41	H	0	-7.412922	-5.055103	2.161403
42	H	0	-6.934896	-3.934296	0.874410
43	H	0	-4.996721	-4.171839	3.079036
44	H	0	-5.992708	-5.633742	-0.044251
45	H	0	-4.422895	-6.285334	-0.323470
46	H	0	-2.717001	-6.072795	1.272196
47	H	0	-3.059143	-5.129371	2.754048
48	N	0	-4.707823	-5.389703	-5.265519
49	C	0	-3.904115	-4.228818	-4.925161
50	C	0	-4.851285	-3.088631	-4.551677
51	O	0	-5.953865	-2.943519	-5.135981
52	C	0	-2.988495	-3.723240	-6.088431
53	C	0	-2.258528	-2.451154	-5.729073
54	C	0	-1.137543	-2.497240	-4.885459
55	C	0	-2.753785	-1.199809	-6.117483
56	C	0	-0.533883	-1.323503	-4.439308

57	C	0	-2.157325	-0.016326	-5.675802
58	C	0	-1.049202	-0.091637	-4.834726
59	O	0	-0.410914	1.044856	-4.332053
60	H	0	-5.505497	-5.144698	-5.843877
61	H	0	-4.174634	-6.163706	-5.644422
62	H	0	-3.260141	-4.461642	-4.068730
63	H	0	-2.280155	-4.527472	-6.316130
64	H	0	-3.627238	-3.570265	-6.966065
65	H	0	-0.739966	-3.465098	-4.588096
66	H	0	-3.629074	-1.146157	-6.758471
67	H	0	0.327560	-1.348308	-3.783594
68	H	0	-2.554656	0.945942	-5.983428
69	H	0	-0.646572	1.869319	-4.795151
70	N	0	-4.406625	-2.264858	-3.605828
71	C	0	-5.152026	-1.085788	-3.175320
72	C	0	-4.129562	-0.000995	-2.851472
73	O	0	-3.090051	-0.296087	-2.202470
74	C	0	-5.885949	-1.360572	-1.800512
75	O	0	-5.919772	-0.138166	-1.003499
76	C	0	-5.197506	-2.468833	-1.013589
77	H	0	-3.478001	-2.371458	-3.212660
78	H	0	-5.853726	-0.808044	-3.958752
79	H	0	-6.928579	-1.615249	-2.000319
80	H	0	-5.204302	-0.106996	-0.315132
81	H	0	-4.131474	-2.246829	-0.897302
82	H	0	-5.298546	-3.434451	-1.510570
83	H	0	-5.640045	-2.541060	-0.018963
84	N	0	-4.402256	1.243733	-3.256303
85	C	0	-3.449718	2.328381	-2.984371
86	C	0	-3.468125	2.788739	-1.529140
87	O	0	-2.517827	3.484552	-1.095105

88	C	0	-3.920108	3.450088	-3.931867
89	C	0	-5.439511	3.226338	-4.014562
90	C	0	-5.588288	1.696633	-4.020188
91	H	0	-2.430203	1.991066	-3.187449
92	H	0	-3.457618	3.312473	-4.913792
93	H	0	-3.647309	4.438226	-3.557896
94	H	0	-5.930057	3.649844	-3.132553
95	H	0	-5.884819	3.679750	-4.900529
96	H	0	-5.553405	1.293453	-5.037722
97	H	0	-6.508899	1.365617	-3.533699
98	N	0	-4.549051	2.460814	-0.794031
99	C	0	-4.680729	2.823277	0.612832
100	C	0	-4.094213	1.758994	1.533271
101	O	0	-4.148348	0.529413	1.212120
102	C	0	-6.130380	3.201996	0.949823
103	C	0	-6.557198	4.439418	0.148869
104	C	0	-5.636233	5.662231	0.346534
105	O	0	-5.026863	5.772705	1.465737
106	O	0	-5.562658	6.496417	-0.625777
107	H	0	-5.225996	1.803066	-1.177463
108	H	0	-4.086742	3.734568	0.744393
109	H	0	-6.191720	3.415368	2.021504
110	H	0	-6.792508	2.351771	0.739750
111	H	0	-6.594165	4.214406	-0.920573
112	H	0	-7.571910	4.731242	0.447882
113	N	0	-3.670500	2.167725	2.740809
114	C	0	-3.100967	1.220387	3.702679
115	C	0	-4.179199	0.506911	4.547278
116	O	0	-4.063329	-0.740656	4.757236
117	C	0	-2.222664	2.115234	4.602658
118	C	0	-2.943774	3.472967	4.577865

119	C	0	-3.459738	3.583890	3.140388
120	H	0	-2.525708	0.451019	3.181997
121	H	0	-1.221346	2.198583	4.167799
122	H	0	-2.119802	1.707626	5.610945
123	H	0	-2.277889	4.301045	4.827328
124	H	0	-3.780598	3.461290	5.279605
125	H	0	-4.370474	4.174110	3.029830
126	H	0	-2.696704	4.034123	2.493318
127	N	0	-2.771148	-4.625489	4.544601
128	C	0	-1.427394	-4.080766	4.462932
129	C	0	-0.755970	-4.012694	5.840284
130	O	0	0.350907	-4.472450	6.111234
131	C	0	-1.502556	-2.705135	3.790557
132	C	0	-0.211466	-1.982729	3.883872
133	N	0	0.956960	-2.425820	3.287778
134	C	0	0.147387	-0.900333	4.654353
135	C	0	1.966003	-1.596180	3.694471
136	N	0	1.510250	-0.664353	4.531925
137	H	0	-3.404096	-3.996460	5.035769
138	H	0	-2.819941	-5.556681	4.953199
139	H	0	-0.790685	-4.734301	3.880427
140	H	0	-2.285510	-2.101002	4.266087
141	H	0	-1.807697	-2.884083	2.753975
142	H	0	1.008285	-3.243404	2.666984
143	H	0	-0.487432	-0.303517	5.289199
144	H	0	2.983247	-1.656387	3.346218
145	N	0	10.245027	4.287988	2.313830
146	C	0	9.447950	3.775415	1.219289
147	C	0	10.198720	2.738296	0.389031
148	O	0	10.140683	1.516482	0.561640
149	C	0	8.852826	4.855916	0.286808

150	C	0	7.871082	4.279735	-0.688778
151	C	0	8.129458	3.737746	-1.926214
152	C	0	6.449517	4.124966	-0.462083
153	N	0	6.951792	3.264586	-2.486809
154	C	0	5.905979	3.487444	-1.609707
155	C	0	5.590045	4.483517	0.591873
156	C	0	4.541457	3.188448	-1.718831
157	C	0	4.238084	4.178346	0.493392
158	C	0	3.720578	3.530318	-0.650639
159	H	0	10.891765	5.029054	2.073984
160	H	0	9.732606	4.461303	3.166402
161	H	0	8.616776	3.223865	1.667814
162	H	0	9.673187	5.364978	-0.229217
163	H	0	8.358267	5.593807	0.927948
164	H	0	9.064834	3.661448	-2.457476
165	H	0	6.876510	2.817068	-3.385862
166	H	0	5.977507	4.990018	1.470537
167	H	0	4.141751	2.704322	-2.602537
168	H	0	3.563785	4.446128	1.299463
169	H	0	2.661769	3.299145	-0.697519
170	N	0	8.795766	-0.177258	3.019713
171	C	0	7.606013	-0.322926	2.179337
172	C	0	7.779617	-1.223498	0.954021
173	O	0	7.083571	-2.252409	0.778475
174	C	0	6.958491	0.984484	1.675247
175	C	0	6.315370	1.770994	2.807303
176	C	0	4.921685	1.245098	3.190446
177	O	0	4.203454	2.037099	3.887830
178	O	0	4.578808	0.069026	2.790421
179	H	0	8.592456	-0.097568	4.007046
180	H	0	9.450419	0.529583	2.696499

181	H	0	6.842489	-0.831775	2.773926
182	H	0	7.713143	1.569432	1.138343
183	H	0	6.178772	0.723851	0.948254
184	H	0	6.192969	2.825057	2.543099
185	H	0	6.955082	1.746098	3.701706
186	N	0	8.737394	-0.902938	0.087926
187	C	0	8.915441	-1.705338	-1.115459
188	C	0	10.331347	-2.212525	-1.322019
189	O	0	10.747383	-2.665518	-2.388506
190	C	0	8.403073	-0.977410	-2.381806
191	C	0	6.901158	-1.078294	-2.514912
192	C	0	6.047262	-0.092633	-2.002993
193	C	0	6.339480	-2.203600	-3.135386
194	C	0	4.659411	-0.226411	-2.118592
195	C	0	4.955520	-2.341936	-3.249913
196	C	0	4.112356	-1.347994	-2.746834
197	H	0	9.325439	-0.086480	0.235834
198	H	0	8.302743	-2.604124	-0.966813
199	H	0	8.881457	-1.430737	-3.254379
200	H	0	8.719615	0.070712	-2.329327
201	H	0	6.471114	0.779928	-1.512387
202	H	0	6.995266	-2.974475	-3.530559
203	H	0	3.994678	0.529389	-1.716323
204	H	0	4.535799	-3.220678	-3.727885
205	H	0	3.035618	-1.441172	-2.828580
206	N	0	4.720479	-3.052707	1.622681
207	C	0	3.901399	-2.787830	0.456493
208	C	0	3.890446	-3.924935	-0.543177
209	O	0	2.925198	-4.251877	-1.239925
210	H	0	5.680977	-3.221333	1.344046
211	H	0	4.736609	-2.215614	2.210559

212	H	0	4.232002	-1.906274	-0.121625
213	H	0	2.859337	-2.607803	0.734576
214	N	0	1.967201	-7.212889	1.196574
215	C	0	0.513926	-7.172860	1.388123
216	C	0	-0.303505	-7.115807	0.088419
217	O	0	-1.563853	-6.984684	0.136584
218	C	0	0.103936	-6.002787	2.275134
219	O	0	0.511077	-4.735905	1.721561
220	H	0	2.451944	-7.951474	1.685235
221	H	0	2.408825	-6.303675	1.279861
222	H	0	0.194804	-8.091331	1.894509
223	H	0	-0.975766	-6.041379	2.440107
224	H	0	0.611133	-6.105801	3.236644
225	H	0	0.028464	-4.531272	0.860441
226	N	0	0.389051	-7.089091	-1.047439
227	C	0	-0.222144	-6.921573	-2.344080
228	C	0	-0.318356	-8.233421	-3.103268
229	O	0	-0.198992	-8.323638	-4.328842
230	C	0	0.511226	-5.867514	-3.181633
231	O	0	-0.245746	-5.556385	-4.367303
232	H	0	1.399265	-7.176495	-0.940938
233	H	0	-1.252080	-6.579200	-2.182373
234	H	0	1.519231	-6.216138	-3.433761
235	H	0	0.593991	-4.953461	-2.596223
236	H	0	-0.257670	-6.338898	-4.955245
237	O	0	-0.786652	-4.283301	-0.493020
238	H	0	-0.646163	-3.462740	-1.019777
239	H	0	-1.607968	-4.762885	-0.692509
240	O	0	1.362771	0.549863	-2.199549
241	H	0	1.144211	-0.353475	-1.899677
242	H	0	0.759778	0.821122	-2.924976

243	O	0	-8.702249	0.053298	1.705828
244	H	0	-9.643515	0.222101	1.490986
245	O	0	-3.988739	11.085767	-3.134056
246	H	0	-4.413478	11.449831	-3.938550
247	O	0	-1.540252	-3.370791	6.747163
248	H	0	-1.130358	-3.306613	7.634609
249	O	0	-0.581494	-9.290476	-2.309441
250	H	0	-0.669558	-10.131381	-2.805818
251	O	0	11.013582	3.289312	-0.538876
252	H	0	11.529277	2.624915	-1.043593
253	O	0	-5.163676	1.234144	4.975900
254	H	0	-6.300048	0.254746	5.092420
255	O	0	11.095600	-2.163038	-0.202264
256	H	0	11.992007	-2.531175	-0.349484
257	O	0	5.091418	-4.555503	-0.640115
258	H	0	5.085090	-5.259516	-1.321622
259	C	0	0.616871	0.783281	1.005413
260	C	0	-0.416079	1.725776	0.531974
261	H	0	-1.047183	1.434652	-0.303586
262	C	0	0.309074	3.302044	2.262728
263	C	0	-0.279059	-1.194729	-0.043715
264	O	0	0.054113	4.324479	2.943135
265	O	0	0.050513	-1.779896	-1.107637
266	C	0	-1.646829	-1.343129	0.548927
267	H	0	-1.932855	-2.397977	0.554829
268	H	0	-1.706066	-0.931563	1.555067
269	H	0	-2.352606	-0.803341	-0.097651
270	C	0	1.471707	2.457856	2.562947
271	H	0	2.195882	2.787347	3.300222
272	C	0	-0.567025	2.916447	1.135565
273	H	0	-1.321735	3.620707	0.799682

274	C	0	1.629001	1.266927	1.956571
275	H	0	2.472623	0.618771	2.188196
276	N	0	0.645715	-0.455098	0.654845

Tyrosine Hydroxylase: Overall Charge = +1, Total energy = -11829.2219389 Ha

1	C	0	2.984319	-9.373815	2.942801
2	O	0	4.665120	-8.151136	1.636194
3	C	0	1.672534	-8.618395	2.673653
4	C	0	1.853676	-7.100920	2.557885
5	C	0	0.995875	-9.176477	1.418154
6	H	0	2.756651	-10.442293	2.967158
7	H	0	1.047989	-8.833226	3.549753
8	H	0	2.320159	-6.838110	1.599789
9	H	0	2.474125	-6.672934	3.351389
10	H	0	0.878099	-6.606932	2.608120
11	H	0	0.023357	-8.700220	1.287775
12	H	0	0.833690	-10.257863	1.492440
13	H	0	1.600789	-8.974341	0.523880
14	C	0	3.342303	-7.257384	-2.227003
15	C	0	2.031894	-7.410838	-2.960099
16	O	0	1.791897	-8.498341	-3.533371
17	H	0	4.125345	-7.211825	-3.000816
18	H	0	3.373227	-6.303022	-1.692522
19	C	0	-0.096345	-6.348200	-3.567095
20	C	0	-0.897175	-7.648715	-3.581862
21	O	0	-0.784548	-8.538994	-4.448875
22	C	0	-0.054684	-5.743429	-4.981542
23	C	0	-0.002010	-4.209980	-4.884837
24	C	0	0.283817	-3.564606	-6.242164
25	C	0	-1.333989	-3.729157	-4.291146
26	H	0	-0.654017	-5.670378	-2.921454
27	H	0	-0.951385	-6.033534	-5.544006
28	H	0	0.813075	-6.153623	-5.509570
29	H	0	0.812517	-3.941832	-4.195007

30	H	0	0.289096	-2.472916	-6.161817
31	H	0	-0.488646	-3.847240	-6.966877
32	H	0	1.254250	-3.884550	-6.634497
33	H	0	-2.122038	-3.768578	-5.051649
34	H	0	-1.273293	-2.708462	-3.904406
35	H	0	-1.621045	-4.406600	-3.474201
36	C	0	-2.882848	-8.742760	-2.481373
37	C	0	-4.194239	-8.479417	-3.279654
38	O	0	-4.396638	-7.345815	-3.796778
39	C	0	-3.222234	-8.879461	-0.986758
40	C	0	-2.155575	-9.607301	-0.157681
41	C	0	-2.508420	-9.479280	1.330220
42	C	0	-2.041870	-11.086197	-0.548242
43	H	0	-2.437044	-9.664348	-2.869327
44	H	0	-4.170520	-9.421890	-0.876408
45	H	0	-3.395869	-7.874117	-0.576118
46	H	0	-1.187037	-9.123724	-0.346990
47	H	0	-3.508987	-9.889281	1.513404
48	H	0	-2.509507	-8.432970	1.655840
49	H	0	-1.798511	-10.030682	1.953312
50	H	0	-3.000028	-11.594346	-0.386688
51	H	0	-1.286379	-11.583051	0.068062
52	H	0	-1.753874	-11.222298	-1.594635
53	C	0	-6.365630	-9.243154	-4.073660
54	O	0	-8.118566	-8.674897	-2.504341
55	C	0	-7.086769	-10.577125	-4.222163
56	H	0	-6.180640	-8.788058	-5.051272
57	H	0	-6.537626	-11.213081	-4.915772
58	H	0	-8.094941	-10.410532	-4.608682
59	C	0	-6.782245	-7.192815	1.675426
60	C	0	-6.262302	-6.133537	0.675599

61	C	0	-5.591352	-4.944957	1.318537
62	C	0	-6.181652	-3.675669	1.279756
63	C	0	-5.544750	-2.573313	1.857689
64	C	0	-4.349045	-5.092436	1.952478
65	C	0	-4.307827	-2.731399	2.486398
66	C	0	-7.833743	-6.591790	2.583923
67	O	0	-7.577971	-6.290192	3.778079
68	H	0	-5.957187	-7.504764	2.330097
69	H	0	-7.105819	-5.808455	0.056342
70	H	0	-5.552792	-6.642037	0.009801
71	H	0	-7.137655	-3.548180	0.779811
72	H	0	-6.012086	-1.595309	1.810928
73	H	0	-3.877502	-6.070928	1.983500
74	H	0	-3.811882	-1.878381	2.936965
75	C	0	-10.088787	-5.827036	2.826983
76	O	0	-11.018631	-6.486496	4.961819
77	C	0	-11.300082	-5.470815	1.952843
78	C	0	-10.980537	-4.431664	0.862082
79	C	0	-12.257696	-4.117599	0.072684
80	C	0	-10.378679	-3.150412	1.452386
81	H	0	-9.741411	-4.938119	3.361130
82	H	0	-11.691293	-6.385991	1.489328
83	H	0	-12.085040	-5.075087	2.609215
84	H	0	-10.247307	-4.868908	0.170022
85	H	0	-12.046448	-3.422236	-0.744883
86	H	0	-13.004310	-3.654686	0.728377
87	H	0	-12.694461	-5.026177	-0.353444
88	H	0	-10.229022	-2.401648	0.667800
89	H	0	-9.408234	-3.333340	1.924655
90	H	0	-11.052306	-2.723000	2.205155
91	C	0	5.412360	-4.936575	3.133930

92	O	0	6.567712	-4.022700	5.102917
93	C	0	5.563536	-4.169633	1.806865
94	C	0	4.425209	-4.463662	0.838888
95	H	0	5.458406	-6.007051	2.891699
96	H	0	6.509491	-4.492772	1.356077
97	H	0	4.621218	-3.971965	-0.119831
98	H	0	3.466267	-4.119343	1.232691
99	H	0	4.361365	-5.543347	0.660869
100	C	0	11.235943	-4.577336	1.636293
101	O	0	10.365411	-2.258300	1.673148
102	C	0	11.181903	-5.291708	0.237425
103	C	0	9.885051	-4.966778	-0.475153
104	C	0	9.735273	-3.760425	-1.180349
105	C	0	8.762197	-5.801817	-0.366495
106	C	0	8.506053	-3.367096	-1.705370
107	C	0	7.524738	-5.426642	-0.896668
108	C	0	7.386271	-4.192807	-1.539110
109	H	0	12.185969	-4.813610	2.122388
110	H	0	11.262591	-6.368491	0.417148
111	H	0	12.044663	-4.979888	-0.356642
112	H	0	10.598346	-3.114718	-1.318262
113	H	0	8.853392	-6.752882	0.150958
114	H	0	8.411679	-2.448911	-2.278090
115	H	0	6.655203	-6.067857	-0.794676
116	C	0	12.202726	0.726379	-2.413319
117	O	0	14.626952	1.125604	-2.155642
118	C	0	11.471962	2.032911	-2.834234
119	C	0	10.241746	2.337753	-1.983685
120	C	0	9.170794	1.222098	-2.007378
121	H	0	11.767402	-0.129715	-2.935328
122	H	0	12.162524	2.880727	-2.747657

123	H	0	11.193364	1.945684	-3.889521
124	H	0	9.790441	3.260584	-2.362744
125	H	0	10.575069	2.550132	-0.960903
126	H	0	9.331213	0.524891	-2.836332
127	H	0	9.178327	0.628364	-1.082383
128	C	0	4.574526	-0.169420	-5.282345
129	C	0	4.706280	0.289850	-3.814776
130	O	0	5.504088	1.202913	-3.471103
131	C	0	5.319800	-1.548892	-5.370576
132	C	0	6.800385	-1.469960	-5.821217
133	O	0	7.802527	0.592744	-5.025967
134	H	0	3.514112	-0.302862	-5.509465
135	H	0	5.281067	-2.054629	-4.397463
136	H	0	4.794656	-2.183698	-6.093114
137	H	0	6.833168	-1.025840	-6.819424
138	H	0	7.174020	-2.495418	-5.887728
139	N	0	3.772663	-0.166599	-2.974406
140	C	0	3.826086	0.196507	-1.567855
141	O	0	5.742463	-1.294440	-1.720704
142	C	0	2.573047	-0.460261	-0.968554
143	C	0	2.405836	-1.721005	-1.833933
144	C	0	2.786312	-1.235772	-3.241661
145	H	0	3.863625	1.278227	-1.417357
146	H	0	2.719708	-0.672637	0.092353
147	H	0	1.709920	0.205671	-1.077330
148	H	0	1.398733	-2.139617	-1.802348
149	H	0	3.105558	-2.496644	-1.507279
150	H	0	3.216748	-2.033322	-3.849039
151	H	0	1.917954	-0.823684	-3.769382
152	C	0	4.874028	3.903528	2.575198
153	C	0	4.005128	3.315825	3.694036

154	O	0	3.069589	3.982890	4.204666
155	C	0	4.378069	3.376896	1.212176
156	C	0	2.900905	3.462653	1.064198
157	C	0	1.914145	2.507812	1.147304
158	C	0	0.918586	4.453399	0.933657
159	N	0	0.681508	3.141863	1.056116
160	H	0	4.768659	4.985272	2.638634
161	H	0	4.694917	2.333381	1.079008
162	H	0	4.874101	3.975993	0.447220
163	H	0	2.001972	1.446941	1.308036
164	H	0	0.155865	5.213624	0.844548
165	C	0	3.459905	1.393343	5.142275
166	O	0	2.904617	2.364114	7.304615
167	C	0	3.840662	-0.099816	5.261043
168	C	0	3.995553	-0.880208	3.954328
169	O	0	1.913121	-0.044929	3.004259
170	H	0	2.398770	1.525561	4.932035
171	H	0	4.797454	-0.168916	5.789027
172	H	0	3.089376	-0.582248	5.893496
173	H	0	4.321102	-1.901639	4.163328
174	H	0	4.792026	-0.472147	3.317818
175	C	0	-0.437569	4.019608	7.172198
176	O	0	0.427681	4.147606	9.418749
177	C	0	-0.833004	2.702979	6.432927
178	C	0	-1.314887	2.983656	5.046208
179	C	0	-0.675125	2.973652	3.833537
180	C	0	-2.765637	3.488347	3.413113
181	N	0	-1.592147	3.271145	2.823597
182	H	0	-1.268302	4.716610	6.976920
183	H	0	-1.606887	2.171761	6.993704
184	H	0	0.052767	2.060953	6.390525

185	H	0	0.354342	2.745218	3.613347
186	H	0	-3.689217	3.724414	2.913328
187	C	0	-10.702916	2.887519	0.487675
188	O	0	-8.839500	4.506404	0.486078
189	C	0	-10.244263	1.397700	0.449882
190	C	0	-8.749694	1.152210	0.387578
191	C	0	-7.842256	1.829050	1.214997
192	C	0	-8.233775	0.249469	-0.554163
193	C	0	-6.465751	1.664892	1.060342
194	C	0	-6.860305	0.053455	-0.698333
195	C	0	-5.971030	0.789842	0.087479
196	H	0	-10.756767	3.232316	1.527973
197	H	0	-10.692270	0.958850	-0.449118
198	H	0	-10.691866	0.889589	1.312584
199	H	0	-8.206900	2.529804	1.957474
200	H	0	-8.917646	-0.291817	-1.200913
201	H	0	-5.771811	2.226262	1.676799
202	H	0	-6.465251	-0.631994	-1.439514
203	C	0	-8.634507	4.516096	-2.245626
204	O	0	-7.733122	6.748122	-1.964797
205	C	0	-8.709689	4.240157	-3.767991
206	C	0	-7.367409	4.379856	-4.409044
207	C	0	-6.965957	5.256943	-5.384661
208	C	0	-6.226540	3.543752	-4.104453
209	C	0	-5.165047	3.961803	-4.950407
210	C	0	-6.018275	2.477353	-3.210141
211	C	0	-3.911340	3.335065	-4.931070
212	C	0	-4.775095	1.855257	-3.183935
213	C	0	-3.734425	2.280464	-4.041865
214	H	0	-7.639035	4.223759	-1.891084
215	H	0	-9.066263	3.208643	-3.884256

216	H	0	-9.451724	4.893243	-4.237792
217	H	0	-7.523982	6.036839	-5.877650
218	H	0	-6.814486	2.133617	-2.554312
219	H	0	-3.111741	3.657596	-5.588463
220	H	0	-4.595959	1.045641	-2.484767
221	H	0	-2.775872	1.775456	-4.002149
222	C	0	-5.014380	5.870804	0.586747
223	C	0	-4.848470	7.322210	0.197410
224	O	0	-3.742198	7.909631	0.264931
225	C	0	-5.105304	4.959799	-0.655037
226	C	0	-3.741925	4.693174	-1.280117
227	O	0	-3.254008	2.853768	0.302527
228	H	0	-4.103781	5.626137	1.151185
229	H	0	-5.551600	4.010076	-0.333918
230	H	0	-5.772959	5.413937	-1.396379
231	H	0	-3.222418	5.627393	-1.511128
232	H	0	-3.866385	4.143414	-2.222001
233	C	0	-5.795245	9.394737	-0.639418
234	O	0	-6.789839	11.588993	-0.336652
235	C	0	-5.828577	9.519024	-2.192257
236	C	0	-4.629013	8.869393	-2.836576
237	C	0	-4.674234	7.542697	-3.286710
238	C	0	-3.432820	9.588919	-2.963518
239	C	0	-3.536432	6.950403	-3.844488
240	C	0	-2.298479	8.998760	-3.520165
241	C	0	-2.347137	7.673623	-3.960878
242	H	0	-4.812811	9.727753	-0.291024
243	H	0	-5.860039	10.583488	-2.442304
244	H	0	-6.753767	9.052198	-2.545467
245	H	0	-5.601298	6.979568	-3.199982
246	H	0	-3.387229	10.614180	-2.608143

247	H	0	-3.577723	5.919848	-4.178546
248	H	0	-1.378786	9.567227	-3.597712
249	H	0	-1.467500	7.209063	-4.392274
250	C	0	-0.619940	7.600777	-0.318404
251	O	0	1.009690	9.359792	0.013261
252	H	0	0.254117	6.956451	-0.437535
253	H	0	-1.316212	7.415327	-1.139137
254	C	0	4.183247	8.939673	2.068447
255	C	0	4.706585	9.468847	0.728971
256	O	0	5.660111	8.871574	0.171810
257	C	0	4.038349	7.428731	1.889719
258	H	0	4.923313	9.162741	2.840814
259	H	0	4.987931	6.972789	1.612727
260	H	0	3.641769	6.979303	2.806343
261	C	0	4.625260	11.061201	-1.035177
262	O	0	4.074718	13.352358	-0.497839
263	C	0	3.584986	10.787123	-2.128226
264	H	0	5.554779	10.547258	-1.270986
265	H	0	3.479887	9.703821	-2.247554
266	H	0	2.621705	11.203464	-1.811574
267	Fe	0	-1.225755	2.637407	0.966120
268	C	0	-0.403190	-3.112436	1.489110
269	C	0	-0.510990	-2.496536	2.818323
270	C	0	-0.713721	-2.293018	0.300378
271	C	0	-0.754466	-1.182499	2.944350
272	C	0	-0.932418	-0.972840	0.424525
273	C	0	-0.910383	-0.343648	1.750701
274	O	0	-1.032216	0.893046	1.891396
275	H	0	-0.339884	-3.145082	3.668835
276	H	0	-0.732007	-2.778252	-0.669298
277	H	0	-0.796159	-0.679359	3.901639

278	H	0	-1.134741	-0.333592	-0.427482
279	N	0	12.242952	0.447613	-0.970055
280	H	0	11.331425	0.439649	-0.526472
281	H	0	12.905395	1.033081	-0.469450
282	C	0	13.668034	0.908839	-2.892389
283	O	0	13.752343	0.876361	-4.244999
284	H	0	14.657173	1.052817	-4.577748
285	C	0	4.874501	12.546149	-0.973797
286	O	0	6.064160	12.902744	-1.507509
287	H	0	6.205501	13.872872	-1.506311
288	N	0	2.897973	9.548326	2.439979
289	H	0	2.112093	9.437606	1.808422
290	H	0	2.660443	9.507194	3.420053
291	C	0	-0.138942	9.038870	-0.356330
292	O	0	-1.061481	9.957422	-0.693394
293	H	0	-0.732820	10.878551	-0.614562
294	N	0	-1.325099	7.367444	0.950038
295	H	0	-2.316109	7.581033	0.922659
296	H	0	-0.805507	7.618799	1.781614
297	N	0	-9.006037	-6.355795	2.033394
298	H	0	-9.192261	-6.785989	1.133301
299	N	0	-7.359629	-8.292173	0.885971
300	H	0	-6.877671	-8.490936	0.017735
301	H	0	-7.569074	-9.132073	1.412725
302	C	0	-10.482101	-6.812690	3.903209
303	O	0	-10.254184	-8.107478	3.564186
304	H	0	-10.541563	-8.730809	4.263202
305	O	0	-7.105777	-11.271299	-2.957171
306	H	0	-7.705268	-10.794509	-2.346998
307	N	0	-5.071968	-9.488113	-3.400193
308	H	0	-4.969637	-10.378593	-2.924027

309	N	0	-1.909713	-7.661374	-2.706996
310	H	0	-2.082643	-6.832988	-2.155178
311	N	0	1.233166	-6.352826	-2.962437
312	H	0	1.430937	-5.593460	-2.316383
313	N	0	3.559228	-8.328959	-1.244830
314	H	0	3.620003	-9.247803	-1.669325
315	H	0	4.309927	-8.122376	-0.595171
316	C	0	-7.173221	-8.275492	-3.193161
317	O	0	-6.829881	-6.979355	-3.198343
318	H	0	-5.937824	-6.826062	-3.632544
319	O	0	-4.614068	0.630132	-0.120453
320	H	0	-4.112104	1.475447	0.048377
321	N	0	-11.981697	2.983789	-0.232148
322	H	0	-12.578954	2.174658	-0.102459
323	H	0	-12.486819	3.849136	-0.074284
324	N	0	-5.642431	5.010442	-5.716634
325	H	0	-5.114779	5.522775	-6.404596
326	N	0	-9.609398	3.759013	-1.517271
327	H	0	-10.391265	3.309718	-1.982060
328	O	0	4.072711	11.424502	-3.322481
329	H	0	3.433896	11.350953	-4.053991
330	O	0	3.131419	7.172639	0.777643
331	H	0	2.421426	7.845156	0.674892
332	N	0	4.130671	10.553875	0.224329
333	H	0	3.374485	11.005991	0.725749
334	N	0	2.248222	4.675571	0.927030
335	H	0	2.671704	5.618205	0.811239
336	N	0	6.284490	3.529254	2.752987
337	H	0	6.532522	2.567164	2.556976
338	H	0	6.761387	3.925589	3.551261
339	N	0	4.243915	2.047501	4.080382

340	H	0	5.095415	1.594398	3.781184
341	C	0	3.763689	2.045683	6.480214
342	O	0	5.090498	2.208866	6.691243
343	H	0	5.295167	2.610233	7.561490
344	C	0	2.770415	-0.926602	3.102895
345	O	0	2.697611	-2.080415	2.373136
346	N	0	-2.633746	3.331733	4.756286
347	H	0	-3.380621	3.428740	5.428821
348	N	0	0.754371	4.662870	6.601628
349	H	0	1.585976	4.076589	6.638168
350	H	0	0.586671	5.010915	5.664755
351	C	0	-0.470886	3.868426	8.625922
352	O	0	-1.674788	3.378947	9.046187
353	H	0	-1.715387	3.285419	10.019858
354	O	0	6.144302	-3.821035	-2.012977
355	H	0	5.997278	-2.823388	-1.959211
356	N	0	10.123947	-5.018492	2.491387
357	H	0	10.312943	-5.940466	2.877932
358	H	0	9.247881	-5.026945	1.972833
359	C	0	11.235329	-3.056653	1.332579
360	O	0	12.305631	-2.696587	0.570959
361	H	0	12.286692	-1.757038	0.260973
362	C	0	3.979926	-9.163233	1.815248
363	O	0	4.088580	-10.259726	1.015974
364	H	0	4.766660	-10.161857	0.315431
365	N	0	3.580357	-9.015090	4.236497
366	H	0	3.908350	-8.054232	4.275984
367	H	0	4.293058	-9.664279	4.550891
368	N	0	7.833880	1.762802	-2.232916
369	H	0	7.364195	1.483261	-3.104642
370	C	0	7.224655	2.606651	-1.400178

371	N	0	6.031943	3.124119	-1.739519
372	H	0	5.671328	3.948844	-1.293262
373	H	0	5.520481	2.666173	-2.492823
374	N	0	7.794705	2.969129	-0.232813
375	H	0	7.285548	3.482792	0.480159
376	H	0	8.716787	2.651376	0.018592
377	N	0	5.177644	0.731538	-6.275391
378	H	0	6.149034	0.931114	-6.008498
379	H	0	4.646344	1.593408	-6.376890
380	C	0	7.770273	-0.689787	-4.914354
381	O	0	8.510192	-1.360087	-4.117114
382	C	0	5.110103	-0.456743	-0.989822
383	O	0	5.365167	-0.207100	0.238150
384	C	0	6.611528	-4.614853	4.023932
385	O	0	7.750625	-5.067676	3.464271
386	H	0	8.610279	-4.735327	3.820789
387	N	0	4.165015	-4.601617	3.835061
388	H	0	3.668779	-3.823119	3.408318
389	H	0	4.292891	-4.469559	4.832650
390	O	0	5.655656	-2.764273	2.153848
391	H	0	5.520682	-2.160756	1.388558
392	C	0	-2.854609	3.894998	-0.382388
393	N	0	-6.198709	5.613156	1.418693
394	H	0	-7.034689	5.290352	0.937026
395	H	0	-6.378200	6.280982	2.156991
396	N	0	-5.930205	7.999038	-0.230667
397	H	0	-6.807423	7.522601	-0.404285
398	C	0	-6.812502	10.388073	-0.074584
399	O	0	-7.750290	9.817766	0.721696
400	H	0	-8.397398	10.467586	1.068272
401	O	0	-1.617063	4.209004	-0.243354

402	C	0	-9.633743	3.792798	-0.174427
403	C	0	-8.714394	5.999025	-1.974177
404	O	0	-9.977979	6.446853	-1.816998
405	H	0	-10.027862	7.414802	-1.670175
406	C	0	-3.713030	-3.996001	2.535377
407	H	0	-2.757368	-4.131364	3.030917
408	H	0	1.951971	-2.084108	1.738662
409	N	0	-0.003224	-4.335996	1.425402
410	C	0	-0.679586	-6.215386	0.058967
411	H	0	-0.849506	-6.733716	1.004486
412	H	0	-1.651973	-5.828596	-0.272811
413	H	0	-0.263041	-6.904638	-0.677131
414	C	0	0.244892	-5.056904	0.256811
415	O	0	1.183612	-4.762609	-0.503508

Tyrosinase: Overall Charge = +2, Total energy = -8951.75842002 Ha

1	C	0	6.224640	3.131245	5.883652
2	O	0	4.346947	2.350509	7.146579
3	C	0	6.433676	2.378737	4.546720
4	C	0	5.151639	2.046036	3.851017
5	N	0	4.546064	0.792712	3.865626
6	C	0	4.309985	2.841918	3.110244
7	C	0	3.398576	0.846192	3.154259
8	N	0	3.222784	2.085865	2.679977
9	H	0	7.024105	1.474281	4.727256
10	H	0	7.031734	3.038572	3.913669
11	H	0	4.884620	-0.046217	4.321404
12	H	0	4.412734	3.883166	2.855283
13	H	0	2.739037	0.009269	3.013742
14	H	0	5.539827	3.963821	5.703913

15	C	0	-3.586096	4.708284	5.045997
16	O	0	-4.980690	5.344731	6.842279
17	C	0	-3.250280	3.195693	5.027307
18	C	0	-1.887914	2.988349	4.462454
19	N	0	-0.784041	2.782870	5.272342
20	C	0	-1.395514	3.047524	3.181127
21	C	0	0.326507	2.719275	4.508148
22	N	0	-0.012941	2.876792	3.223161
23	H	0	-3.657663	5.038447	4.005615
24	H	0	-3.290288	2.804616	6.049210
25	H	0	-4.002488	2.660840	4.440366
26	H	0	-0.804703	2.728625	6.280800
27	H	0	-1.921196	3.222157	2.258172
28	H	0	1.321491	2.569919	4.891946
29	C	0	2.602296	8.968558	-0.243461
30	O	0	4.368755	9.586344	-1.704188
31	C	0	2.543802	7.536247	-0.828980
32	C	0	2.129968	6.486073	0.160528
33	N	0	1.256089	6.735262	1.210177
34	C	0	2.399163	5.140120	0.225721
35	C	0	1.011372	5.587537	1.874801
36	N	0	1.691156	4.585626	1.297623
37	H	0	3.263822	8.959727	0.630717
38	H	0	1.842239	7.532773	-1.673884
39	H	0	3.525442	7.279600	-1.232748
40	H	0	0.876778	7.662769	1.385242
41	H	0	2.995618	4.543914	-0.442589
42	H	0	0.365513	5.500373	2.730829
43	C	0	-11.981154	0.665797	-0.081096
44	O	0	-14.327618	0.200075	0.158271
45	C	0	-11.773247	1.248788	1.328518

46	C	0	-10.356361	1.050180	1.818866
47	C	0	-9.914541	-0.219615	2.217193
48	C	0	-9.460889	2.124967	1.884742
49	C	0	-8.609261	-0.410594	2.672712
50	C	0	-8.153348	1.939839	2.346916
51	C	0	-7.724067	0.669536	2.741427
52	H	0	-11.782692	-0.412062	-0.045162
53	H	0	-12.485408	0.763447	2.003001
54	H	0	-12.015809	2.318031	1.308553
55	H	0	-10.600509	-1.060854	2.176827
56	H	0	-9.792374	3.114692	1.586076
57	H	0	-8.283515	-1.398576	2.977381
58	H	0	-7.478107	2.786036	2.409623
59	H	0	-6.711400	0.523074	3.099222
60	C	0	-4.668308	1.060943	-5.167745
61	C	0	-4.498600	-0.223549	-4.354675
62	O	0	-3.482500	-0.892909	-4.478692
63	C	0	-4.625458	2.298678	-4.229675
64	C	0	-3.698581	2.218975	-3.061649
65	N	0	-4.081029	1.616889	-1.873864
66	C	0	-2.432651	2.708430	-2.817554
67	C	0	-3.075786	1.762491	-0.972681
68	N	0	-2.049135	2.420444	-1.505792
69	H	0	-3.776241	1.077792	-5.807539
70	H	0	-4.371650	3.172948	-4.835898
71	H	0	-5.647428	2.449920	-3.861034
72	H	0	-4.922284	1.087674	-1.681377
73	H	0	-1.786358	3.253733	-3.485260
74	H	0	-3.164910	1.397929	0.032881
75	N	0	-5.485886	-0.553678	-3.524536
76	C	0	-5.452263	-1.794141	-2.741480

77	O	0	-4.537719	-3.932863	-3.397210
78	C	0	-6.693083	-1.801062	-1.828513
79	C	0	-6.624325	-0.619968	-0.861607
80	O	0	-5.516498	-0.295472	-0.349555
81	H	0	-6.378634	-0.088467	-3.663789
82	H	0	-4.545840	-1.796612	-2.129492
83	H	0	-6.713609	-2.722835	-1.237613
84	H	0	-7.606381	-1.776096	-2.430341
85	C	0	-1.153056	-4.453773	-3.875027
86	O	0	-0.562794	-6.747406	-3.855896
87	C	0	-1.261573	-3.793998	-2.458734
88	C	0	-0.402311	-2.575665	-2.345256
89	N	0	0.980324	-2.672852	-2.239086
90	C	0	-0.668031	-1.217507	-2.371153
91	C	0	1.495589	-1.415337	-2.190952
92	N	0	0.523203	-0.500065	-2.267100
93	H	0	-0.108194	-4.411924	-4.192531
94	H	0	-0.963091	-4.511629	-1.683716
95	H	0	-2.311032	-3.527957	-2.299851
96	H	0	1.465649	-3.557286	-2.064729
97	H	0	-1.622412	-0.727563	-2.479419
98	H	0	2.548632	-1.201427	-2.106646
99	C	0	3.486030	-6.055203	-1.821497
100	C	0	2.071853	-6.212216	-1.295166
101	O	0	1.301266	-5.252993	-1.247844
102	C	0	3.494467	-5.944102	-3.354633
103	C	0	4.647456	-5.060265	-3.827528
104	S	0	4.333075	-3.236850	-3.475303
105	C	0	3.179209	-2.862360	-4.901010
106	H	0	3.803474	-5.074864	-1.433946
107	H	0	2.539065	-5.538787	-3.706988

108	H	0	3.585516	-6.948987	-3.786418
109	H	0	5.570843	-5.299577	-3.294501
110	H	0	4.824701	-5.156450	-4.899989
111	H	0	3.732262	-2.903851	-5.839155
112	H	0	2.785338	-1.858310	-4.742306
113	H	0	2.350199	-3.570640	-4.914785
114	N	0	1.739844	-7.427256	-0.892987
115	C	0	0.423890	-7.738099	-0.389109
116	C	0	0.183512	-7.322461	1.057436
117	O	0	-0.966236	-7.347080	1.499401
118	H	0	2.479552	-8.123750	-0.934521
119	H	0	0.252463	-8.813684	-0.463163
120	H	0	-0.334628	-7.231447	-0.991325
121	N	0	1.215024	-6.961724	1.793567
122	C	0	1.014301	-6.528914	3.163191
123	C	0	1.625664	-5.176978	3.507749
124	O	0	2.751104	-4.893989	3.172653
125	C	0	1.528335	-7.593852	4.178598
126	C	0	0.700749	-8.877105	4.049620
127	C	0	3.022871	-7.891715	4.007519
128	H	0	2.148574	-6.934323	1.404317
129	H	0	-0.070014	-6.462831	3.287371
130	H	0	1.367751	-7.167473	5.178971
131	H	0	0.835177	-9.316096	3.054617
132	H	0	-0.365380	-8.682630	4.196794
133	H	0	1.026044	-9.614087	4.789638
134	H	0	3.632494	-6.990193	4.102246
135	H	0	3.214668	-8.341041	3.026234
136	H	0	3.345626	-8.610332	4.766581
137	N	0	0.849462	-4.369406	4.214950
138	C	0	1.280612	-3.030031	4.611633

139	O	0	3.554605	-2.279824	4.572788
140	C	0	0.195055	-2.350550	5.469855
141	C	0	0.713462	-1.010556	6.005134
142	C	0	-1.081010	-2.133008	4.644028
143	H	0	-0.049301	-4.684528	4.552640
144	H	0	1.382205	-2.429444	3.696078
145	H	0	-0.025950	-3.010441	6.319503
146	H	0	1.017213	-0.359979	5.176230
147	H	0	1.571446	-1.140015	6.672333
148	H	0	-0.073705	-0.497785	6.563558
149	H	0	-0.871934	-1.448116	3.812327
150	H	0	-1.858232	-1.679990	5.264622
151	H	0	-1.486640	-3.057992	4.223438
152	C	0	5.074562	-3.790272	0.925053
153	O	0	6.579046	-3.506997	-0.909477
154	C	0	3.868408	-2.861549	0.805398
155	H	0	4.889676	-4.676558	0.299874
156	H	0	3.631206	-2.664330	-0.246762
157	H	0	4.077885	-1.921662	1.326364
158	H	0	3.010489	-3.350193	1.277097
159	C	0	5.926353	-0.244973	-4.687045
160	O	0	4.931288	1.844419	-5.265258
161	C	0	6.580138	-0.312362	-3.283214
162	C	0	5.873823	0.546886	-2.264986
163	C	0	6.121593	1.924430	-2.192247
164	C	0	4.938124	-0.022550	-1.390900
165	C	0	5.441102	2.719577	-1.269437
166	C	0	4.252605	0.770024	-0.467549
167	C	0	4.507292	2.141907	-0.406598
168	H	0	4.870004	-0.525482	-4.605564
169	H	0	6.561284	-1.360078	-2.964426

170	H	0	7.630048	-0.012079	-3.384693
171	H	0	6.857539	2.372102	-2.854821
172	H	0	4.766719	-1.095968	-1.439812
173	H	0	5.644376	3.783612	-1.217776
174	H	0	3.521528	0.330827	0.204628
175	H	0	3.993718	2.751542	0.326772
176	C	0	2.572970	4.731349	-6.090353
177	O	0	1.785725	6.882145	-6.826359
178	C	0	3.183793	4.903789	-4.672233
179	C	0	2.432265	4.008783	-3.735245
180	N	0	1.394063	4.531134	-2.957813
181	C	0	2.462380	2.634137	-3.589134
182	C	0	0.821629	3.486306	-2.358807
183	N	0	1.433860	2.317623	-2.719644
184	H	0	1.490367	4.625003	-5.955451
185	H	0	3.069346	5.935585	-4.331950
186	H	0	4.250896	4.669448	-4.719190
187	H	0	3.123545	1.897497	-4.014666
188	H	0	-0.029651	3.507466	-1.697494
189	Cu	0	1.474724	2.700466	1.877717
190	C	0	-1.374119	-2.392250	0.772936
191	C	0	-1.938801	-1.032280	0.911890
192	C	0	0.884516	-1.451944	0.828130
193	C	0	0.325893	-0.131091	1.071014
194	O	0	1.114818	0.840591	1.264244
195	H	0	-3.018473	-0.919683	0.894362
196	H	0	1.960120	-1.515087	0.774097
197	H	0	1.122390	1.369555	-2.460892
198	N	0	1.290628	9.411587	0.187915
199	H	0	1.208405	10.094531	0.926201
200	H	0	0.499151	9.308123	-0.432432

201	C	0	3.286997	9.903499	-1.255020
202	O	0	2.668185	11.057589	-1.584624
203	H	0	1.803450	11.149399	-1.129896
204	C	0	2.655368	6.022746	-6.921492
205	O	0	3.900660	6.645974	-6.597405
206	H	0	3.835891	7.607478	-6.774166
207	N	0	2.999058	3.515087	-6.797274
208	H	0	2.604604	2.660286	-6.425929
209	H	0	4.011446	3.412501	-6.806470
210	N	0	6.714587	-1.091201	-5.595194
211	H	0	7.051192	-1.942830	-5.158735
212	H	0	6.267955	-1.282440	-6.486044
213	C	0	5.963934	1.177928	-5.190162
214	O	0	7.167988	1.630100	-5.601251
215	H	0	7.786590	0.864542	-5.693660
216	C	0	2.711181	-2.951400	5.155943
217	O	0	3.138395	-4.312913	5.245707
218	H	0	4.113405	-4.351507	5.148955
219	N	0	4.376010	-7.124527	-1.346631
220	H	0	4.948026	-7.565875	-2.054687
221	H	0	4.881164	-6.933400	-0.491102
222	N	0	7.481001	3.663715	6.369543
223	H	0	8.160020	2.954238	6.624679
224	H	0	7.386233	4.373335	7.085887
225	C	0	5.506658	2.226299	6.874168
226	O	0	6.320809	1.256706	7.381051
227	H	0	5.869286	0.665177	8.018122
228	C	0	-4.924727	4.916541	5.719320
229	O	0	-6.282757	4.748510	5.304841
230	H	0	-6.817398	5.004201	6.083625
231	N	0	-2.530175	5.493063	5.701621

232	H	0	-2.390390	6.407832	5.287892
233	H	0	-2.616880	5.525979	6.711524
234	N	0	5.263020	-4.142804	2.340078
235	H	0	4.377247	-4.297168	2.805166
236	H	0	5.925255	-4.895671	2.488469
237	C	0	6.265082	-3.132267	0.205696
238	O	0	6.841406	-2.093423	0.855012
239	H	0	7.547973	-1.663295	0.328261
240	N	0	-11.065960	1.291902	-1.045936
241	H	0	-11.312335	1.120605	-2.014800
242	H	0	-10.933404	2.285572	-0.882855
243	C	0	-13.457045	0.786042	-0.487840
244	O	0	-13.704064	1.544255	-1.579433
245	H	0	-14.656958	1.579988	-1.806120
246	N	0	-5.928415	1.140382	-5.922434
247	H	0	-6.169999	2.062391	-6.257587
248	H	0	-6.012971	0.449520	-6.659934
249	C	0	-5.360010	-3.039865	-3.642126
250	O	0	-6.227685	-3.043077	-4.667669
251	H	0	-6.163724	-3.837949	-5.238441
252	N	0	-7.753287	0.059635	-0.601437
253	H	0	-7.741053	0.806848	0.085286
254	H	0	-8.652669	-0.146337	-1.018179
255	N	0	-1.991859	-3.825196	-4.890005
256	H	0	-1.808043	-2.829820	-4.991821
257	H	0	-2.977201	-3.980788	-4.689891
258	C	0	-1.465649	-5.937285	-3.800582
259	O	0	-2.771498	-6.252010	-3.652056
260	H	0	-2.925761	-7.219050	-3.604600
261	N	0	-2.061939	-3.485957	0.761039
262	C	0	-3.452871	-3.584732	0.992124

263	C	0	-4.161043	-4.500319	0.045922
264	H	0	-4.073139	-4.150135	-0.988317
265	H	0	-3.697683	-5.490529	0.089654
266	H	0	-5.212003	-4.569387	0.323361
267	O	0	-3.979899	-3.038051	1.971382
268	C	0	-1.124597	0.028605	1.087994
269	H	0	-1.497036	1.032971	1.236491
270	C	0	0.085204	-2.525748	0.680413
271	H	0	0.471570	-3.525239	0.497658

DOPA decarboxylase: Overall Charge = -1, Total energy = -7657.88069644 Ha

1	N	0	7.967714	0.344639	7.956355
2	C	0	7.149063	-0.559074	7.165214
3	C	0	7.706335	-1.971380	7.115476
4	O	0	6.982825	-2.930163	6.854369
5	C	0	7.119175	-0.073466	5.685015
6	C	0	6.158963	1.039345	5.401341
7	C	0	6.311574	2.384901	5.641130
8	C	0	4.883671	0.882376	4.733883
9	N	0	5.201872	3.073243	5.173759
10	C	0	4.316213	2.176841	4.599595
11	C	0	4.191220	-0.223367	4.205644
12	C	0	3.106672	2.398018	3.926806
13	C	0	2.987951	-0.015204	3.541651
14	C	0	2.458100	1.288505	3.395909
15	H	0	8.078614	0.071194	8.926713
16	H	0	7.665075	1.310430	7.880603
17	H	0	6.112113	-0.672351	7.513392
18	H	0	6.851374	-0.924765	5.049511
19	H	0	8.146574	0.211645	5.434245

20	H	0	7.140046	2.918430	6.080064
21	H	0	5.077655	4.072206	5.218591
22	H	0	4.590754	-1.226300	4.325438
23	H	0	2.722390	3.404885	3.795095
24	H	0	2.441624	-0.859010	3.130358
25	H	0	1.530435	1.423129	2.847950
26	N	0	-5.474377	-2.896831	4.916442
27	C	0	-4.143644	-2.571744	4.456290
28	C	0	-3.265271	-3.780506	4.104964
29	O	0	-3.595013	-4.960676	4.348409
30	C	0	-3.449904	-1.557393	5.407736
31	C	0	-3.703500	-0.156383	4.905449
32	C	0	-2.750908	0.484642	4.103569
33	C	0	-4.948171	0.463662	5.083995
34	C	0	-3.038893	1.689285	3.462242
35	C	0	-5.247127	1.668784	4.449556
36	C	0	-4.301233	2.271967	3.616434
37	O	0	-4.649991	3.444177	2.975978
38	H	0	-5.484278	-3.402568	5.798118
39	H	0	-6.069418	-3.359349	4.235995
40	H	0	-4.267282	-2.058934	3.491440
41	H	0	-3.852137	-1.706126	6.415121
42	H	0	-2.372105	-1.746845	5.455555
43	H	0	-1.768721	0.036644	3.972258
44	H	0	-5.696862	-0.022933	5.699409
45	H	0	-2.286634	2.183754	2.858261
46	H	0	-6.214327	2.142385	4.570352
47	H	0	-4.044834	3.625993	2.214289
48	N	0	-2.135297	-3.445829	3.427711
49	C	0	-1.262596	-4.391683	2.760525
50	C	0	0.172912	-3.928020	2.947344

51	O	0	0.419083	-2.697607	3.071839
52	C	0	-1.558242	-4.404452	1.233663
53	C	0	-2.982123	-4.803594	0.938077
54	C	0	-3.939339	-3.847271	0.578525
55	C	0	-3.372840	-6.145817	1.052569
56	C	0	-5.262716	-4.230074	0.337127
57	C	0	-4.692003	-6.528187	0.814810
58	C	0	-5.642617	-5.568062	0.455230
59	H	0	-1.889955	-2.471176	3.296438
60	H	0	-1.435010	-5.380479	3.194025
61	H	0	-1.358778	-3.391691	0.862544
62	H	0	-0.853197	-5.077248	0.731639
63	H	0	-3.653487	-2.804849	0.468422
64	H	0	-2.635889	-6.894395	1.329672
65	H	0	-5.986159	-3.477401	0.043882
66	H	0	-4.978076	-7.569874	0.903498
67	H	0	-6.665789	-5.867261	0.255498
68	N	0	1.149995	-4.851462	2.912304
69	C	0	2.539627	-4.413514	2.959802
70	C	0	2.929885	-3.768759	1.635012
71	O	0	2.291156	-3.977051	0.581671
72	C	0	3.323866	-5.733348	3.127792
73	C	0	2.462706	-6.730465	2.333989
74	C	0	1.020679	-6.306885	2.655581
75	H	0	2.693112	-3.710966	3.783490
76	H	0	3.358468	-6.002037	4.187120
77	H	0	4.347064	-5.654020	2.753551
78	H	0	2.656628	-6.618508	1.263654
79	H	0	2.652225	-7.767096	2.615081
80	H	0	0.640818	-6.811912	3.549381
81	H	0	0.341274	-6.501504	1.823612

82	N	0	4.079040	-3.050164	1.693786
83	C	0	4.863076	-2.739162	0.514151
84	C	0	6.054482	-3.660712	0.499366
85	O	0	6.606994	-4.217285	1.446965
86	C	0	5.254812	-1.227153	0.488179
87	O	0	5.503834	-0.795631	-0.849094
88	C	0	6.471542	-0.902839	1.337037
89	H	0	4.545448	-2.963970	2.589090
90	H	0	4.262008	-2.908165	-0.383137
91	H	0	4.365683	-0.718642	0.890134
92	H	0	4.619849	-0.809941	-1.323513
93	H	0	6.365548	-1.285485	2.357397
94	H	0	6.607448	0.180921	1.388650
95	H	0	7.368221	-1.342338	0.887790
96	N	0	-10.843834	0.390025	-3.029079
97	C	0	-10.203841	1.508366	-2.375870
98	C	0	-10.668368	2.786525	-3.041811
99	O	0	-11.261202	2.978114	-4.103701
100	C	0	-8.639466	1.356125	-2.359542
101	C	0	-7.740914	2.208227	-1.507195
102	N	0	-7.680602	3.598875	-1.530272
103	C	0	-6.672718	1.812604	-0.738139
104	C	0	-6.623768	4.033982	-0.832017
105	N	0	-5.991672	2.958257	-0.349372
106	H	0	-10.906565	-0.455557	-2.479059
107	H	0	-10.577866	0.230988	-3.992999
108	H	0	-10.554141	1.561117	-1.340679
109	H	0	-8.289886	1.409436	-3.399687
110	H	0	-8.475951	0.325836	-2.025749
111	H	0	-8.410587	4.169931	-1.947279
112	H	0	-6.338153	0.828113	-0.452537

113	H	0	-6.339480	5.059421	-0.683970
114	N	0	-9.317208	-3.785227	0.737876
115	C	0	-8.498281	-3.264973	1.817350
116	C	0	-9.043753	-3.540363	3.202808
117	O	0	-8.297931	-3.900832	4.114254
118	C	0	-8.228911	-1.756280	1.546141
119	O	0	-8.040507	-1.691248	0.102812
120	C	0	-6.990013	-1.240867	2.249505
121	H	0	-9.134259	-4.748396	0.483844
122	H	0	-10.313607	-3.610622	0.842024
123	H	0	-7.533108	-3.779653	1.797065
124	H	0	-9.116028	-1.167715	1.803321
125	H	0	-8.611852	-2.441668	-0.247795
126	H	0	-7.059341	-1.390067	3.331988
127	H	0	-6.849925	-0.174244	2.059926
128	H	0	-6.107010	-1.779560	1.885915
129	N	0	4.512710	-4.349875	-6.131715
130	C	0	5.124006	-3.554736	-5.077404
131	C	0	4.422383	-3.642743	-3.736927
132	O	0	4.771274	-2.952102	-2.779435
133	C	0	5.160186	-2.037301	-5.415412
134	C	0	3.767382	-1.537566	-5.622914
135	N	0	3.155079	-1.458003	-6.870459
136	C	0	2.768011	-1.291021	-4.707782
137	C	0	1.833915	-1.176524	-6.687654
138	N	0	1.568486	-1.084423	-5.387357
139	H	0	5.153778	-4.846919	-6.732998
140	H	0	3.755839	-3.905030	-6.637308
141	H	0	6.150358	-3.902780	-4.922846
142	H	0	5.786414	-1.868959	-6.296398
143	H	0	5.605688	-1.530747	-4.557713

144	H	0	3.617436	-1.551622	-7.763162
145	H	0	2.837664	-1.259698	-3.624188
146	H	0	1.130288	-1.059768	-7.492387
147	N	0	3.426901	-4.509545	-3.661169
148	C	0	2.798314	-4.749935	-2.382564
149	C	0	3.307891	-6.091908	-1.884159
150	O	0	4.141841	-6.161418	-0.987424
151	C	0	1.269555	-4.650722	-2.409930
152	C	0	0.841019	-3.245439	-2.849513
153	C	0	-0.628866	-2.941576	-2.548454
154	C	0	-1.050464	-1.529799	-2.947025
155	N	0	-0.953358	-1.301700	-4.430394
156	H	0	3.207744	-5.058096	-4.486154
157	H	0	3.181524	-4.017776	-1.675342
158	H	0	0.916358	-4.847107	-1.390594
159	H	0	0.844495	-5.417936	-3.068722
160	H	0	1.475516	-2.505178	-2.339568
161	H	0	1.035704	-3.137333	-3.922878
162	H	0	-0.806467	-3.038939	-1.470410
163	H	0	-1.283204	-3.673668	-3.042408
164	H	0	-2.084895	-1.340692	-2.651952
165	H	0	-0.411969	-0.784166	-2.460818
166	H	0	0.080238	-1.153723	-4.776134
167	H	0	-1.490084	-0.445604	-4.658785
168	H	0	-1.356781	-2.090244	-4.943026
169	N	0	8.479404	-0.636903	-4.329930
170	C	0	7.570180	0.342163	-4.853441
171	C	0	8.121227	1.166373	-5.948944
172	O	0	9.250895	1.667172	-5.912662
173	C	0	7.098974	1.287741	-3.716532
174	C	0	5.936200	2.137112	-4.166445

175	C	0	6.130620	3.423311	-4.681891
176	C	0	4.636971	1.610265	-4.122023
177	C	0	5.046217	4.170151	-5.151224
178	C	0	3.554449	2.343760	-4.610485
179	C	0	3.758931	3.629592	-5.122801
180	H	0	8.274672	-0.936012	-3.386081
181	H	0	8.719840	-1.401337	-4.947391
182	H	0	6.650389	-0.094142	-5.261781
183	H	0	7.948407	1.894643	-3.385827
184	H	0	6.790176	0.644427	-2.883564
185	H	0	7.133347	3.840566	-4.714490
186	H	0	4.469099	0.628995	-3.682195
187	H	0	5.208052	5.168260	-5.542322
188	H	0	2.555679	1.915748	-4.595770
189	H	0	2.919113	4.204559	-5.496492
190	N	0	5.806273	4.049117	2.118312
191	C	0	4.887706	4.570197	1.102569
192	C	0	4.220566	5.754476	1.818878
193	O	0	3.337237	5.556527	2.654889
194	C	0	3.789405	3.609384	0.605170
195	C	0	4.426015	2.273977	0.185332
196	C	0	2.995077	4.283130	-0.520431
197	C	0	5.593607	2.425093	-0.793299
198	H	0	5.354510	3.456771	2.807277
199	H	0	6.648642	3.624769	1.748642
200	H	0	5.471577	4.951357	0.261607
201	H	0	3.111472	3.416152	1.445483
202	H	0	4.771883	1.751701	1.088997
203	H	0	3.658366	1.631780	-0.261691
204	H	0	3.632027	4.502612	-1.384882
205	H	0	2.552787	5.230505	-0.182961

206	H	0	2.180221	3.637113	-0.853180
207	H	0	6.447392	2.938801	-0.337415
208	H	0	5.301716	2.988461	-1.685049
209	H	0	5.923162	1.431912	-1.101963
210	N	0	1.775288	7.782602	1.721610
211	C	0	0.440385	8.180296	1.295748
212	C	0	-0.280972	9.001732	2.359993
213	O	0	-1.343715	9.548070	2.113295
214	C	0	-0.489774	7.005098	0.901276
215	C	0	-0.822586	6.107483	2.069156
216	C	0	-2.017409	6.297912	2.778647
217	C	0	0.049682	5.085401	2.476623
218	C	0	-2.334663	5.493127	3.874956
219	C	0	-0.266601	4.280451	3.573586
220	C	0	-1.456394	4.485269	4.277595
221	H	0	1.788035	7.149396	2.517456
222	H	0	2.299981	7.392263	0.943730
223	H	0	0.535872	8.846353	0.432936
224	H	0	-1.407127	7.417746	0.469513
225	H	0	0.027978	6.449004	0.112752
226	H	0	-2.701655	7.079880	2.462909
227	H	0	0.977050	4.914565	1.934527
228	H	0	-3.269337	5.641680	4.402233
229	H	0	0.409027	3.488453	3.878407
230	H	0	-1.704606	3.851820	5.121269
231	N	0	-6.485584	-1.190108	-4.474709
232	C	0	-6.535528	-1.530697	-3.177949
233	C	0	-7.534264	-2.579030	-2.782665
234	C	0	-5.674489	-0.926657	-2.231371
235	O	0	-5.772211	-1.208272	-0.901347
236	C	0	-4.732542	0.023899	-2.665674

237	C	0	-4.734503	0.394438	-4.025874
238	C	0	-5.618284	-0.246337	-4.886670
239	C	0	-3.839079	1.463937	-4.570029
240	O	0	-2.443697	1.089307	-4.366227
241	15	0	-1.322925	2.417139	-4.557471
242	O	0	-1.369431	2.929020	-6.060717
243	O	0	-1.579656	3.409468	-3.333925
244	O	0	0.069103	1.464011	-4.359021
245	H	0	-8.469106	-2.125692	-2.430343
246	H	0	-7.147911	-3.236185	-1.997985
247	H	0	-7.775083	-3.181544	-3.658510
248	H	0	-6.699508	-1.468151	-0.547930
249	H	0	-5.632089	-0.002135	-5.942059
250	H	0	-4.022539	1.600211	-5.638531
251	H	0	-4.017477	2.420617	-4.062692
252	C	0	-0.412238	1.289132	-0.156548
253	C	0	0.366819	1.802773	-1.196859
254	C	0	-0.034167	0.055705	0.411464
255	C	0	1.077728	-0.642766	-0.035715
256	C	0	1.908066	-0.138449	-1.083537
257	O	0	2.960732	-0.797134	-1.525557
258	H	0	-5.089368	2.985957	0.155662
259	H	0	0.086681	2.722047	-1.698998
260	H	0	-0.636038	-0.363040	1.215233
261	H	0	1.334239	-1.598062	0.413513
262	H	0	0.357040	1.237521	-3.445825
263	O	0	9.041418	-2.083147	7.313462
264	H	0	9.346966	-3.011120	7.230417
265	O	0	6.605356	-3.788192	-0.814144
266	H	0	7.408115	-4.351040	-0.765481
267	O	0	7.337872	1.415638	-7.119180

268	H	0	7.842891	2.019987	-7.702016
269	O	0	2.814883	-7.303234	-2.462303
270	H	0	3.271563	-8.062964	-2.043950
271	O	0	-10.440163	-3.382653	3.466043
272	H	0	-10.619582	-3.608338	4.403057
273	O	0	-10.278193	3.905861	-2.243435
274	H	0	-10.643616	4.737946	-2.613154
275	O	0	0.303157	9.127155	3.659046
276	H	0	-0.295854	9.658994	4.224599
277	O	0	4.660616	6.989338	1.486823
278	H	0	4.186549	7.703577	1.974287
279	C	0	-3.771059	0.657532	-1.731095
280	H	0	-3.429741	1.673404	-1.984899
281	O	0	-3.302151	0.090940	-0.727301
282	C	0	-2.197620	3.068389	0.206082
283	C	0	-1.485407	4.202569	-0.442492
284	H	0	-0.434716	4.234639	-0.143851
285	H	0	-1.536347	4.085368	-1.533399
286	H	0	-1.983588	5.124427	-0.149050
287	H	0	-2.271261	1.165751	0.692678
288	C	0	1.504343	1.110831	-1.634636
289	H	0	2.149673	1.556494	-2.388567
290	N	0	-1.626141	1.863423	0.330705
291	O	0	-3.401129	3.218584	0.639302

Monoamine Oxidase: Overall Charge = -1, Total energy = -12612.2966770 Ha

1	N	0	5.169485	0.913997	7.462559
2	C	0	4.056769	0.187744	8.030346
3	C	0	4.358411	-1.305608	8.013833

4	O	0	4.771880	-1.915525	7.020677
5	C	0	2.748299	0.395401	7.220539
6	C	0	2.313873	1.844911	7.135824
7	C	0	1.938165	2.553553	8.280416
8	C	0	2.360222	2.522972	5.926107
9	C	0	1.596793	3.891719	8.214321
10	C	0	2.013218	3.864040	5.852091
11	C	0	1.635817	4.536301	6.998906
12	O	0	1.312689	5.863061	6.936891
13	H	0	5.019073	1.304700	6.539956
14	H	0	5.706728	1.484761	8.096540
15	H	0	3.884536	0.475256	9.071779
16	H	0	1.977644	-0.225613	7.688920
17	H	0	2.912879	0.006749	6.207759
18	H	0	1.903276	2.041288	9.238091
19	H	0	2.703460	2.010452	5.036556
20	H	0	1.304348	4.427702	9.111397
21	H	0	2.037755	4.390375	4.904919
22	H	0	1.060002	6.233084	7.801003
23	C	0	-12.280612	-1.746695	5.383699
24	C	0	-12.845493	-1.343131	4.017014
25	O	0	-12.104400	-0.817091	3.186841
26	C	0	-11.604412	-3.119124	5.285219
27	C	0	-12.646506	-4.083716	5.736639
28	C	0	-13.366265	-3.365191	6.841876
29	H	0	-11.576209	-0.960234	5.666886
30	H	0	-11.247298	-3.322705	4.278987
31	H	0	-10.736030	-3.139185	5.953599
32	H	0	-12.231026	-5.040178	6.062807
33	H	0	-13.343602	-4.296025	4.914503
34	H	0	-12.833656	-3.482961	7.796185

35	H	0	-14.391343	-3.716343	6.983628
36	N	0	-14.141651	-1.578406	3.802654
37	C	0	-14.772113	-1.410214	2.485904
38	C	0	-15.589501	-0.135147	2.374226
39	O	0	-16.513680	0.073569	3.165219
40	C	0	-15.692420	-2.600091	2.165548
41	C	0	-15.041928	-3.952182	2.330710
42	C	0	-13.870951	-4.274063	1.642186
43	C	0	-15.614839	-4.908143	3.171185
44	C	0	-13.270806	-5.531240	1.798691
45	C	0	-15.031589	-6.169649	3.326466
46	C	0	-13.854981	-6.481396	2.639517
47	H	0	-14.678760	-1.900781	4.602481
48	H	0	-13.948493	-1.401872	1.772288
49	H	0	-16.051901	-2.470113	1.135822
50	H	0	-16.572805	-2.533578	2.815319
51	H	0	-13.414793	-3.544232	0.979158
52	H	0	-16.525383	-4.665078	3.710244
53	H	0	-12.358815	-5.764262	1.260802
54	H	0	-15.490848	-6.900837	3.981794
55	H	0	-13.396548	-7.456251	2.760174
56	N	0	-15.260068	0.726614	1.388644
57	C	0	-16.083042	1.912945	1.137971
58	C	0	-17.471770	1.471741	0.684768
59	O	0	-17.574539	0.639223	-0.206790
60	C	0	-15.347924	2.620775	-0.013233
61	C	0	-13.966534	2.049216	-0.006537
62	C	0	-14.135463	0.633297	0.444047
63	H	0	-16.159962	2.523238	2.040871
64	H	0	-15.369282	3.704274	0.108204
65	H	0	-15.848891	2.381075	-0.956892

66	H	0	-13.479694	2.122595	-0.981236
67	H	0	-13.337659	2.586123	0.712021
68	H	0	-13.246821	0.249808	0.948479
69	H	0	-14.393806	-0.034635	-0.387318
70	N	0	-13.493371	-7.316000	-6.407900
71	C	0	-12.559647	-7.762388	-5.354361
72	C	0	-12.547617	-9.271165	-5.215868
73	O	0	-11.481415	-9.879393	-5.048041
74	C	0	-12.859818	-7.101376	-3.995855
75	C	0	-13.025999	-5.636133	-4.144758
76	C	0	-14.155590	-4.908433	-3.909768
77	C	0	-12.050647	-4.714488	-4.650912
78	N	0	-13.937314	-3.584046	-4.212493
79	C	0	-12.656118	-3.438687	-4.676002
80	C	0	-10.715562	-4.841547	-5.065737
81	C	0	-11.976177	-2.290252	-5.109063
82	C	0	-10.040349	-3.706094	-5.499131
83	C	0	-10.675201	-2.444789	-5.520315
84	H	0	-14.465919	-7.486307	-6.165432
85	H	0	-13.344388	-6.341960	-6.650030
86	H	0	-11.548894	-7.506877	-5.675158
87	H	0	-12.053600	-7.347066	-3.291904
88	H	0	-13.781749	-7.537599	-3.591012
89	H	0	-15.111851	-5.241612	-3.538248
90	H	0	-14.615994	-2.846354	-4.117169
91	H	0	-10.207444	-5.799914	-5.037493
92	H	0	-12.468019	-1.323754	-5.123714
93	H	0	-9.012646	-3.788881	-5.823423
94	H	0	-10.125590	-1.581182	-5.880453
95	N	0	-12.410022	3.090300	-8.500061
96	C	0	-11.834949	2.511273	-7.275319

97	C	0	-10.796699	1.409872	-7.550830
98	O	0	-9.766612	1.339559	-6.860317
99	C	0	-12.914796	2.031556	-6.292924
100	C	0	-12.422688	1.687260	-4.870874
101	C	0	-11.684306	2.860854	-4.177271
102	C	0	-13.573415	1.197491	-3.987442
103	H	0	-11.734301	3.537605	-9.107748
104	H	0	-13.038751	2.476789	-9.007950
105	H	0	-11.268048	3.310723	-6.784981
106	H	0	-10.993308	0.699066	-8.366547
107	H	0	-13.430900	1.156614	-6.714450
108	H	0	-13.655930	2.839847	-6.234285
109	H	0	-11.696624	0.863655	-4.959790
110	H	0	-11.451153	2.591344	-3.142413
111	H	0	-12.317172	3.756522	-4.164440
112	H	0	-10.744894	3.102664	-4.681619
113	H	0	-14.142183	0.395918	-4.472040
114	H	0	-14.268888	2.017966	-3.771559
115	H	0	-13.189688	0.814500	-3.036695
116	N	0	-6.891174	1.455322	-6.436160
117	C	0	-6.340050	0.246024	-5.797466
118	C	0	-5.025968	-0.194196	-6.441877
119	O	0	-4.029823	-0.370427	-5.742530
120	C	0	-7.390491	-0.880843	-5.824474
121	C	0	-6.880020	-2.234825	-5.355196
122	C	0	-6.643086	-2.482794	-4.003928
123	C	0	-6.700251	-3.280155	-6.272212
124	C	0	-6.185430	-3.750859	-3.567950
125	C	0	-6.247106	-4.558960	-5.838573
126	C	0	-5.987152	-4.780457	-4.492098
127	H	0	-7.890544	1.428601	-6.598791

128	H	0	-6.557916	2.326639	-6.046682
129	H	0	-6.050817	0.423499	-4.754090
130	H	0	-7.770399	-0.963009	-6.849642
131	H	0	-8.221392	-0.546715	-5.193448
132	H	0	-6.800543	-1.693549	-3.273539
133	H	0	-6.902559	-3.111389	-7.325583
134	H	0	-5.984110	-3.921537	-2.518312
135	H	0	-6.110859	-5.350081	-6.566761
136	H	0	-5.636941	-5.749941	-4.155217
137	N	0	-2.269927	2.515179	-5.887402
138	C	0	-1.836194	2.620357	-4.489737
139	C	0	-1.010657	1.405431	-4.050132
140	O	0	-0.084114	1.538114	-3.260996
141	C	0	-3.057731	2.782427	-3.587584
142	C	0	-2.903909	3.000586	-2.081819
143	C	0	-1.924346	4.129321	-1.758002
144	C	0	-4.288632	3.318533	-1.532337
145	H	0	-1.554076	2.720372	-6.572926
146	H	0	-3.148013	2.976407	-6.090216
147	H	0	-1.160973	3.472778	-4.327583
148	H	0	-3.705458	1.909455	-3.759293
149	H	0	-3.609393	3.652814	-3.979743
150	H	0	-2.533427	2.074619	-1.619272
151	H	0	-1.929577	4.333374	-0.681324
152	H	0	-2.218963	5.050449	-2.279177
153	H	0	-0.901578	3.872433	-2.051184
154	H	0	-5.016821	2.552977	-1.823193
155	H	0	-4.638393	4.281271	-1.923040
156	H	0	-4.278617	3.381769	-0.440343
157	N	0	-1.341702	0.222888	-4.564468
158	C	0	-0.601442	-1.005675	-4.212878

159	C	0	0.799493	-1.024735	-4.789771
160	O	0	1.747479	-1.487302	-4.131529
161	C	0	-1.336965	-2.260853	-4.707974
162	S	0	-2.796525	-2.750346	-3.737980
163	H	0	-2.124329	0.169287	-5.216027
164	H	0	-0.461154	-1.051007	-3.127645
165	H	0	-1.695434	-2.097923	-5.726886
166	H	0	-0.634537	-3.096527	-4.727781
167	H	0	-2.139885	-2.974309	-2.548657
168	N	0	-4.258846	-6.771032	2.248831
169	C	0	-3.394472	-5.730271	1.674315
170	C	0	-3.932430	-4.303205	1.899691
171	O	0	-3.147997	-3.373967	2.098596
172	C	0	-3.032193	-6.015201	0.172218
173	C	0	-1.673935	-5.400028	-0.189914
174	C	0	-4.159129	-5.595083	-0.803649
175	C	0	-1.201434	-5.703737	-1.645037
176	H	0	-4.135975	-7.687137	1.836400
177	H	0	-4.266948	-6.799303	3.260972
178	H	0	-2.426873	-5.700548	2.200905
179	H	0	-2.927453	-7.110289	0.109527
180	H	0	-1.721181	-4.312057	-0.052699
181	H	0	-0.915841	-5.775061	0.508554
182	H	0	-3.968848	-5.985091	-1.807269
183	H	0	-4.218301	-4.500376	-0.866240
184	H	0	-5.128232	-5.976411	-0.465041
185	H	0	-1.136155	-6.784455	-1.814095
186	H	0	-0.212614	-5.265117	-1.803762
187	H	0	-1.885912	-5.284346	-2.386747
188	N	0	-5.253829	-4.137198	1.892991
189	C	0	-5.847766	-2.787814	1.983451

190	C	0	-6.129736	-2.351539	3.418950
191	O	0	-6.424773	-1.184450	3.665648
192	C	0	-7.154161	-2.642786	1.130219
193	C	0	-8.306707	-3.497726	1.685690
194	C	0	-6.891630	-3.041883	-0.320564
195	C	0	-9.150348	-2.830999	2.737429
196	H	0	-5.831781	-4.968750	1.846216
197	H	0	-5.093476	-2.087300	1.613392
198	H	0	-7.429910	-1.583287	1.182607
199	H	0	-7.918986	-4.455992	2.059172
200	H	0	-8.954564	-3.758166	0.839789
201	H	0	-6.008022	-2.535071	-0.726435
202	H	0	-7.753936	-2.784654	-0.942733
203	H	0	-6.729113	-4.122053	-0.394532
204	H	0	-10.066086	-3.407803	2.873531
205	H	0	-9.447872	-1.821006	2.439889
206	H	0	-8.659657	-2.765737	3.713214
207	N	0	-1.181779	-2.086749	7.009715
208	C	0	-0.939746	-0.740971	7.538962
209	C	0	-1.278729	-0.593633	9.026583
210	O	0	-1.441693	0.527189	9.505060
211	C	0	-1.717844	0.305728	6.719297
212	C	0	-1.165190	0.534285	5.308497
213	C	0	-1.288080	-0.703074	4.403111
214	O	0	-0.278925	-1.321988	4.029018
215	N	0	-2.521666	-1.089747	4.077090
216	H	0	-0.667100	-2.284631	6.158439
217	H	0	-1.039045	-2.819998	7.698045
218	H	0	0.124617	-0.456272	7.494230
219	H	0	-1.686251	1.257792	7.255891
220	H	0	-2.763544	-0.018074	6.672166

221	H	0	-1.709103	1.358113	4.836347
222	H	0	-0.109449	0.834732	5.372958
223	H	0	-3.227516	-0.470498	4.488646
224	N	0	-9.626930	8.306667	1.095341
225	C	0	-9.505557	7.607301	-0.182685
226	C	0	-10.159713	8.539766	-1.201007
227	O	0	-11.365397	8.778961	-1.142993
228	C	0	-10.222921	6.232782	-0.137351
229	C	0	-9.591607	5.323941	0.928736
230	C	0	-10.206146	5.541966	-1.509531
231	C	0	-10.583027	4.376501	1.582054
232	H	0	-10.578454	8.571328	1.324282
233	H	0	-8.958100	9.049958	1.252158
234	H	0	-8.447671	7.473230	-0.425183
235	H	0	-11.264883	6.444604	0.145575
236	H	0	-9.134934	5.955516	1.699700
237	H	0	-8.781163	4.746906	0.460164
238	H	0	-9.174143	5.409995	-1.854040
239	H	0	-10.753251	6.115400	-2.265589
240	H	0	-10.669552	4.553269	-1.441013
241	H	0	-11.078498	3.742363	0.838223
242	H	0	-11.359533	4.935996	2.114018
243	H	0	-10.088242	3.717352	2.301438
244	N	0	-4.560501	8.952497	-1.476449
245	C	0	-4.687710	8.227889	-0.214980
246	C	0	-3.878950	8.852262	0.906886
247	O	0	-2.720843	9.212450	0.711046
248	C	0	-4.228105	6.783189	-0.416895
249	C	0	-4.513279	5.828194	0.731307
250	C	0	-5.815407	5.413882	1.016185
251	C	0	-3.471959	5.287751	1.484342

252	C	0	-6.081543	4.499159	2.040092
253	C	0	-3.728346	4.359512	2.515909
254	C	0	-5.030638	3.977285	2.782818
255	O	0	-5.290735	3.070471	3.795687
256	H	0	-3.596486	9.037799	-1.787308
257	H	0	-5.034423	9.849130	-1.487547
258	H	0	-5.741721	8.230168	0.078934
259	H	0	-3.152143	6.790869	-0.634013
260	H	0	-4.734942	6.438453	-1.324025
261	H	0	-6.644193	5.801199	0.430303
262	H	0	-2.447243	5.587426	1.288178
263	H	0	-7.095589	4.183540	2.257473
264	H	0	-2.905759	3.955508	3.096962
265	H	0	-4.477996	2.757487	4.231930
266	N	0	0.237006	10.103568	-0.297672
267	C	0	1.073273	9.612578	-1.407959
268	O	0	-0.550729	10.395603	-2.976999
269	C	0	0.873386	8.102496	-1.650333
270	C	0	1.481647	7.194551	-0.599027
271	C	0	2.853555	7.209697	-0.343466
272	C	0	0.683710	6.292888	0.097919
273	C	0	3.412650	6.367883	0.631107
274	C	0	1.232619	5.435713	1.057062
275	C	0	2.593822	5.471248	1.320701
276	H	0	-0.755758	9.918627	-0.379760
277	H	0	0.633298	9.967119	0.620410
278	H	0	2.120713	9.843651	-1.204181
279	H	0	-0.206080	7.924655	-1.713347
280	H	0	1.294922	7.826009	-2.627850
281	H	0	3.499864	7.888651	-0.892476
282	H	0	-0.383979	6.271159	-0.099097

283	H	0	4.475642	6.414404	0.835337
284	H	0	0.600523	4.741914	1.596896
285	H	0	3.012739	4.799775	2.059594
286	N	0	7.266525	1.858776	-4.376455
287	C	0	7.802918	1.036081	-3.304427
288	C	0	8.731078	-0.059476	-3.787794
289	O	0	9.676986	-0.429863	-3.083585
290	C	0	6.690453	0.337130	-2.511380
291	C	0	5.401757	1.113878	-2.366236
292	C	0	5.395343	2.413646	-1.865426
293	C	0	4.175576	0.529119	-2.711314
294	C	0	4.195750	3.123978	-1.725169
295	C	0	2.982848	1.231450	-2.576680
296	C	0	3.001363	2.524711	-2.082046
297	O	0	1.807487	3.213277	-1.957600
298	H	0	7.959091	2.297813	-4.974628
299	H	0	6.519486	1.423894	-4.905856
300	H	0	8.411336	1.660093	-2.642904
301	H	0	7.092472	0.070145	-1.525779
302	H	0	6.450685	-0.612337	-3.007069
303	H	0	6.334353	2.895813	-1.611135
304	H	0	4.146347	-0.481038	-3.108059
305	H	0	4.208129	4.138913	-1.336188
306	H	0	2.028627	0.798705	-2.853793
307	H	0	1.926033	4.068234	-1.499595
308	N	0	7.776268	-5.372573	0.433678
309	C	0	7.644004	-4.823922	1.771429
310	C	0	8.914388	-5.116198	2.573617
311	O	0	9.756252	-5.920828	2.158683
312	C	0	6.445732	-5.416532	2.530800
313	C	0	5.094852	-5.057505	1.950259

314	C	0	4.673151	-5.571768	0.708986
315	C	0	4.234353	-4.212474	2.638356
316	C	0	3.418940	-5.223637	0.175803
317	C	0	2.986095	-3.873155	2.113278
318	C	0	2.587736	-4.381439	0.891166
319	O	0	1.347414	-4.023089	0.395351
320	H	0	8.114278	-6.336150	0.447166
321	H	0	8.349063	-4.797377	-0.177571
322	H	0	7.528106	-3.733692	1.729550
323	H	0	6.500706	-5.057544	3.563373
324	H	0	6.568356	-6.508540	2.544104
325	H	0	5.330379	-6.230930	0.157543
326	H	0	4.537466	-3.817068	3.603950
327	H	0	3.087707	-5.619840	-0.776731
328	H	0	2.286689	-3.293288	2.700405
329	H	0	0.763933	-3.668113	1.096574
330	O	0	20.782872	0.920129	0.561052
331	C	0	21.103141	0.970172	-1.849954
332	O	0	21.655033	2.242041	-1.554992
333	C	0	22.147031	-0.121708	-2.047201
334	N	0	22.780401	-0.191014	-3.383501
335	C	0	22.198503	0.016165	-4.617477
336	N	0	23.136636	-0.167664	-5.574331
337	C	0	24.317028	-0.475988	-4.974698
338	C	0	25.596300	-0.753251	-5.469106
339	N	0	25.848381	-0.785661	-6.787846
340	N	0	26.610545	-1.057671	-4.571019
341	C	0	26.377847	-1.067312	-3.212422
342	N	0	25.114575	-0.787903	-2.735363
343	C	0	24.100876	-0.497893	-3.598219
344	N	0	7.393573	-1.351442	2.889847

345	C	0	6.706616	-1.728878	4.023035
346	O	0	6.930116	-2.818177	4.549789
347	N	0	5.815179	-0.847081	4.602497
348	C	0	5.568231	0.387899	4.025994
349	O	0	4.775829	1.160651	4.572715
350	C	0	6.248175	0.769848	2.874738
351	N	0	6.029702	2.010833	2.283131
352	C	0	6.849094	2.465335	1.271764
353	C	0	6.804732	3.812777	0.897929
354	C	0	7.850694	4.385653	0.170545
355	C	0	7.730721	5.837852	-0.197169
356	C	0	8.963453	3.611340	-0.198008
357	C	0	10.129338	4.170518	-0.981967
358	C	0	9.001375	2.258118	0.178436
359	C	0	7.944702	1.676740	0.907711
360	N	0	7.917395	0.314918	1.230179
361	C	0	7.171170	-0.107106	2.313282
362	C	0	8.890789	-0.631875	0.569646
363	C	0	10.061584	-1.014581	1.493564
364	O	0	10.710456	0.165064	1.925279
365	C	0	11.015355	-1.940286	0.732709
366	O	0	10.323431	-3.125747	0.375589
367	C	0	12.289418	-2.349661	1.489847
368	O	0	12.873504	-1.230287	2.136685
369	C	0	13.294404	-2.916439	0.492299
370	O	0	14.410122	-3.440967	1.195999
371	15	0	15.630377	-4.166164	0.429628
372	O	0	16.748127	-4.354719	1.555266
373	O	0	15.185344	-5.286043	-0.395613
374	O	0	16.096704	-3.076103	-0.616815
375	15	0	16.609690	-1.523932	-0.545982

376	O	0	16.443648	-0.946533	-2.026662
377	O	0	16.015459	-0.792549	0.636972
378	O	0	18.237116	-1.650728	-0.238875
379	C	0	19.172242	-1.775812	-1.272709
380	C	0	20.447041	-1.086879	-0.817377
381	O	0	21.442819	-1.321564	-1.787008
382	C	0	20.284932	0.428002	-0.695303
383	H	0	20.158253	0.626522	1.264172
384	H	0	20.474300	1.064898	-2.737433
385	H	0	21.746086	2.326174	-0.581133
386	H	0	22.972515	0.014373	-1.338306
387	H	0	21.164389	0.261682	-4.778257
388	H	0	26.775554	-1.004079	-7.115794
389	H	0	25.121652	-0.580127	-7.454802
390	H	0	27.175134	-1.298228	-2.525582
391	H	0	5.366040	-1.094364	5.494777
392	H	0	5.964619	4.407745	1.234677
393	H	0	6.814366	6.268765	0.206402
394	H	0	7.717629	5.968656	-1.285074
395	H	0	8.576546	6.419688	0.184460
396	H	0	10.575327	5.027165	-0.466066
397	H	0	9.794575	4.524749	-1.963371
398	H	0	10.897398	3.411802	-1.130008
399	H	0	9.888829	1.679010	-0.036283
400	H	0	9.232198	-0.177427	-0.360011
401	H	0	8.329397	-1.540537	0.337556
402	H	0	9.651270	-1.571963	2.343311
403	H	0	11.626195	-0.047596	2.224840
404	H	0	11.309730	-1.442902	-0.201145
405	H	0	10.486592	-3.871622	0.986699
406	H	0	12.034695	-3.110588	2.242128

407	H	0	13.847246	-1.197217	2.018298
408	H	0	12.822697	-3.693104	-0.119558
409	H	0	13.621025	-2.097877	-0.164154
410	H	0	19.383642	-2.829781	-1.482512
411	H	0	18.804598	-1.307320	-2.192747
412	H	0	20.756118	-1.495152	0.151903
413	H	0	19.231612	0.709598	-0.796099
414	O	0	-13.723818	-9.959186	-5.271026
415	H	0	-13.580558	-10.947523	-5.196727
416	O	0	-9.392492	9.128982	-2.166811
417	H	0	-9.931969	9.724153	-2.762305
418	O	0	-1.369828	-1.701528	9.815875
419	H	0	-1.580089	-1.466588	10.767972
420	O	0	-4.961574	-0.405292	-7.787220
421	H	0	-4.045506	-0.680004	-8.080765
422	O	0	-6.057507	-3.260927	4.434799
423	H	0	-6.235745	-2.832511	5.323558
424	O	0	-18.588550	1.989569	1.269721
425	H	0	-19.424483	1.578615	0.894564
426	N	0	-13.353084	-1.947875	6.383954
427	H	0	-13.430150	-1.233766	7.108490
428	O	0	-4.429239	8.991956	2.147370
429	H	0	-3.790692	9.407473	2.799426
430	O	0	1.015023	-0.542980	-6.042538
431	H	0	1.980699	-0.599628	-6.302485
432	O	0	1.563082	10.890801	-3.486654
433	H	0	1.170957	11.299408	-4.310989
434	O	0	8.511795	-0.650050	-4.996642
435	H	0	9.194638	-1.355324	-5.188423
436	O	0	9.139036	-4.485092	3.755988
437	H	0	10.019261	-4.741582	4.148781

438	O	0	4.109167	-1.934469	9.186967
439	H	0	4.305512	-2.898678	9.169949
440	C	0	0.166793	-0.829214	2.635913
441	C	0	-0.021339	1.650615	2.225744
442	C	0	1.641747	-0.613050	2.805846
443	C	0	1.388670	1.826706	2.609292
444	C	0	2.186949	0.608251	2.826926
445	H	0	2.244355	-1.487532	3.001273
446	O	0	1.886916	2.956247	2.761085
447	H	0	-0.575450	2.529698	1.918287
448	C	0	0.698028	10.357425	-2.679212
449	H	0	-0.927975	10.868283	-3.752734
450	H	0	3.236688	0.769593	3.031287
451	N	0	-0.262334	-1.871275	1.687202
452	H	0	-1.151655	-2.318758	1.961050
453	C	0	-0.132598	-1.686817	0.300094
454	C	0	1.073333	-0.955609	-0.219513
455	H	0	1.979817	-1.239971	0.318191
456	H	0	1.193306	-1.209922	-1.273779
457	H	0	0.943802	0.129329	-0.139654
458	O	0	-0.982688	-2.170130	-0.467764
459	C	0	-0.589019	0.442024	2.266204
460	H	0	-1.628656	0.296359	1.995939

Catechol-O-methyltransferase: Overall Charge = 0, Total energy = -8468.41381102 Ha

1	C	0	3.106895	9.732914	-1.616195
2	C	0	3.508097	8.741468	-2.700066
3	O	0	3.240856	8.948002	-3.885739
4	C	0	1.768231	9.319748	-0.958910
5	C	0	1.846743	8.002666	-0.254332

6	C	0	2.303854	7.772307	1.022263
7	C	0	1.550403	6.710582	-0.820405
8	N	0	2.313058	6.413619	1.285272
9	C	0	1.856578	5.735398	0.171066
10	C	0	1.075212	6.298727	-2.078292
11	C	0	1.690067	4.364277	-0.067744
12	C	0	0.891004	4.941397	-2.311407
13	C	0	1.194158	3.990056	-1.310383
14	H	0	3.877327	9.704967	-0.840144
15	H	0	0.987741	9.303528	-1.731697
16	H	0	1.515336	10.125902	-0.264704
17	H	0	2.629831	8.477164	1.771445
18	H	0	2.620874	6.016544	2.166903
19	H	0	0.853202	7.028927	-2.851676
20	H	0	1.979160	3.616036	0.662594
21	H	0	0.512255	4.600819	-3.268808
22	H	0	1.037627	2.938973	-1.523838
23	N	0	4.255762	4.131815	-2.998914
24	C	0	4.229350	2.807408	-2.361585
25	C	0	5.593738	2.141698	-2.120978
26	O	0	5.714465	0.904627	-2.161018
27	C	0	3.303015	1.798970	-3.076632
28	C	0	3.764113	1.446973	-4.486486
29	S	0	2.584042	0.174782	-5.211254
30	C	0	3.378443	-0.042687	-6.889388
31	H	0	3.437226	4.684827	-2.763965
32	H	0	4.441491	4.122440	-3.995717
33	H	0	3.833871	2.933330	-1.340858
34	H	0	2.300905	2.237514	-3.120456
35	H	0	3.245998	0.886718	-2.475571
36	H	0	3.752452	2.316725	-5.148653

37	H	0	4.762416	1.006197	-4.474889
38	H	0	3.368584	0.904061	-7.428549
39	H	0	2.789639	-0.781803	-7.430269
40	H	0	4.400609	-0.400231	-6.769542
41	N	0	6.623627	2.953577	-1.821393
42	C	0	7.892214	2.408767	-1.359241
43	C	0	7.746886	1.868879	0.043763
44	O	0	6.933360	2.404645	0.812268
45	C	0	8.951168	3.509567	-1.401351
46	C	0	9.276196	3.882804	-2.837431
47	O	0	9.283802	3.023235	-3.745959
48	N	0	9.574825	5.180400	-3.055317
49	H	0	6.444279	3.950975	-1.804126
50	H	0	8.193503	1.612191	-2.047926
51	H	0	9.873127	3.148139	-0.930962
52	H	0	8.614055	4.376959	-0.824595
53	H	0	9.553303	5.870414	-2.321084
54	H	0	9.841221	5.480001	-3.981940
55	N	0	8.574421	0.885281	0.466270
56	C	0	8.432386	0.363587	1.825992
57	C	0	8.667889	1.452589	2.862108
58	O	0	8.108332	1.420986	3.969342
59	C	0	9.402273	-0.826993	2.024248
60	C	0	10.873173	-0.386798	2.004663
61	C	0	9.070950	-1.584237	3.314771
62	H	0	9.187764	0.409903	-0.181895
63	H	0	7.405050	0.003410	1.970354
64	H	0	9.369578	2.255639	2.592396
65	H	0	9.217605	-1.500715	1.175556
66	H	0	11.121350	0.204217	1.117556
67	H	0	11.524986	-1.264302	2.017709

68	H	0	11.108493	0.214132	2.890543
69	H	0	9.233659	-0.950746	4.192682
70	H	0	9.718054	-2.461077	3.406267
71	H	0	8.029097	-1.918811	3.320824
72	N	0	6.410624	-7.948813	-1.827258
73	C	0	5.872887	-6.588553	-1.764143
74	C	0	5.212153	-6.277055	-3.095264
75	O	0	5.305255	-5.134021	-3.585888
76	C	0	6.983308	-5.587328	-1.478215
77	C	0	6.463500	-4.218450	-1.106011
78	O	0	5.243174	-4.111714	-0.653782
79	O	0	7.269432	-3.242198	-1.198340
80	H	0	5.739668	-8.635829	-2.154387
81	H	0	6.858359	-8.243371	-0.966305
82	H	0	5.102842	-6.463621	-0.984124
83	H	0	7.658699	-5.495138	-2.329481
84	H	0	7.567272	-5.956864	-0.625474
85	C	0	-1.194493	-8.671688	-2.088569
86	C	0	-1.873717	-10.032593	-2.285445
87	O	0	-1.582978	-10.981809	-1.544230
88	C	0	-1.792120	-7.865709	-0.888784
89	C	0	-1.094090	-6.506145	-0.718780
90	C	0	0.440734	-6.571002	-0.617464
91	C	0	1.027933	-5.186727	-0.884776
92	N	0	2.535656	-5.167745	-0.805277
93	H	0	-0.190465	-8.990528	-1.773809
94	H	0	-1.699520	-8.476264	0.015020
95	H	0	-2.858359	-7.688848	-1.072107
96	H	0	-1.367467	-5.878773	-1.575334
97	H	0	-1.495442	-6.019621	0.179171
98	H	0	0.829416	-7.255575	-1.379126

99	H	0	0.747859	-6.949691	0.364619
100	H	0	0.660553	-4.435697	-0.179178
101	H	0	0.759649	-4.879302	-1.897015
102	H	0	2.935474	-5.962359	-1.310221
103	H	0	2.865139	-5.174011	0.172177
104	H	0	2.921723	-4.294083	-1.200884
105	N	0	5.776588	-7.413319	3.184918
106	C	0	5.260689	-6.100651	3.556387
107	C	0	3.744371	-6.093413	3.658087
108	O	0	3.059150	-6.898361	3.044176
109	C	0	5.718995	-5.054931	2.510842
110	C	0	5.566763	-3.612894	2.991056
111	O	0	5.470499	-3.371750	4.226853
112	O	0	5.573116	-2.690379	2.075101
113	H	0	5.709466	-8.093528	3.936039
114	H	0	5.325222	-7.773623	2.347756
115	H	0	5.689037	-5.811645	4.519854
116	H	0	5.188717	-5.183329	1.561041
117	H	0	6.782057	-5.238419	2.314981
118	N	0	3.201518	-5.074079	4.377341
119	C	0	1.793155	-4.757188	4.308575
120	C	0	0.849138	-5.790659	4.890166
121	O	0	-0.305162	-5.901526	4.502819
122	C	0	1.310269	-4.384666	2.900103
123	C	0	2.195508	-3.302764	2.330606
124	O	0	3.033837	-3.579290	1.407861
125	N	0	2.079296	-2.082610	2.851486
126	H	0	3.840007	-4.401421	4.796501
127	H	0	1.660864	-3.881507	4.958628
128	H	0	0.276059	-4.032316	2.956101
129	H	0	1.356990	-5.261087	2.253358

130	H	0	1.372908	-1.878604	3.545460
131	C	0	-3.539061	-3.146111	3.251811
132	C	0	-4.167123	-4.367375	3.873390
133	O	0	-5.370539	-4.540010	4.062492
134	C	0	-4.411715	-2.521214	2.139666
135	C	0	-3.359723	-1.932082	1.183457
136	C	0	-2.230287	-2.974237	1.218177
137	H	0	-3.419990	-2.424558	4.074371
138	H	0	-4.990779	-3.302540	1.634403
139	H	0	-5.104342	-1.776285	2.534652
140	H	0	-2.994815	-0.976352	1.577207
141	H	0	-3.743812	-1.765181	0.174677
142	H	0	-2.450800	-3.788786	0.511967
143	H	0	-1.260327	-2.544446	0.945771
144	C	0	5.265090	4.427016	4.422645
145	C	0	4.629931	5.818420	4.384912
146	O	0	3.532531	6.031462	3.877708
147	C	0	5.626822	4.023651	2.970930
148	C	0	4.428505	3.699028	2.061468
149	C	0	4.181321	2.206524	1.836225
150	O	0	4.336077	1.403730	2.824429
151	O	0	3.753816	1.858459	0.675900
152	H	0	6.193925	4.509283	5.000659
153	H	0	6.232638	4.818559	2.522237
154	H	0	6.264146	3.139562	3.036817
155	H	0	4.548699	4.164025	1.080373
156	H	0	3.508647	4.099419	2.505327
157	C	0	-9.710874	-4.893047	-1.091072
158	C	0	-9.858994	-4.685027	-2.592136
159	O	0	-9.375866	-5.445780	-3.410241
160	C	0	-8.315239	-4.349314	-0.657836

161	C	0	-8.201682	-2.870205	-0.873998
162	C	0	-8.656315	-1.881455	-0.034359
163	C	0	-7.734031	-2.210118	-2.073427
164	N	0	-8.499368	-0.643794	-0.639592
165	C	0	-7.930947	-0.814267	-1.888169
166	C	0	-7.190731	-2.670102	-3.287159
167	C	0	-7.581422	0.125370	-2.866676
168	C	0	-6.855105	-1.741140	-4.264242
169	C	0	-7.044838	-0.356338	-4.053972
170	H	0	-10.488352	-4.331791	-0.569676
171	H	0	-8.197651	-4.612166	0.396774
172	H	0	-7.546538	-4.890104	-1.223877
173	H	0	-9.093327	-1.963927	0.948521
174	H	0	-8.770929	0.237636	-0.235419
175	H	0	-7.028029	-3.728480	-3.470563
176	H	0	-7.730901	1.186955	-2.703584
177	H	0	-6.436317	-2.089501	-5.202355
178	H	0	-6.766875	0.342438	-4.834617
179	N	0	-3.626827	9.911137	-1.928269
180	C	0	-3.122597	8.562611	-2.162539
181	C	0	-3.931125	7.895284	-3.278567
182	O	0	-5.131476	7.725770	-3.116652
183	C	0	-3.211414	7.712139	-0.883165
184	C	0	-2.760393	6.273290	-1.167566
185	C	0	-2.353228	8.331316	0.222675
186	H	0	-3.389146	10.587029	-2.644666
187	H	0	-4.613740	9.932840	-1.691421
188	H	0	-2.072972	8.628661	-2.469192
189	H	0	-4.266606	7.705740	-0.572001
190	H	0	-2.759556	5.675120	-0.252698
191	H	0	-1.737915	6.265698	-1.565199

192	H	0	-3.412917	5.769026	-1.889382
193	H	0	-2.627822	9.373207	0.394296
194	H	0	-1.292190	8.291045	-0.057857
195	H	0	-2.474914	7.771091	1.154692
196	C	0	-8.838922	5.899399	1.168951
197	C	0	-9.764986	4.724588	1.427129
198	O	0	-9.948511	4.293754	2.574258
199	C	0	-7.377686	5.397507	1.095726
200	C	0	-7.091389	4.332129	0.024844
201	C	0	-7.518957	4.803054	-1.371732
202	C	0	-5.594200	3.995870	0.034769
203	H	0	-9.118382	6.379957	0.229605
204	H	0	-7.102853	5.013029	2.087608
205	H	0	-6.758355	6.282586	0.906065
206	H	0	-7.657726	3.419842	0.274859
207	H	0	-7.022423	5.750234	-1.618421
208	H	0	-8.600952	4.946495	-1.441081
209	H	0	-7.229956	4.066149	-2.127849
210	H	0	-5.012324	4.895089	-0.197950
211	H	0	-5.355307	3.237713	-0.719025
212	H	0	-5.273767	3.620002	1.012324
213	N	0	-9.814882	1.762594	4.224494
214	C	0	-8.662338	1.983399	5.067111
215	C	0	-8.807703	3.217976	5.942894
216	O	0	-9.754587	3.986306	5.823460
217	C	0	-7.383338	2.161372	4.225768
218	C	0	-7.120300	0.952975	3.336738
219	S	0	-5.408005	1.114901	2.569696
220	C	0	-5.740351	0.555775	0.816191
221	H	0	-10.019747	2.558483	3.622069
222	H	0	-10.638028	1.458397	4.732242

223	H	0	-8.522027	1.119628	5.727799
224	H	0	-6.538870	2.332197	4.902007
225	H	0	-7.492438	3.057097	3.600709
226	H	0	-7.154325	0.021623	3.906180
227	H	0	-7.850905	0.896277	2.529007
228	H	0	-6.455487	1.230337	0.344785
229	H	0	-6.117121	-0.468000	0.805176
230	H	0	-4.791309	0.603590	0.279186
231	Mg	0	4.573468	-2.542789	0.390234
232	C	0	-0.419722	0.136624	-0.833885
233	C	0	0.840660	0.579447	-0.213537
234	C	0	1.954689	-0.171246	-0.288348
235	C	0	-0.382616	-1.090097	-1.647809
236	C	0	0.742470	-1.820364	-1.758117
237	H	0	2.877510	0.207267	0.156812
238	H	0	2.678377	-1.308386	2.588757
239	O	0	-5.124044	-4.650601	-4.916870
240	C	0	-4.016178	-3.846886	-4.415014
241	C	0	-2.922375	-3.710003	-5.481079
242	C	0	-3.479149	-4.524538	-3.153214
243	O	0	-3.099609	-5.879594	-3.437951
244	C	0	-4.474977	-4.556985	-1.992398
245	H	0	-4.815560	-5.213250	-5.653575
246	H	0	-4.436894	-2.874091	-4.151254
247	H	0	-3.336442	-3.298511	-6.405028
248	H	0	-2.484096	-4.692003	-5.685455
249	H	0	-2.128381	-3.045424	-5.130982
250	H	0	-2.556173	-4.022253	-2.846162
251	H	0	-3.895938	-6.384272	-3.701374
252	H	0	-4.782664	-3.549021	-1.695922
253	H	0	-4.025577	-5.062938	-1.133458

254	H	0	-5.370626	-5.107317	-2.300244
255	N	0	4.338059	3.438109	4.952536
256	H	0	4.256390	2.568644	4.405304
257	H	0	4.342194	3.319984	5.955807
258	O	0	5.334067	6.872326	4.902963
259	H	0	4.843061	7.740610	4.809870
260	O	0	-2.781082	-10.207171	-3.292204
261	H	0	-3.133819	-11.143293	-3.325222
262	N	0	-1.091932	-7.847626	-3.259480
263	H	0	-0.824820	-8.340852	-4.094773
264	H	0	-1.799286	-7.129183	-3.386564
265	N	0	-9.794755	-6.306325	-0.742699
266	H	0	-10.708801	-6.739377	-0.848412
267	H	0	-9.076663	-6.861944	-1.197821
268	O	0	-10.555888	-3.586081	-3.030717
269	H	0	-10.560670	-3.521334	-4.030822
270	O	0	-10.397303	4.120733	0.377521
271	H	0	-10.987593	3.360450	0.661356
272	N	0	-8.949938	6.870858	2.256731
273	H	0	-8.848780	6.453071	3.183591
274	H	0	-9.784308	7.445986	2.207336
275	N	0	3.015587	11.085392	-2.138736
276	H	0	2.401068	11.166141	-2.943384
277	H	0	3.916304	11.510654	-2.330489
278	O	0	4.256070	7.657588	-2.351207
279	H	0	4.511667	7.131811	-3.156370
280	O	0	-3.313730	7.503637	-4.433383
281	H	0	-3.941220	7.089961	-5.093748
282	O	0	-7.845906	3.501553	6.884555
283	H	0	-8.068350	4.343296	7.388362
284	O	0	4.497415	-7.239768	-3.740984

285	H	0	4.123860	-6.926115	-4.620063
286	O	0	1.282870	-6.614999	5.892672
287	H	0	0.553001	-7.251068	6.177514
288	O	0	-3.237187	-5.275602	4.263540
289	H	0	-3.629779	-6.057658	4.699765
290	N	0	-2.228328	-3.423292	2.625010
291	H	0	-1.870841	-4.354298	2.815214
292	H	0	6.570004	-1.883911	-0.814472
293	O	0	5.830372	-1.291169	-0.461332
294	H	0	5.800194	-0.406786	-0.868212
295	H	0	-1.298468	-1.356730	-2.162084
296	H	0	0.793758	-2.699994	-2.385885
297	H	0	0.864183	1.526688	0.318155
298	C	0	1.936246	-1.455985	-0.990548
299	O	0	2.918993	-2.250633	-0.916132
300	N	0	-1.541503	0.760847	-0.727890
301	C	0	-2.002604	1.863390	1.427896
302	H	0	-2.131661	2.861295	1.845464
303	H	0	-2.903233	1.275861	1.627676
304	H	0	-1.166656	1.356017	1.918823
305	C	0	-1.769075	1.975469	-0.048574
306	O	0	-1.864337	3.029134	-0.687318

Aldehyde Dehydrogenase: Overall Charge = 0, Total energy = -6683.04500365 Ha

1	N	0	5.581712	0.771113	-6.851193
2	C	0	5.933234	0.917263	-5.441473
3	C	0	7.191213	0.179705	-5.035578
4	O	0	8.026699	0.733567	-4.290529
5	H	0	6.254631	1.164118	-7.496827
6	H	0	5.287443	-0.159873	-7.117166

7	H	0	6.111735	1.962976	-5.194968
8	H	0	5.107149	0.555654	-4.821215
9	N	0	9.590474	2.638958	-2.736041
10	C	0	10.553987	2.338013	-1.692426
11	C	0	11.677922	1.406708	-2.083006
12	O	0	12.774806	1.367774	-1.525159
13	C	0	9.792595	1.700493	-0.480738
14	O	0	8.476985	2.300475	-0.425433
15	C	0	10.506960	1.924146	0.837823
16	H	0	9.287484	1.842986	-3.295516
17	H	0	9.823483	3.443083	-3.305966
18	H	0	11.019873	3.266406	-1.354890
19	H	0	9.674853	0.627011	-0.690514
20	H	0	8.306487	2.623356	-1.354905
21	H	0	10.619493	2.996606	1.022095
22	H	0	11.498532	1.464048	0.821978
23	H	0	9.933979	1.498665	1.665057
24	N	0	0.854692	-8.102216	0.268759
25	C	0	1.472787	-7.683415	-0.987738
26	C	0	2.861953	-8.301084	-0.986340
27	O	0	3.790289	-7.750633	-0.388829
28	C	0	1.575156	-6.157039	-1.181274
29	C	0	0.206350	-5.523827	-1.198373
30	C	0	-0.384222	-5.045176	-0.020299
31	C	0	-0.517206	-5.449065	-2.396351
32	C	0	-1.671592	-4.500449	-0.040634
33	C	0	-1.800278	-4.903192	-2.421222
34	C	0	-2.381292	-4.427301	-1.242599
35	H	0	1.261296	-7.669192	1.091803
36	H	0	-0.156883	-8.059271	0.270105
37	H	0	0.894637	-8.119778	-1.806785

38	H	0	2.100873	-5.948547	-2.120981
39	H	0	2.182879	-5.753465	-0.364182
40	H	0	0.169238	-5.094980	0.912338
41	H	0	-0.065446	-5.813443	-3.314398
42	H	0	-2.124704	-4.136607	0.875935
43	H	0	-2.339973	-4.846728	-3.359445
44	H	0	-3.383782	-4.011139	-1.250815
45	N	0	5.821434	-6.025826	-2.759260
46	C	0	5.929453	-4.740067	-2.053730
47	C	0	6.504753	-4.951562	-0.660178
48	O	0	7.421350	-4.232593	-0.253449
49	C	0	4.578950	-4.001388	-1.994746
50	C	0	4.684553	-2.715390	-1.166580
51	C	0	4.100356	-3.698624	-3.417110
52	H	0	5.244678	-6.695047	-2.254252
53	H	0	6.713866	-6.424443	-3.027958
54	H	0	6.660162	-4.108754	-2.566691
55	H	0	3.866547	-4.680516	-1.508159
56	H	0	4.916566	-2.906044	-0.113597
57	H	0	3.738988	-2.166353	-1.197916
58	H	0	5.467552	-2.060801	-1.570113
59	H	0	3.116890	-3.219664	-3.395028
60	H	0	4.039692	-4.612057	-4.011004
61	H	0	4.799737	-3.011449	-3.910408
62	N	0	9.835958	-3.696190	1.135848
63	C	0	9.756420	-2.866445	2.360887
64	C	0	10.646269	-3.412124	3.460558
65	O	0	11.153668	-2.658728	4.272302
66	C	0	8.320145	-2.671773	2.908929
67	C	0	7.560116	-1.662998	2.097694
68	C	0	8.090350	-0.667752	1.313248

69	C	0	6.127461	-1.487895	2.052949
70	N	0	7.083826	0.125286	0.787178
71	C	0	5.866717	-0.356790	1.226170
72	C	0	5.048788	-2.182865	2.628404
73	C	0	4.561746	0.087216	0.972194
74	C	0	3.755399	-1.746909	2.371832
75	C	0	3.516256	-0.620787	1.551517
76	H	0	9.149059	-3.401538	0.447216
77	H	0	9.680211	-4.678533	1.351491
78	H	0	10.191101	-1.885844	2.156304
79	H	0	7.813735	-3.644969	2.893709
80	H	0	8.364829	-2.347588	3.957722
81	H	0	9.120603	-0.456655	1.082314
82	H	0	7.273063	0.970896	0.248639
83	H	0	5.225143	-3.046873	3.261202
84	H	0	4.375463	0.948772	0.340807
85	H	0	2.912859	-2.274312	2.804588
86	H	0	2.494569	-0.306682	1.366781
87	N	0	-12.617794	3.065600	-1.706892
88	C	0	-11.354457	2.443010	-2.074816
89	C	0	-10.619704	1.991008	-0.815672
90	O	0	-10.009933	0.924216	-0.793570
91	C	0	-10.474994	3.458473	-2.840076
92	C	0	-11.093513	3.886733	-4.126764
93	N	0	-11.157273	3.075834	-5.250253
94	C	0	-11.742117	5.047339	-4.485905
95	C	0	-11.822097	3.761105	-6.232149
96	N	0	-12.191918	4.962232	-5.802746
97	H	0	-13.320540	2.424537	-1.356658
98	H	0	-12.992051	3.685303	-2.418321
99	H	0	-11.464748	1.537114	-2.688151

100	H	0	-9.489096	3.011957	-3.010059
101	H	0	-10.336429	4.339258	-2.205203
102	H	0	-10.768302	2.148704	-5.335886
103	H	0	-11.898702	5.928677	-3.886769
104	H	0	-12.002241	3.354140	-7.211364
105	N	0	-10.719672	2.794134	0.235214
106	C	0	-10.116584	2.475821	1.521127
107	C	0	-10.445458	1.101947	2.077629
108	O	0	-9.591741	0.474958	2.684165
109	H	0	-11.383650	3.556691	0.156389
110	H	0	-10.440677	3.227767	2.243927
111	H	0	-9.026869	2.510388	1.457507
112	N	0	-9.956444	-2.018506	-1.394184
113	C	0	-8.703080	-2.201778	-2.111348
114	C	0	-8.168976	-3.623770	-1.935874
115	O	0	-8.162167	-4.160252	-0.823714
116	C	0	-7.579241	-1.240687	-1.590210
117	C	0	-6.365495	-1.140765	-2.480671
118	C	0	-6.142019	0.025221	-3.230704
119	C	0	-5.443058	-2.188232	-2.609067
120	C	0	-5.046045	0.144474	-4.080796
121	C	0	-4.346088	-2.088090	-3.463104
122	C	0	-4.145585	-0.915993	-4.204091
123	O	0	-3.081142	-0.764856	-5.065800
124	H	0	-10.211246	-1.033052	-1.340874
125	H	0	-9.927250	-2.435935	-0.466848
126	H	0	-8.852992	-2.017119	-3.178233
127	H	0	-8.044537	-0.255556	-1.485614
128	H	0	-7.305018	-1.577537	-0.580507
129	H	0	-6.838029	0.853745	-3.138409
130	H	0	-5.567522	-3.095098	-2.023797

131	H	0	-4.867956	1.050265	-4.647863
132	H	0	-3.636516	-2.904212	-3.551408
133	H	0	-2.337842	-1.383207	-4.843407
134	N	0	-6.077715	-5.599431	3.078674
135	C	0	-5.096672	-4.603952	3.486039
136	C	0	-5.221088	-4.369290	4.969954
137	O	0	-6.323075	-4.152207	5.477344
138	C	0	-5.260431	-3.213441	2.828164
139	S	0	-5.193584	-3.295308	0.956672
140	H	0	-7.043342	-5.321413	3.211569
141	H	0	-5.892911	-6.022366	2.178905
142	H	0	-4.098946	-4.986097	3.259300
143	H	0	-6.207611	-2.760330	3.129632
144	H	0	-4.442993	-2.547002	3.111341
145	H	0	-6.470327	-3.743497	0.718138
146	N	0	-4.609059	-1.724848	6.498534
147	C	0	-5.188472	-0.400864	6.726733
148	C	0	-6.623323	-0.233405	6.237518
149	O	0	-7.119072	0.890397	6.212390
150	C	0	-4.301441	0.719180	6.134056
151	C	0	-4.235862	0.650644	4.596786
152	C	0	-2.910505	0.672527	6.777629
153	C	0	-3.469448	1.828100	3.985036
154	H	0	-5.333017	-2.437108	6.518585
155	H	0	-4.035247	-1.798109	5.666022
156	H	0	-5.252712	-0.225184	7.810714
157	H	0	-4.784246	1.665828	6.402670
158	H	0	-5.256293	0.637573	4.192814
159	H	0	-3.755249	-0.284273	4.279079
160	H	0	-2.329750	1.557868	6.507727
161	H	0	-2.356329	-0.213441	6.451532

162	H	0	-2.988240	0.639301	7.868668
163	H	0	-3.860026	2.777784	4.367933
164	H	0	-3.567224	1.830474	2.895026
165	H	0	-2.403573	1.781867	4.224772
166	N	0	-5.482307	5.893382	2.476670
167	C	0	-4.658722	4.870588	1.841354
168	C	0	-3.471589	5.410335	1.066329
169	O	0	-2.990245	4.746139	0.155443
170	H	0	-4.990267	6.442985	3.170549
171	H	0	-5.988529	6.479955	1.824759
172	H	0	-5.237585	4.267776	1.139835
173	H	0	-4.258382	4.192474	2.601829
174	N	0	0.207447	6.030761	0.877826
175	C	0	1.381599	5.443129	1.499419
176	C	0	2.541408	5.701434	0.543271
177	O	0	2.411244	5.503541	-0.648188
178	C	0	1.192687	3.929362	1.716623
179	C	0	2.455594	3.305750	2.324413
180	C	0	-0.034306	3.653063	2.590814
181	H	0	-0.020669	5.553424	0.007154
182	H	0	0.330497	7.024817	0.693735
183	H	0	1.563186	5.927187	2.466047
184	H	0	1.027228	3.487762	0.722162
185	H	0	2.316947	2.231123	2.473785
186	H	0	2.672703	3.757042	3.300117
187	H	0	3.332241	3.443010	1.682909
188	H	0	0.091956	4.111335	3.578984
189	H	0	-0.163858	2.575312	2.729726
190	H	0	-0.940151	4.060311	2.135763
191	N	0	3.656639	6.191381	1.057220
192	C	0	4.798170	6.483339	0.202150

193	C	0	6.082366	6.128940	0.941643
194	O	0	6.109983	6.078645	2.171208
195	C	0	4.826316	7.984735	-0.162056
196	O	0	6.014266	8.318222	-0.910101
197	H	0	3.796495	6.300318	2.054183
198	H	0	4.706555	5.894208	-0.712614
199	H	0	4.751229	8.575487	0.756972
200	H	0	3.970915	8.209411	-0.799722
201	H	0	6.775726	8.466257	-0.314028
202	N	0	7.154466	5.908741	0.180657
203	C	0	8.444519	5.513411	0.752136
204	C	0	9.028279	6.545685	1.736298
205	O	0	9.815113	6.192537	2.638887
206	C	0	9.455541	5.379783	-0.394882
207	H	0	7.094147	6.029887	-0.821815
208	H	0	8.349092	4.579347	1.310742
209	H	0	9.572154	6.339931	-0.905681
210	H	0	9.104416	4.632109	-1.106963
211	H	0	10.428420	5.073886	-0.006099
212	C	0	-2.119487	0.273153	-2.051165
213	C	0	-2.381354	1.714261	-2.181745
214	C	0	-4.280421	1.515207	-0.643019
215	O	0	10.893275	-4.817739	3.548543
216	H	0	11.476198	-4.992535	4.316691
217	O	0	7.422528	-1.150851	-5.504379
218	H	0	8.280070	-1.472517	-5.154197
219	O	0	-7.716937	-4.211808	-3.061595
220	H	0	-7.343093	-5.105529	-2.904954
221	O	0	-2.913957	6.685917	1.392777
222	H	0	-1.959830	6.674756	1.120015
223	O	0	8.658376	7.920856	1.606872

224	H	0	9.121209	8.447196	2.292334
225	O	0	3.087652	-9.528684	-1.684000
226	H	0	4.018466	-9.806406	-1.549489
227	O	0	-4.056006	-4.397123	5.798436
228	H	0	-4.284005	-4.177126	6.726483
229	O	0	-7.373498	-1.375592	5.816559
230	H	0	-8.267933	-1.088238	5.534698
231	O	0	5.982556	-5.988181	0.173655
232	H	0	6.460658	-5.975651	1.030221
233	O	0	11.325498	0.552469	-3.077607
234	H	0	12.036526	-0.082432	-3.304223
235	O	0	-11.718397	0.695300	1.875764
236	H	0	-11.900536	-0.193164	2.246847
237	C	0	-3.993275	0.074879	-0.486582
238	O	0	-5.223374	2.051673	-0.026741
239	H	0	-4.641535	-0.488988	0.175730
240	H	0	-1.720673	2.263374	-2.841591
241	C	0	-3.406390	2.296150	-1.540648
242	H	0	-3.613233	3.354382	-1.618269
243	C	0	-2.979807	-0.509937	-1.146107
244	H	0	-2.774401	-1.571850	-1.039401
245	N	0	-1.159610	-0.231795	-2.742604
246	C	0	0.332182	-2.015860	-1.970993
247	H	0	-0.153821	-2.344138	-1.043496
248	H	0	0.844766	-2.871909	-2.408143
249	H	0	1.041604	-1.223898	-1.724493
250	C	0	-0.715644	-1.523638	-2.920207
251	O	0	-1.113887	-2.199994	-3.897323

Sult1a3: Overall Charge = 0, Total energy = -5481.80349221 Ha

1	N	0	-0.326619	6.471363	4.876634
2	C	0	-1.654234	5.930497	5.190060
3	C	0	-2.167416	6.470315	6.517104
4	O	0	-3.328594	6.868262	6.629428
5	C	0	-1.635883	4.390507	5.178979
6	C	0	-1.686130	3.799016	3.780280
7	C	0	-0.992540	4.370854	2.701184
8	C	0	-2.458249	2.652320	3.545934
9	C	0	-1.077854	3.812218	1.423010
10	C	0	-2.535159	2.086049	2.271274
11	C	0	-1.845885	2.664985	1.203695
12	H	0	-0.317104	7.463983	4.671705
13	H	0	0.382070	6.216266	5.557025
14	H	0	-2.365075	6.287577	4.441460
15	H	0	-0.734321	4.059482	5.711422
16	H	0	-2.492882	4.012875	5.748078
17	H	0	-0.394705	5.257053	2.879924
18	H	0	-3.009169	2.204743	4.367853
19	H	0	-0.549795	4.277206	0.595109
20	H	0	-3.143961	1.202897	2.109051
21	H	0	-1.907649	2.232962	0.210092
22	C	0	-1.840431	-7.009329	1.386511
23	C	0	-1.295032	-8.275772	0.746270
24	O	0	-0.874969	-9.188326	1.446454
25	C	0	-0.656693	-6.198490	2.002340
26	C	0	-1.200979	-5.715817	3.358075
27	C	0	-2.089748	-6.880799	3.795293
28	H	0	-2.328623	-6.423556	0.606931
29	H	0	-0.338854	-5.388313	1.345953

30	H	0	0.195920	-6.864052	2.168152
31	H	0	-1.802285	-4.809439	3.231667
32	H	0	-0.402537	-5.504128	4.070754
33	H	0	-1.481172	-7.702405	4.194667
34	H	0	-2.836784	-6.599523	4.539104
35	N	0	-8.105324	3.330630	-4.262581
36	C	0	-7.519578	2.755468	-3.070916
37	C	0	-7.866124	3.727619	-1.944037
38	O	0	-7.251661	4.783402	-1.808496
39	C	0	-6.022217	2.550544	-3.236171
40	C	0	-5.375020	1.651676	-2.203013
41	C	0	-6.077745	1.108088	-1.118543
42	C	0	-4.013341	1.336766	-2.345768
43	C	0	-5.432220	0.265932	-0.205274
44	C	0	-3.368692	0.499505	-1.435108
45	C	0	-4.079738	-0.047916	-0.359899
46	H	0	-7.728193	4.234647	-4.524596
47	H	0	-9.116196	3.296862	-4.308884
48	H	0	-8.017305	1.804634	-2.866501
49	H	0	-5.872038	2.121577	-4.232998
50	H	0	-5.529622	3.531405	-3.234632
51	H	0	-7.131146	1.329922	-0.967456
52	H	0	-3.457172	1.756285	-3.178282
53	H	0	-5.992095	-0.139260	0.631503
54	H	0	-2.311169	0.287897	-1.559132
55	H	0	-3.584794	-0.706911	0.346242
56	N	0	-0.258643	8.747404	-1.519649
57	C	0	1.089805	8.784744	-2.072307
58	C	0	1.540960	10.172182	-2.544288
59	O	0	0.724572	11.005528	-2.948647
60	C	0	1.326597	7.765694	-3.202346

61	C	0	1.195398	6.311695	-2.694185
62	O	0	1.263882	5.398716	-3.577145
63	O	0	1.048230	6.140132	-1.428759
64	H	0	-0.421792	7.816131	-1.146533
65	H	0	-0.969919	9.026031	-2.189997
66	H	0	1.767559	8.492541	-1.263963
67	H	0	2.328100	7.881012	-3.634868
68	H	0	0.603389	7.924428	-4.011533
69	N	0	-11.158983	-0.123002	-3.005374
70	C	0	-10.122818	-1.024861	-2.514222
71	C	0	-9.588715	-0.561007	-1.157369
72	O	0	-9.517923	0.633450	-0.890004
73	C	0	-8.948329	-1.059736	-3.514295
74	C	0	-7.730450	-1.894322	-3.089521
75	C	0	-6.651130	-1.848269	-4.181084
76	C	0	-5.512741	-2.852036	-4.019707
77	H	0	-12.051119	-0.229406	-2.534543
78	H	0	-10.856893	0.847490	-3.002162
79	H	0	-10.535306	-2.030470	-2.401279
80	H	0	-8.634225	-0.023334	-3.704712
81	H	0	-9.348667	-1.443941	-4.458421
82	H	0	-8.038266	-2.933032	-2.911160
83	H	0	-7.321816	-1.518627	-2.141139
84	H	0	-7.118417	-2.053920	-5.151361
85	H	0	-6.236143	-0.832006	-4.246624
86	H	0	-4.871160	-2.827337	-4.901273
87	H	0	-5.903601	-3.866406	-3.909007
88	N	0	-7.109635	-2.168807	2.593738
89	C	0	-6.459833	-3.224996	3.363878
90	C	0	-6.703168	-2.967040	4.850989
91	O	0	-6.230932	-3.728481	5.696931

92	C	0	-4.986794	-3.433998	3.016919
93	C	0	-4.857837	-3.889165	1.600624
94	N	0	-4.343680	-5.117732	1.208092
95	C	0	-5.227626	-3.255293	0.439669
96	C	0	-4.403369	-5.186882	-0.152775
97	N	0	-4.931527	-4.065962	-0.645603
98	H	0	-8.106941	-2.133160	2.778161
99	H	0	-6.694148	-1.255574	2.759648
100	H	0	-6.997723	-4.161717	3.171090
101	H	0	-4.565653	-4.172048	3.705490
102	H	0	-4.439633	-2.496300	3.168820
103	H	0	-3.962971	-5.855472	1.830828
104	H	0	-5.706364	-2.300902	0.326336
105	H	0	-4.072419	-6.032635	-0.729345
106	N	0	7.532605	-7.431927	-2.255192
107	C	0	6.798049	-6.188480	-2.450330
108	C	0	7.490711	-5.076038	-1.665794
109	O	0	7.557525	-3.936781	-2.111462
110	C	0	5.348068	-6.371374	-1.938100
111	C	0	4.608572	-5.074931	-1.715616
112	C	0	3.910439	-4.444301	-2.753068
113	C	0	4.651595	-4.459494	-0.457209
114	C	0	3.270568	-3.221674	-2.536200
115	C	0	4.016948	-3.236369	-0.239239
116	C	0	3.323558	-2.612133	-1.278966
117	H	0	8.404958	-7.494789	-2.766049
118	H	0	7.644271	-7.679522	-1.278685
119	H	0	6.807611	-5.887190	-3.498133
120	H	0	5.407995	-6.925335	-0.994106
121	H	0	4.830840	-7.009557	-2.660057
122	H	0	3.867961	-4.914667	-3.730985

123	H	0	5.188528	-4.943878	0.353666
124	H	0	2.730679	-2.742070	-3.346220
125	H	0	4.060525	-2.781491	0.744534
126	H	0	2.858327	-1.644616	-1.122972
127	N	0	8.609666	-1.810692	-3.585037
128	C	0	7.741402	-0.690310	-3.238319
129	C	0	8.515564	0.215070	-2.291022
130	O	0	8.660447	-0.082324	-1.106593
131	C	0	6.388257	-1.173682	-2.716174
132	C	0	5.251244	-0.137282	-2.854665
133	C	0	4.806061	0.414257	-1.520395
134	O	0	3.578770	0.567124	-1.240651
135	O	0	5.766481	0.698642	-0.679875
136	H	0	9.518494	-1.551613	-3.949418
137	H	0	8.627885	-2.547658	-2.890990
138	H	0	7.567901	-0.111181	-4.152889
139	H	0	6.489102	-1.499904	-1.677426
140	H	0	6.146287	-2.063760	-3.303057
141	H	0	5.582355	0.710622	-3.470764
142	H	0	4.380145	-0.571990	-3.349504
143	N	0	7.864656	2.764888	-0.705020
144	C	0	7.143730	3.429259	0.370477
145	C	0	7.198627	2.625950	1.682695
146	O	0	6.970510	3.166285	2.769729
147	C	0	5.705277	3.767734	-0.016113
148	H	0	7.298453	2.013794	-1.100294
149	H	0	8.776377	2.432400	-0.401915
150	H	0	7.660249	4.365744	0.615299
151	H	0	5.162606	2.855739	-0.270401
152	H	0	5.707634	4.433934	-0.882420
153	H	0	5.202423	4.269663	0.814475

154	N	0	7.510860	1.339342	1.581076
155	C	0	7.596214	0.497500	2.762643
156	C	0	8.989247	0.581507	3.364251
157	O	0	9.961659	0.840783	2.654857
158	C	0	7.126500	-0.941141	2.462390
159	C	0	5.632297	-0.970083	2.417354
160	N	0	4.897423	-0.217049	1.505774
161	C	0	4.725278	-1.576122	3.247065
162	C	0	3.594347	-0.356689	1.754452
163	H	0	7.695722	0.921289	0.672236
164	H	0	6.919099	0.923035	3.512033
165	H	0	7.469923	-1.615216	3.250493
166	H	0	7.564215	-1.280305	1.516106
167	H	0	5.306833	0.319337	0.632152
168	H	0	4.866786	-2.230131	4.087935
169	H	0	2.789856	0.098262	1.197297
170	C	0	-0.992694	-2.258852	-1.926917
171	C	0	-0.172185	-1.291530	-2.679182
172	C	0	0.475478	-0.244367	-0.567164
173	C	0	-0.082273	-1.388177	0.174105
174	O	0	-1.819421	-2.998308	-2.503430
175	H	0	-0.178938	-1.378104	-3.758965
176	H	0	0.033754	-1.374668	1.251697
177	O	0	-7.839534	-2.149744	5.142258
178	H	0	-8.028479	-2.178712	6.103956
179	O	0	-1.291057	-8.363298	-0.680965
180	H	0	-0.883788	-9.211410	-0.956738
181	O	0	7.954629	-5.441910	-0.439362
182	H	0	8.356869	-4.695386	0.051900
183	O	0	-1.300674	6.400771	7.558855
184	H	0	-1.685986	6.738000	8.394022

185	O	0	-8.996426	3.420046	-1.257296
186	H	0	-9.252141	4.123962	-0.623693
187	O	0	-9.203173	-1.563737	-0.317194
188	H	0	-8.765189	-1.249572	0.502828
189	O	0	2.889897	10.372026	-2.574429
190	H	0	3.123227	11.242886	-2.958522
191	O	0	8.983471	1.335197	-2.884940
192	H	0	9.424676	1.972628	-2.286237
193	O	0	9.054807	0.250185	4.681281
194	H	0	9.970783	0.253874	5.029280
195	N	0	-2.752094	-7.251308	2.524082
196	H	0	-3.225963	-8.149341	2.519627
197	N	0	3.467429	-1.179315	2.813534
198	H	0	2.590133	-1.458273	3.228979
199	N	0	0.816930	0.799362	0.118390
200	C	0	1.008384	2.069694	-0.482947
201	C	0	2.060805	2.922189	0.137652
202	H	0	2.061813	3.902983	-0.341464
203	H	0	1.856962	3.028612	1.207682
204	H	0	3.026150	2.423672	0.014330
205	O	0	0.240813	2.449225	-1.383117
206	C	0	-0.810928	-2.320996	-0.463913
207	H	0	-1.324176	-3.119004	0.059965
208	C	0	0.527283	-0.337000	-2.040153
209	H	0	1.135476	0.385289	-2.569139
210	H	0	-3.652556	-2.716184	-3.016116
211	H	0	-4.758140	-1.605196	-2.485840
212	H	0	-4.861704	-3.264673	-1.970053
213	N	0	-4.652987	-2.572305	-2.814386

3. Optimized coordinates for Dopamine in the active sites labelled below. Optimizations carried out with M062X/6-31G and implicit solvent using the PCM model in the Gaussian 16 software (see manuscript for references). Columns are: atom number, atom symbol, atom type (gaussian 16), and X, Y and Z coordinates in Angstroms.

Phenylalanine Hydroxylase: Overall Charge = 0, Total energy = -7288.90767565 Ha

1	N	0	-2.907930	9.769585	-0.068134
2	C	0	-2.007834	9.659056	-1.222221
3	C	0	-2.587195	10.395904	-2.428306
4	O	0	-3.029296	9.868886	-3.450190
5	C	0	-1.666922	8.192796	-1.568561
6	C	0	-0.821386	7.586894	-0.471424
7	C	0	-1.410715	7.136563	0.719723
8	C	0	0.575884	7.555071	-0.579539
9	C	0	-0.622815	6.700890	1.786334
10	C	0	1.374052	7.116092	0.478476
11	C	0	0.765071	6.709901	1.661580
12	O	0	1.601619	6.288059	2.709144
13	H	0	-2.432395	9.971298	0.800803
14	H	0	-3.572570	8.994648	0.019496
15	H	0	-1.079385	10.188701	-0.977900
16	H	0	-2.611277	7.646648	-1.677151
17	H	0	-1.140169	8.148324	-2.526965
18	H	0	-2.491233	7.134120	0.829573
19	H	0	1.049188	7.894022	-1.495814
20	H	0	-1.095007	6.373391	2.707576
21	H	0	2.454862	7.112156	0.404488
22	H	0	1.197679	6.392348	3.590803
23	N	0	-6.564314	-0.969383	4.882478
24	C	0	-5.796447	-1.823771	3.927198
25	C	0	-6.151734	-1.357725	2.532390
26	O	0	-7.071204	-0.563247	2.311648
27	C	0	-6.077035	-3.322780	4.123304

28	C	0	-7.472423	-3.771272	3.655634
29	C	0	-7.480260	-4.380121	2.246786
30	N	0	-6.711942	-5.629033	2.190671
31	C	0	-5.527851	-5.821532	1.586458
32	N	0	-4.802587	-4.801690	1.103318
33	N	0	-5.047923	-7.064927	1.459701
34	H	0	-6.463707	-1.307931	5.840983
35	H	0	-7.554276	-0.917096	4.629557
36	H	0	-4.735645	-1.634372	4.117960
37	H	0	-5.921280	-3.546616	5.182497
38	H	0	-5.291681	-3.884230	3.605049
39	H	0	-8.184968	-2.936615	3.668115
40	H	0	-7.870167	-4.517164	4.350240
41	H	0	-8.505997	-4.611489	1.950316
42	H	0	-7.083342	-3.687551	1.501666
43	H	0	-7.153357	-6.439203	2.605508
44	H	0	-5.026411	-3.824228	1.280802
45	H	0	-3.972842	-5.024030	0.562561
46	H	0	-4.136636	-7.200351	1.021746
47	H	0	-5.596287	-7.869804	1.718688
48	N	0	-5.746578	-4.351947	-5.303257
49	C	0	-4.870058	-3.281176	-4.865640
50	C	0	-5.622626	-2.039244	-4.387524
51	O	0	-6.700061	-1.682902	-4.924502
52	C	0	-3.962080	-2.836017	-6.050896
53	C	0	-3.200034	-1.558882	-5.772261
54	C	0	-2.070410	-1.563882	-4.939025
55	C	0	-3.659833	-0.330787	-6.261572
56	C	0	-1.409809	-0.379744	-4.611380
57	C	0	-3.008869	0.864146	-5.943249
58	C	0	-1.880025	0.827170	-5.127476
59	O	0	-1.177232	1.985713	-4.785249
60	H	0	-6.445889	-4.022053	-5.963208

61	H	0	-6.180784	-4.862109	-4.541171
62	H	0	-4.231996	-3.652193	-4.055986
63	H	0	-3.284644	-3.666077	-6.271564
64	H	0	-4.613025	-2.695783	-6.921909
65	H	0	-1.712960	-2.513939	-4.550333
66	H	0	-4.541696	-0.302868	-6.893866
67	H	0	-0.519960	-0.383570	-3.989621
68	H	0	-3.369670	1.808801	-6.338158
69	H	0	-1.356530	2.737681	-5.379193
70	N	0	-5.026709	-1.350192	-3.418434
71	C	0	-5.570773	-0.101193	-2.893980
72	C	0	-4.387344	0.803062	-2.553735
73	O	0	-3.353554	0.338321	-2.000177
74	C	0	-6.372071	-0.282839	-1.560110
75	O	0	-5.469810	-0.494138	-0.432196
76	C	0	-7.309141	-1.473310	-1.599791
77	H	0	-4.151520	-1.662545	-3.018619
78	H	0	-6.231618	0.319550	-3.652432
79	H	0	-6.934195	0.641429	-1.381834
80	H	0	-4.985177	0.290647	-0.065912
81	H	0	-6.727927	-2.396093	-1.683261
82	H	0	-7.975499	-1.403984	-2.462364
83	H	0	-7.902645	-1.507805	-0.683339
84	N	0	-4.485891	2.094316	-2.882692
85	C	0	-3.350321	2.972662	-2.565939
86	C	0	-3.216915	3.165892	-1.064639
87	O	0	-2.089832	3.366270	-0.546662
88	C	0	-3.675445	4.277120	-3.322015
89	C	0	-5.206805	4.257668	-3.473667
90	C	0	-5.545974	2.773064	-3.667237
91	H	0	-2.423557	2.508610	-2.901510
92	H	0	-3.195587	4.245241	-4.303827
93	H	0	-3.310623	5.159860	-2.794053

94	H	0	-5.686400	4.640700	-2.567349
95	H	0	-5.549540	4.862793	-4.313196
96	H	0	-5.471156	2.472840	-4.717692
97	H	0	-6.540486	2.522981	-3.294067
98	N	0	-4.359974	3.168894	-0.351839
99	C	0	-4.383088	3.460874	1.076850
100	C	0	-3.927287	2.268741	1.910566
101	O	0	-4.095596	1.085261	1.484096
102	C	0	-5.749507	4.023646	1.492013
103	C	0	-6.025406	5.364252	0.797594
104	C	0	-4.939390	6.428443	1.051092
105	O	0	-4.293794	6.364140	2.153322
106	O	0	-4.769735	7.316362	0.139703
107	H	0	-5.236347	2.971313	-0.814139
108	H	0	-3.653859	4.264077	1.239018
109	H	0	-5.743376	4.166951	2.576118
110	H	0	-6.535062	3.292773	1.261725
111	H	0	-6.128326	5.239764	-0.284224
112	H	0	-6.980946	5.764722	1.158611
113	N	0	-3.402729	2.537432	3.117817
114	C	0	-2.944942	1.460960	3.999931
115	C	0	-4.084920	0.862563	4.852708
116	O	0	-3.765272	-0.005501	5.715723
117	C	0	-1.900749	2.156347	4.891302
118	C	0	-2.455699	3.587055	5.006058
119	C	0	-2.983277	3.878218	3.596573
120	H	0	-2.521301	0.639976	3.408512
121	H	0	-0.926280	2.161931	4.392163
122	H	0	-1.803553	1.648467	5.851664
123	H	0	-1.702325	4.313665	5.315129
124	H	0	-3.277550	3.616391	5.729453
125	H	0	-3.797783	4.603025	3.555347
126	H	0	-2.180858	4.256446	2.947294

127	N	0	-3.029140	-4.074147	5.466617
128	C	0	-2.050482	-4.064604	4.393812
129	C	0	-0.707075	-4.652801	4.843455
130	O	0	-0.344609	-5.818039	4.667101
131	C	0	-1.989632	-2.631436	3.780954
132	C	0	-0.635286	-2.071270	3.513253
133	N	0	0.325738	-2.639512	2.688512
134	C	0	-0.010167	-1.014039	4.132628
135	C	0	1.488804	-1.923112	2.859472
136	N	0	1.315355	-0.930366	3.725778
137	H	0	-2.806223	-3.467656	6.248865
138	H	0	-3.352986	-4.989481	5.754807
139	H	0	-2.416751	-4.762519	3.640485
140	H	0	-2.468925	-1.950885	4.495377
141	H	0	-2.619093	-2.633044	2.881126
142	H	0	0.178545	-3.403215	2.019033
143	H	0	-0.428042	-0.352147	4.874334
144	H	0	2.425366	-2.185083	2.391563
145	N	0	10.647524	2.622744	2.169498
146	C	0	9.742792	2.299298	1.086423
147	C	0	10.299721	1.215539	0.167631
148	O	0	9.971001	0.027059	0.178122
149	C	0	9.328951	3.516293	0.222459
150	C	0	8.338304	3.180208	-0.855687
151	C	0	8.559835	2.480535	-2.022324
152	C	0	6.939651	3.551356	-0.865672
153	N	0	7.383490	2.395921	-2.749934
154	C	0	6.374714	3.045627	-2.067756
155	C	0	6.130109	4.307732	0.002749
156	C	0	5.031440	3.255036	-2.409744
157	C	0	4.804928	4.540401	-0.346664
158	C	0	4.257975	4.006425	-1.537106
159	H	0	11.402004	3.254446	1.931193

160	H	0	10.197818	2.843965	3.046555
161	H	0	8.842485	1.862179	1.521891
162	H	0	10.240504	3.952763	-0.201572
163	H	0	8.899408	4.259219	0.902304
164	H	0	9.473747	2.060697	-2.410948
165	H	0	7.279453	1.911052	-3.626529
166	H	0	6.536448	4.718196	0.921820
167	H	0	4.615928	2.843558	-3.322442
168	H	0	4.168867	5.114070	0.315775
169	H	0	3.212673	4.184242	-1.764833
170	N	0	8.578500	-1.617056	2.697384
171	C	0	7.349109	-1.536295	1.906294
172	C	0	7.341812	-2.376792	0.626940
173	O	0	6.484825	-3.274717	0.419147
174	C	0	6.861395	-0.097242	1.607048
175	C	0	6.891724	0.787246	2.870313
176	C	0	6.141326	0.148768	4.048584
177	O	0	6.808826	-0.685090	4.770417
178	O	0	4.915654	0.450852	4.214958
179	H	0	8.343758	-1.349185	3.656289
180	H	0	9.334402	-1.051716	2.314132
181	H	0	6.575358	-2.003715	2.524600
182	H	0	7.492095	0.354599	0.829039
183	H	0	5.838560	-0.135548	1.209957
184	H	0	6.446233	1.758365	2.634532
185	H	0	7.930469	0.943121	3.180544
186	N	0	8.303264	-2.148096	-0.263142
187	C	0	8.315792	-2.892958	-1.515455
188	C	0	9.631366	-3.589251	-1.813774
189	O	0	9.932874	-4.026594	-2.924205
190	C	0	7.870620	-2.008954	-2.704315
191	C	0	6.411313	-1.626040	-2.595362
192	C	0	6.013380	-0.503863	-1.855857

193	C	0	5.424698	-2.409124	-3.213245
194	C	0	4.660783	-0.166159	-1.734057
195	C	0	4.074025	-2.067058	-3.113060
196	C	0	3.689114	-0.943495	-2.373982
197	H	0	9.000791	-1.425503	-0.104656
198	H	0	7.576369	-3.694924	-1.395052
199	H	0	8.050380	-2.564327	-3.628813
200	H	0	8.500481	-1.111203	-2.722727
201	H	0	6.768856	0.115420	-1.379204
202	H	0	5.720282	-3.281980	-3.788108
203	H	0	4.374693	0.697746	-1.140720
204	H	0	3.322813	-2.669870	-3.612395
205	H	0	2.638223	-0.678563	-2.302747
206	N	0	4.071479	-3.775903	1.334919
207	C	0	3.258326	-3.324990	0.223892
208	C	0	3.041223	-4.384769	-0.835203
209	O	0	2.091779	-4.355937	-1.642719
210	H	0	5.071482	-3.721554	1.164213
211	H	0	3.742226	-4.619245	1.786312
212	H	0	3.752035	-2.475123	-0.264254
213	H	0	2.258865	-2.990780	0.523520
214	N	0	0.717079	-7.455934	0.810268
215	C	0	-0.706091	-7.215272	1.070005
216	C	0	-1.553701	-6.960720	-0.186110
217	O	0	-2.776013	-6.666773	-0.075664
218	C	0	-0.744421	-5.845564	1.761748
219	O	0	-0.203978	-4.820550	0.874359
220	H	0	0.941105	-8.386038	0.468513
221	H	0	1.310898	-7.219745	1.599447
222	H	0	-1.210847	-7.964387	1.688842
223	H	0	-1.771941	-5.558743	1.972561
224	H	0	-0.164717	-5.893540	2.677940
225	H	0	0.560293	-5.188182	0.380274

226	N	0	-0.907799	-6.968322	-1.349187
227	C	0	-1.454502	-6.446402	-2.584059
228	C	0	-1.580225	-7.490365	-3.671797
229	O	0	-1.366615	-7.242528	-4.862096
230	C	0	-0.536080	-5.303076	-3.017270
231	O	0	-1.009019	-4.575423	-4.167504
232	H	0	0.087988	-7.173780	-1.308105
233	H	0	-2.460633	-6.064803	-2.372789
234	H	0	0.475508	-5.685554	-3.194252
235	H	0	-0.488629	-4.593267	-2.193058
236	H	0	-1.047748	-5.171875	-4.943980
237	O	0	0.485786	-2.556446	-3.063349
238	H	0	1.156663	-3.049175	-2.534924
239	H	0	0.159395	-3.111959	-3.805984
240	O	0	-0.078562	2.049822	-2.192944
241	H	0	-0.637298	2.542854	-1.554106
242	H	0	-0.397583	2.160882	-3.112221
243	O	0	-5.422760	-1.941630	1.567166
244	H	0	-5.516218	-1.439854	0.641558
245	O	0	-2.573510	11.745743	-2.258549
246	H	0	-2.973018	12.218967	-3.017872
247	O	0	-0.023331	-3.811617	5.661186
248	H	0	0.783037	-4.221614	6.039447
249	O	0	-1.979910	-8.692697	-3.220388
250	H	0	-2.087896	-9.357979	-3.932774
251	O	0	11.277628	1.677497	-0.646600
252	H	0	11.689784	0.976588	-1.195426
253	O	0	-5.306023	1.187799	4.556070
254	H	0	-6.111361	0.088723	4.824348
255	O	0	10.436472	-3.720850	-0.730812
256	H	0	11.264027	-4.202673	-0.940286
257	O	0	3.950187	-5.373134	-0.856080
258	H	0	3.780066	-6.029032	-1.565390

259	C	0	0.272621	1.158719	0.978813
260	C	0	-0.186341	2.275181	1.682948
261	H	0	-1.252397	2.401982	1.834547
262	C	0	2.062807	3.128061	1.883095
263	C	0	2.530054	2.011050	1.180394
264	C	0	0.700685	3.248469	2.145200
265	H	0	0.335002	4.109222	2.693628
266	C	0	1.646948	1.033400	0.741309
267	H	0	2.065982	0.190366	0.202116
268	O	0	3.878993	1.870389	0.904510
269	H	0	4.355532	2.715630	1.046069
270	O	0	3.016057	4.053528	2.270467
271	H	0	2.605663	4.925453	2.509646
272	C	0	-0.730264	0.144390	0.475245
273	H	0	-1.638370	0.680828	0.168558
274	H	0	-1.016873	-0.539097	1.288216
275	C	0	-0.194741	-0.645207	-0.709068
276	H	0	0.592869	-1.344357	-0.416692
277	H	0	0.176242	0.052546	-1.465209
278	H	0	-2.036375	-0.851104	-1.705385
279	H	0	-0.795329	-1.977571	-2.148531
280	H	0	-1.673452	-2.141397	-0.706694
281	N	0	-1.273036	-1.463281	-1.360770

Tyrosine Hydroxylase: Overall Charge = +1, Total energy = -11832.0801844 Ha

1	C	0	3.479505	-9.239666	2.945491
2	O	0	5.088684	-7.924893	1.622529
3	C	0	2.131601	-8.542757	2.706267
4	C	0	2.231181	-7.030663	2.903482
5	C	0	1.562886	-8.956065	1.346680
6	H	0	3.278607	-10.319460	2.889092

7	H	0	1.460578	-8.936888	3.481841
8	H	0	2.967676	-6.582649	2.220987
9	H	0	2.561715	-6.810131	3.919764
10	H	0	1.251160	-6.559350	2.779066
11	H	0	0.539955	-8.583372	1.247757
12	H	0	1.508436	-10.047009	1.263843
13	H	0	2.183756	-8.599739	0.511017
14	C	0	3.809118	-7.103864	-2.219003
15	C	0	2.518799	-7.322812	-2.970395
16	O	0	2.282352	-8.454450	-3.431102
17	H	0	4.602976	-7.028249	-2.981340
18	H	0	3.789737	-6.145977	-1.689428
19	C	0	0.350011	-6.368904	-3.611656
20	C	0	-0.384812	-7.707692	-3.637275
21	O	0	-0.204612	-8.583633	-4.507086
22	C	0	0.387008	-5.766925	-5.030181
23	C	0	0.332132	-4.230646	-4.954831
24	C	0	0.581155	-3.592927	-6.323097
25	C	0	-1.032285	-3.832402	-4.375450
26	H	0	-0.260059	-5.716597	-2.990360
27	H	0	-0.476269	-6.120888	-5.606460
28	H	0	1.288223	-6.123834	-5.541150
29	H	0	1.120466	-3.880520	-4.269558
30	H	0	0.509380	-2.502671	-6.262800
31	H	0	-0.165815	-3.943046	-7.044622
32	H	0	1.573683	-3.851708	-6.704408
33	H	0	-1.813013	-3.948788	-5.135143
34	H	0	-1.045566	-2.800597	-4.015467
35	H	0	-1.272714	-4.506573	-3.541431
36	C	0	-2.328652	-8.902054	-2.567839
37	C	0	-3.639497	-8.704340	-3.385800
38	O	0	-3.911310	-7.596722	-3.901353
39	C	0	-2.691215	-9.027334	-1.077147

40	C	0	-1.614810	-9.694636	-0.211127
41	C	0	-1.966912	-9.498770	1.269800
42	C	0	-1.472758	-11.187728	-0.531024
43	H	0	-1.829576	-9.805222	-2.933227
44	H	0	-3.625976	-9.595081	-0.976142
45	H	0	-2.904625	-8.019232	-0.694296
46	H	0	-0.654298	-9.203703	-0.427260
47	H	0	-2.949945	-9.937395	1.479517
48	H	0	-2.009832	-8.435911	1.535424
49	H	0	-1.235424	-9.988582	1.920124
50	H	0	-2.415642	-11.707574	-0.324167
51	H	0	-0.693145	-11.638865	0.090443
52	H	0	-1.206571	-11.365759	-1.577053
53	C	0	-5.756855	-9.576218	-4.212829
54	O	0	-6.371548	-8.339346	-2.191559
55	C	0	-6.411051	-10.936070	-4.405543
56	H	0	-5.582924	-9.091465	-5.178759
57	H	0	-5.793998	-11.537134	-5.079126
58	H	0	-7.406728	-10.825005	-4.845752
59	C	0	-6.364825	-7.553385	1.528123
60	C	0	-5.909214	-6.462112	0.528069
61	C	0	-5.636526	-5.110518	1.138960
62	C	0	-6.529163	-4.050885	0.930547
63	C	0	-6.281410	-2.786644	1.473251
64	C	0	-4.493541	-4.885892	1.919118
65	C	0	-5.134087	-2.572021	2.239987
66	C	0	-7.458639	-7.007337	2.420865
67	O	0	-7.239804	-6.694057	3.620339
68	H	0	-5.530860	-7.816243	2.191516
69	H	0	-6.695078	-6.375072	-0.230831
70	H	0	-5.017178	-6.841438	0.016022
71	H	0	-7.417606	-4.218893	0.328133
72	H	0	-6.977688	-1.972493	1.293554

73	H	0	-3.796940	-5.701897	2.093095
74	H	0	-4.924669	-1.589751	2.647974
75	C	0	-9.746028	-6.352304	2.636034
76	O	0	-10.665843	-7.067437	4.758771
77	C	0	-10.957320	-6.030384	1.749876
78	C	0	-10.672151	-4.935295	0.705446
79	C	0	-11.928721	-4.703896	-0.143430
80	C	0	-10.214322	-3.625725	1.360663
81	H	0	-9.436949	-5.457971	3.184237
82	H	0	-11.286135	-6.948224	1.244794
83	H	0	-11.778137	-5.702926	2.400186
84	H	0	-9.869190	-5.289975	0.043618
85	H	0	-11.738011	-3.961105	-0.923496
86	H	0	-12.747602	-4.334146	0.484676
87	H	0	-12.259063	-5.629912	-0.624228
88	H	0	-10.070544	-2.849474	0.601411
89	H	0	-9.266535	-3.741343	1.895869
90	H	0	-10.970685	-3.270198	2.071019
91	C	0	5.676907	-4.684505	3.173268
92	O	0	6.811612	-3.613898	5.096280
93	C	0	5.811398	-3.876742	1.855436
94	C	0	4.798801	-4.318540	0.806658
95	H	0	5.771248	-5.748337	2.927109
96	H	0	6.828871	-4.023453	1.470113
97	H	0	4.981014	-3.789407	-0.135149
98	H	0	3.787871	-4.080868	1.152272
99	H	0	4.872311	-5.396828	0.626858
100	C	0	11.496877	-4.030632	1.764891
101	O	0	10.543302	-1.746278	1.846570
102	C	0	11.596042	-4.801688	0.411708
103	C	0	10.312067	-4.717079	-0.380020
104	C	0	10.080976	-3.663095	-1.279541
105	C	0	9.295107	-5.664081	-0.197731

106	C	0	8.881127	-3.551941	-1.979766
107	C	0	8.082240	-5.564611	-0.882916
108	C	0	7.883958	-4.507295	-1.770764
109	H	0	12.437008	-4.181895	2.306433
110	H	0	11.819200	-5.846143	0.653456
111	H	0	12.434189	-4.404126	-0.165203
112	H	0	10.860592	-2.923859	-1.441449
113	H	0	9.454586	-6.497702	0.479658
114	H	0	8.714944	-2.746680	-2.692392
115	H	0	7.307254	-6.310608	-0.731899
116	C	0	12.255963	1.318222	-2.269757
117	O	0	14.650255	1.848849	-1.975496
118	C	0	11.478743	2.585247	-2.734611
119	C	0	10.231555	2.907512	-1.916524
120	C	0	9.137971	1.813351	-1.930635
121	H	0	11.875364	0.438103	-2.794347
122	H	0	12.142029	3.457038	-2.674910
123	H	0	11.216896	2.452036	-3.789292
124	H	0	9.812062	3.832863	-2.324919
125	H	0	10.548224	3.128646	-0.889076
126	H	0	9.347630	1.049025	-2.685617
127	H	0	9.070999	1.306446	-0.958849
128	C	0	4.727536	0.039594	-5.255227
129	C	0	4.813612	0.503777	-3.785586
130	O	0	5.515557	1.499958	-3.457314
131	C	0	5.494004	-1.323654	-5.352502
132	C	0	6.975892	-1.228728	-5.787709
133	O	0	7.895637	0.815609	-4.876234
134	H	0	3.672052	-0.121154	-5.492723
135	H	0	5.453138	-1.835867	-4.383876
136	H	0	4.981590	-1.959079	-6.083923
137	H	0	7.016328	-0.751676	-6.771102
138	H	0	7.354553	-2.249843	-5.885328

139	N	0	3.891500	0.000211	-2.959726
140	C	0	3.905189	0.364936	-1.552836
141	O	0	6.070760	-0.747126	-1.714084
142	C	0	2.657945	-0.321872	-0.980974
143	C	0	2.519449	-1.576051	-1.861089
144	C	0	2.932734	-1.084665	-3.256561
145	H	0	3.899948	1.451183	-1.416662
146	H	0	2.795047	-0.555119	0.074956
147	H	0	1.780592	0.324593	-1.096656
148	H	0	1.510065	-1.993283	-1.856797
149	H	0	3.215213	-2.350075	-1.518978
150	H	0	3.389190	-1.873158	-3.856794
151	H	0	2.072648	-0.684014	-3.807139
152	C	0	4.700848	4.117119	2.605387
153	C	0	3.845590	3.485537	3.711023
154	O	0	2.857683	4.101277	4.183462
155	C	0	4.241796	3.455384	1.272681
156	C	0	2.758655	3.448960	1.102025
157	C	0	1.821402	2.436954	1.117187
158	C	0	0.724317	4.321851	0.908693
159	N	0	0.549212	2.993368	0.982031
160	H	0	4.460711	5.182551	2.606484
161	H	0	4.611106	2.422513	1.253461
162	H	0	4.721465	3.985883	0.442584
163	H	0	1.977513	1.375991	1.216767
164	H	0	-0.067392	5.048989	0.801442
165	C	0	3.376796	1.536906	5.150816
166	O	0	2.736527	2.514066	7.288246
167	C	0	3.859313	0.076804	5.218870
168	C	0	3.724662	-0.660454	3.891423
169	O	0	1.335754	-0.752643	4.296861
170	H	0	2.309458	1.606396	4.934196
171	H	0	4.910365	0.077054	5.524003

172	H	0	3.289952	-0.445034	5.993220
173	H	0	4.288709	-1.600739	3.909762
174	H	0	4.156671	-0.108722	3.047543
175	C	0	-0.679469	3.961505	7.121182
176	O	0	0.085500	4.270009	9.375280
177	C	0	-0.919016	2.659456	6.321722
178	C	0	-1.421379	2.927232	4.937368
179	C	0	-0.797441	2.929967	3.716699
180	C	0	-2.914417	3.341628	3.315931
181	N	0	-1.737416	3.174535	2.714630
182	H	0	-1.555640	4.599990	6.912668
183	H	0	-1.631870	2.026998	6.858255
184	H	0	0.027711	2.111264	6.264935
185	H	0	0.241925	2.764125	3.491567
186	H	0	-3.852704	3.527626	2.820836
187	C	0	-10.771213	2.316362	0.280238
188	O	0	-8.957222	3.982437	0.313100
189	C	0	-10.156079	0.889999	0.253493
190	C	0	-8.743270	0.838433	0.806267
191	C	0	-8.505401	0.639300	2.171564
192	C	0	-7.633710	1.044122	-0.030361
193	C	0	-7.207618	0.653717	2.690541
194	C	0	-6.330953	1.074794	0.469956
195	C	0	-6.130421	0.881927	1.835490
196	H	0	-10.856704	2.642495	1.325362
197	H	0	-10.167764	0.544862	-0.786759
198	H	0	-10.811727	0.227978	0.829868
199	H	0	-9.341230	0.470460	2.842902
200	H	0	-7.800204	1.192969	-1.092974
201	H	0	-7.042760	0.493334	3.750789
202	H	0	-5.477142	1.266886	-0.170933
203	C	0	-8.746215	4.049273	-2.421374
204	O	0	-7.969672	6.330911	-2.151351

205	C	0	-8.779070	3.769459	-3.944049
206	C	0	-7.459463	4.062601	-4.582353
207	C	0	-7.154394	5.028227	-5.508425
208	C	0	-6.237745	3.329226	-4.329505
209	C	0	-5.230528	3.897482	-5.154350
210	C	0	-5.913532	2.236642	-3.505225
211	C	0	-3.921210	3.397149	-5.177736
212	C	0	-4.614494	1.743645	-3.517493
213	C	0	-3.628075	2.319949	-4.349230
214	H	0	-7.741796	3.806110	-2.051208
215	H	0	-9.017948	2.704853	-4.062320
216	H	0	-9.587551	4.337332	-4.415203
217	H	0	-7.791368	5.775775	-5.953576
218	H	0	-6.671192	1.770990	-2.883098
219	H	0	-3.165576	3.830249	-5.823554
220	H	0	-4.352339	0.899163	-2.889973
221	H	0	-2.624514	1.910699	-4.344539
222	C	0	-5.242839	5.583430	0.465977
223	C	0	-5.144675	7.041612	0.079065
224	O	0	-4.067699	7.683496	0.142747
225	C	0	-5.268986	4.739933	-0.824916
226	C	0	-3.882268	4.669745	-1.452461
227	O	0	-3.391565	2.951314	0.198130
228	H	0	-4.327442	5.363913	1.028936
229	H	0	-5.603161	3.730893	-0.560586
230	H	0	-5.986946	5.170439	-1.531846
231	H	0	-3.461897	5.666234	-1.618701
232	H	0	-3.926439	4.170017	-2.429517
233	C	0	-6.181967	9.064174	-0.772423
234	O	0	-7.283842	11.208039	-0.471993
235	C	0	-6.207313	9.193528	-2.324776
236	C	0	-4.950003	8.659252	-2.962887
237	C	0	-4.882896	7.352237	-3.461990

238	C	0	-3.813996	9.476518	-3.043543
239	C	0	-3.694797	6.877924	-4.027827
240	C	0	-2.630181	9.003838	-3.607478
241	C	0	-2.567515	7.698285	-4.101540
242	H	0	-5.222429	9.446516	-0.411697
243	H	0	-6.327893	10.252831	-2.570184
244	H	0	-7.084709	8.650778	-2.690758
245	H	0	-5.760799	6.710906	-3.405573
246	H	0	-3.856750	10.487464	-2.648736
247	H	0	-3.646006	5.860476	-4.397017
248	H	0	-1.758861	9.647006	-3.649310
249	H	0	-1.649320	7.323523	-4.539854
250	C	0	-0.928002	7.533843	-0.371956
251	O	0	0.599640	9.375956	-0.005039
252	H	0	-0.022204	6.931846	-0.474746
253	H	0	-1.601931	7.312421	-1.201974
254	C	0	3.764173	9.112379	2.087890
255	C	0	4.280379	9.668166	0.756446
256	O	0	5.269866	9.118318	0.212824
257	C	0	3.723557	7.589815	1.954147
258	H	0	4.479738	9.391562	2.866382
259	H	0	4.701594	7.197080	1.679285
260	H	0	3.380919	7.147912	2.895607
261	C	0	4.145653	11.255523	-1.008844
262	O	0	3.469135	13.516328	-0.486623
263	C	0	3.138470	10.930513	-2.118737
264	H	0	5.103662	10.789544	-1.229868
265	H	0	3.092293	9.843699	-2.242562
266	H	0	2.149742	11.295046	-1.816909
267	Fe	0	-1.464154	2.592107	0.822165
268	C	0	-0.991549	-3.165834	1.294560
269	C	0	-0.837232	-2.407641	2.463806
270	C	0	-1.631321	-2.602646	0.185204

271	C	0	-1.308118	-1.090226	2.525586
272	C	0	-2.081837	-1.285162	0.236155
273	C	0	-1.905674	-0.534535	1.390354
274	O	0	-2.290514	0.802932	1.417263
275	H	0	-0.399822	-2.854793	3.352484
276	H	0	-1.792440	-3.185494	-0.714760
277	H	0	-2.562700	-0.823102	-0.618259
278	H	0	-3.256091	0.946436	1.639723
279	N	0	12.288294	1.040380	-0.826278
280	H	0	11.368625	0.991376	-0.400184
281	H	0	12.911440	1.659300	-0.315187
282	C	0	13.717568	1.574386	-2.726684
283	O	0	13.828048	1.535303	-4.077157
284	H	0	14.728033	1.758054	-4.394976
285	C	0	4.318515	12.751130	-0.943836
286	O	0	5.500894	13.166454	-1.450619
287	H	0	5.594006	14.142357	-1.447622
288	N	0	2.444106	9.654887	2.439577
289	H	0	1.677118	9.508844	1.791419
290	H	0	2.191523	9.597427	3.415099
291	C	0	-0.520137	8.994516	-0.402811
292	O	0	-1.481556	9.863607	-0.764304
293	H	0	-1.202666	10.800364	-0.678544
294	N	0	-1.640157	7.264303	0.885364
295	H	0	-2.639182	7.433351	0.843340
296	H	0	-1.143198	7.530016	1.725973
297	N	0	-8.632799	-6.830436	1.852799
298	H	0	-8.788237	-7.286640	0.959786
299	N	0	-6.873735	-8.680441	0.732495
300	H	0	-6.508014	-8.737783	-0.212086
301	H	0	-6.903547	-9.569645	1.214922
302	C	0	-10.112510	-7.362619	3.699571
303	O	0	-9.839269	-8.645331	3.347789

304	H	0	-10.109308	-9.285254	4.038687
305	O	0	-6.475804	-11.516392	-3.087323
306	H	0	-6.828608	-12.424424	-3.106495
307	N	0	-4.463167	-9.756445	-3.519841
308	H	0	-4.339426	-10.626315	-3.016042
309	N	0	-1.407774	-7.772539	-2.778760
310	H	0	-1.643099	-6.955423	-2.230344
311	N	0	1.668127	-6.305660	-2.986756
312	H	0	2.036370	-5.366862	-2.864233
313	N	0	4.064508	-8.163486	-1.232691
314	H	0	4.129189	-9.083550	-1.654219
315	H	0	4.822605	-7.937174	-0.598327
316	C	0	-6.625706	-8.651216	-3.346721
317	O	0	-7.761339	-8.183639	-3.929872
318	H	0	-7.887920	-8.414572	-4.870510
319	O	0	-4.812216	0.905775	2.317229
320	H	0	-4.762847	0.868432	3.291215
321	N	0	-12.042050	2.348405	-0.459197
322	H	0	-12.597370	1.506473	-0.356825
323	H	0	-12.592004	3.185453	-0.303851
324	N	0	-5.816323	4.933826	-5.858347
325	H	0	-5.347799	5.535854	-6.515938
326	N	0	-9.692600	3.243442	-1.707852
327	H	0	-10.467983	2.791039	-2.182132
328	O	0	3.611585	11.596338	-3.303424
329	H	0	2.992375	11.487230	-4.047261
330	O	0	2.818992	7.214361	0.875539
331	H	0	2.066381	7.836061	0.765661
332	N	0	3.658177	10.722940	0.242986
333	H	0	2.869526	11.133045	0.730309
334	N	0	2.037854	4.623568	0.967862
335	H	0	2.413605	5.588482	0.905679
336	N	0	6.125422	3.814446	2.805110

337	H	0	6.652435	3.639999	1.958390
338	H	0	6.610937	4.418465	3.455662
339	N	0	4.142332	2.230908	4.101291
340	H	0	5.054997	1.875853	3.832526
341	C	0	3.625934	2.202884	6.493489
342	O	0	4.942468	2.390057	6.742646
343	H	0	5.114694	2.803920	7.614095
344	C	0	2.304305	-0.963311	3.550895
345	O	0	2.165184	-1.511572	2.321842
346	N	0	-2.758965	3.200284	4.659424
347	H	0	-3.500708	3.272840	5.340549
348	N	0	0.486924	4.664595	6.568355
349	H	0	0.667186	5.559289	7.010714
350	H	0	1.323500	4.086430	6.525119
351	C	0	-0.727404	3.807806	8.573776
352	O	0	-1.842004	3.135438	8.984984
353	H	0	-1.902285	3.076626	9.960533
354	O	0	6.704671	-4.360919	-2.491515
355	H	0	6.071361	-5.079053	-2.315289
356	N	0	10.395707	-4.528034	2.602987
357	H	0	10.618032	-5.447952	2.976936
358	H	0	9.521921	-4.561546	2.082125
359	C	0	11.424107	-2.511708	1.461137
360	O	0	12.447540	-2.110302	0.657919
361	H	0	12.390171	-1.167832	0.361663
362	C	0	4.480028	-8.977401	1.833491
363	O	0	4.648291	-10.081682	1.060122
364	H	0	5.321013	-9.967249	0.357542
365	N	0	4.036643	-8.851337	4.248317
366	H	0	3.335120	-8.810531	4.979764
367	H	0	4.841263	-9.397131	4.535603
368	N	0	7.819124	2.332404	-2.300792
369	H	0	7.345758	1.861570	-3.086206

370	C	0	7.184672	3.309168	-1.656573
371	N	0	5.959586	3.690160	-2.053217
372	H	0	5.520204	4.520511	-1.691053
373	H	0	5.462867	3.070928	-2.695403
374	N	0	7.755393	3.942558	-0.607053
375	H	0	7.307011	4.737505	-0.179102
376	H	0	8.687917	3.724620	-0.297804
377	N	0	5.299512	0.970581	-6.239062
378	H	0	6.262296	1.191690	-5.958894
379	H	0	4.746067	1.820941	-6.317750
380	C	0	7.927338	-0.471777	-4.845649
381	O	0	8.713754	-1.156260	-4.108037
382	C	0	5.211290	-0.223437	-0.954885
383	O	0	5.259847	-0.219444	0.343420
384	C	0	6.844354	-4.303706	4.081517
385	O	0	8.000017	-4.813281	3.579432
386	H	0	8.835845	-4.428198	3.937745
387	N	0	4.403139	-4.413903	3.855474
388	H	0	3.911715	-3.611036	3.483013
389	H	0	3.815895	-5.220251	4.004842
390	O	0	5.608178	-2.490411	2.222065
391	H	0	5.569905	-1.857013	1.457729
392	C	0	-2.956370	3.907765	-0.565476
393	N	0	-6.425269	5.265525	1.279604
394	H	0	-7.234042	4.890657	0.790942
395	H	0	-6.646456	5.915592	2.021476
396	N	0	-6.252692	7.663099	-0.365418
397	H	0	-7.107672	7.147201	-0.534373
398	C	0	-7.256903	10.004432	-0.223250
399	O	0	-8.188467	9.382155	0.541456
400	H	0	-8.873376	9.997621	0.877818
401	O	0	-1.688057	4.158086	-0.516436
402	C	0	-9.739177	3.275025	-0.365523

403	C	0	-8.905191	5.526083	-2.151263
404	O	0	-10.191748	5.900952	-1.982191
405	H	0	-10.294558	6.864865	-1.836191
406	C	0	-4.244549	-3.626809	2.467637
407	H	0	-3.353877	-3.463261	3.064096
408	H	0	1.231966	-1.727551	2.087785
409	O	0	-1.224310	-0.320978	3.656340
410	H	0	-0.392120	-0.502174	4.156434
411	C	0	-0.474594	-4.584726	1.238783
412	H	0	-1.314809	-5.284683	1.139918
413	H	0	0.045274	-4.820695	2.171780
414	C	0	0.470390	-4.750903	0.049778
415	H	0	1.328303	-4.080878	0.113202
416	H	0	-0.052138	-4.561529	-0.888663
417	H	0	1.432714	-6.345493	-0.943767
418	H	0	1.702181	-6.338479	0.708744
419	H	0	0.248568	-6.843934	0.097662
420	N	0	1.003741	-6.160512	-0.020442

Tyrosinase: Overall Charge = +3, Total energy = -8954.65100594 Ha

1	C	0	6.196272	2.814470	5.970125
2	O	0	4.297725	2.045925	7.202746
3	C	0	6.360178	2.074842	4.618565
4	C	0	5.032228	1.778210	3.997954
5	N	0	4.371666	0.554541	4.065998
6	C	0	4.159400	2.623297	3.355804
7	C	0	3.156853	0.672707	3.486611
8	N	0	2.997050	1.926710	3.045281
9	H	0	6.940795	1.158935	4.767680
10	H	0	6.938354	2.731364	3.963814
11	H	0	4.718729	-0.310268	4.464841
12	H	0	4.282058	3.663424	3.105531
13	H	0	2.456056	-0.137964	3.391676
14	H	0	5.530207	3.666046	5.810292
15	C	0	-3.574961	4.587611	5.089671
16	O	0	-4.968206	5.224846	6.886741
17	C	0	-3.284707	3.056783	5.127456
18	C	0	-1.900804	2.740629	4.669433
19	N	0	-0.850922	2.551759	5.558126
20	C	0	-1.343170	2.625509	3.421006
21	C	0	0.290746	2.341482	4.869550
22	N	0	0.026189	2.379226	3.558965
23	H	0	-3.619569	4.900097	4.044007
24	H	0	-3.414137	2.703134	6.156441
25	H	0	-4.020914	2.534386	4.509650
26	H	0	-0.923957	2.602274	6.564411
27	H	0	-1.812731	2.727595	2.455780
28	H	0	1.256635	2.187787	5.319280
29	C	0	2.727519	8.803285	-0.099818
30	O	0	4.514691	9.407811	-1.540777

31	C	0	2.613207	7.400755	-0.741479
32	C	0	2.221414	6.345980	0.245489
33	N	0	1.440679	6.643526	1.352567
34	C	0	2.460465	4.992962	0.311450
35	C	0	1.223224	5.518358	2.058565
36	N	0	1.827904	4.487583	1.448819
37	H	0	3.402180	8.739067	0.761725
38	H	0	1.875621	7.453284	-1.553551
39	H	0	3.571668	7.138383	-1.193748
40	H	0	1.098966	7.588494	1.525636
41	H	0	3.014187	4.355415	-0.359636
42	H	0	0.645864	5.470584	2.966025
43	C	0	-12.014418	0.783833	-0.146016
44	O	0	-14.370923	0.360352	0.072489
45	C	0	-11.791434	1.329993	1.274998
46	C	0	-10.388527	1.049239	1.766903
47	C	0	-10.011106	-0.253989	2.120443
48	C	0	-9.442068	2.075797	1.876913
49	C	0	-8.719587	-0.525551	2.574141
50	C	0	-8.148045	1.809840	2.338656
51	C	0	-7.782709	0.506126	2.686701
52	H	0	-11.839882	-0.298863	-0.131781
53	H	0	-12.530015	0.866536	1.936366
54	H	0	-11.981121	2.409935	1.275025
55	H	0	-10.736925	-1.058825	2.047299
56	H	0	-9.722538	3.090892	1.613375
57	H	0	-8.449102	-1.539067	2.847575
58	H	0	-7.433574	2.619708	2.436491
59	H	0	-6.780232	0.297642	3.042759
60	C	0	-4.663913	1.110821	-5.182840
61	C	0	-4.523625	-0.188503	-4.387564
62	O	0	-3.520373	-0.875595	-4.515275
63	C	0	-4.553881	2.341669	-4.246211

64	C	0	-3.165837	2.511999	-3.739757
65	N	0	-2.576114	1.636635	-2.839455
66	C	0	-2.159006	3.365402	-4.123855
67	C	0	-1.257813	1.968101	-2.716410
68	N	0	-0.970815	3.016719	-3.483462
69	H	0	-3.776654	1.111158	-5.831478
70	H	0	-4.815797	3.231377	-4.826699
71	H	0	-5.285703	2.255408	-3.435558
72	H	0	-3.028627	0.866060	-2.367936
73	H	0	-2.211230	4.184237	-4.820452
74	H	0	-0.563637	1.360782	-2.158008
75	N	0	-5.522688	-0.511309	-3.567957
76	C	0	-5.517777	-1.763536	-2.803193
77	O	0	-4.640710	-3.909867	-3.484729
78	C	0	-6.803029	-1.780824	-1.929817
79	C	0	-6.763798	-0.731898	-0.815134
80	O	0	-5.752302	-0.702867	-0.045962
81	H	0	-6.389202	0.008624	-3.658269
82	H	0	-4.626427	-1.804323	-2.172268
83	H	0	-6.885538	-2.759189	-1.443293
84	H	0	-7.679863	-1.658214	-2.571609
85	C	0	-1.263992	-4.489218	-3.949958
86	O	0	-0.718277	-6.794078	-3.960892
87	C	0	-1.378374	-3.888024	-2.515568
88	C	0	-0.534076	-2.665854	-2.390133
89	N	0	0.787451	-2.729676	-1.975086
90	C	0	-0.755391	-1.341999	-2.703204
91	C	0	1.310131	-1.470727	-2.031900
92	N	0	0.401702	-0.599574	-2.469325
93	H	0	-0.213362	-4.461877	-4.249573
94	H	0	-1.048606	-4.625702	-1.775541
95	H	0	-2.434449	-3.660534	-2.339923
96	H	0	1.245441	-3.602986	-1.698573

97	H	0	-1.656817	-0.901532	-3.099413
98	H	0	2.324973	-1.230914	-1.759689
99	C	0	3.330476	-6.210320	-1.892371
100	C	0	1.910192	-6.347618	-1.377199
101	O	0	1.158198	-5.374033	-1.319930
102	C	0	3.346668	-6.050735	-3.420686
103	C	0	4.476098	-5.119524	-3.859001
104	S	0	4.143134	-3.323982	-3.396965
105	C	0	2.856428	-2.919398	-4.692809
106	H	0	3.661939	-5.246058	-1.477958
107	H	0	2.382212	-5.658930	-3.763215
108	H	0	3.468036	-7.036569	-3.887506
109	H	0	5.411259	-5.371598	-3.352653
110	H	0	4.637557	-5.154633	-4.937895
111	H	0	3.291866	-3.019261	-5.687131
112	H	0	2.545802	-1.888763	-4.520217
113	H	0	1.989066	-3.571488	-4.585428
114	N	0	1.552276	-7.561555	-0.994717
115	C	0	0.227569	-7.854053	-0.503035
116	C	0	-0.013863	-7.454844	0.947864
117	O	0	-1.166592	-7.463575	1.382553
118	H	0	2.278550	-8.271502	-1.042757
119	H	0	0.036374	-8.925210	-0.592935
120	H	0	-0.517386	-7.325498	-1.103350
121	N	0	1.019811	-7.124931	1.695309
122	C	0	0.818979	-6.708295	3.069918
123	C	0	1.454229	-5.373640	3.437920
124	O	0	2.586962	-5.107706	3.113718
125	C	0	1.306876	-7.798072	4.072018
126	C	0	0.457117	-9.063921	3.917771
127	C	0	2.796794	-8.120521	3.905711
128	H	0	1.955681	-7.108219	1.310968
129	H	0	-0.264556	-6.623941	3.189201

130	H	0	1.147661	-7.384732	5.078083
131	H	0	0.591308	-9.490473	2.917357
132	H	0	-0.606460	-8.852210	4.059697
133	H	0	0.763230	-9.817583	4.649108
134	H	0	3.422003	-7.231983	4.019335
135	H	0	2.986921	-8.556945	2.918336
136	H	0	3.101204	-8.857394	4.654797
137	N	0	0.689316	-4.561535	4.152193
138	C	0	1.143860	-3.236562	4.571009
139	O	0	3.432096	-2.530228	4.556730
140	C	0	0.079054	-2.566445	5.465626
141	C	0	0.600521	-1.226712	5.997764
142	C	0	-1.224517	-2.348297	4.685420
143	H	0	-0.211288	-4.869976	4.490558
144	H	0	1.247101	-2.615869	3.669853
145	H	0	-0.110573	-3.234888	6.316232
146	H	0	0.812885	-0.544446	5.166400
147	H	0	1.512307	-1.341800	6.592326
148	H	0	-0.155731	-0.753837	6.629488
149	H	0	-1.038115	-1.700656	3.818939
150	H	0	-1.966185	-1.860605	5.323552
151	H	0	-1.662473	-3.282619	4.323164
152	C	0	4.945383	-4.016963	0.896578
153	O	0	6.466380	-3.736103	-0.924811
154	C	0	3.760645	-3.045842	0.774713
155	H	0	4.742766	-4.888395	0.256648
156	H	0	3.593741	-2.770743	-0.272085
157	H	0	3.955968	-2.146836	1.367389
158	H	0	2.857626	-3.530513	1.156907
159	C	0	5.900778	-0.407527	-4.658072
160	O	0	4.949681	1.708994	-5.211397
161	C	0	6.633763	-0.589100	-3.295597
162	C	0	6.327627	0.319644	-2.131231

163	C	0	6.889157	1.601782	-2.056939
164	C	0	5.607327	-0.163981	-1.032107
165	C	0	6.741054	2.376290	-0.905911
166	C	0	5.470141	0.601831	0.127011
167	C	0	6.039747	1.874191	0.192697
168	H	0	4.843062	-0.670010	-4.561361
169	H	0	6.448537	-1.626374	-2.995008
170	H	0	7.704211	-0.507339	-3.525524
171	H	0	7.469973	1.980020	-2.894543
172	H	0	5.174664	-1.160371	-1.088932
173	H	0	7.189242	3.363010	-0.859184
174	H	0	4.929260	0.202004	0.979513
175	H	0	5.943687	2.468355	1.094962
176	C	0	2.652057	4.652616	-6.008096
177	O	0	1.911843	6.828996	-6.717242
178	C	0	3.289541	4.930528	-4.623828
179	C	0	3.058119	3.867590	-3.605464
180	N	0	1.856008	3.243786	-3.306349
181	C	0	3.976509	3.331683	-2.729908
182	C	0	2.095870	2.357952	-2.288944
183	N	0	3.370914	2.389693	-1.907843
184	H	0	1.568681	4.554672	-5.866651
185	H	0	2.901773	5.896008	-4.273295
186	H	0	4.367687	5.047026	-4.743720
187	H	0	0.903731	3.396750	-3.666327
188	H	0	5.029471	3.544044	-2.667459
189	H	0	1.355411	1.679639	-1.902547
190	Cu	0	1.401933	2.661436	2.153417
191	C	0	-2.085801	-1.278261	0.571496
192	C	0	-1.905584	1.118896	0.387806
193	C	0	-0.703019	-1.364255	0.753903
194	C	0	-0.527394	1.014269	0.568576
195	O	0	0.221931	2.208179	0.594584

196	H	0	-0.233971	-2.334206	0.879400
197	H	0	-0.172599	2.918309	0.045989
198	N	0	1.437898	9.275531	0.367232
199	H	0	1.402470	9.984370	1.085901
200	H	0	0.639850	9.221198	-0.251957
201	C	0	3.436472	9.739332	-1.093516
202	O	0	2.845757	10.910962	-1.415989
203	H	0	1.981318	11.026460	-0.968173
204	C	0	2.765712	5.954434	-6.819926
205	O	0	4.020374	6.548430	-6.479387
206	H	0	3.984824	7.508825	-6.669596
207	N	0	3.060140	3.438976	-6.730297
208	H	0	2.646376	2.586710	-6.373961
209	H	0	4.070249	3.311991	-6.727129
210	N	0	6.677788	-1.255635	-5.573649
211	H	0	7.016087	-2.107708	-5.139168
212	H	0	6.222576	-1.452361	-6.459554
213	C	0	5.968651	1.021492	-5.140072
214	O	0	7.185442	1.447551	-5.555520
215	H	0	7.777606	0.663218	-5.662138
216	C	0	2.572210	-3.193838	5.124969
217	O	0	2.972462	-4.564417	5.197312
218	H	0	3.947588	-4.621574	5.110563
219	N	0	4.196670	-7.303427	-1.427962
220	H	0	4.764404	-7.745542	-2.139030
221	H	0	4.704801	-7.127870	-0.570769
222	N	0	7.461362	3.309163	6.463724
223	H	0	8.125188	2.584364	6.714438
224	H	0	7.384071	4.021358	7.179093
225	C	0	5.456487	1.903186	6.935477
226	O	0	6.245708	0.903339	7.421591
227	H	0	5.778231	0.306127	8.041736
228	C	0	-4.913527	4.812017	5.757966

229	O	0	-6.271931	4.676430	5.332996
230	H	0	-6.806847	4.928855	6.112713
231	N	0	-2.508206	5.342110	5.762993
232	H	0	-2.348189	6.259909	5.363561
233	H	0	-2.608319	5.366925	6.772257
234	N	0	5.117990	-4.393316	2.307056
235	H	0	4.224325	-4.509409	2.768282
236	H	0	5.736465	-5.184829	2.444245
237	C	0	6.152862	-3.371684	0.193851
238	O	0	6.754676	-2.367870	0.874870
239	H	0	7.437474	-1.903833	0.345284
240	N	0	-11.081320	1.406012	-1.096146
241	H	0	-11.324900	1.253628	-2.068860
242	H	0	-10.936475	2.395774	-0.919082
243	C	0	-13.485171	0.938640	-0.559794
244	O	0	-13.709732	1.713389	-1.644476
245	H	0	-14.659866	1.768589	-1.878771
246	N	0	-5.917110	1.225551	-5.943586
247	H	0	-6.138948	2.156917	-6.266626
248	H	0	-6.039827	0.527886	-6.667317
249	C	0	-5.444129	-2.997608	-3.721493
250	O	0	-6.312806	-2.966122	-4.745969
251	H	0	-6.267316	-3.753053	-5.329263
252	N	0	-7.795875	0.103295	-0.650021
253	H	0	-7.773617	0.840087	0.047643
254	H	0	-8.629706	0.083980	-1.227306
255	N	0	-2.071656	-3.810264	-4.955360
256	H	0	-1.834344	-2.824711	-5.043980
257	H	0	-3.063534	-3.918316	-4.757729
258	C	0	-1.605390	-5.967669	-3.899056
259	O	0	-2.917607	-6.263806	-3.761370
260	H	0	-3.084587	-7.229298	-3.725426
261	C	0	-2.690826	-0.025131	0.378495

262	H	0	-3.768072	0.047559	0.241593
263	C	0	0.088577	-0.211569	0.738600
264	H	0	1.167554	-0.275090	0.823620
265	O	0	-2.364984	2.415663	0.240611
266	H	0	-3.316706	2.483890	0.041920
267	C	0	-2.951078	-2.516586	0.627264
268	H	0	-2.335905	-3.407633	0.480865
269	H	0	-3.673112	-2.490978	-0.197509
270	C	0	-3.716042	-2.625502	1.965182
271	H	0	-3.541750	-1.748678	2.590151
272	H	0	-3.434482	-3.516602	2.524973
273	H	0	-5.468864	-3.566524	1.266565
274	H	0	-5.718547	-2.640184	2.614010
275	H	0	-5.499355	-1.893451	1.112544
276	N	0	-5.204580	-2.691634	1.730107

DOPA decarboxylase: Overall Charge = 0, Total energy = -7659.07746958 Ha

1	N	0	8.251287	-7.453492	-1.555461
2	C	0	7.425374	-6.472182	-2.239295
3	C	0	7.998810	-6.048275	-3.580558
4	O	0	7.281300	-5.562784	-4.452545
5	C	0	7.387822	-5.176489	-1.377567
6	C	0	6.418606	-5.213735	-0.240469
7	C	0	6.592532	-5.697168	1.034922
8	C	0	5.097121	-4.632255	-0.284202
9	N	0	5.450219	-5.456184	1.785089
10	C	0	4.525271	-4.780020	1.005858
11	C	0	4.374748	-3.960504	-1.286238
12	C	0	3.279195	-4.227236	1.335214
13	C	0	3.141716	-3.407955	-0.968418
14	C	0	2.607547	-3.530907	0.335506

15	H	0	8.325770	-8.347242	-2.029109
16	H	0	7.984502	-7.574844	-0.583441
17	H	0	6.393550	-6.788436	-2.448314
18	H	0	7.123869	-4.331959	-2.024152
19	H	0	8.411655	-5.012254	-1.025536
20	H	0	7.453900	-6.166197	1.484098
21	H	0	5.343536	-5.672527	2.763636
22	H	0	4.779670	-3.876573	-2.291205
23	H	0	2.880105	-4.313781	2.341436
24	H	0	2.576334	-2.865485	-1.717351
25	H	0	1.655092	-3.059184	0.554817
26	N	0	-5.218008	-3.947037	-4.147313
27	C	0	-3.903032	-3.560269	-3.690717
28	C	0	-3.101337	-2.697081	-4.677086
29	O	0	-3.508425	-2.432567	-5.829683
30	C	0	-3.098107	-4.828133	-3.320315
31	C	0	-3.348581	-5.356279	-1.920614
32	C	0	-2.386441	-6.186085	-1.322841
33	C	0	-4.495483	-5.032510	-1.178481
34	C	0	-2.554920	-6.679494	-0.028569
35	C	0	-4.667852	-5.508374	0.120335
36	C	0	-3.697151	-6.330938	0.693572
37	O	0	-3.917424	-6.757190	1.994229
38	H	0	-5.193993	-4.622004	-4.906369
39	H	0	-5.841740	-3.180619	-4.383303
40	H	0	-4.012910	-2.944207	-2.785088
41	H	0	-3.342929	-5.602393	-4.060424
42	H	0	-2.024253	-4.640182	-3.430367
43	H	0	-1.490202	-6.448424	-1.876803
44	H	0	-5.262389	-4.415868	-1.633086
45	H	0	-1.799187	-7.322190	0.411512
46	H	0	-5.548511	-5.255469	0.698063
47	H	0	-3.236618	-7.378222	2.313069

48	N	0	-1.927451	-2.247761	-4.169373
49	C	0	-1.020390	-1.377931	-4.884633
50	C	0	0.402782	-1.827730	-4.611946
51	O	0	0.663994	-2.494024	-3.566086
52	C	0	-1.143964	0.085328	-4.354504
53	C	0	-2.535662	0.618254	-4.579319
54	C	0	-3.514005	0.521673	-3.581335
55	C	0	-2.886905	1.156690	-5.825407
56	C	0	-4.824818	0.938077	-3.835804
57	C	0	-4.192046	1.577675	-6.078850
58	C	0	-5.167307	1.462811	-5.083872
59	H	0	-1.655111	-2.498452	-3.215087
60	H	0	-1.269595	-1.413343	-5.949216
61	H	0	-0.896964	0.061070	-3.284974
62	H	0	-0.392150	0.714603	-4.843673
63	H	0	-3.251007	0.126889	-2.603472
64	H	0	-2.131183	1.240689	-6.601523
65	H	0	-5.566138	0.863088	-3.047980
66	H	0	-4.448133	1.992879	-7.046900
67	H	0	-6.183206	1.791097	-5.277457
68	N	0	1.385308	-1.416867	-5.419434
69	C	0	2.770847	-1.539467	-4.971454
70	C	0	3.095211	-0.421678	-3.979533
71	O	0	2.410487	0.619172	-3.903136
72	C	0	3.569690	-1.311981	-6.265637
73	C	0	2.702180	-0.305083	-7.040094
74	C	0	1.263166	-0.753356	-6.739699
75	H	0	2.948645	-2.518084	-4.521027
76	H	0	3.646099	-2.257872	-6.808977
77	H	0	4.579611	-0.944218	-6.069907
78	H	0	2.869461	0.704941	-6.655958
79	H	0	2.912357	-0.307904	-8.109942
80	H	0	0.898582	-1.474585	-7.477009

81	H	0	0.570144	0.089439	-6.694838
82	N	0	4.256758	-0.605043	-3.290188
83	C	0	4.976370	0.512213	-2.716693
84	C	0	6.210540	0.745519	-3.535715
85	O	0	6.792455	-0.015006	-4.307832
86	C	0	5.315446	0.336439	-1.210263
87	O	0	5.882567	1.549587	-0.676313
88	C	0	6.306357	-0.787505	-0.958070
89	H	0	4.767852	-1.470060	-3.419746
90	H	0	4.352886	1.404175	-2.807683
91	H	0	4.357452	0.113113	-0.720451
92	H	0	5.590353	2.356860	-1.163905
93	H	0	5.891900	-1.754127	-1.259357
94	H	0	6.538165	-0.837523	0.108609
95	H	0	7.240713	-0.606646	-1.499380
96	N	0	-11.428600	2.321983	1.041005
97	C	0	-10.318755	1.873477	1.866705
98	C	0	-10.672346	2.168395	3.300665
99	O	0	-11.291094	3.132530	3.749050
100	C	0	-8.959831	2.510810	1.456155
101	C	0	-7.771664	2.084882	2.251274
102	N	0	-7.076227	2.948432	3.085661
103	C	0	-7.111478	0.880988	2.347623
104	C	0	-6.034568	2.270498	3.643898
105	N	0	-6.031526	1.006395	3.217103
106	H	0	-11.390255	1.994589	0.083507
107	H	0	-11.616743	3.316591	1.106631
108	H	0	-10.226699	0.788384	1.782647
109	H	0	-9.064889	3.601616	1.482271
110	H	0	-8.813365	2.224601	0.409196
111	H	0	-7.291523	3.923725	3.232285
112	H	0	-7.329213	-0.042655	1.839137
113	H	0	-5.293478	2.708986	4.295889

114	N	0	-9.148257	0.241976	-3.997735
115	C	0	-8.310443	-0.918827	-3.758762
116	C	0	-8.819060	-2.196633	-4.392139
117	O	0	-8.047262	-2.969028	-4.961862
118	C	0	-8.131630	-1.082883	-2.218409
119	O	0	-8.032513	0.273742	-1.716452
120	C	0	-6.888046	-1.868022	-1.853108
121	H	0	-8.923745	0.766818	-4.834580
122	H	0	-10.146629	0.064926	-3.922673
123	H	0	-7.326545	-0.742436	-4.201744
124	H	0	-9.031555	-1.544479	-1.795750
125	H	0	-8.560532	0.817896	-2.373562
126	H	0	-6.923774	-2.869018	-2.296444
127	H	0	-6.799449	-1.968881	-0.768963
128	H	0	-5.996102	-1.352919	-2.227783
129	N	0	4.519483	7.297630	-2.518986
130	C	0	5.145787	6.085999	-2.011804
131	C	0	4.477636	4.800048	-2.455359
132	O	0	4.840514	3.703918	-2.028837
133	C	0	5.164578	6.012438	-0.461467
134	C	0	3.775466	5.931253	0.090549
135	N	0	3.092178	6.999373	0.658291
136	C	0	2.860825	4.902081	0.060035
137	C	0	1.819902	6.600554	0.946186
138	N	0	1.646514	5.330787	0.584870
139	H	0	5.147832	7.987221	-2.904595
140	H	0	3.796441	7.698855	-1.933519
141	H	0	6.179361	6.041494	-2.370417
142	H	0	5.705513	6.873989	-0.060601
143	H	0	5.709950	5.105158	-0.197055
144	H	0	3.479839	7.910786	0.855593
145	H	0	2.997955	3.899563	-0.308276
146	H	0	1.081863	7.241040	1.394473

147	N	0	3.495123	4.931117	-3.330450
148	C	0	2.900182	3.745708	-3.903701
149	C	0	3.438364	3.621068	-5.319510
150	O	0	4.294291	2.789515	-5.602731
151	C	0	1.368802	3.730524	-3.847177
152	C	0	0.880377	3.610046	-2.400837
153	C	0	-0.637428	3.438019	-2.295038
154	C	0	-1.120739	3.163841	-0.873578
155	N	0	-0.923662	4.335671	0.048296
156	H	0	3.265010	5.871391	-3.641071
157	H	0	3.281847	2.875018	-3.379787
158	H	0	1.026094	2.863180	-4.423644
159	H	0	0.964837	4.631003	-4.325216
160	H	0	1.370221	2.740802	-1.937449
161	H	0	1.195897	4.498065	-1.841528
162	H	0	-0.955580	2.590562	-2.915117
163	H	0	-1.148777	4.323529	-2.695738
164	H	0	-2.187676	2.929858	-0.872419
165	H	0	-0.593210	2.314987	-0.429724
166	H	0	0.093697	4.586665	0.246103
167	H	0	-1.394826	4.110653	0.970943
168	H	0	-1.368633	5.172745	-0.339252
169	N	0	8.481265	4.677035	0.675145
170	C	0	7.548260	4.911558	1.739505
171	C	0	8.061956	5.767949	2.828211
172	O	0	9.186095	5.625839	3.323194
173	C	0	7.142007	3.542803	2.358851
174	C	0	5.883162	3.636850	3.183214
175	C	0	5.915159	3.874063	4.561451
176	C	0	4.642377	3.484306	2.548346
177	C	0	4.725866	3.955462	5.292656
178	C	0	3.453611	3.566514	3.272158
179	C	0	3.494888	3.801973	4.650545

180	H	0	8.280493	3.859220	0.115370
181	H	0	8.752674	5.482032	0.125551
182	H	0	6.612266	5.382514	1.417101
183	H	0	7.987221	3.157683	2.938652
184	H	0	6.967500	2.860337	1.517578
185	H	0	6.871550	3.983490	5.065395
186	H	0	4.622596	3.263044	1.484013
187	H	0	4.761980	4.133532	6.361441
188	H	0	2.501143	3.438453	2.769012
189	H	0	2.572285	3.859049	5.217386
190	N	0	5.904839	-2.814547	3.489458
191	C	0	4.955550	-1.986511	4.238483
192	C	0	4.291267	-2.997467	5.185170
193	O	0	3.430765	-3.771233	4.762310
194	C	0	3.858802	-1.279482	3.418544
195	C	0	4.445715	-0.565255	2.189385
196	C	0	3.061514	-0.326758	4.319759
197	C	0	5.665690	0.309983	2.491886
198	H	0	5.512014	-3.256438	2.665909
199	H	0	6.803154	-2.385976	3.309242
200	H	0	5.515572	-1.258855	4.832420
201	H	0	3.176723	-2.058417	3.052029
202	H	0	4.708493	-1.318684	1.433915
203	H	0	3.651638	0.051041	1.746160
204	H	0	3.680419	0.511854	4.658641
205	H	0	2.680335	-0.841437	5.211469
206	H	0	2.200622	0.082809	3.781759
207	H	0	6.545223	-0.299466	2.724444
208	H	0	5.480752	0.974657	3.341369
209	H	0	5.905643	0.932525	1.624859
210	N	0	1.819296	-3.475946	7.124974
211	C	0	0.469717	-3.193809	7.594639
212	C	0	-0.236157	-4.448279	8.100451

213	O	0	-1.311241	-4.372208	8.672490
214	C	0	-0.462635	-2.479226	6.580768
215	C	0	-0.754759	-3.301380	5.349626
216	C	0	-1.921947	-4.076824	5.278713
217	C	0	0.126941	-3.301876	4.258406
218	C	0	-2.211981	-4.826016	4.135897
219	C	0	-0.159371	-4.054336	3.118902
220	C	0	-1.330575	-4.811303	3.052531
221	H	0	1.858091	-4.050844	6.286428
222	H	0	2.338566	-2.612709	6.989093
223	H	0	0.549138	-2.548972	8.476309
224	H	0	-1.394414	-2.227697	7.097842
225	H	0	0.028117	-1.538715	6.305350
226	H	0	-2.611090	-4.075384	6.118518
227	H	0	1.030789	-2.699713	4.295054
228	H	0	-3.127916	-5.403288	4.076845
229	H	0	0.514096	-4.029082	2.268945
230	H	0	-1.563645	-5.367293	2.151514
231	N	0	-6.274509	4.718894	-0.394295
232	C	0	-6.432395	3.464279	-0.850620
233	C	0	-7.436317	3.260796	-1.950047
234	C	0	-5.646812	2.406073	-0.344297
235	O	0	-5.839147	1.115560	-0.728987
236	C	0	-4.636183	2.682034	0.597848
237	C	0	-4.518619	3.994781	1.088893
238	C	0	-5.360058	4.972313	0.556897
239	C	0	-3.600902	4.412376	2.207172
240	O	0	-2.360553	3.668345	2.213805
241	15	0	-1.743229	3.249242	3.848233
242	O	0	-1.327330	4.649280	4.512129
243	O	0	-3.091903	2.600821	4.510938
244	O	0	-0.572011	2.199939	3.514956
245	H	0	-8.382133	2.848323	-1.580677

246	H	0	-7.054015	2.593232	-2.728800
247	H	0	-7.656490	4.229912	-2.398192
248	H	0	-6.745938	0.865306	-1.108659
249	H	0	-5.285339	6.000288	0.893469
250	H	0	-3.385023	5.482605	2.124426
251	H	0	-4.106307	4.239660	3.164722
252	C	0	-1.923065	-1.350195	1.934149
253	C	0	-0.822758	-0.488060	1.925184
254	C	0	-2.096909	-2.228412	0.856012
255	C	0	-1.224270	-2.205018	-0.221627
256	C	0	-0.140469	-1.319157	-0.244435
257	O	0	0.733079	-1.250725	-1.303872
258	H	0	-0.658140	0.245111	2.706829
259	H	0	-2.942211	-2.914613	0.839525
260	O	0	9.339545	-6.184096	-3.715247
261	H	0	9.653649	-5.855289	-4.584011
262	O	0	6.731387	2.058018	-3.310063
263	H	0	7.554106	2.177512	-3.830714
264	O	0	7.248102	6.818168	3.356585
265	H	0	7.731557	7.236312	4.099121
266	O	0	2.946832	4.482637	-6.349438
267	H	0	3.419458	4.281511	-7.184405
268	O	0	-10.210659	-2.519327	-4.333135
269	H	0	-10.364078	-3.371080	-4.793663
270	O	0	-10.275815	1.115628	4.183334
271	H	0	-10.507470	1.348643	5.107075
272	O	0	0.377220	-5.723749	7.897150
273	H	0	-0.211873	-6.418319	8.260402
274	O	0	4.707928	-2.986853	6.471807
275	H	0	4.242441	-3.657617	7.024603
276	C	0	-3.718626	1.589218	1.031582
277	H	0	-3.491408	1.533335	2.103228
278	O	0	-3.218833	0.775400	0.234295

279	C	0	0.063346	-0.484963	0.847210
280	H	0	0.914424	0.187634	0.825204
281	H	0	0.547592	-1.884692	-2.046301
282	C	0	-3.019243	-1.284471	2.979575
283	H	0	-3.332808	-2.300186	3.250365
284	H	0	-3.889484	-0.806614	2.504200
285	C	0	-2.657366	-0.496477	4.239730
286	H	0	-2.235101	-1.153093	5.000448
287	H	0	-1.927910	0.287485	4.023246
288	O	0	-1.390691	-3.023060	-1.346844
289	H	0	-1.915459	-3.825465	-1.146680
290	H	0	-3.537289	1.293650	4.824653
291	H	0	-4.682789	0.195648	4.191363
292	H	0	-4.099548	-0.114048	5.745952
293	N	0	-3.844093	0.221356	4.818501

Monoamine Oxidase: Overall Charge = -1, Total energy = -12614.9174460 Ha

1	N	0	5.169485	0.913997	7.462559
2	C	0	4.056769	0.187744	8.030346
3	C	0	4.358411	-1.305608	8.013833
4	O	0	4.771880	-1.915525	7.020677
5	C	0	2.748299	0.395401	7.220539
6	C	0	2.313873	1.844911	7.135824
7	C	0	1.938165	2.553553	8.280416
8	C	0	2.360222	2.522972	5.926107
9	C	0	1.596793	3.891719	8.214321
10	C	0	2.013218	3.864040	5.852091
11	C	0	1.635817	4.536301	6.998906
12	O	0	1.312689	5.863061	6.936891
13	H	0	5.019073	1.304700	6.539956
14	H	0	5.706728	1.484761	8.096540

15	H	0	3.884536	0.475256	9.071779
16	H	0	1.977644	-0.225613	7.688920
17	H	0	2.912879	0.006749	6.207759
18	H	0	1.903276	2.041288	9.238091
19	H	0	2.703460	2.010452	5.036556
20	H	0	1.304348	4.427702	9.111397
21	H	0	2.037755	4.390375	4.904919
22	H	0	1.060002	6.233084	7.801003
23	C	0	-12.280612	-1.746695	5.383699
24	C	0	-12.845493	-1.343131	4.017014
25	O	0	-12.104400	-0.817091	3.186841
26	C	0	-11.604412	-3.119124	5.285219
27	C	0	-12.646506	-4.083716	5.736639
28	C	0	-13.366265	-3.365191	6.841876
29	H	0	-11.576209	-0.960234	5.666886
30	H	0	-11.247298	-3.322705	4.278987
31	H	0	-10.736030	-3.139185	5.953599
32	H	0	-12.231026	-5.040178	6.062807
33	H	0	-13.343602	-4.296025	4.914503
34	H	0	-12.833656	-3.482961	7.796185
35	H	0	-14.391343	-3.716343	6.983628
36	N	0	-14.141651	-1.578406	3.802654
37	C	0	-14.772113	-1.410214	2.485904
38	C	0	-15.589501	-0.135147	2.374226
39	O	0	-16.513680	0.073569	3.165219
40	C	0	-15.692420	-2.600091	2.165548
41	C	0	-15.041928	-3.952182	2.330710
42	C	0	-13.870951	-4.274063	1.642186
43	C	0	-15.614839	-4.908143	3.171185
44	C	0	-13.270806	-5.531240	1.798691
45	C	0	-15.031589	-6.169649	3.326466
46	C	0	-13.854981	-6.481396	2.639517
47	H	0	-14.678760	-1.900781	4.602481

48	H	0	-13.948493	-1.401872	1.772288
49	H	0	-16.051901	-2.470113	1.135822
50	H	0	-16.572805	-2.533578	2.815319
51	H	0	-13.414793	-3.544232	0.979158
52	H	0	-16.525383	-4.665078	3.710244
53	H	0	-12.358815	-5.764262	1.260802
54	H	0	-15.490848	-6.900837	3.981794
55	H	0	-13.396548	-7.456251	2.760174
56	N	0	-15.260068	0.726614	1.388644
57	C	0	-16.083042	1.912945	1.137971
58	C	0	-17.471770	1.471741	0.684768
59	O	0	-17.574539	0.639223	-0.206790
60	C	0	-15.347924	2.620775	-0.013233
61	C	0	-13.966534	2.049216	-0.006537
62	C	0	-14.135463	0.633297	0.444047
63	H	0	-16.159962	2.523238	2.040871
64	H	0	-15.369282	3.704274	0.108204
65	H	0	-15.848891	2.381075	-0.956892
66	H	0	-13.479694	2.122595	-0.981236
67	H	0	-13.337659	2.586123	0.712021
68	H	0	-13.246821	0.249808	0.948479
69	H	0	-14.393806	-0.034635	-0.387318
70	N	0	-13.493371	-7.316000	-6.407900
71	C	0	-12.559647	-7.762388	-5.354361
72	C	0	-12.547617	-9.271165	-5.215868
73	O	0	-11.481415	-9.879393	-5.048041
74	C	0	-12.859818	-7.101376	-3.995855
75	C	0	-13.025999	-5.636133	-4.144758
76	C	0	-14.155590	-4.908433	-3.909768
77	C	0	-12.050647	-4.714488	-4.650912
78	N	0	-13.937314	-3.584046	-4.212493
79	C	0	-12.656118	-3.438687	-4.676002
80	C	0	-10.715562	-4.841547	-5.065737

81	C	0	-11.976177	-2.290252	-5.109063
82	C	0	-10.040349	-3.706094	-5.499131
83	C	0	-10.675201	-2.444789	-5.520315
84	H	0	-14.465919	-7.486307	-6.165432
85	H	0	-13.344388	-6.341960	-6.650030
86	H	0	-11.548894	-7.506877	-5.675158
87	H	0	-12.053600	-7.347066	-3.291904
88	H	0	-13.781749	-7.537599	-3.591012
89	H	0	-15.111851	-5.241612	-3.538248
90	H	0	-14.615994	-2.846354	-4.117169
91	H	0	-10.207444	-5.799914	-5.037493
92	H	0	-12.468019	-1.323754	-5.123714
93	H	0	-9.012646	-3.788881	-5.823423
94	H	0	-10.125590	-1.581182	-5.880453
95	N	0	-12.410022	3.090300	-8.500061
96	C	0	-11.834949	2.511273	-7.275319
97	C	0	-10.796699	1.409872	-7.550830
98	O	0	-9.766612	1.339559	-6.860317
99	C	0	-12.914796	2.031556	-6.292924
100	C	0	-12.422688	1.687260	-4.870874
101	C	0	-11.684306	2.860854	-4.177271
102	C	0	-13.573415	1.197491	-3.987442
103	H	0	-11.734301	3.537605	-9.107748
104	H	0	-13.038751	2.476789	-9.007950
105	H	0	-11.268048	3.310723	-6.784981
106	H	0	-10.993308	0.699066	-8.366547
107	H	0	-13.430900	1.156614	-6.714450
108	H	0	-13.655930	2.839847	-6.234285
109	H	0	-11.696624	0.863655	-4.959790
110	H	0	-11.451153	2.591344	-3.142413
111	H	0	-12.317172	3.756522	-4.164440
112	H	0	-10.744894	3.102664	-4.681619
113	H	0	-14.142183	0.395918	-4.472040

114	H	0	-14.268888	2.017966	-3.771559
115	H	0	-13.189688	0.814500	-3.036695
116	N	0	-6.891174	1.455322	-6.436160
117	C	0	-6.340050	0.246024	-5.797466
118	C	0	-5.025968	-0.194196	-6.441877
119	O	0	-4.029823	-0.370427	-5.742530
120	C	0	-7.390491	-0.880843	-5.824474
121	C	0	-6.880020	-2.234825	-5.355196
122	C	0	-6.643086	-2.482794	-4.003928
123	C	0	-6.700251	-3.280155	-6.272212
124	C	0	-6.185430	-3.750859	-3.567950
125	C	0	-6.247106	-4.558960	-5.838573
126	C	0	-5.987152	-4.780457	-4.492098
127	H	0	-7.890544	1.428601	-6.598791
128	H	0	-6.557916	2.326639	-6.046682
129	H	0	-6.050817	0.423499	-4.754090
130	H	0	-7.770399	-0.963009	-6.849642
131	H	0	-8.221392	-0.546715	-5.193448
132	H	0	-6.800543	-1.693549	-3.273539
133	H	0	-6.902559	-3.111389	-7.325583
134	H	0	-5.984110	-3.921537	-2.518312
135	H	0	-6.110859	-5.350081	-6.566761
136	H	0	-5.636941	-5.749941	-4.155217
137	N	0	-2.269927	2.515179	-5.887402
138	C	0	-1.836194	2.620357	-4.489737
139	C	0	-1.010657	1.405431	-4.050132
140	O	0	-0.084114	1.538114	-3.260996
141	C	0	-3.057731	2.782427	-3.587584
142	C	0	-2.903909	3.000586	-2.081819
143	C	0	-1.924346	4.129321	-1.758002
144	C	0	-4.288632	3.318533	-1.532337
145	H	0	-1.554076	2.720372	-6.572926
146	H	0	-3.148013	2.976407	-6.090216

147	H	0	-1.160973	3.472778	-4.327583
148	H	0	-3.705458	1.909455	-3.759293
149	H	0	-3.609393	3.652814	-3.979743
150	H	0	-2.533427	2.074619	-1.619272
151	H	0	-1.929577	4.333374	-0.681324
152	H	0	-2.218963	5.050449	-2.279177
153	H	0	-0.901578	3.872433	-2.051184
154	H	0	-5.016821	2.552977	-1.823193
155	H	0	-4.638393	4.281271	-1.923040
156	H	0	-4.278617	3.381769	-0.440343
157	N	0	-1.341702	0.222888	-4.564468
158	C	0	-0.601442	-1.005675	-4.212878
159	C	0	0.799493	-1.024735	-4.789771
160	O	0	1.747479	-1.487302	-4.131529
161	C	0	-1.336965	-2.260853	-4.707974
162	S	0	-2.796525	-2.750346	-3.737980
163	H	0	-2.124329	0.169287	-5.216027
164	H	0	-0.461154	-1.051007	-3.127645
165	H	0	-1.695434	-2.097923	-5.726886
166	H	0	-0.634537	-3.096527	-4.727781
167	H	0	-2.139885	-2.974309	-2.548657
168	N	0	-4.258846	-6.771032	2.248831
169	C	0	-3.394472	-5.730271	1.674315
170	C	0	-3.932430	-4.303205	1.899691
171	O	0	-3.147997	-3.373967	2.098596
172	C	0	-3.032193	-6.015201	0.172218
173	C	0	-1.673935	-5.400028	-0.189914
174	C	0	-4.159129	-5.595083	-0.803649
175	C	0	-1.201434	-5.703737	-1.645037
176	H	0	-4.135975	-7.687137	1.836400
177	H	0	-4.266948	-6.799303	3.260972
178	H	0	-2.426873	-5.700548	2.200905
179	H	0	-2.927453	-7.110289	0.109527

180	H	0	-1.721181	-4.312057	-0.052699
181	H	0	-0.915841	-5.775061	0.508554
182	H	0	-3.968848	-5.985091	-1.807269
183	H	0	-4.218301	-4.500376	-0.866240
184	H	0	-5.128232	-5.976411	-0.465041
185	H	0	-1.136155	-6.784455	-1.814095
186	H	0	-0.212614	-5.265117	-1.803762
187	H	0	-1.885912	-5.284346	-2.386747
188	N	0	-5.253829	-4.137198	1.892991
189	C	0	-5.847766	-2.787814	1.983451
190	C	0	-6.129736	-2.351539	3.418950
191	O	0	-6.424773	-1.184450	3.665648
192	C	0	-7.154161	-2.642786	1.130219
193	C	0	-8.306707	-3.497726	1.685690
194	C	0	-6.891630	-3.041883	-0.320564
195	C	0	-9.150348	-2.830999	2.737429
196	H	0	-5.831781	-4.968750	1.846216
197	H	0	-5.093476	-2.087300	1.613392
198	H	0	-7.429910	-1.583287	1.182607
199	H	0	-7.918986	-4.455992	2.059172
200	H	0	-8.954564	-3.758166	0.839789
201	H	0	-6.008022	-2.535071	-0.726435
202	H	0	-7.753936	-2.784654	-0.942733
203	H	0	-6.729113	-4.122053	-0.394532
204	H	0	-10.066086	-3.407803	2.873531
205	H	0	-9.447872	-1.821006	2.439889
206	H	0	-8.659657	-2.765737	3.713214
207	N	0	-1.181779	-2.086749	7.009715
208	C	0	-0.939746	-0.740971	7.538962
209	C	0	-1.278729	-0.593633	9.026583
210	O	0	-1.441693	0.527189	9.505060
211	C	0	-1.717844	0.305728	6.719297
212	C	0	-1.165190	0.534285	5.308497

213	C	0	-1.288080	-0.703074	4.403111
214	O	0	-0.278925	-1.321988	4.029018
215	N	0	-2.521666	-1.089747	4.077090
216	H	0	-0.667100	-2.284631	6.158439
217	H	0	-1.039045	-2.819998	7.698045
218	H	0	0.124617	-0.456272	7.494230
219	H	0	-1.686251	1.257792	7.255891
220	H	0	-2.763544	-0.018074	6.672166
221	H	0	-1.709103	1.358113	4.836347
222	H	0	-0.109449	0.834732	5.372958
223	H	0	-3.227516	-0.470498	4.488646
224	N	0	-9.626930	8.306667	1.095341
225	C	0	-9.505557	7.607301	-0.182685
226	C	0	-10.159713	8.539766	-1.201007
227	O	0	-11.365397	8.778961	-1.142993
228	C	0	-10.222921	6.232782	-0.137351
229	C	0	-9.591607	5.323941	0.928736
230	C	0	-10.206146	5.541966	-1.509531
231	C	0	-10.583027	4.376501	1.582054
232	H	0	-10.578454	8.571328	1.324282
233	H	0	-8.958100	9.049958	1.252158
234	H	0	-8.447671	7.473230	-0.425183
235	H	0	-11.264883	6.444604	0.145575
236	H	0	-9.134934	5.955516	1.699700
237	H	0	-8.781163	4.746906	0.460164
238	H	0	-9.174143	5.409995	-1.854040
239	H	0	-10.753251	6.115400	-2.265589
240	H	0	-10.669552	4.553269	-1.441013
241	H	0	-11.078498	3.742363	0.838223
242	H	0	-11.359533	4.935996	2.114018
243	H	0	-10.088242	3.717352	2.301438
244	N	0	-4.560501	8.952497	-1.476449
245	C	0	-4.687710	8.227889	-0.214980

246	C	0	-3.878950	8.852262	0.906886
247	O	0	-2.720843	9.212450	0.711046
248	C	0	-4.228105	6.783189	-0.416895
249	C	0	-4.513279	5.828194	0.731307
250	C	0	-5.815407	5.413882	1.016185
251	C	0	-3.471959	5.287751	1.484342
252	C	0	-6.081543	4.499159	2.040092
253	C	0	-3.728346	4.359512	2.515909
254	C	0	-5.030638	3.977285	2.782818
255	O	0	-5.290735	3.070471	3.795687
256	H	0	-3.596486	9.037799	-1.787308
257	H	0	-5.034423	9.849130	-1.487547
258	H	0	-5.741721	8.230168	0.078934
259	H	0	-3.152143	6.790869	-0.634013
260	H	0	-4.734942	6.438453	-1.324025
261	H	0	-6.644193	5.801199	0.430303
262	H	0	-2.447243	5.587426	1.288178
263	H	0	-7.095589	4.183540	2.257473
264	H	0	-2.905759	3.955508	3.096962
265	H	0	-4.477996	2.757487	4.231930
266	N	0	0.237006	10.103568	-0.297672
267	C	0	1.073273	9.612578	-1.407959
268	O	0	-0.550729	10.395603	-2.976999
269	C	0	0.873386	8.102496	-1.650333
270	C	0	1.481647	7.194551	-0.599027
271	C	0	2.853555	7.209697	-0.343466
272	C	0	0.683710	6.292888	0.097919
273	C	0	3.412650	6.367883	0.631107
274	C	0	1.232619	5.435713	1.057062
275	C	0	2.593822	5.471248	1.320701
276	H	0	-0.755758	9.918627	-0.379760
277	H	0	0.633298	9.967119	0.620410
278	H	0	2.120713	9.843651	-1.204181

279	H	0	-0.206080	7.924655	-1.713347
280	H	0	1.294922	7.826009	-2.627850
281	H	0	3.499864	7.888651	-0.892476
282	H	0	-0.383979	6.271159	-0.099097
283	H	0	4.475642	6.414404	0.835337
284	H	0	0.600523	4.741914	1.596896
285	H	0	3.012739	4.799775	2.059594
286	N	0	7.266525	1.858776	-4.376455
287	C	0	7.802918	1.036081	-3.304427
288	C	0	8.731078	-0.059476	-3.787794
289	O	0	9.676986	-0.429863	-3.083585
290	C	0	6.690453	0.337130	-2.511380
291	C	0	5.401757	1.113878	-2.366236
292	C	0	5.395343	2.413646	-1.865426
293	C	0	4.175576	0.529119	-2.711314
294	C	0	4.195750	3.123978	-1.725169
295	C	0	2.982848	1.231450	-2.576680
296	C	0	3.001363	2.524711	-2.082046
297	O	0	1.807487	3.213277	-1.957600
298	H	0	7.959091	2.297813	-4.974628
299	H	0	6.519486	1.423894	-4.905856
300	H	0	8.411336	1.660093	-2.642904
301	H	0	7.092472	0.070145	-1.525779
302	H	0	6.450685	-0.612337	-3.007069
303	H	0	6.334353	2.895813	-1.611135
304	H	0	4.146347	-0.481038	-3.108059
305	H	0	4.208129	4.138913	-1.336188
306	H	0	2.028627	0.798705	-2.853793
307	H	0	1.926033	4.068234	-1.499595
308	N	0	7.776268	-5.372573	0.433678
309	C	0	7.644004	-4.823922	1.771429
310	C	0	8.914388	-5.116198	2.573617
311	O	0	9.756252	-5.920828	2.158683

312	C	0	6.445732	-5.416532	2.530800
313	C	0	5.094852	-5.057505	1.950259
314	C	0	4.673151	-5.571768	0.708986
315	C	0	4.234353	-4.212474	2.638356
316	C	0	3.418940	-5.223637	0.175803
317	C	0	2.986095	-3.873155	2.113278
318	C	0	2.587736	-4.381439	0.891166
319	O	0	1.347414	-4.023089	0.395351
320	H	0	8.114278	-6.336150	0.447166
321	H	0	8.349063	-4.797377	-0.177571
322	H	0	7.528106	-3.733692	1.729550
323	H	0	6.500706	-5.057544	3.563373
324	H	0	6.568356	-6.508540	2.544104
325	H	0	5.330379	-6.230930	0.157543
326	H	0	4.537466	-3.817068	3.603950
327	H	0	3.087707	-5.619840	-0.776731
328	H	0	2.286689	-3.293288	2.700405
329	H	0	0.763933	-3.668113	1.096574
330	O	0	20.782872	0.920129	0.561052
331	C	0	21.103141	0.970172	-1.849954
332	O	0	21.655033	2.242041	-1.554992
333	C	0	22.147031	-0.121708	-2.047201
334	N	0	22.780401	-0.191014	-3.383501
335	C	0	22.198503	0.016165	-4.617477
336	N	0	23.136636	-0.167664	-5.574331
337	C	0	24.317028	-0.475988	-4.974698
338	C	0	25.596300	-0.753251	-5.469106
339	N	0	25.848381	-0.785661	-6.787846
340	N	0	26.610545	-1.057671	-4.571019
341	C	0	26.377847	-1.067312	-3.212422
342	N	0	25.114575	-0.787903	-2.735363
343	C	0	24.100876	-0.497893	-3.598219
344	N	0	7.393573	-1.351442	2.889847

345	C	0	6.706616	-1.728878	4.023035
346	O	0	6.930116	-2.818177	4.549789
347	N	0	5.815179	-0.847081	4.602497
348	C	0	5.568231	0.387899	4.025994
349	O	0	4.775829	1.160651	4.572715
350	C	0	6.248175	0.769848	2.874738
351	N	0	6.029702	2.010833	2.283131
352	C	0	6.849094	2.465335	1.271764
353	C	0	6.804732	3.812777	0.897929
354	C	0	7.850694	4.385653	0.170545
355	C	0	7.730721	5.837852	-0.197169
356	C	0	8.963453	3.611340	-0.198008
357	C	0	10.129338	4.170518	-0.981967
358	C	0	9.001375	2.258118	0.178436
359	C	0	7.944702	1.676740	0.907711
360	N	0	7.917395	0.314918	1.230179
361	C	0	7.171170	-0.107106	2.313282
362	C	0	8.890789	-0.631875	0.569646
363	C	0	10.061584	-1.014581	1.493564
364	O	0	10.710456	0.165064	1.925279
365	C	0	11.015355	-1.940286	0.732709
366	O	0	10.323431	-3.125747	0.375589
367	C	0	12.289418	-2.349661	1.489847
368	O	0	12.873504	-1.230287	2.136685
369	C	0	13.294404	-2.916439	0.492299
370	O	0	14.410122	-3.440967	1.195999
371	15	0	15.630377	-4.166164	0.429628
372	O	0	16.748127	-4.354719	1.555266
373	O	0	15.185344	-5.286043	-0.395613
374	O	0	16.096704	-3.076103	-0.616815
375	15	0	16.609690	-1.523932	-0.545982
376	O	0	16.443648	-0.946533	-2.026662
377	O	0	16.015459	-0.792549	0.636972

378	O	0	18.237116	-1.650728	-0.238875
379	C	0	19.172242	-1.775812	-1.272709
380	C	0	20.447041	-1.086879	-0.817377
381	O	0	21.442819	-1.321564	-1.787008
382	C	0	20.284932	0.428002	-0.695303
383	H	0	20.158253	0.626522	1.264172
384	H	0	20.474300	1.064898	-2.737433
385	H	0	21.746086	2.326174	-0.581133
386	H	0	22.972515	0.014373	-1.338306
387	H	0	21.164389	0.261682	-4.778257
388	H	0	26.775554	-1.004079	-7.115794
389	H	0	25.121652	-0.580127	-7.454802
390	H	0	27.175134	-1.298228	-2.525582
391	H	0	5.366040	-1.094364	5.494777
392	H	0	5.964619	4.407745	1.234677
393	H	0	6.814366	6.268765	0.206402
394	H	0	7.717629	5.968656	-1.285074
395	H	0	8.576546	6.419688	0.184460
396	H	0	10.575327	5.027165	-0.466066
397	H	0	9.794575	4.524749	-1.963371
398	H	0	10.897398	3.411802	-1.130008
399	H	0	9.888829	1.679010	-0.036283
400	H	0	9.232198	-0.177427	-0.360011
401	H	0	8.329397	-1.540537	0.337556
402	H	0	9.651270	-1.571963	2.343311
403	H	0	11.626195	-0.047596	2.224840
404	H	0	11.309730	-1.442902	-0.201145
405	H	0	10.486592	-3.871622	0.986699
406	H	0	12.034695	-3.110588	2.242128
407	H	0	13.847246	-1.197217	2.018298
408	H	0	12.822697	-3.693104	-0.119558
409	H	0	13.621025	-2.097877	-0.164154
410	H	0	19.383642	-2.829781	-1.482512

411	H	0	18.804598	-1.307320	-2.192747
412	H	0	20.756118	-1.495152	0.151903
413	H	0	19.231612	0.709598	-0.796099
414	O	0	-13.723818	-9.959186	-5.271026
415	H	0	-13.580558	-10.947523	-5.196727
416	O	0	-9.392492	9.128982	-2.166811
417	H	0	-9.931969	9.724153	-2.762305
418	O	0	-1.369828	-1.701528	9.815875
419	H	0	-1.580089	-1.466588	10.767972
420	O	0	-4.961574	-0.405292	-7.787220
421	H	0	-4.045506	-0.680004	-8.080765
422	O	0	-6.057507	-3.260927	4.434799
423	H	0	-6.235745	-2.832511	5.323558
424	O	0	-18.588550	1.989569	1.269721
425	H	0	-19.424483	1.578615	0.894564
426	N	0	-13.353084	-1.947875	6.383954
427	H	0	-13.430150	-1.233766	7.108490
428	O	0	-4.429239	8.991956	2.147370
429	H	0	-3.790692	9.407473	2.799426
430	O	0	1.015023	-0.542980	-6.042538
431	H	0	1.980699	-0.599628	-6.302485
432	O	0	1.563082	10.890801	-3.486654
433	H	0	1.170957	11.299408	-4.310989
434	O	0	8.511795	-0.650050	-4.996642
435	H	0	9.194638	-1.355324	-5.188423
436	O	0	9.139036	-4.485092	3.755988
437	H	0	10.019261	-4.741582	4.148781
438	O	0	4.109167	-1.934469	9.186967
439	H	0	4.305512	-2.898678	9.169949
440	C	0	0.166793	-0.829214	2.635913
441	C	0	-0.021339	1.650615	2.225744
442	C	0	1.641747	-0.613050	2.805846
443	C	0	1.388670	1.826706	2.609292

444	C	0	2.186949	0.608251	2.826926
445	H	0	2.244355	-1.487532	3.001273
446	O	0	1.886916	2.956247	2.761085
447	H	0	-0.575450	2.529698	1.918287
448	C	0	0.698028	10.357425	-2.679212
449	H	0	-0.927975	10.868283	-3.752734
450	H	0	3.236688	0.769593	3.031287
451	N	0	-0.262334	-1.871275	1.687202
452	H	0	-1.151655	-2.318758	1.961050
453	C	0	-0.132598	-1.686817	0.300094
454	C	0	1.073333	-0.955609	-0.219513
455	H	0	1.979817	-1.239971	0.318191
456	H	0	1.193306	-1.209922	-1.273779
457	H	0	0.943802	0.129329	-0.139654
458	O	0	-0.982688	-2.170130	-0.467764
459	C	0	-0.589019	0.442024	2.266204
460	H	0	-1.628656	0.296359	1.995939

Catechol-O-methyltransferase: Overall Charge = 0, Total energy = -8546.03186336 Ha

1	C	0	3.609931	9.607122	-1.551354
2	C	0	3.946495	8.597740	-2.640649
3	O	0	3.678432	8.820534	-3.823222
4	C	0	2.244037	9.266350	-0.905672
5	C	0	2.245323	7.952218	-0.189770
6	C	0	2.684922	7.714731	1.091813
7	C	0	1.884316	6.669265	-0.741233
8	N	0	2.622919	6.361366	1.372088
9	C	0	2.137742	5.692560	0.263972
10	C	0	1.398634	6.262271	-1.996899
11	C	0	1.914076	4.327378	0.041543
12	C	0	1.157647	4.910492	-2.215286

13	C	0	1.414866	3.958282	-1.201514
14	H	0	4.373503	9.534934	-0.771987
15	H	0	1.471731	9.286325	-1.686363
16	H	0	2.028135	10.090176	-0.219488
17	H	0	3.045109	8.412230	1.832201
18	H	0	2.912172	5.956419	2.255669
19	H	0	1.215102	6.991350	-2.781447
20	H	0	2.159318	3.569021	0.776677
21	H	0	0.775817	4.575234	-3.173886
22	H	0	1.237639	2.907824	-1.400934
23	N	0	4.444122	3.955447	-2.953406
24	C	0	4.354285	2.633085	-2.317666
25	C	0	5.684558	1.895120	-2.092350
26	O	0	5.736278	0.653067	-2.135973
27	C	0	3.372625	1.682883	-3.049569
28	C	0	3.833865	1.347845	-4.464211
29	S	0	2.586590	0.213291	-5.299128
30	C	0	3.349085	0.183291	-7.006852
31	H	0	4.639678	3.937453	-3.948091
32	H	0	3.644002	4.538855	-2.726789
33	H	0	3.961704	2.776610	-1.298273
34	H	0	2.398553	2.181899	-3.093674
35	H	0	3.248708	0.762474	-2.469383
36	H	0	3.907301	2.244067	-5.086387
37	H	0	4.795512	0.832095	-4.451537
38	H	0	3.362444	1.189915	-7.424195
39	H	0	2.729998	-0.464856	-7.625145
40	H	0	4.361204	-0.216552	-6.951655
41	N	0	6.745771	2.632016	-1.793643
42	C	0	8.000474	2.038064	-1.354100
43	C	0	7.841109	1.503923	0.049497
44	O	0	7.065252	2.080712	0.827182
45	C	0	9.110735	3.090093	-1.376336

46	C	0	9.483456	3.457404	-2.802443
47	O	0	9.374935	2.633813	-3.737486
48	N	0	9.959184	4.707502	-2.980883
49	H	0	6.625556	3.638929	-1.780621
50	H	0	8.264192	1.235072	-2.050144
51	H	0	10.003135	2.683771	-0.885354
52	H	0	8.803479	3.970891	-0.802425
53	H	0	10.031591	5.370841	-2.225584
54	H	0	10.266308	4.995789	-3.898363
55	N	0	8.588669	0.474625	0.447367
56	C	0	8.463782	-0.039176	1.822501
57	C	0	8.767702	1.033780	2.857536
58	O	0	8.218722	1.029890	3.970534
59	C	0	9.344960	-1.294946	2.011631
60	C	0	10.842615	-0.970491	1.937379
61	C	0	9.003179	-2.009722	3.324024
62	H	0	9.195390	-0.013563	-0.197282
63	H	0	7.414324	-0.306177	1.992973
64	H	0	9.077389	-1.960708	1.179101
65	H	0	11.104665	-0.408123	1.036101
66	H	0	11.423652	-1.896372	1.940984
67	H	0	11.150254	-0.375104	2.803720
68	H	0	9.247153	-1.379735	4.185168
69	H	0	9.585302	-2.931946	3.402765
70	H	0	7.941066	-2.267458	3.380273
71	N	0	5.979556	-8.241108	-1.754126
72	C	0	5.500210	-6.838064	-1.749147
73	C	0	4.843409	-6.489703	-3.073862
74	O	0	4.992529	-5.351999	-3.564134
75	C	0	6.626803	-5.866643	-1.438538
76	C	0	6.189607	-4.463355	-1.062953
77	O	0	4.993174	-4.268707	-0.592708
78	O	0	7.060522	-3.539890	-1.163408

79	H	0	5.285357	-8.891404	-2.108794
80	H	0	6.330601	-8.530119	-0.847050
81	H	0	4.711938	-6.674263	-0.992193
82	H	0	7.327171	-5.799948	-2.272853
83	H	0	7.182188	-6.262694	-0.577962
84	C	0	-1.677957	-8.551870	-1.965859
85	C	0	-2.423743	-9.863755	-2.195370
86	O	0	-2.176173	-10.828367	-1.458709
87	C	0	-2.360333	-7.762023	-0.834786
88	C	0	-1.683058	-6.406638	-0.594669
89	C	0	-0.156042	-6.473595	-0.481052
90	C	0	0.417009	-5.063595	-0.458889
91	N	0	1.918468	-5.053975	-0.431493
92	H	0	-0.697614	-8.891567	-1.612132
93	H	0	-2.338843	-8.374876	0.072612
94	H	0	-3.411181	-7.590543	-1.098195
95	H	0	-1.942949	-5.741044	-1.425791
96	H	0	-2.099262	-5.964445	0.318993
97	H	0	0.248066	-6.997549	-1.354101
98	H	0	0.142848	-7.027197	0.418787
99	H	0	0.066733	-4.491408	0.405349
100	H	0	0.127734	-4.518824	-1.359771
101	H	0	2.298947	-5.379676	-1.323008
102	H	0	2.294908	-5.629931	0.327341
103	H	0	2.245220	-4.026670	-0.333113
104	N	0	5.400973	-7.688454	3.304974
105	C	0	4.972475	-6.329046	3.577731
106	C	0	3.457389	-6.241001	3.694844
107	O	0	2.724024	-7.006532	3.086627
108	C	0	5.427340	-5.310968	2.517080
109	C	0	5.181710	-3.888384	3.023219
110	O	0	5.457760	-3.613479	4.221149
111	O	0	4.659644	-3.031389	2.196516

112	H	0	5.402075	-8.278151	4.129983
113	H	0	4.863541	-8.118259	2.557138
114	H	0	5.421523	-6.012744	4.523855
115	H	0	4.917209	-5.463311	1.561088
116	H	0	6.502172	-5.449825	2.354786
117	N	0	2.992591	-5.235915	4.444238
118	C	0	1.587955	-4.803043	4.366010
119	C	0	0.594500	-5.785810	4.957355
120	O	0	-0.567173	-5.835350	4.581992
121	C	0	1.176739	-4.337372	2.971083
122	C	0	0.379466	-3.048586	3.056475
123	O	0	-0.306463	-2.778628	4.069023
124	N	0	0.470210	-2.254777	1.973479
125	H	0	3.651068	-4.622870	4.912067
126	H	0	1.512933	-3.933582	5.032119
127	H	0	0.557889	-5.099322	2.485165
128	H	0	2.080896	-4.177574	2.371743
129	H	0	1.189263	-2.399560	1.265678
130	C	0	-3.629658	-2.869493	3.372992
131	C	0	-4.352778	-4.044686	4.004924
132	O	0	-5.557340	-4.204870	4.195994
133	C	0	-4.605394	-1.976182	2.595015
134	C	0	-4.623638	-2.581918	1.162509
135	C	0	-3.492825	-3.641810	1.150275
136	H	0	-3.089285	-2.346830	4.167807
137	H	0	-5.595262	-1.957087	3.055629
138	H	0	-4.217097	-0.956304	2.581239
139	H	0	-4.435447	-1.805653	0.416009
140	H	0	-5.590298	-3.035903	0.927460
141	H	0	-3.926144	-4.655518	1.206109
142	H	0	-2.874936	-3.591634	0.249493
143	C	0	5.506155	4.166075	4.453837
144	C	0	4.984257	5.605595	4.428452

145	O	0	3.894449	5.877722	3.932743
146	C	0	5.847570	3.749260	2.999724
147	C	0	4.641434	3.463741	2.088969
148	C	0	4.268306	1.982060	1.950315
149	O	0	4.511114	1.200100	2.940024
150	O	0	3.670223	1.632507	0.870207
151	H	0	6.457964	4.205609	5.001762
152	H	0	6.475581	4.528826	2.553670
153	H	0	6.455313	2.844351	3.060518
154	H	0	4.822719	3.843994	1.080204
155	H	0	3.751321	3.977574	2.473254
156	C	0	-9.942095	-4.352734	-0.922298
157	C	0	-10.115284	-4.097683	-2.411722
158	O	0	-9.681911	-4.881505	-3.235807
159	C	0	-8.535910	-3.848374	-0.493086
160	C	0	-8.344481	-2.377578	-0.706864
161	C	0	-8.718817	-1.375667	0.157551
162	C	0	-7.811260	-1.726636	-1.883665
163	N	0	-8.451344	-0.140627	-0.408876
164	C	0	-7.886444	-0.323122	-1.656348
165	C	0	-7.297323	-2.193847	-3.107484
166	C	0	-7.446245	0.612306	-2.601007
167	C	0	-6.872969	-1.269383	-4.054346
168	C	0	-6.944825	0.121655	-3.803713
169	H	0	-10.699241	-3.795712	-0.366806
170	H	0	-8.425982	-4.112514	0.562341
171	H	0	-7.784735	-4.421649	-1.051683
172	H	0	-9.163753	-1.448650	1.137589
173	H	0	-8.655779	0.747936	0.019126
174	H	0	-7.229308	-3.255767	-3.325510
175	H	0	-7.517107	1.678082	-2.412391
176	H	0	-6.488204	-1.624950	-5.004143
177	H	0	-6.640758	0.819260	-4.577879

178	N	0	-3.076677	10.151686	-1.855862
179	C	0	-2.678424	8.771382	-2.034310
180	C	0	-3.532821	8.150235	-3.142791
181	O	0	-4.738746	8.044651	-2.968637
182	C	0	-2.799870	7.925433	-0.754494
183	C	0	-2.381254	6.476888	-1.040670
184	C	0	-1.931196	8.524427	0.354349
185	H	0	-2.752230	10.794239	-2.566875
186	H	0	-4.056376	10.277154	-1.626371
187	H	0	-1.628773	8.755508	-2.350199
188	H	0	-3.855669	7.945012	-0.446128
189	H	0	-2.449299	5.873377	-0.130702
190	H	0	-1.341591	6.445510	-1.388415
191	H	0	-3.012296	6.005068	-1.802119
192	H	0	-2.184643	9.571531	0.527822
193	H	0	-0.870844	8.464919	0.075070
194	H	0	-2.065511	7.965241	1.285122
195	C	0	-8.481462	6.408294	1.361465
196	C	0	-9.478473	5.285910	1.619068
197	O	0	-9.672704	4.863298	2.767501
198	C	0	-7.061817	5.842781	1.224318
199	C	0	-6.884929	4.805384	0.099765
200	C	0	-7.428962	5.311051	-1.243967
201	C	0	-5.396583	4.463706	-0.040769
202	H	0	-8.759470	6.909454	0.431424
203	H	0	-6.758013	5.403798	2.184690
204	H	0	-6.404015	6.700440	1.036086
205	H	0	-7.433050	3.890402	0.378622
206	H	0	-6.966532	6.273074	-1.499000
207	H	0	-8.514694	5.434985	-1.228892
208	H	0	-7.184658	4.601185	-2.040819
209	H	0	-4.845965	5.357146	-0.357687
210	H	0	-5.242838	3.686951	-0.798162

211	H	0	-4.973855	4.111148	0.904736
212	N	0	-9.684983	2.351387	4.407128
213	C	0	-8.485905	2.482645	5.243982
214	C	0	-8.556343	3.721948	6.122332
215	O	0	-9.461954	4.539670	6.014203
216	C	0	-7.217441	2.647736	4.382845
217	C	0	-7.024118	1.525296	3.375750
218	S	0	-5.325149	1.752445	2.599382
219	C	0	-5.600453	0.955602	0.933450
220	H	0	-9.842789	3.173922	3.827681
221	H	0	-10.512995	2.104607	4.938635
222	H	0	-8.383001	1.598845	5.881199
223	H	0	-6.353269	2.715226	5.053041
224	H	0	-7.287261	3.601299	3.843396
225	H	0	-7.067210	0.540300	3.846097
226	H	0	-7.774747	1.576413	2.585781
227	H	0	-6.240895	1.588939	0.318818
228	H	0	-6.051053	-0.030443	1.058945
229	H	0	-4.621500	0.857274	0.463819
230	Mg	0	4.373767	-2.597498	0.364113
231	C	0	-0.094642	-0.200018	-2.452998
232	C	0	0.353553	0.264853	-1.213458
233	C	0	1.269107	-0.475376	-0.462981
234	C	0	0.413948	-1.410042	-2.943788
235	C	0	1.321137	-2.150364	-2.193536
236	H	0	1.653545	-0.092137	0.476086
237	H	0	-0.120659	-1.438581	1.914148
238	O	0	-5.406593	-4.221302	-4.865178
239	C	0	-4.255174	-3.568223	-4.294158
240	C	0	-3.166864	-3.488178	-5.371520
241	C	0	-3.741856	-4.276299	-3.038737
242	O	0	-3.438378	-5.648944	-3.329595
243	C	0	-4.726488	-4.257920	-1.868181

244	H	0	-5.137301	-4.889526	-5.523902
245	H	0	-4.596841	-2.570080	-4.009428
246	H	0	-3.569735	-3.031128	-6.278323
247	H	0	-2.807540	-4.496260	-5.602631
248	H	0	-2.313462	-2.893571	-5.033849
249	H	0	-2.789246	-3.821058	-2.747694
250	H	0	-4.265817	-6.138004	-3.512076
251	H	0	-4.933228	-3.238258	-1.529595
252	H	0	-4.327270	-4.844898	-1.038624
253	H	0	-5.673613	-4.705374	-2.191468
254	N	0	4.581799	3.192006	5.051571
255	H	0	4.496504	2.345588	4.483426
256	H	0	4.704256	3.041480	6.043227
257	O	0	5.770136	6.660853	4.987731
258	H	0	5.305828	7.513417	4.849708
259	O	0	-3.392456	-9.991630	-3.238792
260	H	0	-3.760752	-10.899953	-3.215805
261	N	0	-1.515698	-7.717896	-3.164950
262	H	0	-1.251031	-8.212307	-4.005502
263	H	0	-2.192518	-6.971352	-3.292750
264	N	0	-10.092858	-5.788877	-0.647262
265	H	0	-11.055120	-6.107888	-0.663713
266	H	0	-9.498383	-6.361648	-1.238519
267	O	0	-10.795755	-2.923464	-2.861648
268	H	0	-10.771463	-2.897015	-3.841510
269	O	0	-10.201245	4.708226	0.529002
270	H	0	-10.782855	3.992733	0.864464
271	N	0	-8.577986	7.385901	2.454987
272	H	0	-8.447684	6.962292	3.368697
273	H	0	-9.418154	7.952421	2.421338
274	N	0	3.597686	10.965860	-2.080822
275	H	0	2.961674	11.075554	-2.865038
276	H	0	4.519059	11.324842	-2.306666

277	O	0	4.597380	7.379133	-2.272446
278	H	0	4.710644	6.801629	-3.056781
279	O	0	-2.918167	7.704756	-4.354495
280	H	0	-3.604918	7.353322	-4.960384
281	O	0	-7.547383	3.957169	7.108319
282	H	0	-7.741825	4.804006	7.561361
283	O	0	4.054044	-7.465891	-3.758321
284	H	0	3.705955	-7.074921	-4.587155
285	O	0	1.009665	-6.690795	5.984558
286	H	0	0.230770	-7.220778	6.253975
287	O	0	-3.426345	-5.059238	4.399745
288	H	0	-3.896687	-5.815996	4.806309
289	N	0	-2.692925	-3.304540	2.336504
290	H	0	-2.025544	-4.001155	2.655946
291	H	0	6.496528	-2.131784	-0.687859
292	O	0	5.832291	-1.471447	-0.292531
293	H	0	5.801917	-0.617052	-0.780260
294	H	0	0.104747	-1.772312	-3.920325
295	H	0	-0.025172	1.203172	-0.821385
296	C	0	1.731916	-1.711611	-0.922872
297	C	0	-1.157373	0.553203	-3.223520
298	H	0	-1.047943	1.631138	-3.059203
299	H	0	-1.046659	0.368701	-4.297701
300	C	0	-2.544330	0.097642	-2.759187
301	H	0	-2.696029	0.308523	-1.700209
302	H	0	-2.682264	-0.970329	-2.931300
303	O	0	2.504889	-2.525574	-0.131364
304	O	0	1.847665	-3.365013	-2.627600
305	H	0	1.640385	-3.550954	-3.561934
306	H	0	-4.578146	0.482497	-3.246852
307	H	0	-3.606441	1.826523	-3.345162
308	H	0	-3.542895	0.666824	-4.529066
309	N	0	-3.640492	0.816019	-3.518682

310	O	0	9.696138	1.942193	2.483369
311	H	0	9.866991	2.611460	3.179048

Aldehyde Dehydrogenase: Overall Charge = +1, Total energy = -6685.95728741 Ha

1	N	0	-5.830976	-0.873696	-6.965919
2	C	0	-6.167315	-1.001223	-5.550789
3	C	0	-7.426862	-0.267637	-5.143158
4	O	0	-8.250480	-0.816512	-4.381662
5	H	0	-6.505223	-1.285462	-7.598402
6	H	0	-5.551406	0.056592	-7.249718
7	H	0	-6.334751	-2.044528	-5.286952
8	H	0	-5.338020	-0.623808	-4.944354
9	N	0	-9.795300	-2.716989	-2.807265
10	C	0	-10.724513	-2.371713	-1.746669
11	C	0	-11.870651	-1.470325	-2.143643
12	O	0	-12.954647	-1.424563	-1.561644
13	C	0	-9.927600	-1.668794	-0.596076
14	O	0	-8.606875	-2.257851	-0.551760
15	C	0	-10.596461	-1.828150	0.755078
16	H	0	-9.506847	-1.943205	-3.404105
17	H	0	-10.046634	-3.542480	-3.337331
18	H	0	-11.169912	-3.285446	-1.347432
19	H	0	-9.824829	-0.607025	-0.865362
20	H	0	-8.459558	-2.624204	-1.468061
21	H	0	-10.690457	-2.890810	0.998213
22	H	0	-11.593244	-1.379018	0.747629
23	H	0	-10.001543	-1.352444	1.538367
24	N	0	-1.127879	8.131307	-0.012706
25	C	0	-1.731133	7.700306	-1.271907
26	C	0	-3.125119	8.306976	-1.265701
27	O	0	-4.043026	7.758367	-0.650731
28	C	0	-1.779123	6.171027	-1.478219

29	C	0	-0.369610	5.632737	-1.530022
30	C	0	0.302776	5.268261	-0.355929
31	C	0	0.328977	5.600596	-2.746491
32	C	0	1.655860	4.916134	-0.390153
33	C	0	1.674792	5.238014	-2.788582
34	C	0	2.347610	4.908220	-1.606201
35	H	0	-1.531546	7.698912	0.811578
36	H	0	-0.115914	8.125079	-0.003012
37	H	0	-1.159926	8.154802	-2.086304
38	H	0	-2.312900	5.944180	-2.408184
39	H	0	-2.342688	5.730110	-0.649050
40	H	0	-0.227925	5.281683	0.590471
41	H	0	-0.185025	5.878201	-3.661656
42	H	0	2.179503	4.686613	0.533449
43	H	0	2.204368	5.237408	-3.734846
44	H	0	3.410194	4.683070	-1.624059
45	N	0	-6.083919	5.982101	-2.974266
46	C	0	-6.174768	4.706398	-2.248491
47	C	0	-6.738404	4.934559	-0.853519
48	O	0	-7.643886	4.214266	-0.424556
49	C	0	-4.804910	4.003377	-2.164862
50	C	0	-4.872730	2.749463	-1.284137
51	C	0	-4.310922	3.661397	-3.572533
52	H	0	-5.513421	6.665333	-2.481117
53	H	0	-6.980751	6.366062	-3.249457
54	H	0	-6.893893	4.051446	-2.747001
55	H	0	-4.115038	4.723141	-1.704326
56	H	0	-5.094133	2.978584	-0.236273
57	H	0	-3.916795	2.218470	-1.307581
58	H	0	-5.648193	2.063185	-1.647288
59	H	0	-3.302867	3.237484	-3.528196
60	H	0	-4.296321	4.550475	-4.205449
61	H	0	-4.973005	2.918025	-4.034504

62	N	0	-10.040650	3.680489	0.995401
63	C	0	-9.942173	2.869693	2.232175
64	C	0	-10.825516	3.424902	3.332536
65	O	0	-11.318578	2.679930	4.160628
66	C	0	-8.474609	2.797762	2.692290
67	C	0	-7.709742	1.767901	1.911079
68	C	0	-8.231948	0.798470	1.091221
69	C	0	-6.282426	1.554262	1.927545
70	N	0	-7.225940	-0.021179	0.604722
71	C	0	-6.016529	0.422471	1.103178
72	C	0	-5.211465	2.219625	2.550010
73	C	0	-4.713784	-0.051812	0.898852
74	C	0	-3.919472	1.755347	2.340171
75	C	0	-3.675165	0.628846	1.522289
76	H	0	-9.176933	3.739649	0.469799
77	H	0	-10.853600	3.477332	0.427606
78	H	0	-10.320354	1.845807	2.118101
79	H	0	-8.031859	3.794632	2.572255
80	H	0	-8.435852	2.555409	3.762131
81	H	0	-9.255277	0.636192	0.797304
82	H	0	-7.412284	-0.869838	0.070352
83	H	0	-5.392404	3.084437	3.180402
84	H	0	-4.523079	-0.913553	0.268926
85	H	0	-3.082330	2.260388	2.808576
86	H	0	-2.654502	0.292694	1.374650
87	N	0	12.436758	-2.947880	-1.972238
88	C	0	11.164897	-2.340850	-2.336650
89	C	0	10.439272	-1.875680	-1.077136
90	O	0	9.821214	-0.813588	-1.064864
91	C	0	10.274873	-3.373790	-3.066978
92	C	0	10.864978	-3.810002	-4.363807
93	N	0	10.899133	-3.010244	-5.496482
94	C	0	11.510164	-4.971596	-4.725755

95	C	0	11.544328	-3.702940	-6.486133
96	N	0	11.929104	-4.898115	-6.053161
97	H	0	13.146281	-2.294072	-1.661776
98	H	0	12.795493	-3.595202	-2.666838
99	H	0	11.260166	-1.445162	-2.966852
100	H	0	9.278879	-2.940095	-3.211959
101	H	0	10.163381	-4.248872	-2.418903
102	H	0	10.504446	-2.085646	-5.583455
103	H	0	11.684150	-5.846047	-4.121335
104	H	0	11.700433	-3.305341	-7.473278
105	N	0	10.555995	-2.662060	-0.015321
106	C	0	9.963307	-2.329065	1.271711
107	C	0	10.286772	-0.944424	1.804167
108	O	0	9.434203	-0.315029	2.409834
109	H	0	11.222076	-3.423809	-0.095155
110	H	0	10.307948	-3.064544	2.002352
111	H	0	8.873678	-2.391192	1.224494
112	N	0	9.738246	2.119516	-1.708886
113	C	0	8.476491	2.282035	-2.415885
114	C	0	7.932631	3.702287	-2.256821
115	O	0	7.932722	4.255355	-1.152627
116	C	0	7.383204	1.323523	-1.827907
117	C	0	6.071731	1.188024	-2.565407
118	C	0	5.703946	-0.062313	-3.091453
119	C	0	5.173528	2.252666	-2.728933
120	C	0	4.499664	-0.246243	-3.763482
121	C	0	3.967678	2.088257	-3.418771
122	C	0	3.634839	0.834971	-3.938233
123	O	0	2.453192	0.593989	-4.616253
124	H	0	10.019791	1.142185	-1.661354
125	H	0	9.715370	2.541169	-0.783727
126	H	0	8.608010	2.073196	-3.479760
127	H	0	7.857927	0.339676	-1.784777

128	H	0	7.211912	1.649519	-0.791901
129	H	0	6.366418	-0.911081	-2.947186
130	H	0	5.403734	3.232181	-2.322823
131	H	0	4.216085	-1.215883	-4.153875
132	H	0	3.296683	2.930838	-3.557685
133	H	0	1.913136	1.395659	-4.741246
134	N	0	5.876000	5.736921	2.748698
135	C	0	4.906936	4.740268	3.180867
136	C	0	5.048148	4.528648	4.666745
137	O	0	6.156805	4.328023	5.166146
138	C	0	5.068068	3.364965	2.506693
139	S	0	4.946235	3.511547	0.638039
140	H	0	6.845482	5.469882	2.873716
141	H	0	5.674351	6.152089	1.849200
142	H	0	3.904425	5.119208	2.968687
143	H	0	6.028874	2.913035	2.766151
144	H	0	4.262853	2.692561	2.812240
145	H	0	6.252843	3.810292	0.341356
146	N	0	4.472475	1.903029	6.240963
147	C	0	5.064779	0.587261	6.482992
148	C	0	6.495972	0.423567	5.982300
149	O	0	7.000296	-0.696492	5.968631
150	C	0	4.170746	-0.577819	6.011700
151	C	0	4.064414	-0.672539	4.480971
152	C	0	2.796739	-0.467683	6.685081
153	C	0	3.251873	-1.892379	4.032129
154	H	0	5.196365	2.615751	6.225860
155	H	0	3.879628	1.954788	5.419020
156	H	0	5.169375	0.465050	7.573164
157	H	0	4.661782	-1.493760	6.360927
158	H	0	5.072123	-0.732805	4.051249
159	H	0	3.600949	0.241528	4.080237
160	H	0	2.219937	-1.384003	6.539837

161	H	0	2.222913	0.368013	6.271660
162	H	0	2.904016	-0.302522	7.761414
163	H	0	3.632272	-2.801557	4.512106
164	H	0	3.318033	-2.026092	2.947993
165	H	0	2.194013	-1.792137	4.293199
166	N	0	5.366164	-5.767813	2.325292
167	C	0	4.528416	-4.761430	1.683045
168	C	0	3.337503	-5.321847	0.928457
169	O	0	2.841990	-4.675346	0.012370
170	H	0	4.895496	-6.299895	3.046783
171	H	0	5.865211	-6.365513	1.678455
172	H	0	5.112288	-4.193491	0.958133
173	H	0	4.137930	-4.060720	2.427647
174	N	0	-0.337897	-5.973871	0.786319
175	C	0	-1.510498	-5.386186	1.410902
176	C	0	-2.677714	-5.667944	0.470524
177	O	0	-2.561033	-5.487033	-0.725059
178	C	0	-1.333707	-3.867561	1.605766
179	C	0	-2.587441	-3.256319	2.244554
180	C	0	-0.085508	-3.563345	2.440452
181	H	0	-0.130329	-5.513813	-0.098921
182	H	0	-0.454632	-6.972769	0.624565
183	H	0	-1.677463	-5.857847	2.386172
184	H	0	-1.203585	-3.434231	0.602442
185	H	0	-2.461453	-2.178341	2.379337
186	H	0	-2.768102	-3.700643	3.230811
187	H	0	-3.480684	-3.414001	1.631267
188	H	0	-0.167023	-4.026824	3.430922
189	H	0	0.021694	-2.482684	2.579262
190	H	0	0.815856	-3.945806	1.955543
191	N	0	-3.783810	-6.158773	1.003025
192	C	0	-4.931466	-6.472555	0.164028
193	C	0	-6.210957	-6.116989	0.911059

194	O	0	-6.226700	-6.048414	2.139961
195	C	0	-4.951196	-7.979508	-0.176702
196	O	0	-6.143475	-8.333384	-0.908259
197	H	0	-3.912902	-6.254163	2.002877
198	H	0	-4.853800	-5.896804	-0.760482
199	H	0	-4.862937	-8.555242	0.750649
200	H	0	-4.099978	-8.207432	-0.818807
201	H	0	-6.897372	-8.481443	-0.302690
202	N	0	-7.292357	-5.916696	0.157694
203	C	0	-8.579888	-5.523154	0.736367
204	C	0	-9.145305	-6.544821	1.741660
205	O	0	-9.925913	-6.184220	2.646794
206	C	0	-9.604190	-5.389803	-0.397042
207	H	0	-7.241681	-6.055660	-0.843057
208	H	0	-8.477801	-4.585967	1.289278
209	H	0	-9.724623	-6.347112	-0.912006
210	H	0	-9.263838	-4.636309	-1.108589
211	H	0	-10.572733	-5.088472	0.006545
212	C	0	3.621028	-0.557791	0.770926
213	C	0	3.689287	-1.655988	-0.092381
214	C	0	6.102561	-1.502645	-0.169050
215	O	0	-11.082630	4.829719	3.401937
216	H	0	-11.649221	4.998833	4.184080
217	O	0	-7.673505	1.053982	-5.629696
218	H	0	-8.530636	1.373600	-5.276669
219	O	0	7.464188	4.266754	-3.387163
220	H	0	7.098407	5.166832	-3.247678
221	O	0	2.793589	-6.596865	1.279432
222	H	0	1.836202	-6.601283	1.013996
223	O	0	-8.765866	-7.918809	1.629153
224	H	0	-9.216723	-8.438276	2.327694
225	O	0	-3.367570	9.522079	-1.979508
226	H	0	-4.298394	9.795555	-1.836604

227	O	0	3.891234	4.560151	5.506574
228	H	0	4.131159	4.357171	6.435613
229	O	0	7.232704	1.565177	5.536318
230	H	0	8.130222	1.281976	5.260224
231	O	0	-6.215303	5.987447	-0.039575
232	H	0	-6.686443	5.988259	0.820916
233	O	0	-11.553661	-0.649575	-3.177873
234	H	0	-12.278338	-0.032707	-3.410851
235	O	0	11.552583	-0.533728	1.580336
236	H	0	11.738050	0.360354	1.936269
237	C	0	6.046701	-0.437330	0.726630
238	O	0	7.291163	-1.967075	-0.704011
239	H	0	8.042261	-1.335495	-0.606668
240	H	0	6.971611	0.019245	1.066928
241	H	0	2.780079	-2.153198	-0.413221
242	C	0	4.912928	-2.144761	-0.541531
243	C	0	4.817454	0.029118	1.197185
244	H	0	4.803310	0.844866	1.907708
245	C	0	2.268344	-0.021728	1.191747
246	H	0	1.480323	-0.651906	0.766918
247	H	0	2.146550	-0.058443	2.280361
248	C	0	2.046855	1.430472	0.764031
249	H	0	1.003385	1.727051	0.868612
250	H	0	2.666110	2.118786	1.339745
251	O	0	4.989735	-3.262276	-1.350431
252	H	0	4.157001	-3.779970	-1.294123
253	H	0	2.229074	2.588273	-0.988157
254	H	0	3.448498	1.464346	-0.803035
255	H	0	1.930334	0.978757	-1.298148
256	N	0	2.436223	1.624933	-0.681696

Sult1a3: Overall Charge = +1, Total energy = -5484.73675126 Ha

1	N	0	-0.642445	6.371995	4.976955
2	C	0	-1.954096	5.783517	5.271110
3	C	0	-2.504212	6.302193	6.592003
4	O	0	-3.680242	6.658294	6.688989
5	C	0	-1.867134	4.256571	5.317404
6	C	0	-1.715987	3.603307	3.959668
7	C	0	-1.515334	4.334122	2.782477
8	C	0	-1.820519	2.206999	3.879747
9	C	0	-1.437402	3.681781	1.548287
10	C	0	-1.732019	1.554101	2.652892
11	C	0	-1.544889	2.292656	1.481055
12	H	0	-0.661161	7.367580	4.789192
13	H	0	0.071453	6.123847	5.653593
14	H	0	-2.673282	6.099776	4.512626
15	H	0	-1.028177	3.978213	5.969117
16	H	0	-2.772441	3.861173	5.794594
17	H	0	-1.399836	5.409240	2.830115
18	H	0	-1.981931	1.633905	4.788001
19	H	0	-1.313031	4.267112	0.641033
20	H	0	-1.822077	0.473769	2.608250
21	H	0	-1.489695	1.782148	0.528104
22	C	0	-1.626124	-7.147401	1.447449
23	C	0	-1.027399	-8.392284	0.812573
24	O	0	-0.584533	-9.290609	1.517193
25	C	0	-0.498007	-6.267406	2.065721
26	C	0	-1.087444	-5.801089	3.409182
27	C	0	-1.921224	-7.005999	3.847276
28	H	0	-2.132040	-6.599369	0.659401
29	H	0	-0.225115	-5.444816	1.403346
30	H	0	0.391142	-6.879148	2.246743
31	H	0	-1.735218	-4.930375	3.261724
32	H	0	-0.312607	-5.538499	4.131006
33	H	0	-1.275971	-7.788817	4.267829

34	H	0	-2.691696	-6.754122	4.577789
35	N	0	-8.178682	2.973116	-4.272580
36	C	0	-7.588842	2.417317	-3.073613
37	C	0	-7.985384	3.374028	-1.950338
38	O	0	-7.410894	4.450804	-1.804902
39	C	0	-6.085943	2.321062	-3.245914
40	C	0	-5.364000	1.601908	-2.135149
41	C	0	-6.024386	1.082376	-1.014802
42	C	0	-3.969210	1.466916	-2.224770
43	C	0	-5.297523	0.462216	0.008249
44	C	0	-3.247511	0.847563	-1.209299
45	C	0	-3.910970	0.351084	-0.082127
46	H	0	-7.824314	3.887846	-4.528898
47	H	0	-9.187549	2.910171	-4.333843
48	H	0	-8.032420	1.437894	-2.874972
49	H	0	-5.902531	1.822425	-4.204269
50	H	0	-5.684903	3.338643	-3.340105
51	H	0	-7.104519	1.157972	-0.915447
52	H	0	-3.450653	1.877506	-3.087124
53	H	0	-5.823182	0.075277	0.874511
54	H	0	-2.169763	0.764230	-1.285603
55	H	0	-3.338673	-0.109563	0.715836
56	N	0	-0.568731	8.661550	-1.414743
57	C	0	0.785040	8.747819	-1.948912
58	C	0	1.192950	10.151339	-2.412506
59	O	0	0.353117	10.955859	-2.826448
60	C	0	1.092486	7.722140	-3.060182
61	C	0	1.068414	6.272189	-2.560201
62	O	0	1.225083	5.342380	-3.392137
63	O	0	0.910174	6.086606	-1.275062
64	H	0	-0.707971	7.751973	-0.986271
65	H	0	-1.288423	8.887328	-2.094635
66	H	0	1.458997	8.496300	-1.122603

67	H	0	2.085997	7.896708	-3.491033
68	H	0	0.366841	7.818632	-3.876184
69	N	0	-11.123798	-0.589359	-3.062137
70	C	0	-10.063161	-1.455110	-2.558315
71	C	0	-9.564306	-0.974653	-1.193483
72	O	0	-9.539956	0.221026	-0.923374
73	C	0	-8.857460	-1.377271	-3.530674
74	C	0	-7.515595	-1.815396	-2.933760
75	C	0	-6.337151	-1.545696	-3.875309
76	C	0	-5.034371	-1.916309	-3.180405
77	N	0	-3.822739	-1.660159	-4.031062
78	H	0	-12.026487	-0.745144	-2.626295
79	H	0	-10.863034	0.392326	-3.027735
80	H	0	-10.421802	-2.481724	-2.468104
81	H	0	-8.780161	-0.330744	-3.857794
82	H	0	-9.097782	-1.966770	-4.420811
83	H	0	-7.549477	-2.878732	-2.665976
84	H	0	-7.319898	-1.265927	-2.002832
85	H	0	-6.449918	-2.119196	-4.803303
86	H	0	-6.325092	-0.481412	-4.146672
87	H	0	-5.014868	-2.976598	-2.917139
88	H	0	-4.896798	-1.338787	-2.259682
89	H	0	-3.894010	-2.111654	-4.946750
90	H	0	-2.990259	-2.050712	-3.506954
91	H	0	-3.663657	-0.659292	-4.176409
92	N	0	-7.080908	-2.500350	2.589015
93	C	0	-6.404340	-3.533914	3.366331
94	C	0	-6.677121	-3.287959	4.850445
95	O	0	-6.189482	-4.033460	5.701719
96	C	0	-4.884279	-3.590369	3.144499
97	C	0	-4.467886	-3.884300	1.744548
98	N	0	-4.073655	-5.135263	1.280421
99	C	0	-4.326992	-3.033423	0.676917

100	C	0	-3.716062	-5.006789	-0.026596
101	N	0	-3.840244	-3.739205	-0.415473
102	H	0	-8.078378	-2.486710	2.774299
103	H	0	-6.684580	-1.576957	2.739270
104	H	0	-6.857076	-4.505817	3.143440
105	H	0	-4.472391	-4.334764	3.832565
106	H	0	-4.460124	-2.622349	3.435240
107	H	0	-3.919897	-5.980291	1.849748
108	H	0	-4.536336	-1.981278	0.625647
109	H	0	-3.380520	-5.813468	-0.652592
110	N	0	7.804680	-7.228395	-2.066383
111	C	0	7.029016	-6.011574	-2.269655
112	C	0	7.670656	-4.876585	-1.474265
113	O	0	7.702972	-3.734791	-1.917291
114	C	0	5.555033	-6.177854	-1.838871
115	C	0	4.729581	-4.950337	-2.157196
116	C	0	4.226644	-4.764340	-3.452973
117	C	0	4.504627	-3.951778	-1.200123
118	C	0	3.518725	-3.608455	-3.787614
119	C	0	3.800390	-2.791986	-1.534312
120	C	0	3.305453	-2.614242	-2.828650
121	H	0	8.693774	-7.241516	-2.551029
122	H	0	7.899935	-7.488774	-1.091326
123	H	0	7.070626	-5.715663	-3.319574
124	H	0	5.524320	-6.402549	-0.766210
125	H	0	5.173873	-7.053248	-2.373083
126	H	0	4.394264	-5.532546	-4.202629
127	H	0	4.892798	-4.078049	-0.192495
128	H	0	3.137802	-3.483036	-4.795017
129	H	0	3.661284	-2.007220	-0.798854
130	H	0	2.778855	-1.699764	-3.081170
131	N	0	8.698554	-1.569981	-3.373305
132	C	0	7.786044	-0.481879	-3.036854

133	C	0	8.514439	0.448921	-2.077936
134	O	0	8.653654	0.154571	-0.891943
135	C	0	6.413375	-0.852517	-2.486204
136	C	0	5.468151	0.317715	-2.814352
137	C	0	4.271240	0.527231	-1.903648
138	O	0	3.151073	0.785062	-2.431894
139	O	0	4.490798	0.517111	-0.622280
140	H	0	9.578412	-1.278658	-3.778635
141	H	0	8.758818	-2.306127	-2.682004
142	H	0	7.628980	0.087883	-3.960113
143	H	0	6.463994	-1.034309	-1.407531
144	H	0	6.073969	-1.777215	-2.958791
145	H	0	6.037008	1.261956	-2.764737
146	H	0	5.107956	0.230022	-3.842381
147	N	0	7.751382	2.970849	-0.497252
148	C	0	6.992545	3.606923	0.569186
149	C	0	7.058226	2.803665	1.880891
150	O	0	6.796153	3.333428	2.965503
151	C	0	5.530208	3.782650	0.152749
152	H	0	7.222252	2.279084	-1.014518
153	H	0	8.674042	2.653091	-0.223619
154	H	0	7.428749	4.582800	0.803882
155	H	0	5.082660	2.802001	-0.055157
156	H	0	5.476392	4.399444	-0.747641
157	H	0	4.971156	4.271400	0.954348
158	N	0	7.417494	1.529151	1.781755
159	C	0	7.516725	0.688689	2.963244
160	C	0	8.897582	0.821162	3.583895
161	O	0	9.869682	1.116358	2.888327
162	C	0	7.247917	-0.794863	2.610318
163	C	0	5.789749	-1.099166	2.551590
164	N	0	4.928725	-0.675357	1.544426
165	C	0	5.015053	-1.760703	3.468396

166	C	0	3.680521	-1.054910	1.831258
167	H	0	7.734852	1.145400	0.896532
168	H	0	6.782015	1.032587	3.695969
169	H	0	7.691308	-1.430695	3.381040
170	H	0	7.745857	-1.034299	1.663516
171	H	0	5.096722	-0.154669	0.636550
172	H	0	5.280506	-2.240680	4.392498
173	H	0	2.811666	-0.858012	1.228048
174	C	0	-1.357328	-1.813970	-1.597161
175	C	0	-0.647017	-1.000940	-2.495913
176	C	0	0.411844	0.184988	-0.663207
177	C	0	-0.254858	-0.666002	0.226669
178	O	0	-2.290643	-2.675344	-2.127495
179	H	0	-0.121384	-0.528502	1.295351
180	H	0	-2.964117	-3.135220	-1.435182
181	O	0	-7.845602	-2.512021	5.127269
182	H	0	-8.042623	-2.541306	6.087309
183	O	0	-1.000836	-8.476930	-0.614496
184	H	0	-0.560113	-9.309475	-0.886025
185	O	0	8.130559	-5.228055	-0.242068
186	H	0	8.505046	-4.470215	0.253488
187	O	0	-1.649783	6.261641	7.645491
188	H	0	-2.059745	6.581673	8.475700
189	O	0	-9.113309	3.025130	-1.279639
190	H	0	-9.401944	3.717944	-0.647953
191	O	0	-9.154729	-1.964638	-0.349526
192	H	0	-8.724329	-1.638372	0.469960
193	O	0	2.534355	10.399095	-2.423818
194	H	0	2.742368	11.280554	-2.798301
195	O	0	8.950119	1.586089	-2.663675
196	H	0	9.453807	2.197007	-2.086847
197	O	0	8.956914	0.489863	4.901261
198	H	0	9.868504	0.514922	5.259993

199	N	0	-2.544144	-7.424043	2.571858
200	H	0	-2.968119	-8.346475	2.570051
201	N	0	3.712673	-1.719431	2.996535
202	H	0	2.910056	-2.125662	3.456177
203	C	0	-1.117721	-1.668685	-0.232517
204	H	0	-1.643846	-2.302253	0.473502
205	C	0	0.234204	-0.027140	-2.042351
206	H	0	0.792075	0.572537	-2.753706
207	O	0	-0.945508	-1.193663	-3.840112
208	H	0	-0.433527	-0.605982	-4.424873
209	C	0	1.238845	1.345805	-0.147057
210	H	0	0.984796	1.505808	0.908812
211	H	0	2.309998	1.119949	-0.217655
212	C	0	0.965048	2.635655	-0.932771
213	H	0	1.436174	2.590014	-1.915723
214	H	0	-0.108279	2.802166	-1.066859
215	H	0	2.534573	3.799554	-0.186235
216	H	0	1.221112	4.831605	-0.765394
217	H	0	1.155968	3.895636	0.724932
218	N	0	1.514090	3.842904	-0.234083

4. Optimized coordinates for L-DOPA in the active sites labelled below. Optimizations carried out with M062X/6-31G and implicit solvent using the PCM model in the Gaussian 16 software (see manuscript for references). Columns are: atom number, atom symbol, atom type (gaussian 16), and X, Y and Z coordinates in Angstroms.

Phenylalanine Hydroxylase: Overall Charge = -1, Total energy = -7476.95165221 Ha

1	N	0	2.937398	9.753503	0.029240
2	C	0	2.025847	9.666370	1.176442
3	C	0	2.598570	10.418481	2.376253
4	O	0	3.025981	9.902690	3.410061
5	C	0	1.653415	8.212177	1.536423
6	C	0	0.878713	7.611612	0.387782
7	C	0	1.557071	7.168680	-0.757087
8	C	0	-0.522169	7.606656	0.381638
9	C	0	0.857556	6.773216	-1.895620
10	C	0	-1.235780	7.195648	-0.745824
11	C	0	-0.535262	6.805495	-1.882697
12	O	0	-1.289612	6.383809	-2.986389
13	H	0	2.465105	9.904738	-0.851703
14	H	0	3.621969	8.993730	-0.024318
15	H	0	1.108290	10.204891	0.910947
16	H	0	2.582237	7.658091	1.713979
17	H	0	1.065519	8.191728	2.459345
18	H	0	2.641453	7.141777	-0.772936
19	H	0	-1.063540	7.939230	1.261864
20	H	0	1.403578	6.448305	-2.776425
21	H	0	-2.319252	7.188085	-0.758504
22	H	0	-0.780032	6.365510	-3.817856
23	N	0	6.724455	-0.926138	-4.764244
24	C	0	5.906580	-1.789439	-3.861467
25	C	0	6.168190	-1.304022	-2.450728

26	O	0	7.098948	-0.538456	-2.176722
27	C	0	6.198557	-3.289017	-4.041005
28	C	0	7.489001	-3.775474	-3.363490
29	C	0	7.251590	-4.344151	-1.958016
30	N	0	6.425191	-5.555219	-1.982618
31	C	0	5.122242	-5.667405	-1.657694
32	N	0	4.349764	-4.612249	-1.376606
33	N	0	4.573732	-6.893524	-1.619274
34	H	0	6.722618	-1.277476	-5.722486
35	H	0	7.684131	-0.827430	-4.424186
36	H	0	4.862245	-1.604232	-4.125513
37	H	0	6.226139	-3.483250	-5.117876
38	H	0	5.329843	-3.849932	-3.681580
39	H	0	8.227805	-2.966777	-3.290382
40	H	0	7.950680	-4.559260	-3.971618
41	H	0	8.207037	-4.617581	-1.504492
42	H	0	6.787025	-3.612980	-1.294844
43	H	0	6.917291	-6.416145	-2.179982
44	H	0	4.698822	-3.656371	-1.414623
45	H	0	3.341575	-4.747774	-1.205129
46	H	0	3.722778	-7.011316	-1.054691
47	H	0	5.121476	-7.708672	-1.846859
48	N	0	5.639900	-4.305742	5.501656
49	C	0	4.776342	-3.231927	5.047334
50	C	0	5.541088	-2.002220	4.557167
51	O	0	6.614808	-1.643915	5.102184
52	C	0	3.885107	-2.743260	6.232289
53	C	0	3.121076	-1.469241	5.933698
54	C	0	1.910660	-1.506770	5.228058
55	C	0	3.640864	-0.215937	6.287471
56	C	0	1.231339	-0.334605	4.893243

57	C	0	2.982561	0.967377	5.940905
58	C	0	1.776078	0.896276	5.244681
59	O	0	1.067572	2.041553	4.853910
60	H	0	6.351881	-3.971386	6.145653
61	H	0	6.056082	-4.841761	4.747357
62	H	0	4.129187	-3.605782	4.247082
63	H	0	3.205807	-3.561300	6.489284
64	H	0	4.552744	-2.583144	7.087455
65	H	0	1.498390	-2.467146	4.935837
66	H	0	4.582060	-0.160346	6.826282
67	H	0	0.269600	-0.375151	4.395036
68	H	0	3.398523	1.928782	6.225345
69	H	0	1.251265	2.819894	5.411583
70	N	0	4.958234	-1.324660	3.571957
71	C	0	5.514568	-0.087368	3.032914
72	C	0	4.339590	0.818231	2.667951
73	O	0	3.312932	0.351809	2.106402
74	C	0	6.325539	-0.299136	1.705858
75	O	0	5.437845	-0.497962	0.564540
76	C	0	7.234718	-1.510083	1.767787
77	H	0	4.079269	-1.630825	3.171153
78	H	0	6.174444	0.338535	3.788990
79	H	0	6.914587	0.609238	1.530581
80	H	0	4.938242	0.286256	0.221697
81	H	0	6.630811	-2.418248	1.845343
82	H	0	7.887662	-1.450580	2.641480
83	H	0	7.842736	-1.563698	0.861813
84	N	0	4.443064	2.114059	2.977614
85	C	0	3.338675	3.019343	2.654247
86	C	0	3.217463	3.231923	1.156186
87	O	0	2.100729	3.531964	0.660183

88	C	0	3.695997	4.322425	3.401175
89	C	0	5.227524	4.270013	3.529693
90	C	0	5.531624	2.780662	3.737539
91	H	0	2.398120	2.583682	2.989116
92	H	0	3.230280	4.309972	4.390014
93	H	0	3.340335	5.207424	2.870768
94	H	0	5.701129	4.628132	2.610117
95	H	0	5.598120	4.877945	4.355212
96	H	0	5.470204	2.497100	4.793523
97	H	0	6.514072	2.502725	3.351936
98	N	0	4.347035	3.149695	0.429206
99	C	0	4.385222	3.419173	-1.003586
100	C	0	3.930023	2.216865	-1.822882
101	O	0	4.085472	1.041333	-1.370020
102	C	0	5.765157	3.950788	-1.417911
103	C	0	6.063478	5.295632	-0.743710
104	C	0	5.007561	6.379462	-1.035482
105	O	0	4.369096	6.301517	-2.141199
106	O	0	4.852129	7.294561	-0.148632
107	H	0	5.208476	2.885731	0.887249
108	H	0	3.670328	4.230950	-1.184231
109	H	0	5.766320	4.077610	-2.504143
110	H	0	6.534216	3.207116	-1.172577
111	H	0	6.144312	5.188182	0.341849
112	H	0	7.034588	5.666244	-1.094960
113	N	0	3.418327	2.469734	-3.039031
114	C	0	2.962261	1.382335	-3.908600
115	C	0	4.106514	0.764006	-4.741105
116	O	0	3.815355	-0.099513	-5.614024
117	C	0	1.919548	2.067729	-4.809208
118	C	0	2.474752	3.496594	-4.939811

119	C	0	2.995622	3.804070	-3.531846
120	H	0	2.529992	0.572961	-3.308839
121	H	0	0.947347	2.076417	-4.305463
122	H	0	1.814220	1.545841	-5.759987
123	H	0	1.722591	4.220061	-5.259802
124	H	0	3.299419	3.517888	-5.660743
125	H	0	3.807212	4.532799	-3.492254
126	H	0	2.184017	4.178814	-2.892984
127	N	0	3.027021	-4.175494	-5.287710
128	C	0	2.038145	-4.143150	-4.225447
129	C	0	0.695605	-4.730719	-4.677372
130	O	0	0.540400	-5.390317	-5.706147
131	C	0	1.877406	-2.717518	-3.629382
132	C	0	0.801790	-1.862007	-4.220425
133	N	0	-0.439443	-1.734671	-3.614966
134	C	0	0.724772	-1.098418	-5.365482
135	C	0	-1.219963	-0.934858	-4.398016
136	N	0	-0.543304	-0.525570	-5.474315
137	H	0	2.817647	-3.540793	-6.054201
138	H	0	3.219521	-5.108902	-5.635234
139	H	0	2.401741	-4.773163	-3.411125
140	H	0	2.848413	-2.226914	-3.748963
141	H	0	1.685503	-2.827813	-2.559528
142	H	0	-0.673103	-2.203787	-2.739413
143	H	0	1.513945	-0.892919	-6.069358
144	H	0	-2.246841	-0.691640	-4.164785
145	N	0	-10.639397	2.652178	-2.223964
146	C	0	-9.746685	2.340356	-1.127556
147	C	0	-10.318667	1.274365	-0.197311
148	O	0	-9.966276	0.092266	-0.166039
149	C	0	-9.358969	3.583880	-0.293043

150	C	0	-8.446639	3.327860	0.870959
151	C	0	-8.815566	3.018109	2.160662
152	C	0	-7.005160	3.472022	0.883213
153	N	0	-7.695145	2.979196	2.974423
154	C	0	-6.572254	3.273635	2.222478
155	C	0	-6.048821	3.769108	-0.104338
156	C	0	-5.229787	3.404333	2.599326
157	C	0	-4.711611	3.883082	0.259244
158	C	0	-4.310408	3.711689	1.604019
159	H	0	-11.369780	3.320558	-2.014338
160	H	0	-10.190761	2.804845	-3.115336
161	H	0	-8.841418	1.895299	-1.548366
162	H	0	-10.290835	4.042922	0.056925
163	H	0	-8.889873	4.292509	-0.984682
164	H	0	-9.801141	2.853849	2.566846
165	H	0	-7.703288	2.776264	3.960458
166	H	0	-6.348220	3.904301	-1.139352
167	H	0	-4.919209	3.253558	3.627072
168	H	0	-3.969973	4.088225	-0.505691
169	H	0	-3.259820	3.802784	1.858502
170	N	0	-8.591036	-1.607402	-2.666405
171	C	0	-7.368552	-1.521505	-1.865160
172	C	0	-7.378355	-2.341978	-0.572918
173	O	0	-6.527561	-3.238068	-0.340601
174	C	0	-6.832017	-0.096521	-1.611109
175	C	0	-6.659198	0.663346	-2.940579
176	C	0	-5.813036	-0.185735	-3.885679
177	O	0	-6.389350	-0.871602	-4.778311
178	O	0	-4.533028	-0.222333	-3.643623
179	H	0	-8.371464	-1.461481	-3.649105
180	H	0	-9.333751	-0.990560	-2.344653

181	H	0	-6.600035	-2.034888	-2.454977
182	H	0	-7.504252	0.449744	-0.939431
183	H	0	-5.858001	-0.165446	-1.111320
184	H	0	-6.165641	1.620192	-2.749858
185	H	0	-7.631330	0.844429	-3.406739
186	N	0	-8.346667	-2.093704	0.304601
187	C	0	-8.374838	-2.819178	1.568183
188	C	0	-9.697703	-3.502586	1.864696
189	O	0	-9.992432	-3.956133	2.970496
190	C	0	-8.004534	-1.889590	2.756169
191	C	0	-6.624631	-1.300038	2.596195
192	C	0	-6.427749	-0.173864	1.785547
193	C	0	-5.513316	-1.883763	3.220269
194	C	0	-5.145003	0.331227	1.564467
195	C	0	-4.234308	-1.349934	3.042829
196	C	0	-4.044751	-0.249889	2.201200
197	H	0	-9.041080	-1.372894	0.127554
198	H	0	-7.618040	-3.607058	1.491033
199	H	0	-8.075614	-2.473232	3.677581
200	H	0	-8.753827	-1.089934	2.808431
201	H	0	-7.286122	0.312523	1.327387
202	H	0	-5.653831	-2.751659	3.857893
203	H	0	-5.011426	1.188150	0.912803
204	H	0	-3.386012	-1.793726	3.550183
205	H	0	-3.051074	0.162169	2.054265
206	N	0	-4.109896	-3.771364	-1.228202
207	C	0	-3.303991	-3.307500	-0.117258
208	C	0	-3.103608	-4.352007	0.960750
209	O	0	-3.361804	-5.549523	0.863054
210	H	0	-5.107065	-3.768451	-1.048754
211	H	0	-3.743976	-4.594643	-1.689194

212	H	0	-3.776855	-2.435532	0.349228
213	H	0	-2.301181	-3.000095	-0.445670
214	N	0	-0.782508	-7.462134	-0.615010
215	C	0	0.644152	-7.233857	-0.865165
216	C	0	1.481620	-6.964637	0.394627
217	O	0	2.720170	-6.761576	0.316725
218	C	0	1.053492	-6.233256	-1.944993
219	O	0	1.457642	-4.957611	-1.440037
220	H	0	-1.180598	-8.242180	-1.117920
221	H	0	-1.378768	-6.641112	-0.622329
222	H	0	1.019110	-8.206682	-1.215805
223	H	0	1.913779	-6.635128	-2.489889
224	H	0	0.217468	-6.152638	-2.637009
225	H	0	0.718036	-4.282707	-1.279234
226	N	0	0.824968	-6.950244	1.551941
227	C	0	1.306997	-6.260894	2.729606
228	C	0	1.640836	-7.177562	3.885143
229	O	0	1.413292	-6.884905	5.063913
230	C	0	0.234429	-5.240578	3.129905
231	O	0	0.647015	-4.365357	4.197851
232	H	0	-0.160874	-7.203227	1.483186
233	H	0	2.228665	-5.732494	2.453533
234	H	0	-0.678932	-5.779294	3.412135
235	H	0	0.056103	-4.597649	2.267099
236	H	0	0.786768	-4.880485	5.019006
237	O	0	-0.739079	-2.146850	3.302671
238	H	0	-1.442735	-2.552058	2.751725
239	H	0	-0.228048	-2.852526	3.768210
240	O	0	0.182066	2.089962	2.176886
241	H	0	0.674551	2.722485	1.603552
242	H	0	0.346409	2.245369	3.133607

243	O	0	5.350495	-1.838675	-1.532260
244	H	0	5.453898	-1.387496	-0.590300
245	O	0	2.594409	11.765457	2.187339
246	H	0	2.987841	12.247228	2.944477
247	O	0	-0.338247	-4.469174	-3.833299
248	H	0	-1.192971	-4.816135	-4.166365
249	O	0	2.241936	-8.322175	3.510250
250	H	0	2.481537	-8.896744	4.267786
251	O	0	-11.320243	1.743730	0.579249
252	H	0	-11.721595	1.057914	1.154162
253	O	0	5.323410	1.109015	-4.430993
254	H	0	6.189964	0.116289	-4.729335
255	O	0	-10.525901	-3.586241	0.794145
256	H	0	-11.363303	-4.048678	1.008455
257	O	0	-2.558555	-3.853397	2.125177
258	H	0	-2.535484	-4.537529	2.827947
259	C	0	-0.264997	1.023517	-1.279045
260	C	0	0.260388	2.303342	-1.442668
261	H	0	1.299725	2.497857	-1.206912
262	C	0	-1.905499	3.152027	-2.127757
263	C	0	-2.456169	1.861821	-1.975266
264	C	0	-0.556348	3.359501	-1.858264
265	H	0	-0.132135	4.350658	-1.964144
266	C	0	-1.628272	0.818617	-1.552882
267	H	0	-2.074515	-0.168274	-1.445312
268	O	0	-3.785484	1.686675	-2.235812
269	H	0	-4.047748	0.819246	-2.765252
270	O	0	-2.750794	4.165944	-2.534936
271	H	0	-2.263868	5.011817	-2.706180
272	C	0	0.635942	-0.114380	-0.852269
273	H	0	1.623673	0.281250	-0.578870

274	H	0	0.793273	-0.783564	-1.704594
275	C	0	0.088077	-0.959324	0.313776
276	H	0	-1.001612	-0.855943	0.364676
277	H	0	0.407698	0.519994	1.807010
278	H	0	0.199252	-1.109415	2.373510
279	H	0	1.650840	-0.599003	1.668831
280	N	0	0.627228	-0.502417	1.625299
281	C	0	0.387565	-2.447065	0.132014
282	O	0	-0.104351	-2.970153	-0.952175
283	O	0	1.056951	-3.053708	1.010467

Tyrosine Hydroxylase: Overall Charge = 0, Total energy = -12020.0897526 Ha

1	C	0	3.369568	-9.247499	2.878543
2	O	0	5.022308	-7.970371	1.566763
3	C	0	2.073603	-8.451999	2.632470
4	C	0	2.302313	-6.940481	2.746147
5	C	0	1.449800	-8.866589	1.298611
6	H	0	3.112192	-10.313141	2.814795
7	H	0	1.397109	-8.761418	3.441407
8	H	0	2.776402	-6.541726	1.839003
9	H	0	2.966078	-6.703302	3.580760
10	H	0	1.360713	-6.412294	2.935481
11	H	0	0.436386	-8.469105	1.222753
12	H	0	1.363696	-9.956445	1.224290
13	H	0	2.049725	-8.516766	0.446624
14	C	0	3.739000	-7.100105	-2.278092
15	C	0	2.447551	-7.299990	-3.032907
16	O	0	2.224834	-8.411513	-3.556696
17	H	0	4.534591	-7.029374	-3.038827
18	H	0	3.720068	-6.149067	-1.737563

19	C	0	0.292923	-6.316032	-3.676319
20	C	0	-0.458758	-7.645083	-3.709067
21	O	0	-0.292659	-8.523949	-4.579791
22	C	0	0.340435	-5.717435	-5.096111
23	C	0	0.308160	-4.181920	-5.015838
24	C	0	0.568281	-3.539968	-6.380099
25	C	0	-1.051824	-3.764243	-4.437718
26	H	0	-0.310798	-5.655047	-3.063301
27	H	0	-0.526034	-6.058613	-5.676275
28	H	0	1.239489	-6.085577	-5.602500
29	H	0	1.102448	-3.855226	-4.327159
30	H	0	0.515603	-2.448750	-6.313922
31	H	0	-0.183911	-3.872665	-7.104711
32	H	0	1.556524	-3.813561	-6.762651
33	H	0	-1.832349	-3.868944	-5.199678
34	H	0	-1.050480	-2.731958	-4.078888
35	H	0	-1.306104	-4.433197	-3.602711
36	C	0	-2.419879	-8.817948	-2.648991
37	C	0	-3.726287	-8.601775	-3.470584
38	O	0	-3.982862	-7.488503	-3.981965
39	C	0	-2.798372	-8.933329	-1.162721
40	C	0	-1.698838	-9.512431	-0.263855
41	C	0	-2.111177	-9.349625	1.206072
42	C	0	-1.424607	-10.988595	-0.575920
43	H	0	-1.932125	-9.728533	-3.010052
44	H	0	-3.698082	-9.555045	-1.064676
45	H	0	-3.080121	-7.930738	-0.808750
46	H	0	-0.774871	-8.947475	-0.457536
47	H	0	-3.055754	-9.876699	1.385926
48	H	0	-2.267506	-8.297475	1.475094
49	H	0	-1.359393	-9.771014	1.880198

50	H	0	-2.335232	-11.580398	-0.425684
51	H	0	-0.650860	-11.382542	0.090098
52	H	0	-1.083303	-11.138364	-1.604132
53	C	0	-5.852521	-9.442594	-4.306176
54	O	0	-6.453043	-8.201436	-2.283383
55	C	0	-6.523126	-10.793349	-4.505868
56	H	0	-5.670185	-8.956086	-5.269684
57	H	0	-5.910987	-11.400158	-5.178803
58	H	0	-7.515515	-10.668653	-4.949863
59	C	0	-6.449840	-7.435120	1.441347
60	C	0	-5.944607	-6.310164	0.502164
61	C	0	-5.814004	-4.972330	1.200310
62	C	0	-6.532671	-3.859659	0.744379
63	C	0	-6.480528	-2.639809	1.429000
64	C	0	-5.036462	-4.837437	2.361348
65	C	0	-5.716086	-2.526286	2.590283
66	C	0	-7.538904	-6.878744	2.333494
67	O	0	-7.337178	-6.607130	3.546287
68	H	0	-5.637702	-7.733113	2.116868
69	H	0	-6.655714	-6.226073	-0.327553
70	H	0	-4.988198	-6.632153	0.072544
71	H	0	-7.150265	-3.954342	-0.144410
72	H	0	-7.044346	-1.786443	1.062374
73	H	0	-4.484479	-5.693241	2.739073
74	H	0	-5.669569	-1.581759	3.120881
75	C	0	-9.826695	-6.201956	2.536623
76	O	0	-10.734732	-6.902033	4.670549
77	C	0	-11.035060	-5.898225	1.638840
78	C	0	-10.748319	-4.831008	0.566282
79	C	0	-12.014437	-4.599654	-0.268284
80	C	0	-10.258545	-3.516303	1.186119

81	H	0	-9.526878	-5.300352	3.078278
82	H	0	-11.365529	-6.828205	1.157555
83	H	0	-11.855815	-5.552138	2.279615
84	H	0	-9.960911	-5.210914	-0.099782
85	H	0	-11.823445	-3.881356	-1.070901
86	H	0	-12.816312	-4.198754	0.362665
87	H	0	-12.369026	-5.531921	-0.718948
88	H	0	-10.130036	-2.753430	0.410794
89	H	0	-9.294945	-3.635276	1.691067
90	H	0	-10.989114	-3.141556	1.913649
91	C	0	5.624509	-4.725837	3.129956
92	O	0	6.721275	-3.652213	5.043383
93	C	0	5.871463	-3.926543	1.812741
94	C	0	4.828545	-4.206576	0.742833
95	H	0	5.680214	-5.791872	2.882954
96	H	0	6.855141	-4.235898	1.432380
97	H	0	5.104995	-3.685958	-0.181950
98	H	0	3.840110	-3.861547	1.056116
99	H	0	4.786470	-5.282234	0.534846
100	C	0	11.454458	-4.140780	1.738155
101	O	0	10.538285	-1.842045	1.839114
102	C	0	11.548396	-4.910244	0.383569
103	C	0	10.265168	-4.820257	-0.409515
104	C	0	10.037561	-3.766171	-1.309984
105	C	0	9.245923	-5.765366	-0.229427
106	C	0	8.840050	-3.654964	-2.014563
107	C	0	8.035339	-5.665259	-0.918483
108	C	0	7.841023	-4.609495	-1.809086
109	H	0	12.391087	-4.305043	2.282103
110	H	0	11.766058	-5.956177	0.623999
111	H	0	12.388638	-4.516605	-0.192892

112	H	0	10.817813	-3.027075	-1.469171
113	H	0	9.402106	-6.598607	0.449259
114	H	0	8.675898	-2.847907	-2.725660
115	H	0	7.258598	-6.409500	-0.768819
116	C	0	12.292238	1.213764	-2.272965
117	O	0	14.691379	1.716318	-1.970502
118	C	0	11.538898	2.496966	-2.733578
119	C	0	10.294647	2.833839	-1.918047
120	C	0	9.190583	1.751289	-1.941023
121	H	0	11.899394	0.342521	-2.802997
122	H	0	12.216834	3.356838	-2.666234
123	H	0	11.278742	2.374272	-3.789954
124	H	0	9.887001	3.765500	-2.324030
125	H	0	10.610789	3.046903	-0.888803
126	H	0	9.395416	0.989725	-2.700282
127	H	0	9.116158	1.239198	-0.972573
128	C	0	4.755984	0.042776	-5.282847
129	C	0	4.843557	0.500516	-3.811281
130	O	0	5.575194	1.470535	-3.465931
131	C	0	5.509528	-1.328008	-5.387474
132	C	0	6.992648	-1.248166	-5.823756
133	O	0	7.942529	0.775841	-4.896220
134	H	0	3.699913	-0.105222	-5.524715
135	H	0	5.461221	-1.847640	-4.423519
136	H	0	4.990335	-1.951917	-6.124194
137	H	0	7.037763	-0.764533	-6.803733
138	H	0	7.357168	-2.273639	-5.929896
139	N	0	3.913990	0.004959	-2.989717
140	C	0	3.927897	0.364272	-1.581508
141	O	0	6.001933	-0.909033	-1.724141
142	C	0	2.682009	-0.342739	-1.019232

143	C	0	2.560666	-1.588506	-1.913866
144	C	0	2.961246	-1.077373	-3.299137
145	H	0	3.922758	1.447965	-1.433450
146	H	0	2.803177	-0.591180	0.035369
147	H	0	1.797765	0.295471	-1.132691
148	H	0	1.562170	-2.018224	-1.894561
149	H	0	3.269844	-2.353875	-1.578107
150	H	0	3.418652	-1.852794	-3.915138
151	H	0	2.096157	-0.672148	-3.839773
152	C	0	4.760527	4.089188	2.593840
153	C	0	3.894371	3.464251	3.694756
154	O	0	2.914520	4.092540	4.168554
155	C	0	4.290462	3.446656	1.254939
156	C	0	2.806143	3.461710	1.092338
157	C	0	1.852416	2.465883	1.132740
158	C	0	0.784070	4.366507	0.912519
159	N	0	0.588765	3.042953	1.007591
160	H	0	4.536437	5.158107	2.602491
161	H	0	4.646807	2.409230	1.216845
162	H	0	4.771781	3.983288	0.429759
163	H	0	1.990280	1.403926	1.247684
164	H	0	0.001341	5.103443	0.805436
165	C	0	3.396662	1.515461	5.125422
166	O	0	2.765395	2.477200	7.273247
167	C	0	3.881963	0.049196	5.198575
168	C	0	3.925842	-0.674370	3.852944
169	O	0	1.643100	-1.431272	4.211279
170	H	0	2.331173	1.592030	4.899810
171	H	0	4.889703	0.062530	5.626243
172	H	0	3.235546	-0.496359	5.891649
173	H	0	4.746889	-1.402537	3.815499

174	H	0	4.138042	0.001920	3.015058
175	C	0	-0.633191	3.984187	7.095063
176	O	0	0.125675	4.281779	9.352387
177	C	0	-0.932137	2.715703	6.270796
178	C	0	-1.429845	3.056152	4.902409
179	C	0	-0.798025	3.082366	3.688948
180	C	0	-2.889347	3.608937	3.292522
181	N	0	-1.716010	3.410932	2.692122
182	H	0	-1.491396	4.654371	6.905136
183	H	0	-1.667745	2.101427	6.797424
184	H	0	-0.012464	2.127244	6.184560
185	H	0	0.230632	2.864693	3.464289
186	H	0	-3.819755	3.841250	2.802028
187	C	0	-10.726937	2.494776	0.221772
188	O	0	-8.893902	4.139922	0.265457
189	C	0	-10.136506	1.059689	0.193752
190	C	0	-8.744432	0.998172	0.789058
191	C	0	-8.556071	0.810246	2.163196
192	C	0	-7.609733	1.213320	-0.009606
193	C	0	-7.280436	0.854137	2.730472
194	C	0	-6.328200	1.271582	0.538775
195	C	0	-6.176843	1.096627	1.913537
196	H	0	-10.811364	2.818348	1.267928
197	H	0	-10.123054	0.724211	-0.849476
198	H	0	-10.816820	0.401967	0.746124
199	H	0	-9.414735	0.635766	2.803614
200	H	0	-7.740099	1.356214	-1.078128
201	H	0	-7.153362	0.712013	3.798729
202	H	0	-5.453373	1.479121	-0.068948
203	C	0	-8.672966	4.212480	-2.467749
204	O	0	-7.868068	6.482710	-2.186438

205	C	0	-8.706859	3.939635	-3.991995
206	C	0	-7.378520	4.205302	-4.623954
207	C	0	-7.047499	5.163252	-5.549070
208	C	0	-6.174382	3.446241	-4.363226
209	C	0	-5.150146	3.992220	-5.181894
210	C	0	-5.879086	2.348394	-3.535155
211	C	0	-3.851171	3.465241	-5.194663
212	C	0	-4.590445	1.828725	-3.537022
213	C	0	-3.586451	2.383366	-4.362491
214	H	0	-7.672651	3.955260	-2.096429
215	H	0	-8.969015	2.881128	-4.115681
216	H	0	-9.500759	4.527181	-4.463774
217	H	0	-7.665538	5.923847	-5.998770
218	H	0	-6.650782	1.899996	-2.917592
219	H	0	-3.081646	3.882344	-5.834524
220	H	0	-4.349744	0.981783	-2.904369
221	H	0	-2.591263	1.954378	-4.348817
222	C	0	-5.158087	5.690419	0.434843
223	C	0	-5.040395	7.148791	0.053926
224	O	0	-3.955702	7.776327	0.125024
225	C	0	-5.192746	4.841496	-0.853002
226	C	0	-3.807045	4.727430	-1.476179
227	O	0	-3.348215	2.997609	0.180073
228	H	0	-4.245700	5.460396	0.999220
229	H	0	-5.556823	3.843959	-0.584180
230	H	0	-5.895483	5.285764	-1.566880
231	H	0	-3.357952	5.711245	-1.643791
232	H	0	-3.866424	4.229509	-2.453230
233	C	0	-6.049318	9.187910	-0.792183
234	O	0	-7.128857	11.343049	-0.491104
235	C	0	-6.066646	9.322472	-2.344042

236	C	0	-4.817819	8.763525	-2.977947
237	C	0	-4.774233	7.454157	-3.473988
238	C	0	-3.665629	9.557945	-3.055334
239	C	0	-3.593156	6.955041	-4.033116
240	C	0	-2.488848	9.060679	-3.613071
241	C	0	-2.449590	7.752939	-4.103681
242	H	0	-5.086050	9.556160	-0.426545
243	H	0	-6.164850	10.384700	-2.586655
244	H	0	-6.953406	8.798556	-2.714997
245	H	0	-5.664897	6.830521	-3.420529
246	H	0	-3.689782	10.570245	-2.662437
247	H	0	-3.562859	5.935957	-4.399878
248	H	0	-1.604883	9.686534	-3.652496
249	H	0	-1.536922	7.359003	-4.536683
250	C	0	-0.816708	7.589149	-0.384260
251	O	0	0.733063	9.409791	-0.004505
252	H	0	0.081808	6.976413	-0.486612
253	H	0	-1.490634	7.379951	-1.217461
254	C	0	3.888767	9.098021	2.093989
255	C	0	4.415541	9.652469	0.766170
256	O	0	5.399534	9.092527	0.222948
257	C	0	3.825393	7.577046	1.947760
258	H	0	4.605859	9.362087	2.876159
259	H	0	4.798750	7.170761	1.676094
260	H	0	3.468858	7.132295	2.882726
261	C	0	4.305621	11.248431	-0.993130
262	O	0	3.657655	13.515419	-0.461243
263	C	0	3.297646	10.940530	-2.107142
264	H	0	5.258249	10.771283	-1.213518
265	H	0	3.238132	9.854877	-2.235318
266	H	0	2.312731	11.316451	-1.806814

267	Fe	0	-1.425269	2.662924	0.857827
268	C	0	-1.923926	-3.134558	1.742873
269	C	0	-1.166209	-2.354355	2.622352
270	C	0	-2.858613	-2.533314	0.898661
271	C	0	-1.287384	-0.965585	2.620613
272	C	0	-3.004535	-1.149245	0.912182
273	C	0	-2.208432	-0.377708	1.749910
274	O	0	-2.327403	1.011745	1.738721
275	H	0	-0.423086	-2.814948	3.265691
276	H	0	-3.473421	-3.142753	0.243547
277	H	0	-3.727781	-0.656044	0.271135
278	H	0	-3.285902	1.278839	1.820411
279	N	0	12.317202	0.929836	-0.830526
280	H	0	11.396290	0.891695	-0.406122
281	H	0	12.948204	1.537678	-0.315791
282	C	0	13.758104	1.453180	-2.725074
283	O	0	13.872465	1.414824	-4.075231
284	H	0	14.775738	1.628542	-4.389901
285	C	0	4.497326	12.741444	-0.921664
286	O	0	5.684733	13.143930	-1.427002
287	H	0	5.790098	14.118564	-1.420060
288	N	0	2.574829	9.655887	2.444473
289	H	0	1.807730	9.521613	1.793975
290	H	0	2.318914	9.596750	3.419039
291	C	0	-0.390023	9.044564	-0.408136
292	O	0	-1.337922	9.927748	-0.771210
293	H	0	-1.046762	10.860356	-0.681034
294	N	0	-1.535371	7.323717	0.870158
295	H	0	-2.531641	7.508167	0.828324
296	H	0	-1.035233	7.574557	1.713415
297	N	0	-8.709300	-6.684647	1.763058

298	H	0	-8.860762	-7.118513	0.858674
299	N	0	-6.970913	-8.552475	0.639980
300	H	0	-6.614772	-8.601815	-0.308923
301	H	0	-6.991568	-9.444871	1.116542
302	C	0	-10.200465	-7.205300	3.603899
303	O	0	-9.961807	-8.492819	3.243581
304	H	0	-10.237190	-9.128960	3.935808
305	O	0	-6.600538	-11.376741	-3.189673
306	H	0	-6.964328	-12.280323	-3.212980
307	N	0	-4.563336	-9.641916	-3.610333
308	H	0	-4.451195	-10.516207	-3.111652
309	N	0	-1.484968	-7.700306	-2.854049
310	H	0	-1.718481	-6.880666	-2.311686
311	N	0	1.610565	-6.271278	-3.047635
312	H	0	1.894643	-5.383310	-2.655992
313	N	0	3.978025	-8.166940	-1.294956
314	H	0	4.063336	-9.081826	-1.724080
315	H	0	4.717486	-7.942139	-0.638589
316	C	0	-6.711879	-8.510225	-3.438443
317	O	0	-7.843353	-8.030759	-4.019830
318	H	0	-7.973368	-8.259384	-4.960529
319	O	0	-4.882998	1.169948	2.439707
320	H	0	-4.857157	1.084987	3.410988
321	N	0	-11.995464	2.545973	-0.520914
322	H	0	-12.564954	1.713865	-0.416779
323	H	0	-12.531914	3.392660	-0.370476
324	N	0	-5.709553	5.039958	-5.890704
325	H	0	-5.224157	5.631109	-6.545921
326	N	0	-9.631567	3.416047	-1.759856
327	H	0	-10.409449	2.972565	-2.238328
328	O	0	3.782672	11.605064	-3.287745

329	H	0	3.166452	11.502997	-4.035061
330	O	0	2.923008	7.226480	0.858862
331	H	0	2.178927	7.858885	0.751269
332	N	0	3.808123	10.717145	0.255300
333	H	0	3.023790	11.135565	0.742493
334	N	0	2.103278	4.646960	0.951480
335	H	0	2.494542	5.604402	0.878284
336	N	0	6.180573	3.767670	2.796042
337	H	0	6.703987	3.570327	1.952222
338	H	0	6.676505	4.371188	3.438886
339	N	0	4.174335	2.204630	4.081118
340	H	0	5.083201	1.839677	3.814130
341	C	0	3.651330	2.173592	6.471383
342	O	0	4.968558	2.365448	6.716128
343	H	0	5.140778	2.772464	7.590727
344	C	0	2.676298	-1.427803	3.493599
345	O	0	2.789873	-2.096106	2.368587
346	N	0	-2.751365	3.407136	4.630515
347	H	0	-3.493775	3.489137	5.309772
348	N	0	0.543474	4.674515	6.548178
349	H	0	0.745085	5.554197	7.010933
350	H	0	1.368142	4.080937	6.485852
351	C	0	-0.686893	3.825292	8.547038
352	O	0	-1.809181	3.162923	8.953667
353	H	0	-1.874626	3.106750	9.929042
354	O	0	6.667391	-4.466758	-2.538746
355	H	0	6.014418	-5.158662	-2.332150
356	N	0	10.345132	-4.628123	2.571643
357	H	0	10.558475	-5.547240	2.952374
358	H	0	9.474068	-4.657941	2.045853
359	C	0	11.401886	-2.620064	1.440442

360	O	0	12.424314	-2.230875	0.629698
361	H	0	12.380545	-1.285464	0.340537
362	C	0	4.374785	-9.000221	1.767819
363	O	0	4.497078	-10.099071	0.973516
364	H	0	5.160550	-9.989831	0.261665
365	N	0	3.926981	-8.877284	4.183129
366	H	0	3.217586	-8.686756	4.881429
367	H	0	4.641955	-9.505510	4.530699
368	N	0	7.878467	2.284431	-2.312050
369	H	0	7.405292	1.826442	-3.105226
370	C	0	7.250380	3.262855	-1.663163
371	N	0	6.030698	3.657108	-2.062169
372	H	0	5.594340	4.485285	-1.691637
373	H	0	5.529487	3.042836	-2.706113
374	N	0	7.824094	3.884276	-0.608481
375	H	0	7.380360	4.678294	-0.173984
376	H	0	8.753210	3.654906	-0.297235
377	N	0	5.342471	0.970514	-6.261451
378	H	0	6.307586	1.177445	-5.979003
379	H	0	4.800856	1.828687	-6.338128
380	C	0	7.958116	-0.512328	-4.879069
381	O	0	8.739030	-1.213901	-4.151265
382	C	0	5.225077	-0.243468	-0.982421
383	O	0	5.345516	-0.108346	0.300000
384	C	0	6.793039	-4.362930	4.041194
385	O	0	7.951004	-4.873800	3.568739
386	H	0	8.786578	-4.474870	3.913991
387	N	0	4.348776	-4.445227	3.808288
388	H	0	4.361169	-3.524836	4.239530
389	H	0	3.550135	-4.545043	3.187665
390	O	0	5.926882	-2.529911	2.187042

391	H	0	5.685025	-1.901250	1.464088
392	C	0	-2.897302	3.941080	-0.591901
393	N	0	-6.346570	5.384416	1.244024
394	H	0	-7.156019	5.014121	0.752842
395	H	0	-6.566548	6.038465	1.982812
396	N	0	-6.139419	7.786157	-0.390758
397	H	0	-6.999768	7.281290	-0.565897
398	C	0	-7.114046	10.139303	-0.241903
399	O	0	-8.050343	9.527282	0.525201
400	H	0	-8.727858	10.150220	0.862807
401	O	0	-1.625601	4.170298	-0.546458
402	C	0	-9.681272	3.442776	-0.417572
403	C	0	-8.814034	5.690132	-2.192093
404	O	0	-10.096114	6.080868	-2.025433
405	H	0	-10.187098	7.045456	-1.876164
406	C	0	-4.992580	-3.628314	3.053863
407	H	0	-4.385248	-3.541366	3.947986
408	H	0	2.077466	-2.872884	2.054959
409	O	0	-0.534996	-0.150110	3.425955
410	H	0	0.221624	-0.639686	3.844128
411	C	0	-1.559845	-4.584188	1.550018
412	H	0	-2.434653	-5.188490	1.288386
413	H	0	-1.107493	-4.994864	2.459889
414	C	0	-0.527202	-4.646621	0.402046
415	H	0	-1.004559	-4.490316	-0.563331
416	H	0	0.693424	-6.171757	-0.436662
417	H	0	0.855451	-5.936967	1.202668
418	H	0	-0.484735	-6.752215	0.579370
419	N	0	0.171651	-5.980550	0.427108
420	C	0	0.545129	-3.585400	0.629497
421	O	0	1.373295	-3.907778	1.592326

422	O	0	0.518002	-2.523296	-0.013825

Tyrosinase: Overall Charge = +2, Total energy = -9142.63968902 Ha

1	C	0	5.994174	2.929714	6.083343
2	O	0	4.079384	2.099840	7.251987
3	C	0	6.226891	2.191471	4.740182
4	C	0	4.929129	1.856065	4.075064
5	N	0	4.291906	0.619200	4.134967
6	C	0	4.060791	2.674698	3.393871
7	C	0	3.094029	0.705563	3.514401
8	N	0	2.923735	1.950773	3.053259
9	H	0	6.827471	1.292720	4.913022
10	H	0	6.808239	2.862128	4.102814
11	H	0	4.643821	-0.234900	4.552154
12	H	0	4.169280	3.714567	3.135927
13	H	0	2.409238	-0.118480	3.414328
14	H	0	5.307637	3.759437	5.897466
15	C	0	-3.796187	4.404067	4.891984
16	O	0	-5.267130	4.987141	6.645025
17	C	0	-3.446230	2.888000	4.945615
18	C	0	-2.040042	2.636238	4.517454
19	N	0	-1.038414	2.311844	5.419958
20	C	0	-1.424894	2.672671	3.293531
21	C	0	0.130800	2.155387	4.762018
22	N	0	-0.071337	2.366649	3.457883
23	H	0	-3.817389	4.718600	3.845985
24	H	0	-3.566711	2.549368	5.981725
25	H	0	-4.135075	2.293159	4.334921
26	H	0	-1.162496	2.218723	6.417745
27	H	0	-1.846415	2.895717	2.327207
28	H	0	1.069549	1.920178	5.232782
29	C	0	2.536604	8.844906	-0.067770

30	O	0	4.350060	9.513394	-1.446259
31	C	0	2.474559	7.448087	-0.724371
32	C	0	2.107590	6.379987	0.255250
33	N	0	1.313365	6.651609	1.359474
34	C	0	2.395188	5.037415	0.323177
35	C	0	1.134639	5.519926	2.066265
36	N	0	1.778253	4.512464	1.458916
37	H	0	3.192025	8.788590	0.809084
38	H	0	1.745860	7.482956	-1.545118
39	H	0	3.446685	7.219218	-1.165622
40	H	0	0.935219	7.583022	1.529554
41	H	0	2.973660	4.418644	-0.344614
42	H	0	0.555006	5.449762	2.970826
43	C	0	-11.935079	0.365611	-0.636346
44	O	0	-14.282497	-0.133141	-0.497533
45	C	0	-11.817973	0.969131	0.779350
46	C	0	-10.405286	0.918485	1.311962
47	C	0	-9.908775	-0.246085	1.910670
48	C	0	-9.566264	2.037293	1.219125
49	C	0	-8.607075	-0.288080	2.414862
50	C	0	-8.261113	1.996175	1.715996
51	C	0	-7.773998	0.831965	2.320739
52	H	0	-11.715106	-0.706464	-0.589218
53	H	0	-12.500100	0.423521	1.437404
54	H	0	-12.161189	2.010349	0.741266
55	H	0	-10.550915	-1.117455	1.997393
56	H	0	-9.943147	2.952309	0.772260
57	H	0	-8.260957	-1.193595	2.902616
58	H	0	-7.630877	2.876013	1.649778
59	H	0	-6.765514	0.813062	2.725371
60	C	0	-4.436890	0.951846	-5.426481

61	C	0	-4.281737	-0.346790	-4.632870
62	O	0	-3.253571	-1.001257	-4.730550
63	C	0	-4.398149	2.179352	-4.480920
64	C	0	-3.035759	2.382115	-3.921543
65	N	0	-2.488042	1.571263	-2.938167
66	C	0	-2.011134	3.205221	-4.323093
67	C	0	-1.175170	1.911749	-2.783204
68	N	0	-0.852322	2.902300	-3.610849
69	H	0	-3.529105	0.985250	-6.045070
70	H	0	-4.662270	3.064092	-5.068127
71	H	0	-5.157042	2.068983	-3.698681
72	H	0	-2.968594	0.847067	-2.422004
73	H	0	-2.031738	3.972551	-5.077698
74	H	0	-0.506917	1.352219	-2.149481
75	N	0	-5.296422	-0.705442	-3.848034
76	C	0	-5.276914	-1.961060	-3.089267
77	O	0	-4.310495	-4.074967	-3.751217
78	C	0	-6.584305	-2.013944	-2.260484
79	C	0	-6.599695	-0.943683	-1.171027
80	O	0	-5.582842	-0.831287	-0.419948
81	H	0	-6.176102	-0.213728	-3.966122
82	H	0	-4.409610	-1.978693	-2.424252
83	H	0	-6.647585	-2.982713	-1.753163
84	H	0	-7.448256	-1.931358	-2.925749
85	C	0	-0.903696	-4.545113	-4.107435
86	O	0	-0.285227	-6.831550	-4.110784
87	C	0	-1.094416	-3.965267	-2.669988
88	C	0	-0.308590	-2.711345	-2.481286
89	N	0	0.997040	-2.733598	-2.013363
90	C	0	-0.576762	-1.386201	-2.751702
91	C	0	1.465792	-1.452661	-2.003859

92	N	0	0.537822	-0.604936	-2.445456
93	H	0	0.155531	-4.480521	-4.368196
94	H	0	-0.763705	-4.701392	-1.929035
95	H	0	-2.164577	-3.784355	-2.530397
96	H	0	1.480548	-3.595623	-1.745255
97	H	0	-1.480781	-0.967188	-3.164865
98	H	0	2.457787	-1.180571	-1.682299
99	C	0	3.672947	-6.132098	-1.907265
100	C	0	2.241608	-6.316904	-1.439842
101	O	0	1.457666	-5.367804	-1.402996
102	C	0	3.734573	-5.955688	-3.432655
103	C	0	4.836745	-4.972079	-3.823798
104	S	0	4.421093	-3.198776	-3.341385
105	C	0	3.151432	-2.818773	-4.660972
106	H	0	3.959322	-5.161700	-1.474205
107	H	0	2.766133	-5.602099	-3.804425
108	H	0	3.914784	-6.929868	-3.904922
109	H	0	5.767005	-5.195335	-3.295360
110	H	0	5.029151	-4.982215	-4.898162
111	H	0	3.615172	-2.875256	-5.645857
112	H	0	2.792200	-1.806932	-4.471593
113	H	0	2.310903	-3.509807	-4.591435
114	N	0	1.909910	-7.543625	-1.074848
115	C	0	0.579711	-7.880402	-0.628436
116	C	0	0.278275	-7.497064	0.815471
117	O	0	-0.887242	-7.544501	1.211900
118	H	0	2.659662	-8.229867	-1.101532
119	H	0	0.425235	-8.956422	-0.729663
120	H	0	-0.161356	-7.371927	-1.250561
121	N	0	1.275938	-7.138926	1.598100
122	C	0	1.016986	-6.736470	2.967229

123	C	0	1.597176	-5.384506	3.361788
124	O	0	2.731095	-5.081323	3.076587
125	C	0	1.505199	-7.815553	3.980225
126	C	0	0.701745	-9.106828	3.792282
127	C	0	3.009309	-8.089992	3.862751
128	H	0	2.222399	-7.088108	1.244015
129	H	0	-0.071987	-6.686950	3.051151
130	H	0	1.299017	-7.412074	4.981711
131	H	0	0.882324	-9.523353	2.794912
132	H	0	-0.372131	-8.929666	3.899673
133	H	0	1.007252	-9.854562	4.529983
134	H	0	3.601596	-7.182677	4.001478
135	H	0	3.245948	-8.514169	2.880045
136	H	0	3.311813	-8.821400	4.617984
137	N	0	0.784121	-4.600929	4.054428
138	C	0	1.181935	-3.264184	4.493761
139	O	0	3.446373	-2.486574	4.558265
140	C	0	0.101785	-2.665246	5.433029
141	C	0	0.328824	-1.152028	5.540419
142	C	0	-1.326030	-2.948360	4.948425
143	H	0	-0.131630	-4.924708	4.325359
144	H	0	1.260183	-2.620518	3.608072
145	H	0	0.237331	-3.123473	6.421578
146	H	0	0.064435	-0.674017	4.589295
147	H	0	1.367310	-0.906811	5.794433
148	H	0	-0.317195	-0.724555	6.311855
149	H	0	-1.439700	-2.665513	3.897489
150	H	0	-2.029682	-2.328528	5.508085
151	H	0	-1.628392	-3.991016	5.089124
152	C	0	5.125270	-3.904468	0.943319
153	O	0	6.695544	-3.565765	-0.825659

154	C	0	3.914701	-2.971450	0.786433
155	H	0	4.972073	-4.778451	0.293231
156	H	0	3.775998	-2.694854	-0.263989
157	H	0	4.059850	-2.071086	1.391206
158	H	0	3.014734	-3.487236	1.133461
159	C	0	6.147670	-0.235969	-4.560475
160	O	0	5.148728	1.852604	-5.135290
161	C	0	6.841226	-0.402158	-3.175723
162	C	0	6.470324	0.494556	-2.021548
163	C	0	6.983891	1.795812	-1.933514
164	C	0	5.733746	-0.013135	-0.944145
165	C	0	6.772872	2.566363	-0.789767
166	C	0	5.532311	0.750079	0.207377
167	C	0	6.054007	2.041972	0.286726
168	H	0	5.096286	-0.532331	-4.499749
169	H	0	6.676315	-1.445163	-2.883545
170	H	0	7.915770	-0.288415	-3.370393
171	H	0	7.577061	2.192893	-2.753465
172	H	0	5.337503	-1.023813	-1.012515
173	H	0	7.184713	3.568220	-0.731261
174	H	0	4.978655	0.334223	1.043900
175	H	0	5.907160	2.634379	1.183287
176	C	0	2.786460	4.726781	-5.994042
177	O	0	2.001378	6.882685	-6.717371
178	C	0	3.366269	5.015776	-4.587521
179	C	0	3.129658	3.940079	-3.582819
180	N	0	1.951175	3.242345	-3.368378
181	C	0	4.014366	3.461022	-2.641604
182	C	0	2.169997	2.371330	-2.333730
183	N	0	3.409556	2.483296	-1.860757
184	H	0	1.702440	4.594816	-5.890388

185	H	0	2.936363	5.966708	-4.246336
186	H	0	4.443842	5.166711	-4.667597
187	H	0	1.013258	3.344280	-3.779014
188	H	0	5.044915	3.738547	-2.503481
189	H	0	1.446281	1.643267	-2.007889
190	Cu	0	1.350046	2.672740	2.111562
191	C	0	-2.224996	-1.075672	0.894372
192	C	0	-1.926852	1.253608	0.355703
193	C	0	-0.838558	-1.230486	0.971650
194	C	0	-0.552018	1.094101	0.508443
195	O	0	0.258286	2.248031	0.470795
196	H	0	-0.418145	-2.198925	1.219728
197	H	0	-0.100033	2.955250	-0.104749
198	N	0	1.224959	9.280413	0.371891
199	H	0	1.154318	9.986136	1.091048
200	H	0	0.441005	9.203158	-0.262673
201	C	0	3.247757	9.808302	-1.033334
202	O	0	2.630644	10.961747	-1.371858
203	H	0	1.749161	11.048637	-0.951769
204	C	0	2.885440	6.036074	-6.795696
205	O	0	4.108810	6.667328	-6.411381
206	H	0	4.049143	7.627250	-6.597954
207	N	0	3.256221	3.530640	-6.707841
208	H	0	2.858180	2.663798	-6.369192
209	H	0	4.269194	3.435407	-6.671982
210	N	0	6.980755	-1.054091	-5.453754
211	H	0	7.334903	-1.895647	-5.011627
212	H	0	6.560599	-1.262298	-6.354205
213	C	0	6.186038	1.197126	-5.033515
214	O	0	7.401136	1.662825	-5.409066
215	H	0	8.020767	0.897855	-5.499711

216	C	0	2.589874	-3.180074	5.094719
217	O	0	3.030575	-4.537764	5.174213
218	H	0	4.009784	-4.563266	5.127720
219	N	0	4.557606	-7.199941	-1.419495
220	H	0	5.162075	-7.620014	-2.113447
221	H	0	5.031276	-7.012725	-0.545262
222	N	0	7.224026	3.466150	6.623400
223	H	0	7.899363	2.763235	6.903783
224	H	0	7.096015	4.178245	7.331505
225	C	0	5.250286	1.995177	7.022462
226	O	0	6.054241	1.019343	7.533720
227	H	0	5.585111	0.407219	8.137871
228	C	0	-5.162441	4.582581	5.516761
229	O	0	-6.501147	4.406582	5.046651
230	H	0	-7.068591	4.633440	5.810969
231	N	0	-2.776510	5.188060	5.603488
232	H	0	-2.632635	6.112648	5.213754
233	H	0	-2.910017	5.202712	6.608944
234	N	0	5.263279	-4.283071	2.356966
235	H	0	4.358839	-4.429464	2.788149
236	H	0	5.901486	-5.055588	2.511111
237	C	0	6.334057	-3.217676	0.283663
238	O	0	6.880917	-2.198753	0.988520
239	H	0	7.566413	-1.711218	0.484107
240	N	0	-10.991047	1.022205	-1.552256
241	H	0	-11.197579	0.867427	-2.533238
242	H	0	-10.884091	2.015489	-1.368916
243	C	0	-13.395215	0.476362	-1.097660
244	O	0	-13.610636	1.265614	-2.173764
245	H	0	-14.555677	1.303701	-2.431109
246	N	0	-5.667459	1.031306	-6.227565

247	H	0	-5.907925	1.957017	-6.553550
248	H	0	-5.744947	0.334049	-6.957888
249	C	0	-5.134160	-3.187126	-4.010060
250	O	0	-5.966870	-3.178975	-5.065545
251	H	0	-5.872689	-3.961360	-5.649103
252	N	0	-7.685035	-0.177904	-1.005145
253	H	0	-7.703319	0.553143	-0.301314
254	H	0	-8.519942	-0.251523	-1.575972
255	N	0	-1.699160	-3.884250	-5.134966
256	H	0	-1.499401	-2.888280	-5.198393
257	H	0	-2.692584	-4.032503	-4.973249
258	C	0	-1.199622	-6.033813	-4.074502
259	O	0	-2.505528	-6.371489	-3.980609
260	H	0	-2.643107	-7.341809	-3.951536
261	C	0	-2.770414	0.166151	0.533480
262	H	0	-3.846618	0.291075	0.450197
263	C	0	0.010670	-0.140819	0.770637
264	H	0	1.088316	-0.249243	0.821678
265	O	0	-2.327310	2.551636	0.084736
266	H	0	-3.282678	2.647889	-0.082587
267	C	0	-3.134569	-2.216017	1.283462
268	H	0	-2.525769	-3.097079	1.498341
269	H	0	-3.781460	-2.478383	0.437406
270	C	0	-4.038758	-1.921145	2.512740
271	H	0	-3.952340	-2.740252	3.231711
272	H	0	-5.958114	-2.752886	2.135148
273	H	0	-5.910091	-1.171252	2.772495
274	H	0	-5.582908	-1.435202	1.149509
275	N	0	-5.489055	-1.845849	2.108078
276	C	0	-3.739692	-0.607230	3.297684
277	O	0	-4.753810	0.166903	3.466979

278	O	0	-2.566663	-0.453200	3.725120

DOPA decarboxylase: Overall Charge = 0, Total energy = -7847.05377559 Ha

1	N	0	-8.292490	7.425593	-0.531561
2	C	0	-7.399598	6.626431	-1.355573
3	C	0	-7.891608	6.476864	-2.785107
4	O	0	-7.115036	6.199725	-3.696858
5	C	0	-7.340262	5.177491	-0.777541
6	C	0	-6.970255	5.143857	0.670197
7	C	0	-7.842761	5.213271	1.732197
8	C	0	-5.638883	5.078430	1.233018
9	N	0	-7.135826	5.204846	2.922384
10	C	0	-5.783171	5.126499	2.647590
11	C	0	-4.350522	4.948940	0.682534
12	C	0	-4.685256	5.058812	3.512683
13	C	0	-3.257123	4.880596	1.538320
14	C	0	-3.426329	4.937032	2.939771
15	H	0	-8.461987	8.363928	-0.874248
16	H	0	-8.037043	7.413065	0.448983
17	H	0	-6.373784	7.014974	-1.433784
18	H	0	-6.627676	4.599711	-1.375821
19	H	0	-8.333070	4.735765	-0.917502
20	H	0	-8.920139	5.263855	1.725512
21	H	0	-7.545595	5.229052	3.841699
22	H	0	-4.207415	4.890158	-0.391762
23	H	0	-4.813614	5.063327	4.588391
24	H	0	-2.265422	4.762316	1.116104
25	H	0	-2.561116	4.859530	3.587669
26	N	0	5.401435	4.844816	-3.032705
27	C	0	4.079246	4.341596	-2.739680
28	C	0	3.350852	3.695040	-3.932311
29	O	0	3.809616	3.736956	-5.095381

30	C	0	3.221172	5.487886	-2.156285
31	C	0	3.422383	5.748252	-0.674613
32	C	0	2.437319	6.460813	0.027910
33	C	0	4.529518	5.271186	0.043577
34	C	0	2.544832	6.691130	1.397933
35	C	0	4.640965	5.482807	1.418215
36	C	0	3.647809	6.190035	2.093155
37	O	0	3.798631	6.346382	3.463594
38	H	0	5.389551	5.672576	-3.621709
39	H	0	6.056294	4.163009	-3.405193
40	H	0	4.165166	3.548314	-1.981621
41	H	0	3.448901	6.395921	-2.731622
42	H	0	2.158443	5.282300	-2.321540
43	H	0	1.566051	6.829133	-0.504692
44	H	0	5.315677	4.743703	-0.484031
45	H	0	1.771388	7.244494	1.920683
46	H	0	5.489922	5.106752	1.975886
47	H	0	3.103397	6.902590	3.861222
48	N	0	2.187830	3.075185	-3.605884
49	C	0	1.382092	2.338257	-4.563869
50	C	0	-0.080923	2.552414	-4.212088
51	O	0	-0.392873	2.767025	-3.008166
52	C	0	1.678843	0.811306	-4.468910
53	C	0	3.117146	0.514248	-4.812832
54	C	0	4.079521	0.335288	-3.811707
55	C	0	3.518957	0.471989	-6.155731
56	C	0	5.421590	0.136471	-4.152023
57	C	0	4.855292	0.269576	-6.496255
58	C	0	5.813989	0.107349	-5.491392
59	H	0	1.831348	3.106031	-2.645227
60	H	0	1.617886	2.701752	-5.568436

61	H	0	1.451062	0.499825	-3.442419
62	H	0	0.998403	0.266948	-5.134066
63	H	0	3.788441	0.338692	-2.765372
64	H	0	2.776891	0.603698	-6.938393
65	H	0	6.148907	-0.013271	-3.362077
66	H	0	5.149450	0.237735	-7.539106
67	H	0	6.853943	-0.054365	-5.754043
68	N	0	-1.022528	2.432320	-5.156887
69	C	0	-2.433862	2.453701	-4.775103
70	C	0	-2.787387	1.154341	-4.059927
71	O	0	-2.083259	0.127794	-4.155775
72	C	0	-3.174492	2.503987	-6.135725
73	C	0	-2.235518	1.699839	-7.051084
74	C	0	-0.829448	2.109247	-6.584965
75	H	0	-2.649691	3.311677	-4.133746
76	H	0	-3.249821	3.543543	-6.465216
77	H	0	-4.180369	2.083690	-6.070578
78	H	0	-2.386918	0.628444	-6.892085
79	H	0	-2.390281	1.918414	-8.108078
80	H	0	-0.469526	2.993400	-7.120392
81	H	0	-0.105295	1.301958	-6.711430
82	N	0	-3.984588	1.179812	-3.412618
83	C	0	-4.693784	-0.048261	-3.123330
84	C	0	-5.876490	-0.135397	-4.039672
85	O	0	-6.444730	0.755174	-4.669952
86	C	0	-5.106767	-0.201549	-1.634543
87	O	0	-5.716932	-1.488419	-1.415931
88	C	0	-6.090708	0.866544	-1.186149
89	H	0	-4.515568	2.042446	-3.394528
90	H	0	-4.038603	-0.887348	-3.367320
91	H	0	-4.174159	-0.119003	-1.059531

92	H	0	-5.374448	-2.179272	-2.032898
93	H	0	-5.625708	1.857256	-1.191929
94	H	0	-6.422052	0.651658	-0.167504
95	H	0	-6.974132	0.879289	-1.832796
96	N	0	11.478793	-2.402487	1.048761
97	C	0	10.391531	-1.997878	1.925483
98	C	0	10.683133	-2.554224	3.290220
99	O	0	11.311144	-3.575911	3.564306
100	C	0	8.993270	-2.454945	1.414608
101	C	0	7.816912	-1.928369	2.162018
102	N	0	7.177572	-2.600084	3.191829
103	C	0	7.113496	-0.753790	2.009920
104	C	0	6.126830	-1.832013	3.614249
105	N	0	6.056687	-0.701715	2.914234
106	H	0	11.443990	-1.979859	0.128969
107	H	0	11.626317	-3.405453	1.012634
108	H	0	10.380565	-0.908627	2.013654
109	H	0	8.972763	-3.550047	1.363945
110	H	0	8.930344	-2.087581	0.385158
111	H	0	7.430798	-3.506190	3.556968
112	H	0	7.285969	0.024025	1.284231
113	H	0	5.471636	-2.124075	4.416258
114	N	0	9.460112	0.807810	-3.529046
115	C	0	8.572160	1.874193	-3.103557
116	C	0	9.068001	3.266977	-3.430863
117	O	0	8.299550	4.122595	-3.871667
118	C	0	8.263157	1.693285	-1.591380
119	O	0	8.120470	0.257302	-1.424310
120	C	0	6.984033	2.389544	-1.173811
121	H	0	9.316252	0.480863	-4.476695
122	H	0	10.443854	0.970724	-3.331821

123	H	0	7.629291	1.778937	-3.649712
124	H	0	9.118333	2.029731	-0.994929
125	H	0	8.735016	-0.133471	-2.112944
126	H	0	7.039058	3.459651	-1.400190
127	H	0	6.804427	2.269351	-0.103112
128	H	0	6.134649	1.963593	-1.721164
129	N	0	-4.015421	-6.714978	-4.300353
130	C	0	-4.707266	-5.649653	-3.590465
131	C	0	-4.062104	-4.284683	-3.721825
132	O	0	-4.483083	-3.309350	-3.099755
133	C	0	-4.873186	-5.890570	-2.071123
134	C	0	-3.574644	-5.761367	-1.337197
135	N	0	-2.867292	-6.811978	-0.763947
136	C	0	-2.783802	-4.651982	-1.130009
137	C	0	-1.700280	-6.321546	-0.243868
138	N	0	-1.622415	-5.012954	-0.467358
139	H	0	-4.609866	-7.337058	-4.829136
140	H	0	-3.311948	-7.207911	-3.761392
141	H	0	-5.710221	-5.549412	-4.021135
142	H	0	-5.336169	-6.867334	-1.904830
143	H	0	-5.561562	-5.120033	-1.720023
144	H	0	-3.172428	-7.773171	-0.722657
145	H	0	-2.978768	-3.631485	-1.407002
146	H	0	-0.959893	-6.892919	0.289043
147	N	0	-3.033273	-4.208570	-4.548737
148	C	0	-2.451210	-2.916602	-4.831740
149	C	0	-2.922082	-2.513679	-6.219402
150	O	0	-3.790772	-1.661480	-6.372681
151	C	0	-0.923315	-2.916231	-4.680655
152	C	0	-0.525095	-3.061322	-3.206376
153	C	0	0.991966	-3.159286	-2.994321

154	C	0	1.423508	-2.882328	-1.554142
155	N	0	0.968642	-3.880973	-0.542428
156	H	0	-2.754851	-5.058106	-5.032908
157	H	0	-2.879229	-2.180386	-4.159070
158	H	0	-0.540001	-1.967318	-5.072750
159	H	0	-0.492642	-3.724587	-5.284772
160	H	0	-0.914881	-2.189757	-2.659722
161	H	0	-1.012255	-3.948087	-2.786789
162	H	0	1.496653	-2.416218	-3.626479
163	H	0	1.354952	-4.143349	-3.316277
164	H	0	2.513091	-2.829458	-1.485187
165	H	0	1.036123	-1.911157	-1.229165
166	H	0	-0.030749	-4.208601	-0.605459
167	H	0	1.041327	-3.406959	0.432895
168	H	0	1.564043	-4.708985	-0.484612
169	N	0	-8.218894	-4.905916	-0.861769
170	C	0	-7.332539	-5.334342	0.181991
171	C	0	-7.870555	-6.409672	1.040753
172	O	0	-9.022124	-6.399135	1.490723
173	C	0	-7.020775	-4.127274	1.110148
174	C	0	-5.982839	-4.466610	2.151090
175	C	0	-6.347595	-4.882490	3.436387
176	C	0	-4.621878	-4.390572	1.823224
177	C	0	-5.368197	-5.218242	4.375831
178	C	0	-3.640327	-4.724971	2.755963
179	C	0	-4.015253	-5.141009	4.038044
180	H	0	-8.087668	-3.949235	-1.161785
181	H	0	-8.366721	-5.551831	-1.626024
182	H	0	-6.360276	-5.698845	-0.168668
183	H	0	-7.960911	-3.803549	1.569349
184	H	0	-6.656614	-3.318881	0.464473

185	H	0	-7.399414	-4.939749	3.702408
186	H	0	-4.334483	-4.046985	0.833987
187	H	0	-5.662099	-5.535105	5.370107
188	H	0	-2.593723	-4.642936	2.484950
189	H	0	-3.257734	-5.397744	4.769755
190	N	0	-6.043775	1.896973	3.573875
191	C	0	-5.105109	0.953202	4.187899
192	C	0	-4.523895	1.761075	5.358228
193	O	0	-3.669937	2.625669	5.152468
194	C	0	-3.944973	0.458742	3.303281
195	C	0	-4.424894	0.005873	1.918915
196	C	0	-3.195549	-0.651490	4.059058
197	C	0	-5.543975	-1.037346	1.950899
198	H	0	-5.586854	2.679643	3.112587
199	H	0	-6.756693	1.469910	2.994158
200	H	0	-5.673138	0.107310	4.583598
201	H	0	-3.269849	1.314873	3.158879
202	H	0	-4.757357	0.887057	1.353954
203	H	0	-3.556375	-0.403668	1.384120
204	H	0	-3.893764	-1.420398	4.412044
205	H	0	-2.679980	-0.248433	4.942341
206	H	0	-2.455129	-1.144286	3.419835
207	H	0	-6.477143	-0.620387	2.347747
208	H	0	-5.269271	-1.891814	2.575623
209	H	0	-5.744535	-1.405524	0.940350
210	N	0	-2.169724	1.883669	7.490101
211	C	0	-0.835759	1.542475	7.965702
212	C	0	-0.199798	2.680541	8.758236
213	O	0	0.847559	2.512171	9.361637
214	C	0	0.172730	1.095911	6.871852
215	C	0	0.514120	2.190888	5.891275

216	C	0	1.688398	2.943873	6.052391
217	C	0	-0.325068	2.473048	4.803847
218	C	0	2.026699	3.942458	5.137106
219	C	0	0.012397	3.472041	3.887035
220	C	0	1.188936	4.204541	4.049596
221	H	0	-2.187983	2.633014	6.802188
222	H	0	-2.643387	1.059033	7.130957
223	H	0	-0.931546	0.721215	8.683238
224	H	0	1.080037	0.737854	7.367480
225	H	0	-0.281544	0.242664	6.354595
226	H	0	2.344919	2.728208	6.890117
227	H	0	-1.231707	1.891737	4.652637
228	H	0	2.946631	4.504528	5.253531
229	H	0	-0.623055	3.643072	3.023342
230	H	0	1.462055	4.959340	3.320966
231	N	0	6.971146	-4.248102	-0.559018
232	C	0	6.847764	-3.024849	-1.102766
233	C	0	7.775399	-2.687685	-2.235557
234	C	0	5.875733	-2.113542	-0.633706
235	O	0	5.827947	-0.836359	-1.109385
236	C	0	4.953412	-2.531345	0.342699
237	C	0	5.131906	-3.800713	0.926566
238	C	0	6.155867	-4.613100	0.446119
239	C	0	4.245385	-4.332382	2.013171
240	O	0	2.994655	-4.754038	1.413219
241	15	0	1.865415	-5.526336	2.494701
242	O	0	2.567936	-6.850224	3.062770
243	O	0	1.740243	-4.451111	3.771655
244	O	0	0.541877	-5.695551	1.606939
245	H	0	8.721991	-2.266580	-1.874929
246	H	0	7.322621	-1.978212	-2.933465

247	H	0	8.019285	-3.604320	-2.774434
248	H	0	6.740151	-0.419514	-1.263946
249	H	0	6.317048	-5.597652	0.870584
250	H	0	4.730665	-5.190066	2.492120
251	H	0	4.045952	-3.583750	2.786261
252	C	0	0.632487	0.973963	1.887021
253	C	0	-0.765014	1.006324	1.822004
254	C	0	1.357981	1.796780	1.016808
255	C	0	0.707025	2.653633	0.137733
256	C	0	-0.692596	2.693020	0.084196
257	O	0	-1.361228	3.570180	-0.748264
258	H	0	-1.351285	0.357062	2.459407
259	H	0	2.443286	1.763257	1.020522
260	O	0	-9.227628	6.606884	-2.963824
261	H	0	-9.487134	6.458165	-3.897563
262	O	0	-6.362720	-1.477648	-4.118448
263	H	0	-7.151725	-1.506632	-4.700621
264	O	0	-7.048654	-7.527330	1.386203
265	H	0	-7.552463	-8.099600	2.001487
266	O	0	-2.350651	-3.131299	-7.375342
267	H	0	-2.787887	-2.772685	-8.176106
268	O	0	10.442911	3.602437	-3.229042
269	H	0	10.590369	4.534212	-3.495803
270	O	0	10.207113	-1.716976	4.346948
271	H	0	10.393251	-2.135765	5.213493
272	O	0	-0.845091	3.955786	8.787582
273	H	0	-0.298381	4.574099	9.316990
274	O	0	-5.003434	1.474816	6.589694
275	H	0	-4.580728	2.019926	7.294146
276	C	0	3.835465	-1.677981	0.803935
277	H	0	3.371008	-1.968931	1.749606

278	O	0	3.348554	-0.728457	0.164380
279	C	0	-1.419004	1.857479	0.929400
280	H	0	-2.501971	1.895178	0.874523
281	H	0	-0.965772	3.551997	-1.655419
282	C	0	1.387207	0.142985	2.900156
283	H	0	1.656624	0.775990	3.758533
284	H	0	2.319585	-0.199792	2.435428
285	C	0	0.646638	-1.094344	3.430336
286	H	0	-0.227942	-0.801650	4.019238
287	O	0	1.421170	3.480105	-0.739869
288	H	0	2.196462	3.892790	-0.306360
289	H	0	1.650931	-3.403880	3.774102
290	H	0	2.520188	-1.564189	4.243241
291	H	0	1.261490	-1.964753	5.246459
292	N	0	1.561957	-1.908653	4.276081
293	C	0	0.194464	-1.970132	2.260589
294	O	0	1.179474	-2.491214	1.612607
295	O	0	-1.034917	-2.127832	2.008894

Monoamine Oxidase: Overall Charge = -1, Total energy = -12802.9081742 Ha

1	N	0	5.065089	1.304345	7.509049
2	C	0	4.007696	0.512749	8.084076
3	C	0	4.338090	-0.975336	8.107337
4	O	0	4.807599	-1.593810	7.143413
5	C	0	2.716382	0.666751	7.236856
6	C	0	2.274554	2.105671	7.077795
7	C	0	1.897013	2.858111	8.193463
8	C	0	2.320854	2.735842	5.842435
9	C	0	1.553872	4.192176	8.074728
10	C	0	1.972074	4.072501	5.715652

11	C	0	1.592924	4.788687	6.834991
12	O	0	1.268052	6.111559	6.720779
13	H	0	5.025820	1.446465	6.506081
14	H	0	5.985428	1.226707	7.915663
15	H	0	3.805173	0.815887	9.116421
16	H	0	1.942005	0.062273	7.719238
17	H	0	2.906392	0.232362	6.247824
18	H	0	1.864292	2.384100	9.170622
19	H	0	2.648434	2.182739	4.969788
20	H	0	1.259830	4.763097	8.949570
21	H	0	1.990369	4.566641	4.751513
22	H	0	1.015615	6.515122	7.569717
23	C	0	-12.313789	-1.572040	5.457762
24	C	0	-12.878188	-1.223165	4.075949
25	O	0	-12.137150	-0.729029	3.226226
26	C	0	-11.635600	-2.946341	5.413612
27	C	0	-12.676762	-3.893983	5.901778
28	C	0	-13.398313	-3.133631	6.977521
29	H	0	-11.610790	-0.774043	5.710498
30	H	0	-11.277445	-3.188845	4.416394
31	H	0	-10.767742	-2.939009	6.082896
32	H	0	-12.260033	-4.836191	6.265685
33	H	0	-13.372927	-4.139768	5.088192
34	H	0	-12.866113	-3.213083	7.935978
35	H	0	-14.422984	-3.480425	7.132260
36	N	0	-14.173889	-1.468459	3.870044
37	C	0	-14.803555	-1.352872	2.547287
38	C	0	-15.622566	-0.084307	2.385129
39	O	0	-16.547676	0.153997	3.166731
40	C	0	-15.722031	-2.555673	2.273181
41	C	0	-15.069828	-3.899341	2.491669

42	C	0	-13.897898	-4.246355	1.817095
43	C	0	-15.642065	-4.822421	3.368587
44	C	0	-13.296176	-5.495586	2.023178
45	C	0	-15.057216	-6.076054	3.573596
46	C	0	-13.879680	-6.412865	2.900208
47	H	0	-14.711060	-1.760378	4.681487
48	H	0	-13.979263	-1.371569	1.834653
49	H	0	-16.080871	-2.466760	1.238902
50	H	0	-16.602990	-2.465006	2.919254
51	H	0	-13.442449	-3.542624	1.125945
52	H	0	-16.553227	-4.559623	3.897223
53	H	0	-12.383507	-5.748272	1.495408
54	H	0	-15.516014	-6.781677	4.256684
55	H	0	-13.420055	-7.381647	3.059185
56	N	0	-15.293619	0.738618	1.366767
57	C	0	-16.118028	1.913081	1.069223
58	C	0	-17.505791	1.452529	0.632722
59	O	0	-17.606794	0.585541	-0.225621
60	C	0	-15.383020	2.576264	-0.108356
61	C	0	-14.000802	2.007311	-0.078309
62	C	0	-14.168178	0.609873	0.427323
63	H	0	-16.196209	2.558283	1.947393
64	H	0	-15.405943	3.663644	-0.029436
65	H	0	-15.882949	2.299136	-1.042280
66	H	0	-13.513449	2.043045	-1.054829
67	H	0	-13.373154	2.572935	0.619004
68	H	0	-13.279256	0.247650	0.946705
69	H	0	-14.425123	-0.090301	-0.377501
70	N	0	-13.510225	-7.600993	-6.107285
71	C	0	-12.576701	-8.004443	-5.036435
72	C	0	-12.562697	-9.506609	-4.838880

73	O	0	-11.495814	-10.106319	-4.646601
74	C	0	-12.878757	-7.291107	-3.705064
75	C	0	-13.046807	-5.833052	-3.911418
76	C	0	-14.177577	-5.098266	-3.705906
77	C	0	-12.072325	-4.930609	-4.452646
78	N	0	-13.960866	-3.786453	-4.060170
79	C	0	-12.679513	-3.657595	-4.528147
80	C	0	-10.736768	-5.071976	-4.861252
81	C	0	-12.000830	-2.526093	-5.005418
82	C	0	-10.062781	-3.953467	-5.338351
83	C	0	-10.699312	-2.694833	-5.409410
84	H	0	-14.482862	-7.760637	-5.858033
85	H	0	-13.360714	-6.637686	-6.388820
86	H	0	-11.566123	-7.760232	-5.366385
87	H	0	-12.072690	-7.508016	-2.991541
88	H	0	-13.800414	-7.712282	-3.284043
89	H	0	-15.133678	-5.417950	-3.322300
90	H	0	-14.639938	-3.045985	-3.992890
91	H	0	-10.227487	-6.027862	-4.795283
92	H	0	-12.494392	-1.561896	-5.059070
93	H	0	-9.035028	-4.047686	-5.659361
94	H	0	-10.150881	-1.845376	-5.803392
95	N	0	-12.439454	2.716753	-8.605018
96	C	0	-11.864417	2.187085	-7.358169
97	C	0	-10.824493	1.077295	-7.589457
98	O	0	-9.795002	1.035076	-6.896194
99	C	0	-12.944408	1.744659	-6.358438
100	C	0	-12.452878	1.457079	-4.923644
101	C	0	-11.716639	2.657980	-4.276080
102	C	0	-13.603613	1.000714	-4.022488
103	H	0	-11.763442	3.139500	-9.229800

104	H	0	-13.066785	2.082492	-9.088601
105	H	0	-11.298994	3.006122	-6.899407
106	H	0	-11.019906	0.334759	-8.376752
107	H	0	-13.458911	0.853175	-6.745660
108	H	0	-13.686690	2.553642	-6.331890
109	H	0	-11.725716	0.631571	-4.979729
110	H	0	-11.484586	2.429852	-3.231053
111	H	0	-12.350431	3.552770	-4.299354
112	H	0	-10.776822	2.880740	-4.788417
113	H	0	-14.171255	0.180364	-4.476089
114	H	0	-14.300014	1.828208	-3.838792
115	H	0	-13.220119	0.655182	-3.057400
116	N	0	-6.919784	1.171687	-6.474828
117	C	0	-6.367743	-0.010938	-5.788884
118	C	0	-5.052584	-0.474241	-6.414564
119	O	0	-4.056664	-0.621552	-5.708229
120	C	0	-7.416538	-1.139490	-5.772382
121	C	0	-6.904566	-2.473310	-5.250033
122	C	0	-6.668281	-2.667806	-3.889900
123	C	0	-6.722674	-3.553550	-6.125231
124	C	0	-6.209247	-3.917135	-3.404264
125	C	0	-6.268137	-4.813701	-5.641509
126	C	0	-6.008878	-4.981913	-4.287185
127	H	0	-7.919196	1.138068	-6.636045
128	H	0	-6.585987	2.058155	-6.121716
129	H	0	-6.079526	0.207688	-4.753007
130	H	0	-7.795914	-1.262348	-6.793618
131	H	0	-8.248015	-0.781822	-5.155229
132	H	0	-6.828507	-1.851027	-3.190973
133	H	0	-6.924733	-3.426709	-7.184475
134	H	0	-6.009363	-4.046507	-2.348520

135	H	0	-6.130342	-5.632584	-6.337970
136	H	0	-5.657990	-5.937142	-3.912460
137	N	0	-2.300585	2.258656	-5.964612
138	C	0	-1.866742	2.418361	-4.573398
139	C	0	-1.039557	1.220600	-4.085649
140	O	0	-0.119449	1.393725	-3.298972
141	C	0	-3.089471	2.615059	-3.677467
142	C	0	-2.938759	2.890894	-2.181717
143	C	0	-1.960048	4.034512	-1.901665
144	C	0	-4.323262	3.229480	-1.645820
145	H	0	-1.603354	2.486308	-6.661421
146	H	0	-3.209987	2.650176	-6.173689
147	H	0	-1.190719	3.276008	-4.449506
148	H	0	-3.737133	1.736294	-3.814416
149	H	0	-3.638675	3.469380	-4.104246
150	H	0	-2.576645	1.974331	-1.688931
151	H	0	-1.940454	4.268386	-0.829210
152	H	0	-2.283098	4.943380	-2.425341
153	H	0	-0.946076	3.797814	-2.246627
154	H	0	-5.047846	2.450543	-1.906090
155	H	0	-4.672898	4.174445	-2.076236
156	H	0	-4.315840	3.338056	-0.556657
157	N	0	-1.370102	0.020504	-4.552051
158	C	0	-0.628959	-1.191341	-4.152592
159	C	0	0.772960	-1.231580	-4.727148
160	O	0	1.720999	-1.666569	-4.050756
161	C	0	-1.361703	-2.466459	-4.598342
162	S	0	-2.821640	-2.919246	-3.611113
163	H	0	-2.143474	-0.053037	-5.214613
164	H	0	-0.485787	-1.184810	-3.068993
165	H	0	-1.720136	-2.339390	-5.622267

166	H	0	-0.661390	-3.303819	-4.590414
167	H	0	-2.149397	-3.294799	-2.474313
168	N	0	-4.282894	-6.704277	2.527732
169	C	0	-3.419383	-5.685657	1.913572
170	C	0	-3.959450	-4.251619	2.082292
171	O	0	-3.176609	-3.314241	2.245221
172	C	0	-3.055750	-6.028778	0.424001
173	C	0	-1.697991	-5.426429	0.038915
174	C	0	-4.182486	-5.648767	-0.568413
175	C	0	-1.224022	-5.786250	-1.402825
176	H	0	-4.182467	-7.630064	2.131740
177	H	0	-4.273976	-6.710037	3.539991
178	H	0	-2.453039	-5.633899	2.440065
179	H	0	-2.946057	-7.124381	0.404538
180	H	0	-1.752046	-4.332532	0.138432
181	H	0	-0.936890	-5.761644	0.751639
182	H	0	-3.993359	-6.079276	-1.555286
183	H	0	-4.244412	-4.557635	-0.674543
184	H	0	-5.151086	-6.017595	-0.214938
185	H	0	-1.122997	-6.871469	-1.512635
186	H	0	-0.250591	-5.326086	-1.591968
187	H	0	-1.927374	-5.437715	-2.162838
188	N	0	-5.281056	-4.087803	2.068387
189	C	0	-5.877005	-2.736744	2.105352
190	C	0	-6.160618	-2.244965	3.522438
191	O	0	-6.457431	-1.069480	3.723034
192	C	0	-7.182969	-2.627102	1.246209
193	C	0	-8.334750	-3.461212	1.833943
194	C	0	-6.918809	-3.082380	-0.187659
195	C	0	-9.180087	-2.754915	2.858178
196	H	0	-5.856904	-4.922541	2.051752

197	H	0	-5.127277	-2.048380	1.703835
198	H	0	-7.459128	-1.566589	1.257477
199	H	0	-7.947073	-4.404294	2.244199
200	H	0	-8.981403	-3.754520	0.998063
201	H	0	-6.035414	-2.591136	-0.612652
202	H	0	-7.780843	-2.849553	-0.819589
203	H	0	-6.756878	-4.164714	-0.219753
204	H	0	-10.097855	-3.324024	3.011562
205	H	0	-9.473558	-1.755453	2.523430
206	H	0	-8.692880	-2.656102	3.832971
207	N	0	-1.215558	-1.832660	7.103558
208	C	0	-0.975977	-0.466971	7.579662
209	C	0	-1.316051	-0.261804	9.060232
210	O	0	-1.480990	0.876619	9.494320
211	C	0	-1.755095	0.545734	6.718901
212	C	0	-1.200910	0.719395	5.301043
213	C	0	-1.322083	-0.551986	4.443932
214	O	0	-0.311001	-1.184534	4.096241
215	N	0	-2.555526	-0.953406	4.133177
216	H	0	-0.720375	-2.040028	6.242036
217	H	0	-1.042233	-2.541370	7.809931
218	H	0	0.087642	-0.180961	7.522106
219	H	0	-1.720493	1.517262	7.219330
220	H	0	-2.802445	0.223369	6.692492
221	H	0	-1.708946	1.537984	4.787783
222	H	0	-0.135189	0.970172	5.340532
223	H	0	-3.368096	-0.425563	4.413830
224	H	0	-2.702235	-1.781928	3.557882
225	N	0	-9.670537	8.309133	0.780419
226	C	0	-9.547275	7.560385	-0.469137
227	C	0	-10.201932	8.451288	-1.523684

228	O	0	-11.407996	8.690926	-1.475909
229	C	0	-10.262806	6.187705	-0.370441
230	C	0	-9.631042	5.322237	0.730889
231	C	0	-10.244071	5.443649	-1.714478
232	C	0	-10.621673	4.399744	1.420171
233	H	0	-10.622570	8.582289	0.997114
234	H	0	-9.002823	9.059201	0.907443
235	H	0	-8.489009	7.418017	-0.704917
236	H	0	-11.305285	6.408969	-0.096738
237	H	0	-9.175590	5.983912	1.476776
238	H	0	-8.819246	4.728670	0.285938
239	H	0	-9.211635	5.300049	-2.052989
240	H	0	-10.791655	5.986116	-2.492721
241	H	0	-10.706038	4.457681	-1.607406
242	H	0	-11.115596	3.736087	0.701448
243	H	0	-11.399556	4.978525	1.928990
244	H	0	-10.126458	3.770321	2.165458
245	N	0	-4.603079	8.860667	-1.811227
246	C	0	-4.730310	8.185888	-0.522411
247	C	0	-3.923138	8.854882	0.574680
248	O	0	-2.765390	9.208718	0.365644
249	C	0	-4.268541	6.734928	-0.667257
250	C	0	-4.553286	5.825511	0.517426
251	C	0	-5.855031	5.420729	0.817345
252	C	0	-3.511752	5.316186	1.291591
253	C	0	-6.120719	4.546480	1.876085
254	C	0	-3.767641	4.428914	2.358726
255	C	0	-5.069652	4.055598	2.639486
256	O	0	-5.329249	3.188831	3.686928
257	H	0	-3.638422	8.937164	-2.123038
258	H	0	-5.078505	9.755513	-1.856981

259	H	0	-5.784489	8.197690	-0.229464
260	H	0	-3.192000	6.738102	-0.882236
261	H	0	-4.773512	6.355786	-1.561765
262	H	0	-6.683707	5.783329	0.215236
263	H	0	-2.485142	5.604811	1.083846
264	H	0	-7.135468	4.238242	2.102272
265	H	0	-2.937356	4.014245	2.921463
266	H	0	-4.507580	2.865165	4.100021
267	N	0	0.192139	10.063838	-0.675181
268	C	0	1.029643	9.530749	-1.764787
269	O	0	-0.594105	10.249373	-3.364435
270	C	0	0.831925	8.012125	-1.947817
271	C	0	1.440755	7.146935	-0.861220
272	C	0	2.812426	7.173600	-0.605825
273	C	0	0.644205	6.271945	-0.130421
274	C	0	3.371656	6.371808	0.401176
275	C	0	1.191840	5.452753	0.861451
276	C	0	2.554699	5.503108	1.126181
277	H	0	-0.800469	9.874334	-0.750633
278	H	0	0.550416	9.834082	0.244282
279	H	0	2.074628	9.749164	-1.532458
280	H	0	-0.245841	7.824003	-2.014257
281	H	0	1.263515	7.707688	-2.912862
282	H	0	3.455686	7.834200	-1.179933
283	H	0	-0.425413	6.251839	-0.320739
284	H	0	4.434632	6.429279	0.604953
285	H	0	0.566955	4.790395	1.440533
286	H	0	2.975451	4.864232	1.895355
287	N	0	7.235037	1.672892	-4.424443
288	C	0	7.772342	0.895758	-3.318973
289	C	0	8.702429	-0.216561	-3.758349

290	O	0	9.648293	-0.557772	-3.039532
291	C	0	6.660154	0.226849	-2.499926
292	C	0	5.370405	1.007139	-2.386336
293	C	0	5.361878	2.325332	-1.936742
294	C	0	4.145515	0.408175	-2.708785
295	C	0	4.161349	3.039254	-1.825210
296	C	0	2.952784	1.110534	-2.603389
297	C	0	2.967147	2.424554	-2.159356
298	O	0	1.773374	3.116356	-2.062069
299	H	0	7.928093	2.091272	-5.037234
300	H	0	6.494634	1.211401	-4.940786
301	H	0	8.378383	1.547664	-2.682809
302	H	0	7.057538	-0.000188	-1.502266
303	H	0	6.421715	-0.741911	-2.957868
304	H	0	6.299154	2.819406	-1.699871
305	H	0	4.120861	-0.617336	-3.061491
306	H	0	4.172007	4.067479	-1.472953
307	H	0	1.999979	0.662310	-2.862285
308	H	0	1.919425	4.068349	-1.892784
309	N	0	7.753065	-5.360935	0.666956
310	C	0	7.617736	-4.761056	1.982595
311	C	0	8.887854	-5.019807	2.796520
312	O	0	9.731112	-5.838937	2.414008
313	C	0	6.419509	-5.325071	2.763931
314	C	0	5.068649	-4.990926	2.168671
315	C	0	4.648431	-5.553949	0.948189
316	C	0	4.206593	-4.120541	2.822381
317	C	0	3.394493	-5.227752	0.400455
318	C	0	2.957989	-3.804082	2.283956
319	C	0	2.561431	-4.359720	1.082071
320	O	0	1.321594	-4.026147	0.572506

321	H	0	8.091006	-6.323319	0.718964
322	H	0	8.328819	-4.810302	0.036089
323	H	0	7.499192	-3.673384	1.898168
324	H	0	6.472935	-4.924316	3.780964
325	H	0	6.544390	-6.415411	2.821598
326	H	0	5.310141	-6.227017	0.419266
327	H	0	4.515777	-3.673233	3.763056
328	H	0	3.070272	-5.650924	-0.543074
329	H	0	2.297088	-3.118216	2.806867
330	H	0	0.925886	-3.254457	1.018858
331	O	0	20.749459	0.949583	0.556975
332	C	0	21.071629	0.905376	-1.853880
333	O	0	21.621578	2.188586	-1.608628
334	C	0	22.117173	-0.191954	-2.007479
335	N	0	22.751570	-0.312716	-3.339531
336	C	0	22.170378	-0.154896	-4.581155
337	N	0	23.109367	-0.374765	-5.529417
338	C	0	24.289765	-0.657722	-4.917293
339	C	0	25.569831	-0.952401	-5.399581
340	N	0	25.822904	-1.036116	-6.715880
341	N	0	26.583805	-1.219965	-4.489565
342	C	0	26.350006	-1.176639	-3.131869
343	N	0	25.086115	-0.880522	-2.666890
344	C	0	24.072715	-0.625973	-3.541185
345	N	0	7.361705	-1.247586	2.963812
346	C	0	6.674446	-1.581294	4.110481
347	O	0	6.899012	-2.648825	4.679733
348	N	0	5.781379	-0.678680	4.654309
349	C	0	5.533123	0.532351	4.029754
350	O	0	4.739289	1.324885	4.545162
351	C	0	6.213460	0.869939	2.864703

352	N	0	5.993725	2.086321	2.224876
353	C	0	6.813269	2.501956	1.197032
354	C	0	6.767401	3.833643	0.770594
355	C	0	7.813046	4.379066	0.022049
356	C	0	7.691340	5.815569	-0.402348
357	C	0	8.927181	3.592424	-0.315104
358	C	0	10.092869	4.122095	-1.119550
359	C	0	8.966631	2.255084	0.114129
360	C	0	7.910232	1.701244	0.864879
361	N	0	7.884519	0.353094	1.240516
362	C	0	7.138083	-0.027245	2.338829
363	C	0	8.859636	-0.617518	0.618278
364	C	0	10.030283	-0.962111	1.557268
365	O	0	10.677247	0.234496	1.942868
366	C	0	10.985913	-1.915574	0.833894
367	O	0	10.295760	-3.115035	0.523142
368	C	0	12.259942	-2.293216	1.607425
369	O	0	12.842034	-1.148504	2.210315
370	C	0	13.266408	-2.897245	0.633551
371	O	0	14.382290	-3.392205	1.358097
372	15	0	15.604251	-4.145207	0.621675
373	O	0	16.720426	-4.290781	1.755078
374	O	0	15.161183	-5.297346	-0.159568
375	O	0	16.069765	-3.096334	-0.466596
376	15	0	16.580626	-1.541930	-0.456292
377	O	0	16.414766	-1.022911	-1.958434
378	O	0	15.984455	-0.765511	0.696795
379	O	0	18.207975	-1.654355	-0.143239
380	C	0	19.144054	-1.818546	-1.170788
381	C	0	20.417520	-1.110536	-0.741881
382	O	0	21.414374	-1.381697	-1.700932

383	C	0	20.253267	0.407733	-0.679425
384	H	0	20.124900	0.682749	1.270671
385	H	0	20.442561	0.964234	-2.744362
386	H	0	21.710629	2.311520	-0.638701
387	H	0	22.942007	-0.026930	-1.303958
388	H	0	21.136238	0.083334	-4.752136
389	H	0	26.750735	-1.265283	-7.034520
390	H	0	25.096482	-0.857260	-7.390836
391	H	0	27.147123	-1.379393	-2.435984
392	H	0	5.326522	-0.899883	5.549483
393	H	0	5.926326	4.440552	1.082852
394	H	0	6.772859	6.259938	-0.018631
395	H	0	7.681083	5.904128	-1.494522
396	H	0	8.534973	6.413538	-0.041259
397	H	0	10.539415	4.996945	-0.635619
398	H	0	9.757863	4.440208	-2.113124
399	H	0	10.860590	3.358118	-1.239954
400	H	0	9.855099	1.669305	-0.077227
401	H	0	9.201263	-0.200025	-0.328459
402	H	0	8.298541	-1.535000	0.422416
403	H	0	9.620190	-1.486523	2.427749
404	H	0	11.592921	0.035060	2.251494
405	H	0	11.280016	-1.455106	-0.118740
406	H	0	10.453443	-3.832199	1.169232
407	H	0	12.004840	-3.024251	2.388593
408	H	0	13.815747	-1.118333	2.090847
409	H	0	12.795895	-3.698190	0.052848
410	H	0	13.592398	-2.104899	-0.054611
411	H	0	19.356968	-2.879660	-1.339106
412	H	0	18.776473	-1.386920	-2.108670
413	H	0	20.726500	-1.480510	0.242720

414	H	0	19.199665	0.683539	-0.792123
415	O	0	-13.737903	-10.197836	-4.867526
416	H	0	-13.593383	-11.182351	-4.754747
417	O	0	-9.435019	9.002638	-2.511662
418	H	0	-9.974640	9.573887	-3.130035
419	O	0	-1.407051	-1.338424	9.892841
420	H	0	-1.617676	-1.066312	10.834276
421	O	0	-4.986525	-0.737475	-7.749589
422	H	0	-4.070152	-1.022604	-8.032481
423	O	0	-6.084558	-3.112855	4.572855
424	H	0	-6.267396	-2.651000	5.444379
425	O	0	-18.623632	1.990909	1.196583
426	H	0	-19.458801	1.564823	0.836786
427	N	0	-13.386904	-1.735333	6.464245
428	H	0	-13.465277	-0.993514	7.160316
429	O	0	-4.474939	9.042409	1.808736
430	H	0	-3.837064	9.483964	2.444031
431	O	0	0.989923	-0.806532	-5.989610
432	H	0	1.954719	-0.864140	-6.254682
433	O	0	1.568650	10.811191	-3.763783
434	O	0	8.484659	-0.854300	-4.943100
435	H	0	9.141330	-1.566964	-5.087195
436	O	0	9.111520	-4.341217	3.952515
437	H	0	9.953662	-4.633590	4.363318
438	O	0	4.053256	-1.592121	9.278756
439	H	0	4.241508	-2.554445	9.259669
440	C	0	0.345568	-0.270265	1.113780
441	C	0	1.314492	0.031821	2.079266
442	C	0	2.105886	-1.242925	-0.280131
443	C	0	3.062606	-0.888371	0.680368
444	O	0	4.398431	-1.150535	0.449541

445	H	0	4.501008	-1.572965	-0.428589
446	H	0	1.028754	0.519510	2.998838
447	C	0	0.729590	10.266977	-3.047295
448	H	0	-0.790398	10.725458	-4.197757
449	C	0	2.660138	-0.274145	1.859529
450	H	0	3.408256	-0.014600	2.597913
451	C	0	0.767046	-0.931894	-0.061706
452	H	0	0.033385	-1.192873	-0.819973
453	C	0	-1.052553	0.295861	1.115213
454	H	0	-1.656353	-0.204005	0.350744
455	H	0	-1.567559	0.180185	2.071310
456	C	0	-0.995090	1.816592	0.831212
457	H	0	-1.972866	2.201671	0.533023
458	O	0	2.608757	-1.905798	-1.383683
459	H	0	2.079698	-1.852732	-2.213178
460	H	0	0.939101	1.926834	0.118623
461	H	0	-0.121828	1.628063	-1.159263
462	H	0	0.012219	3.122975	-0.481777
463	N	0	0.000245	2.123738	-0.257089
464	C	0	-0.530270	2.534149	2.112927
465	O	0	-1.405532	2.634356	3.026101
466	O	0	0.693243	2.889575	2.154303

Catechol-O-methyltransferase: Overall Charge = -1, Total energy = -8734.02242902 Ha

1	C	0	3.659471	9.609074	-1.544044
2	C	0	3.992038	8.599042	-2.633956
3	O	0	3.719853	8.821234	-3.815697
4	C	0	2.294929	9.264044	-0.897045
5	C	0	2.300457	7.950473	-0.179016
6	C	0	2.742702	7.719232	1.102896
7	C	0	1.937867	6.664158	-0.723095
8	N	0	2.681096	6.367580	1.390446
9	C	0	2.192546	5.693139	0.287300
10	C	0	1.448141	6.248201	-1.974434
11	C	0	1.964676	4.326896	0.075662
12	C	0	1.203263	4.895165	-2.182111
13	C	0	1.460203	3.949034	-1.162411
14	H	0	4.424626	9.537192	-0.766284
15	H	0	1.522264	9.280473	-1.677463
16	H	0	2.076131	10.087628	-0.211393
17	H	0	3.103869	8.420810	1.838971
18	H	0	2.971248	5.966682	2.275584
19	H	0	1.262938	6.971690	-2.763793
20	H	0	2.208086	3.574164	0.816824
21	H	0	0.816086	4.554677	-3.136686
22	H	0	1.275915	2.897481	-1.351627
23	N	0	4.487742	3.956496	-2.945885
24	C	0	4.399895	2.634488	-2.309127
25	C	0	5.730830	1.896430	-2.088059
26	O	0	5.782183	0.654329	-2.131172
27	C	0	3.417315	1.684237	-3.038603
28	C	0	3.871553	1.358407	-4.457690
29	S	0	2.628234	0.216554	-5.287827

30	C	0	3.383687	0.191229	-6.998706
31	H	0	4.681080	3.937781	-3.940970
32	H	0	3.687293	4.539179	-2.718238
33	H	0	4.010292	2.777984	-1.288554
34	H	0	2.440739	2.179138	-3.073529
35	H	0	3.303067	0.760447	-2.462169
36	H	0	3.933379	2.257441	-5.076953
37	H	0	4.837620	0.850633	-4.453401
38	H	0	3.389505	1.197978	-7.415924
39	H	0	2.765573	-0.460245	-7.614479
40	H	0	4.398285	-0.202924	-6.948031
41	N	0	6.793219	2.633293	-1.793484
42	C	0	8.049350	2.039357	-1.358033
43	C	0	7.894823	1.506023	0.046410
44	O	0	7.121804	2.083379	0.826501
45	C	0	9.159666	3.091255	-1.384783
46	C	0	9.527011	3.457696	-2.812501
47	O	0	9.418669	2.632146	-3.745851
48	N	0	9.997765	4.709158	-2.994538
49	H	0	6.673017	3.640203	-1.780081
50	H	0	8.310549	1.235951	-2.054552
51	H	0	10.053849	2.684926	-0.897067
52	H	0	8.854621	3.972213	-0.809955
53	H	0	10.069882	5.374208	-2.240730
54	H	0	10.300926	4.996935	-3.913500
55	N	0	8.643599	0.476806	0.442225
56	C	0	8.523450	-0.036206	1.818068
57	C	0	8.831190	1.037262	2.851442
58	O	0	8.286128	1.034085	3.966359
59	C	0	9.404638	-1.292255	2.004681
60	C	0	10.902155	-0.968369	1.924966

61	C	0	9.067354	-2.006324	3.318600
62	H	0	9.247787	-0.011927	-0.204384
63	H	0	7.474153	-0.301962	1.991817
64	H	0	9.133845	-1.958291	1.173419
65	H	0	11.161213	-0.406353	1.022604
66	H	0	11.482889	-1.894451	1.926805
67	H	0	11.213091	-0.372818	2.790024
68	H	0	9.314263	-1.375842	4.178544
69	H	0	9.649802	-2.928477	3.395844
70	H	0	8.005486	-2.264055	3.378602
71	N	0	6.025234	-8.239668	-1.745278
72	C	0	5.546148	-6.836547	-1.739381
73	C	0	4.884762	-6.488790	-3.061992
74	O	0	5.032361	-5.351392	-3.553400
75	C	0	6.675085	-5.866083	-1.433092
76	C	0	6.240991	-4.462983	-1.052815
77	O	0	5.048060	-4.269169	-0.575081
78	O	0	7.111829	-3.539493	-1.157360
79	H	0	5.329722	-8.889998	-2.097355
80	H	0	6.378780	-8.528286	-0.839039
81	H	0	4.760976	-6.672510	-0.979384
82	H	0	7.371086	-5.798647	-2.271001
83	H	0	7.234689	-6.264184	-0.576201
84	C	0	-1.633033	-8.549191	-1.929938
85	C	0	-2.379857	-9.861071	-2.156110
86	O	0	-2.129864	-10.825315	-1.419781
87	C	0	-2.322411	-7.761949	-0.800944
88	C	0	-1.646152	-6.408460	-0.554041
89	C	0	-0.121456	-6.484619	-0.425514
90	C	0	0.458169	-5.078563	-0.397961
91	N	0	1.959014	-5.078455	-0.351205

92	H	0	-0.652829	-8.887391	-1.575117
93	H	0	-2.305828	-8.376902	0.105230
94	H	0	-3.371806	-7.591192	-1.070647
95	H	0	-1.895263	-5.740888	-1.386789
96	H	0	-2.069322	-5.964285	0.355299
97	H	0	0.289069	-7.009924	-1.294756
98	H	0	0.164544	-7.041818	0.476424
99	H	0	0.097280	-4.501627	0.458337
100	H	0	0.183038	-4.536064	-1.304242
101	H	0	2.346264	-5.415835	-1.235669
102	H	0	2.322065	-5.648444	0.418666
103	H	0	2.291700	-4.050129	-0.266545
104	N	0	5.464521	-7.684125	3.315517
105	C	0	5.037226	-6.324485	3.589033
106	C	0	3.522582	-6.236105	3.711416
107	O	0	2.786936	-7.001853	3.106197
108	C	0	5.485895	-5.307883	2.523724
109	C	0	5.237818	-3.884226	3.026175
110	O	0	5.522941	-3.604384	4.221005
111	O	0	4.704193	-3.032516	2.201402
112	H	0	5.469311	-8.273163	4.140993
113	H	0	4.923618	-8.114410	2.570457
114	H	0	5.490385	-6.006495	4.532562
115	H	0	4.972774	-5.464433	1.570159
116	H	0	6.560524	-5.444223	2.358074
117	N	0	3.060584	-5.230524	4.461880
118	C	0	1.655768	-4.797451	4.388352
119	C	0	0.664220	-5.779723	4.983729
120	O	0	-0.498776	-5.829252	4.612470
121	C	0	1.246499	-4.330016	2.993765
122	C	0	0.426896	-3.054227	3.072330

123	O	0	-0.248312	-2.782067	4.091162
124	N	0	0.483485	-2.277679	1.973719
125	H	0	3.720932	-4.617103	4.926599
126	H	0	1.582670	-3.926954	5.053596
127	H	0	0.645646	-5.100142	2.498644
128	H	0	2.153450	-4.151849	2.403204
129	H	0	1.199728	-2.413860	1.261850
130	C	0	-3.559660	-2.868306	3.412261
131	C	0	-4.286037	-4.038132	4.047920
132	O	0	-5.489995	-4.198148	4.243008
133	C	0	-4.529100	-1.967300	2.640758
134	C	0	-4.608838	-2.611140	1.228928
135	C	0	-3.447602	-3.638354	1.181346
136	H	0	-3.005882	-2.352115	4.202200
137	H	0	-5.502711	-1.893747	3.130600
138	H	0	-4.099837	-0.965774	2.580157
139	H	0	-4.471054	-1.848018	0.459853
140	H	0	-5.572262	-3.102918	1.064909
141	H	0	-3.847240	-4.666532	1.215941
142	H	0	-2.843853	-3.539761	0.274875
143	C	0	5.575830	4.171015	4.457460
144	C	0	5.054097	5.610632	4.433119
145	O	0	3.962590	5.882654	3.941079
146	C	0	5.912747	3.752576	3.002680
147	C	0	4.704294	3.465895	2.095189
148	C	0	4.331311	1.983625	1.959072
149	O	0	4.576428	1.203526	2.949909
150	O	0	3.732639	1.631864	0.880347
151	H	0	6.529450	4.210832	5.002281
152	H	0	6.539780	4.531457	2.553991
153	H	0	6.520324	2.847556	3.063189

154	H	0	4.883501	3.844488	1.085434
155	H	0	3.815404	3.980803	2.480732
156	C	0	-9.892698	-4.348015	-0.859682
157	C	0	-10.071095	-4.093732	-2.348617
158	O	0	-9.640753	-4.878106	-3.173784
159	C	0	-8.484963	-3.843677	-0.435679
160	C	0	-8.283968	-2.378266	-0.677387
161	C	0	-8.631530	-1.358401	0.176938
162	C	0	-7.771743	-1.752061	-1.877344
163	N	0	-8.370031	-0.135616	-0.417946
164	C	0	-7.835271	-0.344008	-1.674796
165	C	0	-7.284268	-2.243819	-3.101889
166	C	0	-7.417827	0.571507	-2.648163
167	C	0	-6.881606	-1.338658	-4.077122
168	C	0	-6.949001	0.057011	-3.853482
169	H	0	-10.647944	-3.790154	-0.302386
170	H	0	-8.377946	-4.088751	0.624592
171	H	0	-7.736488	-4.431669	-0.982781
172	H	0	-9.053279	-1.410819	1.168565
173	H	0	-8.542102	0.762067	0.005123
174	H	0	-7.218972	-3.309568	-3.299537
175	H	0	-7.477063	1.640551	-2.476150
176	H	0	-6.516695	-1.714981	-5.026813
177	H	0	-6.665293	0.739176	-4.649199
178	N	0	-3.028069	10.154675	-1.825356
179	C	0	-2.630682	8.774200	-2.004445
180	C	0	-3.489076	8.152590	-3.109576
181	O	0	-4.694407	8.047322	-2.931124
182	C	0	-2.747117	7.928414	-0.724045
183	C	0	-2.329515	6.479859	-1.011693
184	C	0	-1.874312	8.527881	0.381317

185	H	0	-2.707877	10.796251	-2.539208
186	H	0	-4.006645	10.280058	-1.591015
187	H	0	-1.582231	8.758053	-2.324296
188	H	0	-3.801704	7.947905	-0.411521
189	H	0	-2.390170	5.876878	-0.100946
190	H	0	-1.292409	6.448686	-1.366885
191	H	0	-2.965785	6.006274	-1.767611
192	H	0	-2.127177	9.575010	0.555723
193	H	0	-0.814999	8.468358	0.098120
194	H	0	-2.004936	7.968695	1.312582
195	C	0	-8.422172	6.414021	1.413001
196	C	0	-9.418476	5.291947	1.674724
197	O	0	-9.608747	4.870017	2.824070
198	C	0	-7.002385	5.847465	1.280182
199	C	0	-6.818976	4.801837	0.164825
200	C	0	-7.357202	5.297728	-1.184773
201	C	0	-5.329429	4.461520	0.036636
202	H	0	-8.700208	6.913397	0.482054
203	H	0	-6.700505	5.416219	2.244701
204	H	0	-6.344577	6.704190	1.087492
205	H	0	-7.366510	3.887536	0.447063
206	H	0	-6.892251	6.257124	-1.445344
207	H	0	-8.442927	5.423868	-1.174693
208	H	0	-7.110573	4.581533	-1.975154
209	H	0	-4.779167	5.353715	-0.285236
210	H	0	-5.163490	3.675114	-0.706690
211	H	0	-4.912565	4.120775	0.989172
212	N	0	-9.615703	2.359000	4.465100
213	C	0	-8.413666	2.490531	5.297702
214	C	0	-8.480811	3.730309	6.175586
215	O	0	-9.386632	4.548132	6.070183

216	C	0	-7.147404	2.649530	4.433394
217	C	0	-6.961181	1.521675	3.431674
218	S	0	-5.253711	1.712931	2.668538
219	C	0	-5.522471	0.864599	1.025774
220	H	0	-9.771195	3.178730	3.881019
221	H	0	-10.443380	2.121264	5.001357
222	H	0	-8.310266	1.607519	5.936145
223	H	0	-6.281593	2.715921	5.101663
224	H	0	-7.214028	3.601532	3.890715
225	H	0	-7.027018	0.539632	3.906436
226	H	0	-7.703621	1.580604	2.634217
227	H	0	-6.106181	1.512728	0.370425
228	H	0	-6.039980	-0.083352	1.184435
229	H	0	-4.539489	0.685464	0.586446
230	Mg	0	4.419868	-2.591907	0.368420
231	C	0	-0.090298	-0.355584	-2.531806
232	C	0	0.353024	0.165971	-1.313219
233	C	0	1.277720	-0.531275	-0.534197
234	C	0	0.442731	-1.570857	-2.978801
235	C	0	1.365401	-2.265173	-2.203807
236	H	0	1.646658	-0.113350	0.396496
237	H	0	-0.146070	-1.492114	1.895045
238	O	0	-5.371085	-4.219566	-4.818543
239	C	0	-4.217478	-3.566352	-4.251897
240	C	0	-3.133017	-3.487118	-5.333147
241	C	0	-3.699949	-4.273858	-2.997924
242	O	0	-3.397731	-5.646706	-3.289087
243	C	0	-4.680448	-4.254624	-1.823925
244	H	0	-5.102241	-4.897753	-5.467357
245	H	0	-4.562068	-2.568947	-3.967092
246	H	0	-3.535987	-3.024455	-6.236952

247	H	0	-2.778689	-4.495996	-5.568351
248	H	0	-2.275183	-2.898578	-4.994610
249	H	0	-2.746601	-3.817937	-2.709730
250	H	0	-4.226762	-6.139106	-3.454185
251	H	0	-4.872249	-3.234730	-1.476383
252	H	0	-4.281692	-4.849616	-0.999955
253	H	0	-5.633302	-4.690403	-2.146202
254	N	0	4.653402	3.197445	5.058969
255	H	0	4.564170	2.351490	4.490640
256	H	0	4.779438	3.046576	6.050087
257	O	0	5.842120	6.666038	4.989036
258	H	0	5.377034	7.518470	4.852833
259	O	0	-3.352241	-9.989343	-3.196035
260	H	0	-3.721484	-10.897207	-3.170445
261	N	0	-1.474834	-7.715902	-3.130046
262	H	0	-1.211885	-8.209624	-3.971420
263	H	0	-2.149589	-6.967030	-3.255069
264	N	0	-10.042762	-5.783962	-0.583303
265	H	0	-11.005017	-6.103120	-0.596346
266	H	0	-9.450033	-6.356928	-1.176102
267	O	0	-10.752927	-2.919662	-2.796796
268	H	0	-10.728109	-2.891685	-3.776586
269	O	0	-10.145168	4.713803	0.587531
270	H	0	-10.724217	3.997299	0.925186
271	N	0	-8.514683	7.392244	2.506317
272	H	0	-8.377976	6.968931	3.419252
273	H	0	-9.356664	7.956457	2.477926
274	N	0	3.645610	10.967542	-2.074211
275	H	0	3.008554	11.076453	-2.857666
276	H	0	4.566526	11.326935	-2.301177
277	O	0	4.643988	7.380534	-2.267366

278	H	0	4.752890	6.801753	-3.051378
279	O	0	-2.878768	7.706340	-4.323174
280	H	0	-3.567211	7.350884	-4.924775
281	O	0	-7.468342	3.965893	7.157886
282	H	0	-7.660454	4.813525	7.610408
283	O	0	4.092830	-7.465214	-3.743116
284	H	0	3.740733	-7.074056	-4.570168
285	O	0	1.082829	-6.684198	6.009956
286	H	0	0.304544	-7.213445	6.282559
287	O	0	-3.358446	-5.052771	4.439751
288	H	0	-3.827136	-5.809364	4.848488
289	N	0	-2.638059	-3.311281	2.365840
290	H	0	-1.972905	-4.013315	2.677463
291	H	0	6.547471	-2.130991	-0.681607
292	O	0	5.884288	-1.469182	-0.286920
293	H	0	5.853796	-0.615373	-0.775617
294	H	0	0.140509	-1.973866	-3.941629
295	H	0	-0.048896	1.109664	-0.957941
296	C	0	1.764579	-1.775364	-0.948511
297	C	0	-1.153089	0.354573	-3.335671
298	H	0	-1.058624	1.439929	-3.205465
299	H	0	-1.028539	0.131886	-4.402042
300	C	0	-2.575505	-0.048589	-2.899104
301	H	0	-2.762985	-1.110725	-3.056262
302	O	0	2.550431	-2.547144	-0.125884
303	O	0	1.917740	-3.482444	-2.600877
304	H	0	1.717063	-3.698555	-3.529986
305	H	0	-4.507816	0.341657	-3.686910
306	H	0	-3.621908	1.662950	-3.173390
307	H	0	-3.287315	0.912982	-4.669679
308	N	0	-3.569271	0.764167	-3.697420

309	O	0	9.758247	1.945596	2.473502
310	H	0	9.931280	2.615285	3.168233
311	C	0	-2.844412	0.350679	-1.426817
312	O	0	-2.711859	-0.534733	-0.537479
313	O	0	-3.159125	1.587381	-1.285321

Aldehyde Dehydrogenase: Overall Charge = 0, Total energy = -6873.95594326 Ha

1	N	0	5.914573	0.990325	-7.002724
2	C	0	6.241699	1.099393	-5.583904
3	C	0	7.494955	0.355005	-5.176409
4	O	0	8.315832	0.890848	-4.402789
5	H	0	6.594959	1.406561	-7.625640
6	H	0	5.632538	0.064821	-7.299475
7	H	0	6.412140	2.138673	-5.306453
8	H	0	5.406489	0.718719	-4.987720
9	N	0	9.858632	2.769074	-2.801603
10	C	0	10.777618	2.403432	-1.739023
11	C	0	11.923210	1.503366	-2.140544
12	O	0	13.002350	1.444275	-1.550723
13	C	0	9.968329	1.686565	-0.605685
14	O	0	8.649918	2.280731	-0.562886
15	C	0	10.627045	1.822789	0.752981
16	H	0	9.570348	2.005370	-3.411331
17	H	0	10.118304	3.600672	-3.317824
18	H	0	11.223620	3.308984	-1.322177
19	H	0	9.863153	0.629353	-0.891603
20	H	0	8.511098	2.661589	-1.474522
21	H	0	10.724477	2.881328	1.012183
22	H	0	11.621531	1.368577	0.747010
23	H	0	10.023376	1.338911	1.524458

24	N	0	1.138389	-8.059644	-0.175003
25	C	0	1.735360	-7.631188	-1.439824
26	C	0	3.126286	-8.244519	-1.431005
27	O	0	4.042513	-7.707669	-0.803482
28	C	0	1.754378	-6.105021	-1.664484
29	C	0	0.325370	-5.627543	-1.782263
30	C	0	-0.435407	-5.344380	-0.638772
31	C	0	-0.306057	-5.594294	-3.034275
32	C	0	-1.805662	-5.082424	-0.738577
33	C	0	-1.668740	-5.309170	-3.142139
34	C	0	-2.424873	-5.068102	-1.990991
35	H	0	1.544107	-7.617049	0.643236
36	H	0	0.126193	-8.047508	-0.162275
37	H	0	1.164036	-8.097981	-2.247533
38	H	0	2.316144	-5.872662	-2.576377
39	H	0	2.269087	-5.634563	-0.819491
40	H	0	0.039431	-5.346266	0.337564
41	H	0	0.273871	-5.807618	-3.927384
42	H	0	-2.380138	-4.892381	0.162808
43	H	0	-2.140956	-5.291648	-4.118237
44	H	0	-3.491824	-4.879574	-2.062571
45	N	0	6.107723	-5.913600	-3.091511
46	C	0	6.199611	-4.647129	-2.350027
47	C	0	6.752420	-4.894507	-0.953970
48	O	0	7.658276	-4.183721	-0.510237
49	C	0	4.830486	-3.941942	-2.267568
50	C	0	4.892046	-2.703482	-1.364880
51	C	0	4.352029	-3.574824	-3.674357
52	H	0	5.529505	-6.599182	-2.610718
53	H	0	7.004507	-6.299563	-3.364093
54	H	0	6.924146	-3.988539	-2.835662

55	H	0	4.133818	-4.668076	-1.826951
56	H	0	5.101235	-2.951097	-0.318677
57	H	0	3.937900	-2.169362	-1.389765
58	H	0	5.673333	-2.013353	-1.707685
59	H	0	3.344458	-3.149456	-3.633534
60	H	0	4.342112	-4.453188	-4.322248
61	H	0	5.020514	-2.825276	-4.116726
62	N	0	10.047657	-3.678312	0.932389
63	C	0	9.944412	-2.881836	2.178038
64	C	0	10.817507	-3.454316	3.277741
65	O	0	11.308314	-2.721599	4.118034
66	C	0	8.473821	-2.808308	2.628387
67	C	0	7.718566	-1.767781	1.851796
68	C	0	8.250197	-0.795927	1.040906
69	C	0	6.292538	-1.544734	1.864777
70	N	0	7.251409	0.034327	0.557309
71	C	0	6.037219	-0.404871	1.048324
72	C	0	5.214757	-2.207488	2.478321
73	C	0	4.738437	0.079942	0.843147
74	C	0	3.926678	-1.732749	2.267676
75	C	0	3.692960	-0.598297	1.457711
76	H	0	9.186848	-3.728572	0.401074
77	H	0	10.864623	-3.471276	0.371795
78	H	0	10.327922	-1.858349	2.078633
79	H	0	8.027880	-3.802157	2.495783
80	H	0	8.428473	-2.575943	3.700177
81	H	0	9.275627	-0.638853	0.751280
82	H	0	7.445045	0.887043	0.032213
83	H	0	5.387433	-3.078423	3.102565
84	H	0	4.555880	0.947738	0.219131
85	H	0	3.084281	-2.235515	2.729092

86	H	0	2.675005	-0.253970	1.309334
87	N	0	-12.377375	3.092009	-2.110183
88	C	0	-11.105873	2.483320	-2.473080
89	C	0	-10.391143	1.999774	-1.214199
90	O	0	-9.778177	0.934564	-1.210425
91	C	0	-10.209727	3.520406	-3.189277
92	C	0	-10.795918	3.975491	-4.481572
93	N	0	-10.835342	3.187926	-5.622583
94	C	0	-11.431960	5.145682	-4.831877
95	C	0	-11.474598	3.895665	-6.605432
96	N	0	-11.850574	5.089171	-6.160320
97	H	0	-13.090331	2.438461	-1.807060
98	H	0	-12.731170	3.744968	-2.802010
99	H	0	-11.201715	1.594963	-3.113618
100	H	0	-9.216368	3.082851	-3.339695
101	H	0	-10.094031	4.386889	-2.530554
102	H	0	-10.447805	2.261193	-5.718482
103	H	0	-11.599872	6.015065	-4.218514
104	H	0	-11.633006	3.509488	-7.596738
105	N	0	-10.511569	2.773958	-0.143954
106	C	0	-9.929367	2.422849	1.143051
107	C	0	-10.262979	1.033527	1.656710
108	O	0	-9.417589	0.392909	2.260674
109	H	0	-11.175107	3.538196	-0.217732
110	H	0	-10.274377	3.151510	1.880331
111	H	0	-8.839052	2.477023	1.102128
112	N	0	-9.704570	-1.990952	-1.888809
113	C	0	-8.438586	-2.151068	-2.588992
114	C	0	-7.902692	-3.575720	-2.443171
115	O	0	-7.912870	-4.141838	-1.345702
116	C	0	-7.366944	-1.183586	-1.981215

117	C	0	-6.006474	-1.058510	-2.625551
118	C	0	-5.599085	0.187188	-3.130255
119	C	0	-5.082010	-2.111496	-2.665143
120	C	0	-4.324166	0.381736	-3.653420
121	C	0	-3.800462	-1.934407	-3.192032
122	C	0	-3.424648	-0.683301	-3.684467
123	O	0	-2.164775	-0.433820	-4.204370
124	H	0	-9.976973	-1.012031	-1.822732
125	H	0	-9.690714	-2.431279	-0.972164
126	H	0	-8.562014	-1.932615	-3.652112
127	H	0	-7.843836	-0.199721	-1.993274
128	H	0	-7.258654	-1.479237	-0.926312
129	H	0	-6.281336	1.029950	-3.070308
130	H	0	-5.346515	-3.088891	-2.273773
131	H	0	-4.009321	1.350525	-4.021592
132	H	0	-3.100220	-2.764116	-3.218711
133	H	0	-1.599457	-1.227566	-4.220852
134	N	0	-5.890034	-5.679604	2.551680
135	C	0	-4.920065	-4.692606	3.002448
136	C	0	-5.069591	-4.498265	4.489677
137	O	0	-6.181202	-4.298136	4.983778
138	C	0	-5.147657	-3.305320	2.380598
139	S	0	-4.914952	-3.390949	0.520035
140	H	0	-6.853876	-5.404764	2.708738
141	H	0	-5.720449	-6.004573	1.608087
142	H	0	-3.905432	-5.014735	2.764232
143	H	0	-6.164823	-2.962727	2.586806
144	H	0	-4.432877	-2.588193	2.781610
145	H	0	-6.235403	-3.386250	0.142564
146	N	0	-4.492960	-1.894224	6.099021
147	C	0	-5.080728	-0.578600	6.352707

148	C	0	-6.507653	-0.402182	5.844115
149	O	0	-7.006610	0.720388	5.840391
150	C	0	-4.185533	0.612203	5.952741
151	C	0	-4.113274	0.830957	4.435415
152	C	0	-2.793645	0.421114	6.570981
153	C	0	-3.313706	2.085270	4.066165
154	H	0	-5.233116	-2.588604	6.035482
155	H	0	-3.879746	-1.926909	5.281263
156	H	0	-5.209945	-0.487669	7.445375
157	H	0	-4.652622	1.498816	6.399306
158	H	0	-5.129291	0.928340	4.032809
159	H	0	-3.652025	-0.046350	3.967231
160	H	0	-2.216296	1.347298	6.516966
161	H	0	-2.241644	-0.359281	6.037072
162	H	0	-2.867434	0.129178	7.623226
163	H	0	-3.724577	2.973101	4.562182
164	H	0	-3.343057	2.249742	2.984154
165	H	0	-2.261774	1.994934	4.356384
166	N	0	-5.323553	5.826849	2.269137
167	C	0	-4.486130	4.824227	1.620696
168	C	0	-3.287323	5.387823	0.881052
169	O	0	-2.788594	4.750001	-0.039285
170	H	0	-4.855878	6.346096	3.001863
171	H	0	-5.813242	6.436658	1.626423
172	H	0	-5.066831	4.266015	0.885201
173	H	0	-4.104019	4.115116	2.360759
174	N	0	0.391917	6.024019	0.771933
175	C	0	1.557417	5.423349	1.397492
176	C	0	2.732409	5.710705	0.468592
177	O	0	2.623165	5.544598	-0.729863
178	C	0	1.369972	3.905123	1.582465

179	C	0	2.620182	3.280951	2.215719
180	C	0	0.121501	3.608064	2.420044
181	H	0	0.186195	5.573690	-0.118732
182	H	0	0.515429	7.023685	0.620476
183	H	0	1.720762	5.885927	2.377883
184	H	0	1.234167	3.477565	0.577404
185	H	0	2.486572	2.203104	2.343911
186	H	0	2.805348	3.717607	3.204578
187	H	0	3.513638	3.436123	1.602111
188	H	0	0.201417	4.084726	3.404394
189	H	0	0.016841	2.529285	2.574181
190	H	0	-0.781368	3.981378	1.930377
191	N	0	3.837094	6.189887	1.014480
192	C	0	4.991980	6.508155	0.187188
193	C	0	6.264582	6.137626	0.938682
194	O	0	6.271506	6.054356	2.166769
195	C	0	5.021105	8.018996	-0.134807
196	O	0	6.219475	8.376074	-0.854840
197	H	0	3.960029	6.272358	2.016271
198	H	0	4.917971	5.944042	-0.744773
199	H	0	4.930029	8.583594	0.799116
200	H	0	4.174895	8.258979	-0.779138
201	H	0	6.970394	8.513159	-0.243045
202	N	0	7.350233	5.941155	0.190429
203	C	0	8.631862	5.534627	0.773187
204	C	0	9.195091	6.541550	1.794473
205	O	0	9.967715	6.166479	2.700560
206	C	0	9.663449	5.408624	-0.354350
207	H	0	7.307109	6.092188	-0.808921
208	H	0	8.521141	4.591811	1.314797
209	H	0	9.792138	6.370783	-0.858139

210	H	0	9.324435	4.664476	-1.076318
211	H	0	10.627609	5.098230	0.052889
212	C	0	-3.401944	0.626380	0.856889
213	C	0	-3.515812	1.699518	-0.035572
214	C	0	-5.921648	1.440268	-0.128576
215	O	0	11.067547	-4.861051	3.332136
216	H	0	11.627687	-5.042100	4.116242
217	O	0	7.738751	-0.961879	-5.676983
218	H	0	8.591756	-1.289858	-5.321640
219	O	0	-7.399276	-4.114405	-3.571601
220	H	0	-7.023202	-5.010756	-3.436927
221	O	0	-2.739953	6.656016	1.250925
222	H	0	-1.780424	6.658766	0.993252
223	O	0	8.822885	7.918561	1.695756
224	H	0	9.271049	8.427504	2.403698
225	O	0	3.368026	-9.452211	-2.157808
226	H	0	4.296374	-9.731783	-2.010807
227	O	0	-3.919166	-4.545144	5.336988
228	H	0	-4.153672	-4.289071	6.254600
229	O	0	-7.246618	-1.534840	5.379548
230	H	0	-8.140020	-1.243156	5.099179
231	O	0	6.218802	-5.954533	-0.156293
232	H	0	6.683470	-5.967534	0.707604
233	O	0	11.611250	0.699783	-3.189688
234	H	0	12.335090	0.083188	-3.425997
235	O	0	-11.529398	0.630553	1.420099
236	H	0	-11.719614	-0.267270	1.763820
237	C	0	-5.820563	0.380075	0.768286
238	O	0	-7.125768	1.881660	-0.654211
239	H	0	-7.874935	1.253669	-0.546328
240	H	0	-6.724714	-0.120835	1.102312

241	H	0	-2.628648	2.236743	-0.353807
242	C	0	-4.754807	2.122188	-0.506523
243	C	0	-4.574347	-0.017618	1.263988
244	H	0	-4.523646	-0.811716	1.995345
245	C	0	-2.029958	0.245783	1.376330
246	H	0	-1.288063	0.889463	0.893907
247	H	0	-1.965723	0.427577	2.455226
248	C	0	-1.617094	-1.215488	1.190271
249	H	0	-0.524384	-1.283274	1.199420
250	O	0	-4.871665	3.222937	-1.336317
251	H	0	-4.059249	3.772775	-1.282800
252	H	0	-1.930645	-2.800227	-0.093680
253	H	0	-3.064172	-1.598203	-0.290049
254	H	0	-1.530714	-1.382624	-0.927382
255	N	0	-2.057280	-1.773192	-0.141354
256	C	0	-2.126808	-2.209730	2.269128
257	O	0	-2.168384	-3.428939	1.890901
258	O	0	-2.427150	-1.742032	3.410547

Sult1a3: Overall Charge = +1, Total energy = -5672.71362702 Ha

1	N	0	-0.574542	6.313455	5.122345
2	C	0	-1.884773	5.720925	5.414923
3	C	0	-2.431518	6.227131	6.742210
4	O	0	-3.607555	6.581041	6.845813
5	C	0	-1.797470	4.191611	5.485036
6	C	0	-1.715401	3.513528	4.136211
7	C	0	-1.634118	4.237738	2.942963
8	C	0	-1.773838	2.113149	4.075340
9	C	0	-1.635194	3.579084	1.713595
10	C	0	-1.758028	1.450361	2.848047
11	C	0	-1.691535	2.184851	1.660235
12	H	0	-0.596987	7.306482	4.923000
13	H	0	0.137209	6.074316	5.804088
14	H	0	-2.610045	6.033883	4.662511
15	H	0	-0.930755	3.925791	6.104540
16	H	0	-2.680491	3.808768	6.012625
17	H	0	-1.554078	5.317170	2.968495
18	H	0	-1.843541	1.544402	4.998252
19	H	0	-1.603534	4.130388	0.781271
20	H	0	-1.813680	0.366541	2.820678
21	H	0	-1.693265	1.696002	0.692594
22	C	0	-1.555160	-7.174357	1.473213
23	C	0	-0.957169	-8.412872	0.825237
24	O	0	-0.511270	-9.317084	1.520443
25	C	0	-0.421449	-6.305906	2.098757
26	C	0	-1.024742	-5.822238	3.428882
27	C	0	-1.857647	-7.025133	3.873614
28	H	0	-2.057886	-6.608342	0.695154
29	H	0	-0.126523	-5.493385	1.433683

30	H	0	0.454970	-6.930245	2.298672
31	H	0	-1.674307	-4.956072	3.262472
32	H	0	-0.258649	-5.546985	4.155272
33	H	0	-1.212879	-7.800862	4.307350
34	H	0	-2.635003	-6.769011	4.595382
35	N	0	-8.134666	2.990969	-4.135556
36	C	0	-7.540617	2.424842	-2.943328
37	C	0	-7.934910	3.370974	-1.810369
38	O	0	-7.361013	4.446943	-1.656768
39	C	0	-6.038517	2.321383	-3.125456
40	C	0	-5.318838	1.527231	-2.064991
41	C	0	-5.977158	0.984712	-0.953470
42	C	0	-3.932497	1.344024	-2.191566
43	C	0	-5.254907	0.293441	0.026270
44	C	0	-3.216574	0.647816	-1.222446
45	C	0	-3.875579	0.130141	-0.101938
46	H	0	-7.771113	3.902456	-4.391195
47	H	0	-9.145282	2.944198	-4.184445
48	H	0	-7.986032	1.444921	-2.752728
49	H	0	-5.868129	1.873158	-4.110721
50	H	0	-5.624023	3.337211	-3.167041
51	H	0	-7.050923	1.100471	-0.826474
52	H	0	-3.387507	1.788592	-3.020010
53	H	0	-5.777906	-0.109540	0.887625
54	H	0	-2.143178	0.547770	-1.331730
55	H	0	-3.306080	-0.383030	0.665561
56	N	0	-0.521977	8.660907	-1.248517
57	C	0	0.830176	8.753543	-1.785970
58	C	0	1.235332	10.161432	-2.237939
59	O	0	0.393557	10.968877	-2.641929
60	C	0	1.109826	7.796059	-2.967076

61	C	0	1.057841	6.315969	-2.573588
62	O	0	1.106105	5.449916	-3.489421
63	O	0	0.999979	6.046910	-1.303376
64	H	0	-0.675221	7.730286	-0.872465
65	H	0	-1.238843	8.931923	-1.915057
66	H	0	1.516311	8.461739	-0.985416
67	H	0	2.104638	7.984238	-3.390351
68	H	0	0.381969	7.969182	-3.768338
69	N	0	-11.072605	-0.585393	-2.948812
70	C	0	-10.009630	-1.454549	-2.455994
71	C	0	-9.507275	-0.986040	-1.088311
72	O	0	-9.483270	0.207194	-0.807444
73	C	0	-8.810204	-1.372012	-3.432982
74	C	0	-7.476489	-1.859952	-2.858942
75	C	0	-6.298048	-1.586645	-3.798894
76	C	0	-5.004881	-2.036508	-3.134500
77	N	0	-3.791758	-1.762743	-3.979456
78	H	0	-11.972534	-0.742443	-2.507726
79	H	0	-10.810835	0.395923	-2.910815
80	H	0	-10.368545	-2.481867	-2.372679
81	H	0	-8.711020	-0.318055	-3.729034
82	H	0	-9.070826	-1.928715	-4.338456
83	H	0	-7.534376	-2.930996	-2.627433
84	H	0	-7.262382	-1.347680	-1.911439
85	H	0	-6.438333	-2.112428	-4.751348
86	H	0	-6.249568	-0.511788	-4.019403
87	H	0	-5.015744	-3.110389	-2.935062
88	H	0	-4.845947	-1.517212	-2.183365
89	H	0	-3.918891	-2.070964	-4.947421
90	H	0	-2.984991	-2.285615	-3.553361
91	H	0	-3.560325	-0.764168	-3.986245

92	N	0	-7.011214	-2.543431	2.672861
93	C	0	-6.331341	-3.583284	3.438823
94	C	0	-6.600032	-3.351179	4.925856
95	O	0	-6.109089	-4.103792	5.768907
96	C	0	-4.819341	-3.655964	3.182091
97	C	0	-4.465186	-4.020669	1.780361
98	N	0	-4.102887	-5.297584	1.363629
99	C	0	-4.390888	-3.232165	0.658495
100	C	0	-3.826921	-5.242566	0.031714
101	N	0	-3.970523	-3.998333	-0.422224
102	H	0	-8.007869	-2.530016	2.862351
103	H	0	-6.613037	-1.621361	2.827466
104	H	0	-6.794183	-4.550805	3.214505
105	H	0	-4.390160	-4.373817	3.887648
106	H	0	-4.381035	-2.678645	3.414467
107	H	0	-3.892293	-6.111429	1.962877
108	H	0	-4.608982	-2.184974	0.559697
109	H	0	-3.530690	-6.088090	-0.562800
110	N	0	7.865288	-7.213896	-2.068891
111	C	0	7.087842	-5.996075	-2.258809
112	C	0	7.730662	-4.867696	-1.455069
113	O	0	7.760562	-3.721886	-1.887785
114	C	0	5.620262	-6.184131	-1.817199
115	C	0	4.768282	-4.976854	-2.134723
116	C	0	4.237099	-4.818672	-3.422670
117	C	0	4.541936	-3.971825	-1.185165
118	C	0	3.499107	-3.682102	-3.757222
119	C	0	3.808007	-2.830759	-1.519404
120	C	0	3.284751	-2.680398	-2.806518
121	H	0	8.759748	-7.216119	-2.544146
122	H	0	7.953163	-7.488076	-1.096781

123	H	0	7.120638	-5.690731	-3.306186
124	H	0	5.601866	-6.405499	-0.743447
125	H	0	5.252731	-7.069228	-2.344901
126	H	0	4.406124	-5.593524	-4.165165
127	H	0	4.951096	-4.079145	-0.183560
128	H	0	3.095493	-3.577179	-4.758212
129	H	0	3.665511	-2.039919	-0.790877
130	H	0	2.736547	-1.778915	-3.057068
131	N	0	8.749733	-1.542957	-3.327024
132	C	0	7.837057	-0.458837	-2.978024
133	C	0	8.567447	0.463911	-2.012851
134	O	0	8.710384	0.158983	-0.829974
135	C	0	6.468524	-0.838178	-2.422753
136	C	0	5.532423	0.353935	-2.692495
137	C	0	4.269439	0.432970	-1.852932
138	O	0	3.160812	0.576932	-2.444237
139	O	0	4.415059	0.410968	-0.561354
140	H	0	9.626579	-1.247737	-3.735891
141	H	0	8.814371	-2.284190	-2.641648
142	H	0	7.672789	0.120283	-3.894205
143	H	0	6.532095	-1.063177	-1.352854
144	H	0	6.111275	-1.739123	-2.927536
145	H	0	6.081268	1.291862	-2.506501
146	H	0	5.245440	0.364037	-3.746915
147	N	0	7.806499	2.970634	-0.407109
148	C	0	7.050170	3.596231	0.667280
149	C	0	7.120519	2.781195	1.971435
150	O	0	6.861100	3.300813	3.061589
151	C	0	5.586529	3.764751	0.251038
152	H	0	7.276395	2.297679	-0.945664
153	H	0	8.728699	2.645402	-0.142024

154	H	0	7.481243	4.572556	0.909187
155	H	0	5.144863	2.785045	0.028454
156	H	0	5.527771	4.391071	-0.642130
157	H	0	5.015158	4.236186	1.053533
158	N	0	7.480751	1.507959	1.859691
159	C	0	7.584284	0.656953	3.033219
160	C	0	8.966847	0.785160	3.650972
161	O	0	9.936587	1.087677	2.955278
162	C	0	7.311515	-0.822329	2.665378
163	C	0	5.852526	-1.126409	2.611744
164	N	0	4.984180	-0.711393	1.606528
165	C	0	5.084575	-1.786712	3.535136
166	C	0	3.738933	-1.095459	1.900811
167	H	0	7.796062	1.133101	0.969933
168	H	0	6.851834	0.994159	3.771323
169	H	0	7.758554	-1.467273	3.426422
170	H	0	7.803847	-1.051816	1.713237
171	H	0	5.135187	-0.197473	0.693601
172	H	0	5.356866	-2.261531	4.459935
173	H	0	2.868649	-0.903504	1.298522
174	C	0	-1.396489	-2.175225	-1.664225
175	C	0	-0.653750	-1.435968	-2.597729
176	C	0	0.278900	-0.057153	-0.843656
177	C	0	-0.400987	-0.846439	0.093200
178	O	0	-2.308642	-3.082546	-2.168689
179	H	0	-0.327828	-0.601534	1.148899
180	H	0	-3.010779	-3.459565	-1.484407
181	O	0	-7.768398	-2.578910	5.213146
182	H	0	-7.963340	-2.619124	6.173200
183	O	0	-0.934651	-8.484508	-0.602527
184	H	0	-0.492507	-9.313364	-0.882958

185	O	0	8.194555	-5.229858	-0.227454
186	H	0	8.568881	-4.475851	0.274055
187	O	0	-1.573875	6.177903	7.792733
188	H	0	-1.982552	6.487392	8.627530
189	O	0	-9.060482	3.014839	-1.139475
190	H	0	-9.346763	3.701370	-0.499927
191	O	0	-9.094187	-1.983194	-0.254587
192	H	0	-8.661404	-1.663668	0.566370
193	O	0	2.576499	10.410717	-2.250824
194	H	0	2.782870	11.292976	-2.624380
195	O	0	9.000243	1.606807	-2.589516
196	H	0	9.508401	2.211208	-2.009727
197	O	0	9.030373	0.442001	4.965104
198	H	0	9.942913	0.465547	5.321502
199	N	0	-2.469651	-7.462149	2.597707
200	H	0	-2.866932	-8.396697	2.602507
201	N	0	3.779691	-1.753428	3.069478
202	H	0	2.981097	-2.160637	3.535094
203	C	0	-1.215948	-1.912260	-0.308144
204	H	0	-1.760882	-2.494103	0.427779
205	C	0	0.194030	-0.413927	-2.197349
206	H	0	0.725089	0.172278	-2.935880
207	O	0	-0.883374	-1.745993	-3.936672
208	H	0	-0.376332	-1.169701	-4.536988
209	C	0	1.026131	1.177473	-0.394240
210	H	0	0.665016	1.430692	0.610734
211	H	0	2.100988	0.965097	-0.328309
212	C	0	0.877828	2.441869	-1.287247
213	H	0	1.624409	2.411379	-2.081427
214	H	0	2.065134	3.539683	0.061816
215	H	0	1.106652	4.613838	-0.917541

216	H	0	0.411003	3.664780	0.296072
217	N	0	1.160482	3.627569	-0.406023
218	C	0	-0.516038	2.683068	-1.914503
219	O	0	-0.739311	2.173248	-3.057688
220	O	0	-1.325140	3.385314	-1.217910

Table S1. Counterpoise corrected M062X/6-311+g* energies for PCM with each amino acid in the active site of each enzyme studied, as well as the calculated interaction energy. In each table, the first row is the BSSE-corrected energy for PCM, the second row is the BSSE-corrected energy for the amino acid residue, the third row is the energy for the complex of PCM and amino acid residue, and the fourth row is the difference between the complex and the 2 components, converted into kcal/mol. At the end of each set of rows is the total interaction energy.

MAOB-PCM	Cysteine	Glutamine	Isoleucine	Isoleucine 2	Leucine	Leucine 2	Leucine 3	Phenylalanine 3	Phenylalanine 2	Phenylalanine	tyrosine 4	tyrosine 3	tyrosine 2	tyrosine	Proline	Proline 2	Tryptophan	FAD		
Ligand	-515.395549	-515.396057	-515.3955464	-515.3958344	-515.395283	-441.5921115	-515.395293	-515.3952911	-515.3953096	-515.3955382	-515.3956659	-515.3955468	-515.3967341	-515.3960885	-515.395285	-515.395286	-515.3952863	-515.3967106		
AA	-721.8648341	-531.6823228	-441.5674853	-441.5542436	-441.595876	-515.3954466	-366.34492	-554.65998	-554.689459	-554.6779245	-629.9157421	-629.912052	-629.9103332	-629.914855	-401.062358	-401.069086	-686.2510747	-3351.313381		
Total	-1237.262571	-1047.084787	-956.9651239	-956.9537724	-956.991191	-956.9871489	-881.740079	-1070.055525	-1070.085311	-1070.073953	-1145.31224	-1145.30783	-1145.313022	-1145.315821	-916.457331	-916.464405	-1201.646366	-3866.719463		Total
IE	-1.372935565	-4.020775279	-1.312924322	-2.318287412	-0.01970694	0.256788183	0.084155299	-0.159613316	-0.340078773	-0.307896321	-0.522055901	-0.145248996	-3.736850448	-3.060761673	0.195957412	-0.02092179	-0.002879014	-5.880358894		-22.68439375

ALDH-PCM	Alanine	Cysteine	Glycine	Glycine 2	Glycine 3	Histidine	Isoleucine	Phenylalanine	Serine	Threonine	Tryptophan	Tyrosine	Valine	Valine 2		
Ligand	-515.3946666	-515.3949151	-515.3954116	-515.395116	-515.394661	-515.3953846	-515.394695	-515.3948855	-515.3946592	-515.3946594	-515.394809	-515.3964834	-515.3950329	-515.395144		
AA	-323.6796858	-721.8616177	-284.3790002	-284.3835197	-284.378469	-548.6578244	-402.293111	-554.6929256	-398.8491991	-438.2124017	-686.2415797	-629.9175939	-402.272907	-441.5920741		
Total	-839.0746763	-1237.258113	-799.782726	-799.7822805	-799.773148	-1064.062122	-917.687887	-1070.088385	-914.2443062	-953.6066334	-1201.638287	-1145.317459	-917.6719629	-956.9893883		Total
IE	-0.203227737	-0.991791315	-5.217236347	-2.287127173	-0.01111131	-5.593118303	-0.05086592	-0.360238771	-0.281081585	0.268462997	-1.190923925	-2.122014363	-2.52440734	-1.361842452		-18.0402738

PheOH-PCM	Arginine	Glutamic acid	Glutamic acid 2	Glycine	Histidine	Phenylalanine	Proline	Proline 2	Serine	Serine 2	Threonine	Tryptophan	Tyrosine	Tyrosine 2		
Ligand	-515.3882389	-515.3884674	-515.389026	-515.3883832	-515.389426	-515.3885175	-515.38851	-515.3885708	-515.3882826	-515.3882863	-515.3887143	-515.3888992	-515.3884472	-515.3885975		
AA	-606.8186668	-550.9784443	-550.9819398	-284.3854375	-548.591531	-554.6993002	-401.055876	-401.0656318	-398.9011138	-398.8588593	-438.1598674	-686.2518512	-629.9139344	-629.8811472		
Total	-1122.201086	-1066.378685	-1066.409622	-799.7744477	-1063.98381	-1070.091585	-916.445449	-916.4556606	-914.290286	-914.2506067	-953.5558416	-1201.64486	-1145.301764	-1145.273103		Total
IE	3.651681721	-7.387657421	-24.25688173	-0.393457869	-1.78830983	-2.364184712	-0.66701185	-0.914936461	-0.558186643	-2.171861208	-4.555599116	-2.578778528	0.38758689	-2.107122308		-45.7047191

DDC-PCM	Histidine	Histidine 2	Isoleucine	Lysine	Phenylalanine	Phenylalanine 2	Phenylalanine 3	PLP	Proline	Threonine	Threonine 2	Tryptophan	Tyrosine		
Ligand	-515.3935031	-515.3940601	-515.3938077	-515.3940515	-515.394582	-515.3945857	-515.393512	-515.3944657	-515.3937608	-515.3936388	-515.3935416	-515.3944842	-515.3943908		
AA	-548.6304562	-548.673326	-441.5951773	-497.2799155	-554.695431	-554.6608619	-554.683388	-1156.999788	-400.9517272	-438.1978757	-438.2033436	-686.2518391	-629.8767937		
Total	-1064.023838	-1064.078718	-956.9901204	-1012.687508	-1070.09884	-1070.065938	-1070.07712	-1672.396418	-916.3441952	-953.5922837	-953.5961143	-1201.648389	-1145.284282		Total
IE	0.076098077	-7.110579346	-0.712493112	-8.497243564	-5.54018537	-6.583135021	-0.13723821	-1.357588565	0.811222946	-0.482707918	0.483705658	-1.296383799	-8.218763006		-38.5652912

Sult1a3-PCM	Alanine	Aspartic acid	Glutamic acid	Histidine	Histidine 2	Lysine	Phenylalanine	Phenylalanine 2	Phenylalanine 3	Proline		
Ligand	-514.8285107	-514.8286972	-514.8289917	-514.829066	-514.828546	-514.8290116	-514.829228	-514.829588	-514.8288885	-514.8285138		
AA	-323.6628728	-511.7179036	-550.9873594	-549.0360433	-548.680055	-497.2843592	-554.696512	-554.697447	-554.6972446	-401.0842667		
Total	-838.4923214	-1026.482886	-1065.738006	-1064.047414	-1063.51065	-1012.324592	-1069.53662	-1069.535721	-1069.529154	-915.9148359		Total
IE	-0.588545553	39.98146847	49.16222613	-114.3975986	-1.28553353	-132.5430887	-6.83051558	-5.450435193	-1.895737575	-1.289793694		-175.1375538

TyOH-PCM	Arginine	Glutamic acid	Glutamic acid 2	Glutamic acid 3	Glycine	Glycine 2	Histidine	Histidine 2	Iron	Leucine	Leucine 2	Leucine 3	Phenylalanine	Pheylalanine 2	Proline	Serine	Serine 2	Serine 3	Threonine	Tryptophan	Tyrosine	Tyrosine 2	Valine		
Ligand	-515.3910617	-515.3910834	-515.3917807	-515.3915076	-515.391419	-515.3910696	-515.391565	-515.3919646	-1262.554231	-515.3910689	-515.3914203	-515.3918397	-515.3918484	-515.3910713	-515.391612	-515.391071	-515.3910599	-515.3910421	-515.3912498	-515.3911791	-515.3910807	-515.391876	-402.2930102		
AA	-606.8170123	-550.9666678	-550.9934293	-550.9683325	-284.344438	-284.3825537	-548.649888	-548.6069898	-515.3931639	-441.5980728	-441.5727042	-441.5446775	-554.6604387	-554.6990304	-401.05085	-398.879921	-398.8946756	-398.8566728	-438.2006712	-686.2479479	-629.9138059	-629.8782394	-515.3914828		
Total	-1122.212651	-1066.353399	-1066.385652	-1066.358929	-799.740368	-799.7733383	-1064.03788	-1063.998764	-1778.174447	-956.9890098	-956.9631046	-956.940652	-1070.052579	-1070.08999	-916.4445	-914.271892	-914.2859232	-914.2473733	-953.5930465	-1201.639091	-1145.304695	-1145.279127	-917.6823264		Total
IE	-2.872312412	2.730871771	-0.277162161	0.57187639	-2.83060186	0.178846483	2.244159087	0.119443923	-142.4778367	0.082720185	0.639965564	-2.594616868	-0.183346353	0.070172505	-1.27878655	-0.5650949	-0.117722037	0.214369168	-0.706234332	0.022827541	0.120554615	-5.65487214	1.359579653		-152.56278

Tyronaise-PCM	Alanine	Asparagine	Copper	Glycine	Histidine	Histidine 2	Histidine 3	Histidine 4	Histidine 5	Histidine 6	Methionine	Phenylalanine	Phenylalanine 2	Valine		
Ligand	-515.3883039	-515.3883576	-515.390161	-515.3882999	-515.389022	-515.3885055	-515.388674	-515.3884109	-515.3885239	-515.3896747	-515.3882258	-515.3880897	-515.3882042	-515.3885694		
AA	-323.6888806	-492.3782897	-1639.367183	-284.3444318	-548.656758	-548.6871917	-548.640816	-548.6837754	-548.6286444	-548.6899096	-800.4469742	-554.697261	-554.693692	-402.2746757		
Total	-839.0760144	-1007.768072	-2155.143407	-799.7328278	-1064.04244	-1064.073489	-1064.03324	-1064.06775	-1064.017193	-1064.083009	-1315.839021	-1070.085736	-1070.081954	-917.6629418		Total
IE	0.734299067	-0.894028472	-242.2583708	-0.060318723	2.09473276	1.38578696	-2.35172112	2.783523583	-0.015234048	-2.149297221	-2.397928408	-0.241862869	-0.036416886	0.190287863		-243.216548

COMT-PCM	Asparagine	Asparagine 2	Aspartic acid	Aspartic acid 2	Glutamic acid	Leucine	Lysine	Magnesium	Methionine	Methionine 2	Proline	Threonine	Tryptophan	Tryptophan 2	Valine	Valine 2		
Ligand	-514.8272222	-514.8277644	-514.8274251	-514.8273627	-514.827369	-514.8272099	-514.828105	-514.827969	-514.827405	-514.8278333	-514.8277849	-514.8276775	-514.8275453	-514.8276014	-514.827258	-514.827315		
AA	-492.3600661	-492.3615856	-511.6902308	-511.6652579	-551.001747	-402.2836152	-497.284349	-199.2090243	-800.4702777	-800.4374334	-401.0854352	-438.1441462	-686.2503587	-686.2525475	-402.293948	-441.595501		
Total	-1007.190351	-1007.229473	-1026.404972	-1026.386555	-1065.73867	-917.1148783	-1012.30474	-714.5958222	-1315.302163	-1315.26566	-915.919455	-952.981375	-1201.08111	-1201.081302	-917.121761	-956.424082		Total
IE	-1.922092974	-25.17779271	70.71023218	66.55746467	56.75423641	-2.5434077	-120.660386	-350.6704411	-2.811320999	-0.246685907	-3.912488475	-5.993494464	-2.011498645	-0.723881781	-0.34881935	-0.79506646		-323.7954429

Table S2. Counterpoise corrected M062X/6-311+g* energies for NAPQI with each amino acid in the active site of each enzyme studied, as well as the calculated interaction energy. In each table, the first row is the BSSE-corrected energy for NAPQI, the second row is the BSSE-corrected energy for the amino acid residue, the third row is the energy for the complex of NAPQI and amino acid residue, and the fourth row is the difference between the complex and the 2 components, converted into kcal/mol. At the end of each set of rows is the total interaction energy.

MAOB-NAPQI	Cysteine	Glutamine	Isoleucine	Isoleucine 2	leucine	Leucine 2	Leucine 3	Phenylalanine	Phenylalanine 2	Phenylalanine 3	Tyrosine	Tyrosine 2	Tyrosine 3	Tyrosine 4	Proline	Proline 2	Tryptophan	FAD	
Ligand	-514.017028	-514.018429	-514.0172817	-514.016648	-514.016806	-514.0169891	-514.016644	-514.0166641	-514.016647	-514.0171233	-514.016861	-514.017396	-514.0175283	-514.0170891	-514.01664	-514.01665	-514.0145687	-514.017175	
AA	-721.848692	-531.674213	-441.5147999	-441.586784	-441.591912	-441.5877481	-441.595878	-554.6894441	-554.6612402	-554.682792	-629.915552	-629.910799	-629.914032	-629.9098172	-400.896832	-401.06655	-686.2510768	-3351.31308	
Total	-1235.87313	-1045.69587	-955.5329538	-955.603345	-955.610751	-955.6066627	-955.612519	-1068.70544	-1068.677913	-1068.701328	-1143.93229	-1143.93176	-1143.936983	-1143.929906	-914.913531	-915.08319	-1200.265498	-3865.3387	Total
IE	-4.6480206	-4.53343736	-0.547299353	0.064570109	-1.27572995	-1.208262012	0.00193147	0.419049589	-0.016225513	-0.886386662	0.075567832	-2.23797561	-3.402898677	-1.88204971	-0.03742529	0.00339294	0.092759709	-5.29935726	-25.32779635

ALDH-NAPQI	Alanine	Cysteine	Glycine	Glycine 2	Glycine 3	Histidine	Isoleucine	Phenylalanine	Serine	Theronine	Tryptophan	Tyrosine	Valine	Valine 2	
Ligand	-514.137744	-514.13794	-514.137769	-514.138204	-514.137804	-514.1378169	-514.137997	-514.1385221	-514.1377521	-514.1377591	-514.13784	-514.139701	-514.1379478	-514.1379483	
AA	-323.679661	-721.864191	-284.3784868	-284.378727	-284.383086	-548.6576126	-441.592168	-554.6932049	-398.8597138	-438.2122794	-686.246083	-629.91555	-402.293247	-402.2727218	
Total	-837.81727	-1236.00444	-798.5162561	-798.522419	-798.521836	-1062.795177	-955.730478	-1068.838554	-912.9972249	-952.3502667	-1200.38423	-1144.08062	-916.4313206	-916.410774	Total:
IE	0.084713155	-1.45020269	-0.000219001	-3.44338747	-0.59377145	0.158116706	-0.19647448	-4.283775805	0.151204689	-0.14315939	-0.19031234	-15.9190068	-0.078988386	-0.065257223	-25.97052

PheOH-NAPQI	Arginine	Glutamic acid	Glutamic acid 2	Glycine	Histidine	Phenylalanine	Proline	Proline 2	Serine	Serine 2	Threonine	Tryptophan	Tyrosine	Tyrosine 2	
Ligand	-514.132325	-514.13245	-514.1327876	-514.132397	-514.133297	-514.1323475	-514.132475	-514.1330767	-514.1322027	-514.1323347	-514.132448	-514.132692	-514.1329153	-514.1329153	
AA	-606.825985	-550.967762	-550.9513061	-284.3881	-548.690778	-554.6985701	-401.063001	-401.0738992	-398.9011945	-398.8631957	-438.162296	-686.251439	-629.9149527	-629.9149527	
Total	-1120.95697	-1065.12609	-1065.091374	-798.519035	-1062.83031	-1068.832071	-915.201011	-915.2111006	-913.0341967	-912.996392	-952.298285	-1200.38617	-1144.058144	-1144.058144	Total
IE	0.839115744	-16.2387856	-4.569369812	0.917759627	-3.91041895	-0.723489588	-3.47320296	-2.588228821	-0.501721456	-0.540697953	-2.22251377	-1.28070422	-6.448090584	-6.448090584	-47.188439

DDC-NAPQI	Histidine	Histidine 2	Isoleucine	Lysine	Phenylalanine	Phenylalanine 2	Phenylalanine	PLP	Proline	Threonine	Threonine 2	Tryptophan	Tyrosine	
Ligand	-514.07164	-514.071595	-514.0719536	-514.072061	-514.071921	-514.0712304	-514.071231	-514.0727538	-514.0712153	-514.070915	-514.071862	-514.071457	-514.0715979	
AA	-549.063798	-548.650248	-441.5969912	-497.281168	-554.695753	-554.6841137	-554.658574	-1158.221414	-401.0072654	-438.2023968	-438.196561	-686.250789	-629.8795295	
Total	-1063.15875	-1062.7222	-955.6701832	-1011.35776	-1068.76901	-1068.7582	-1068.73334	-1672.281085	-915.0775828	-952.2730708	-952.27521	-1200.32312	-1143.960216	Total
IE	-20.9048013	-0.22400897	-0.777161103	-2.84541359	-0.83866143	-1.792221725	-2.2190311	8.209001468	0.563443918	0.151195904	-4.25858255	-0.54979559	-5.703403725	-31.1894398

Sult1a3-NAPQI	Alanine	Aspartic acid	Glutamic acid	Histidine	Histidine 2	Lysine	Phenylalanine	Phenylalanine 2	Phenylalanine 3	Proline	
Ligand	-514.138607	-514.138697	-514.1389914	-514.139047	-514.138773	-514.1389834	-514.139235	-514.1394089	-514.1391712	-514.1386667	
AA	-323.665646	-511.717217	-550.9897565	-549.064559	-548.672574	-497.2894548	-554.695984	-554.6964261	-554.6972037	-401.084407	
Total	-837.803632	-1025.85885	-1065.159173	-1063.20427	-1062.80959	-1011.458865	-1068.8411	-1068.838017	-1068.838631	-915.2227596	Total
IE	0.389753681	-1.84050984	-19.09225013	-0.41541129	1.10165003	-19.09284313	-3.06410755	-1.36945477	-1.415775639	0.197067476	-44.6018812

TyOH-NAPQI	Arginine	Glutamic acid	Glutamic acid 2	Glutamic acid 3	Glycine	Glycine 2	Histidine	Histidine 2	Iron	Leucine	Leucine 2	Leucine 3	Phenylalanine	Pheylalanine 2	Proline	Serine	Serine 2	Serine 3	Threonine	Tryptophan	Tyrosine	Tyrosine 2	Valine	
Ligand	-514.141183	-514.141211	-514.1421563	-514.141379	-514.141522	-514.1411893	-514.141722	-514.1417216	-514.1419006	-514.1411796	-514.141635	-514.141839	-514.1419768	-514.1411773	-514.141868	-514.14119	-514.1411778	-514.141174	-514.141565	-514.1412734	-514.1412157	-514.141403	-514.1417394	
AA	-606.817361	-550.964426	-551.5572171	-550.966009	-284.344058	-284.3824889	-548.652142	-548.6521417	-1262.564234	-441.5981906	-441.571582	-441.544525	-554.6620899	-554.6990678	-401.035274	-398.88583	-398.8946787	-398.855094	-438.2055042	-686.2472941	-629.9090911	-629.8714793	-402.2995939	
Total	-1120.9618	-1065.10234	-1065.702365	-1065.10134	-798.490159	-798.5233447	-1062.78856	-1062.788564	-1776.924005	-955.7392043	-955.714884	-955.692615	-1068.807927	-1068.84017	-915.178884	-913.02685	-913.0359745	-912.996125	-952.3492148	-1200.388278	-1144.049857	-1144.015576	-916.4422144	Total
IE	-2.04610077	2.068017171	-1.878164799	3.796208454	-2.87300142	0.209315834	3.32533599	3.325335993	-142.9912217	0.104097551	-1.04576152	-3.92204482	-2.422251931	0.046831034	-1.09296718	0.11209579	-0.074051769	0.089785314	-1.346415131	0.181494573	0.282058618	-1.690346813	-0.552890463	-148.39464

Tyronaise-NAPQ	Alanine	Asparagine	Copper	Glycine	Histidine	Histidine 2	Histidine 3	Histidine 4	Histidine 5	Histidine 6	Methionine	Phenylalanine	Phenylalanine 2	Valine	
Ligand	-514.139767	-514.139999	-514.14017	-514.139687	-514.139677	-514.1396138	-514.139918	-514.1399914	-514.1412076	-514.1399796	-514.139695	-514.139538	-514.1397886	-514.1399093	
AA	-323.689064	-492.377383	-1639.367178	-284.344065	-548.629592	-548.6820089	-548.656837	-548.6426273	-548.6890788	-548.6881294	-800.447221	-554.697264	-554.6977821	-402.2743461	
Total	-837.82763	-1006.52352	-2153.869531	-798.484689	-1062.7693	-1062.818015	-1062.79244	-1062.781847	-1062.838362	-1062.822315	-1314.5899	-1068.83739	-1068.83884	-916.4150314	Total
IE	0.753638909	-3.85453985	-227.2732337	-0.58822741	-0.02014306	2.263866023	2.71040178	0.484249081	-5.067515718	3.635790043	-1.87474738	-0.36790882	-0.796497808	-0.486947372	-230.48182

COMT-NAPQI	Asparagine	Asparagine 2	Aspartic acid	Aspartic acid 2	Glutamic acid	Leucine	Lysine	Magnesium	Methionine	Methionine 2	Proline	Threonine	Tryptophan	Tryptophan 2	Valine	Valine 2	
Ligand	-514.13789	-514.137363	-514.1375246	-514.137409	-514.137601	-514.1375597	-514.137869	-514.1378788	-514.1376031	-514.1380962	-514.137946	-514.137502	-514.1380981	-514.1373713	-514.137547	-514.13733	
AA	-492.368226	-492.353214	-511.6918801	-511.667741	-551.004692	-441.5994689	-497.290804	-199.2090009	-800.473754	-800.4374178	-401.090026	-438.185576	-686.2551441	-686.2561066	-402.298175	-327.04297	
Total	-1006.49901	-1006.49171	-1025.819426	-1025.80392	-1065.16606	-955.7379139	-1011.45333	-713.5266143	-1314.613307	-1314.576309	-915.229608	-952.323191	-1200.392489	-1200.393693	-916.436333	-841.18073	Total
IE	4.459639735	-0.70890438	6.261536402	0.77071156	-14.9136857	-0.555502785	-15.4746378	-112.7851909	-1.223860016	-0.499424143	-1.02661997	-0.07069397	0.472684081	-0.13599539	-0.38370198	-0.2687109	-136.081956

Table S3. Counterpoise corrected M062X/6-311+g* energies for Dopamine with each amino acid in the active site of each enzyme studied, as well as the calculated interaction energy. In each table, the first row is the BSSE-corrected energy for Dopamine, the second row is the BSSE-corrected energy for the amino acid residue, the third row is the energy for the complex of Dopamine and amino acid residue, and the fourth row is the difference between the complex and the 2 components, converted into kcal/mol. At the end of each set of rows is the total interaction energy.

MAOB	Cysteine	Glutamine	Isoleucine	Isoleucine 2	Isoleucine 3	Leucine	Leucine 2	Phenylalanine	Phenylalanine 2	Phenylalanine 3	Tyrosine	Tyrosine 2	Tyrosine 3	Tyrosine 4	Proline	Proline 2	Tryptophan	FAD		
Ligand	-516.937003	-516.936968	-516.936666	-516.936309	-516.936309	-516.9371585	-516.936426	-516.9365817	-516.9363347	-516.9363126	-516.937985	-516.936679	-516.9377874	-516.9366037	-516.936309	-516.93631	-516.9363131	-516.937667		
AA	-721.865186	-531.682319	-441.5674421	-366.344927	-441.595876	-441.5542908	-441.591962	-554.698041	-554.689438	-554.6698499	-629.911623	-629.912368	-629.9161975	-629.9156128	-401.062273	-401.06697	-686.2510702	-3351.31346		
Total	-1238.81325	-1048.62433	-958.5109602	-883.283147	-958.53192	-958.523843	-958.528085	-1071.639615	-1071.63073	-1071.605637	-1146.87306	-1146.85163	-1146.860175	-1146.854238	-917.999591	-918.00316	-1203.188046	-3868.32735		Total
IE	-6.93998336	-3.16632885	-4.299707644	-1.19933192	0.16583696	-20.327357	0.19052067	-3.070245224	-3.110887131	0.329718692	-14.7137988	-1.68278595	-3.8841514	-1.331346746	-0.63259546	0.07414715	-0.41613669	-47.8295616		-111.8439945

ALDH	Alanine	Cysteine	Glycine	Glycine 2	Glycine 3	Histidine	Isoleucine	Phenylalanine	Serine	Theronine	Tryptophan	Tyrosine	Valine	Valine 2		
Ligand	-516.932146	-516.932606	-516.932795	-516.933614	-516.932143	-516.9327858	-516.933016	-516.9325965	-516.9321438	-516.9321406	-516.932235	-516.934397	-516.932181	-516.9323931		
AA	-323.679696	-721.863978	-284.3836382	-284.378856	-284.378487	-548.6685715	-441.592472	-554.6919807	-398.857581	-438.2123552	-686.241545	-629.914394	-402.2930814	-402.266552		
Total	-840.610652	-1238.8053	-801.3233135	-801.329109	-801.31038	-1065.614696	-958.52794	-1071.638453	-915.7872225	-955.1457281	-1203.17434	-1146.86789	-919.2248521	-919.199868		Total
IE	0.746798428	-5.4714028	-4.317395882	-10.5039198	0.15746221	-7.742401719	-1.53879135	-8.707294212	1.570232082	-0.77331196	-0.35159734	-11.9871998	0.257444558	-0.57914546		-49.240523

PheOH	Arginine	Glutamic acid	Glutamic acid 2	Glycine	Histidine	Phenylalanine	Proline	Proline 2	Serine	Serine 2	Threonine	Tryptophan	Tyrosine	Tyrosine 2		
Ligand	-516.92907	-516.929449	-516.9293455	-516.929209	-516.930049	-516.929651	-516.929503	-516.9294567	-516.9290873	-516.9291198	-516.929556	-516.930095	-516.9300614	-516.9293473		
AA	-606.826858	-550.985473	-550.9717196	-284.38512	-548.680829	-554.6992079	-400.993586	-401.0519458	-398.8631027	-398.9004501	-438.157831	-686.25118	-629.9133156	-629.8802968		
Total	-1123.68061	-1067.97309	-1067.978092	-801.324929	-1065.61334	-1071.632712	-917.922014	-917.9900954	-915.8115534	-915.8351463	-955.128636	-1203.1875	-1146.858998	-1146.822885		Total
IE	47.2600867	-36.5023782	-48.3350293	-6.65182849	-1.54783376	-2.417831754	0.67470825	-5.455513627	-12.1507482	-3.495224523	-25.8837491	-3.90513406	-9.802383003	-8.308963104		-116.52582

DDC	Histidine	Histidine 2	Isoleucine	Lysine	Phenylalanine	Phenylalanine 2	Phenylalanine	PLP	Proline	Threonine	Tryptophan	Tyrosine		
Ligand	-516.909418	-516.909956	-516.8966533	-516.909776	-516.910639	-516.9108836	-516.909413	-516.9110985	-516.9096727	-516.9095186	-516.910276	-516.91051		
AA	-548.638836	-548.68086	-441.5951621	-497.27796	-554.696143	-554.6679698	-554.683234	-1157.050997	-400.9552758	-438.1980163	-686.251264	-629.889841		
Total	-1065.55453	-1065.62951	-958.4939889	-1014.12185	-1071.61837	-1071.587553	-1071.59202	-1674.28359	-917.8681957	-955.1073798	-1203.16242	-1146.81417		Total
IE	-3.93835755	-24.2783771	-1.363840442	41.34429611	-7.27025228	-5.459153182	0.39432509	-201.741023	-2.037654496	0.097359354	-0.55284214	-8.67012933		-213.4756489

Sult1a3	Alanine	Aspartic acid	Glutamic acid	Histidine	Histidine 2	Lysine	Phenylalanine	Phenylalanine 2	Phenylalanine 3	Proline		
Ligand	-516.899949	-516.900438	-516.9003756	-516.900898	-516.900207	-516.9017938	-516.900653	-516.9012512	-516.9003807	-516.8999021		
AA	-323.664708	-511.714275	-550.9937416	-548.672374	-549.064431	-497.2946941	-554.696437	-554.6974734	-554.6975171	-401.0842831		
Total	-840.565391	-1028.8139	-1068.035196	-1065.6016	-1065.89114	-1014.164068	-1071.61387	-1071.60521	-1071.599653	-917.9839418		Total
IE	-0.46050224	-124.99207	-88.52802495	-17.7761826	46.123116	20.34397533	-10.5268578	-4.069357692	-1.101580377	0.152742715		-180.834741

TyOH	Arginine	Glutamic acid	Glutamic acid 2	Glutamic acid 3	Glycine	Glycine 2	Histidine	Histidine 2	Iron	Leucine	Leucine 2	Leucine 3	Phenylalanine	Pheylalanine 2	Proline	Serine	Serine 2	Serine 3	Threonine	Tryptophan	Tyrosine	Tyrosine 2	Valine		
Ligand	-516.917691	-516.918131	-516.9190399	-516.91771	-516.917695	-516.9180701	-516.918296	-516.9187393	-516.9198647	-516.917693	-516.918278	-516.918058	-516.9188807	-516.9176978	-516.918161	-516.91769	-516.9176918	-516.917692	-516.9179266	-516.9178197	-516.9188281	-516.9177211	-516.9182552		
AA	-606.817056	-550.968525	-551.5568492	-550.966728	-284.382566	-284.3452813	-548.65	-548.6846245	-1262.554201	-441.5980536	-441.540837	-441.56558	-554.6609908	-554.6990756	-401.049496	-398.85686	-398.8946874	-398.879895	-438.1998496	-686.2479273	-629.8789492	-629.9138357	-402.2931774		
Total	-1123.69094	-1067.94345	-1068.493097	-1067.93442	-801.300436	-801.280661	-1065.57026	-1065.598484	-1779.533872	-958.5146475	-958.47221	-958.483548	-1071.58225	-1071.61674	-917.969479	-915.77545	-915.8115036	-915.802081	-955.1154362	-1203.166042	-1146.813375	-1146.829569	-919.2234691		Total
IE	27.4898433	-35.6381081	-10.79810441	-31.3622044	-0.10979534	-10.86193844	-1.23079776	3.06190123	-37.52874342	0.689705104	-8.21719109	0.056130725	-1.492520051	0.021111302	-1.14311837	-0.5653622	0.549454221	-2.81999444	1.465869095	-0.185337441	-9.788017428	1.247274278	-7.552964131		-124.71291

Tyronaise	Alanine	Asparagine	Copper	Glycine	Histidine	Histidine 2	Histidine 3	Histidine 4	Histidine 5	Histidine 6	Methionine	Phenylalanine	Phenylalanine 2	Valine		
Ligand	-516.92131	-516.921973	-516.9232795	-516.921237	-516.922464	-516.9216349	-516.921569	-516.9226209	-516.9215204	-516.9225078	-516.921255	-516.921342	-516.9212516	-516.9216772		
AA	-323.68899	-492.37373	-1639.357175	-284.351821	-548.641144	-548.6290629	-548.682092	-548.6568849	-548.6873658	-548.6892706	-800.446801	-554.697362	-554.6934032	-402.2325065		
Total	-840.609312	-1009.34049	-2156.473094	-801.279309	-1065.5704	-1065.549257	-1065.60323	-1065.580573	-1065.609074	-1065.615122	-1317.3733	-1071.6247	-1071.615091	-919.1531982		Total
IE	0.619878984	-28.1022687	-114.6079397	-3.92198583	-4.26007979	0.904277585	0.26748408	-0.669748419	-0.117838754	-2.098278189	-3.28809079	-3.76423057	-0.273449187	0.61842379		-158.69385

COMT	Asparagine	Asparagine 2	Aspartic acid	Aspartic acid 2	Glutamic acid	Leucine	Lysine	Magnesium	Methionine	Methionine 2	Proline	Threonine	Tryptophan	Tryptophan 2	Valine	Valine 2		
Ligand	-516.655676	-516.654531	-516.6551255	-516.654727	-516.654708	-516.6546771	-516.657617	-516.6564971	-516.6547474	-516.6555177	-516.655168	-516.655114	-516.6548242	-516.6549766	-516.654562	-516.65452		
AA	-492.361605	-492.36007	-511.6903501	-511.665286	-551.00176	-441.5954976	-497.284542	-199.2090549	-800.470246	-800.4374972	-401.085416	-308.825149	-686.2503375	-686.2525223	-402.293945	-327.04238		
Total	-1009.01919	-1009.01427	-1028.413804	-1028.37274	-1067.72992	-958.2509526	-1013.91324	-715.7381287	-1317.126164	-1317.101068	-917.741772	-825.481174	-1202.908076	-1202.932448	-918.949426	-843.69644		Total
IE	-1.19576265	0.205526304	-42.87649422	-33.0863006	-46.0916822	-0.488208039	18.147672	79.95928233	-0.734545678	-5.05341558	-0.74490774	-0.57154005	-1.828639867	-15.65600623	-0.57666554	0.91345679		-49.678231

Table S4. Counterpoise corrected M062X/6-311+g* energies for L-DOPA with each amino acid in the active site of each enzyme studied, as well as the calculated interaction energy. In each table, the first row is the BSSE-corrected energy for L-DOPA, the second row is the BSSE-corrected energy for the amino acid residue, the third row is the energy for the complex of L-DOPA and amino acid residue, and the fourth row is the difference between the complex and the 2 components, converted into kcal/mol. At the end of each set of rows is the total interaction energy

Sult1a3-LD	Alanine	Aspartic acid	Glutamic acid	Histidine	Histidine 2	Lysine	Phenylalanine	Phenylalanine 2	Phenylalanine 3	Proline		
Ligand	-705.070473	-705.071132	-705.0710719	-705.070665	-705.071396	-705.0722082	-705.072052	-705.0713487	-705.0709894	-705.0704663		
AA	-323.690404	-511.71583	-550.994845	-549.040231	-548.672925	-497.295253	-554.697188	-554.6957764	-554.6977241	-401.0843605		
Total	-1028.76127	-1216.85155	-1256.106967	-1254.10351	-1253.75969	-1202.422001	-1259.77747	-1259.77951	-1259.769422	-1106.155024		Total
IE	-0.24539826	-40.5311066	-25.75956618	4.633920459	-9.64183658	-34.22450555	-5.1654072	-7.77134434	-0.444461841	-0.12370032		-119.273406

ALDH-LD	Alanine	Cysteine	Glycine	Glycine 2	Glycine 3	Histidine	Isoleucine	Phenylalanine	Serine	Theronine	Tryptophan	Tyrosine	Valine	Valine 2		
Ligand	-705.101575	-705.10264	-705.1027977	-705.10211	-705.101579	-705.1020406	-705.102857	-705.1022979	-705.1015734	-705.1015761	-705.101776	-705.103809	-705.1018667	-705.1015287		
AA	-323.667606	-721.864049	-284.3788799	-284.383578	-284.378491	-548.6874346	-441.591994	-554.6915172	-398.8577324	-438.2123483	-686.241614	-629.91448	-402.2855731	-402.2931097		
Total	-1028.76899	-1426.98441	-989.4959749	-989.490072	-989.479923	-1253.799655	-1146.70766	-1259.804484	-1103.968869	-1143.314013	-1391.34336	-1335.02777	-1107.388574	-1107.394587		
IE	0.11805399	-11.1197909	-8.971678397	-2.75075378	0.09225017	-6.387859712	-8.0376487	-6.694734448	0.27429507	-0.055564711	0.019851263	-5.94771388	-0.711474664	0.094902654		-50.077866

COMT-LD	Asparagine	Asparagine 2	Aspartic acid	Aspartic acid 2	Glutamic acid	Leucine	Lysine	Magnesium	Methionine	Methionine 2	Proline	Tryptophan	Tryptophan 2	Valine	Valine 2		
Ligand	-704.522149	-704.52274	-704.522337	-704.522546	-704.52233	-704.5223751	-704.523954	-704.5230186	-704.5230728	-704.5224906	-704.522584	-704.522568	-704.5227084	-704.5221414	-704.52224		
AA	-492.351419	-492.35072	-511.6927239	-511.690342	-551.001776	-441.5953082	-497.285224	-199.2090358	-800.4546315	-800.470411	-401.088289	-686.250449	-686.2526211	-402.2793127	-402.293946		
Total	-1196.87452	-1196.91405	-1216.103219	-1216.09397	-1255.42785	-1146.119749	-1202.01956	-904.3060974	-1504.979895	-1504.999283	-1105.61559	-1390.77622	-1390.780304	-1106.801782	-1106.81689		Total
IE	-0.60033395	-25.4579631	70.181792	74.61912879	60.4014321	-1.296099537	-132.016935	-360.2174485	-1.374594073	-4.004298134	-2.96166788	-2.0106308	-3.121270531	-0.2059254	-0.44329028		-328.5181045

MAOB-LD	Cysteine	Glutamine	Isoleucine	Isoleucine 2	leucine	Leucine 2	(iso)Leucine 3	Phenylalanine	Phenylalanine 2	Phenylalanine 3	Tyrosine	Tyrosine 2	Tyrosine 3	Tyrosine 4	Proline	Proline 2	Tryptophan	FAD		
Ligand	-705.102631	-705.103039	-705.1019795	-705.10213	-705.102901	-705.1019718	-705.10228	-705.1019873	-705.1019672	-705.1027588	-705.103731	-705.102363	-705.1033297	-705.1025862	-705.101974	-705.10197	-705.1019689	-705.103187		
AA	-721.849198	-531.68249	-441.5959021	-441.576552	-441.588006	-441.5868441	-441.58364	-554.6894555	-554.6758477	-554.6980969	-629.911825	-629.912656	-629.9160684	-629.9158172	-401.068242	-401.08282	-686.2510951	-3351.31369		
Total	-1426.96272	-1236.79279	-1146.697811	-1146.67768	-1146.7089	-1146.689091	-1146.68727	-1259.793704	-1259.777784	-1259.807073	-1335.0283	-1335.01575	-1335.027312	-1335.029076	-1106.17026	-1106.185	-1391.353187	-4056.41537		Total
IE	-6.83393865	-4.55565434	0.044465323	0.631661103	-11.2908638	-0.172336071	-0.8491559	-1.419006058	0.019567001	-3.901498901	-7.99868225	-0.45760252	-4.966252	-6.697253271	-0.0244986	-0.1009311	-0.077071972	0.945306666		-47.70374533

Tyrosinase-LD	Alanine	Asparagine	Copper	Glycine	Histidine	Histidine 2	Histidine 3	Histidine 4	Histidine 5	Histidine 6	Methionine	Phenylalanine	Phenylalanine 2	Valine	Valine2	
Ligand	-705.089906	-705.090896	-705.0917707	-705.089828	-705.091639	-705.0901583	-705.090174	-705.0908601	-705.0901084	-705.0908004	-705.089808	-705.090451	-705.0898571	-705.0898914	-705.090612	
AA	-323.688976	-492.359272	-1639.367167	-284.353393	-548.65663	-548.6820481	-548.628631	-548.6786199	-548.687382	-548.6891241	-800.460537	-554.697052	-554.6934658	-402.2696079	-402.222621	
Total	-1028.77912	-1197.46974	-2344.88424	-989.445018	-1253.75146	-1253.767334	-1253.71841	-1253.772105	-1253.772685	-1253.78072	-1505.55051	-1259.79431	-1259.783226	-1107.360712	-1107.31914	Total
IE	-0.14870846	-12.2829212	-266.8812876	-1.12779208	-2.00356002	3.05761534	0.24516169	-1.647335429	3.015592911	-0.499311192	-0.10278104	-4.26897348	0.060609888	-0.761006871	-3.70879889	-287.0535

PheOH-LD	Arginine	Glutamic acid	Glutamic acid 2	Glycine	Histidine	Phenylalanine	Proline	Proline 2	Serine	Serine 2	Threonine	Tryptophan	Tyrosine	Tyrosine 2		
Ligand	-705.077374	-705.077967	-705.0774335	-705.077608	-705.078586	-705.0776151	-705.077478	-705.0778114	-705.0775107	-705.0777912	-705.077693	-705.078324	-705.0780181	-705.0775961		
AA	-606.825043	-551.008419	-550.9850699	-284.389013	-548.687639	-554.6751797	-401.070699	-401.061855	-398.8806273	-398.8717417	-438.167411	-686.251233	-629.9135581	-629.8956661		
Total	-1311.92364	-1256.13005	-1256.080838	-989.474569	-1253.78663	-1259.753966	-1106.14993	-1106.142743	-1103.967581	-1103.964885	-1143.25907	-1391.33147	-1335.001875	-1334.982285		Total
IE	-13.3160359	-27.4014492	-11.50520408	-4.98736832	-12.8070303	-0.734821782	-1.10015719	-1.930502856	-5.925747911	-9.633508274	-8.76180973	-1.1989686	-6.462870943	-5.661865106		-111.42734

DDC-LD	Histidine	Histidine 2	Isoleucine	Lysine	Phenylalanine	Phenylalanine 2	Phenylalanine	PLP	Proline	Threonine	Threonine 2	Tryptophan	Tyrosine		
Ligand	-704.571257	-704.571279	-704.5717735	-704.572037	-704.572973	-704.5713007	-704.572442	-704.5739726	-704.5714975	-704.57112	-704.571311	-704.571674	-704.5723732		
AA	-548.682302	-548.65715	-441.5974525	-497.259337	-554.67409	-554.6850341	-554.695945	-1157.718312	-401.0592461	-438.2036758	-438.189353	-686.255159	-629.912959		
Total	-1253.2529	-1253.2104	-1146.180428	-1202.02732	-1259.2535	-1259.264077	-1259.27997	-1862.216798	-1105.631848	-1142.772389	-1142.76314	-1390.82979	-1334.491399		Total
IE	0.41581666	11.31210058	-7.029191991	-122.959413	-4.04078279	-4.858209297	-7.27139309	47.36874374	-0.692946818	1.51000623	-1.55497544	-1.8554841	-3.806935729		-93.4626649

TyOH-LD	Arginine	Glutamic acid	Glutamic acid 2	Glutamic acid 3	Glycine	Glycine 2	Histidine	Histidine 2	Iron	Leucine	Leucine 2	Leucine 3	Phenylalanine	Pheylalanine 2	Proline	Serine	Serine 2	Serine 3	Threonine	Tryptophan	Tyrosine	Tyrosine 2	Valine		
Ligand	-705.085262	-705.087257	-705.0857549	-705.085292	-705.085281	-705.0855623	-705.086376	-705.0857575	-705.0870875	-705.0852723	-705.085792	-705.085856	-705.0852616	-705.086283	-705.085988	-705.08527	-705.0852657	-705.085278	-705.0857387	-705.0853645	-705.0862799	-705.0852899	-705.0860165		
AA	-606.817105	-551.518763	-550.9785626	-550.976578	-284.382559	-284.3652307	-548.684011	-548.6621704	-1262.516703	-441.5774386	-441.571905	-441.55994	-554.6825606	-554.6693629	-401.056577	-398.87625	-398.8871487	-398.87158	-438.2055777	-686.2362814	-629.8930902	-629.9137037	-402.2933395		
Total	-1311.91246	-1256.65801	-1256.05596	-1256.05576	-989.467386	-989.4600968	-1253.76239	-1253.741706	-1967.903681	-1146.662414	-1146.66075	-1146.65537	-1259.767826	-1259.758657	-1106.14562	-1103.9608	-1103.972673	-1103.95819	-1143.299432	-1391.321601	-1334.98837	-1334.996944	-1107.379621		Total
IE	-6.33384934	-32.622999	5.244398096	3.83723377	0.28467345	-5.83827999	5.01960035	3.90410683	-188.1842682	0.186177049	-1.91678801	-6.00752118	-0.002462347	-1.889557861	-1.91733331	0.46053926	-0.162228149	-0.83571527	-5.092801214	0.028318876	-5.647682136	1.285931373	-0.166561102		-236.20051

Table S5. Counterpoise corrected M062X/6-311+g* energies for the natural substrate of each enzyme in the active site of each enzyme studied, as well as the calculated interaction energy. In each table, the first row is the BSSE-corrected energy for the natural substrates, the second row is the BSSE-corrected energy for the amino acid residue, the third row is the energy for the complex of the natural substrate and amino acid residue, and the fourth row is the difference between the complex and the 2 components, converted into kcal/mol. At the end of each set of rows is the total interaction energy.

ALDH-HV	Alanine	Cysteine	Glycine	Glycine 2	Glycine 3	Histidine	Isoleucine	Phenylalanine	Serine	Theronine	Tryptophan	Tyrosine	Valine	Valine 2		
Ligand	-574.52447	-574.52491	-574.52523	-574.52492	-574.52447	-574.52506	-574.52509	-574.5248075	-574.5244787	-574.5244782	-574.52457	-574.5266702	-574.5244825	-574.52468		
AA	-323.66762	-721.86465	-284.37914	-284.37162	-284.3785	-548.68749	-441.59275	-554.6929153	-398.8652482	-438.2123006	-686.24161	-629.9165459	-402.2931181	-402.28525		
Total	-898.19201	-1296.395	-858.91076	-858.89806	-858.90296	-1123.2222	-1016.1221	-1129.221343	-973.3895347	-1012.736798	-1260.7661	-1204.45165	-976.8175996	-976.81053		
IE	0.0473958	-3.3911511	-4.0076848	-0.9560371	0.0107348	-6.0349233	-2.6522254	-2.271462026	0.120603561	-0.012394568	0.0482505	-5.292469089	0.000635667	-0.3740465		-24.764773

ALDH-DOPAL	Alanine	Cysteine	Glycine	Glycine 2	Glycine 3	Histidine	Isoleucine	Phenylalanine	Serine	Theronine	Tryptophan	Tyrosine	Valine	Valine 2		
Ligand	-535.23118	-535.23171	-535.23119	-535.23248	-535.23181	-535.23177	-535.23198	-535.2315455	-535.2311907	-535.2311848	-535.23127	-535.2331319	-535.2312071	-535.23142		
AA	-323.6676	-721.86454	-284.37849	-284.37884	-284.37163	-548.68747	-441.59266	-554.6929356	-398.8652767	-438.212362	-686.2416	-629.915367	-402.293109	-402.28536		
Total	-858.89871	-1257.1004	-819.60966	-819.62459	-819.60496	-1083.9295	-976.82915	-1089.928073	-934.0962324	-973.4436249	-1221.4728	-1165.153888	-937.5243221	-937.51751		
IE	0.0454505	-2.575673	0.006747	-8.3266689	-0.9576623	-6.4599361	-2.8290732	-2.253800771	0.147437122	-0.049019787	0.0555365	-3.38158971	-0.003770705	-0.4568764		-27.0409

PheOH-Phe	Arginine	Glutamic acid	Glutamic acid	Glycine	Histidine	Phenylalanine	Proline	Proline 2	Serine	Serine 2	Threonine	Tryptophan	Tyrosine	Tyrosine 2		
Ligand	-554.65316	-554.65306	-554.65324	-554.6534	-554.65423	-554.6533	-554.65326	-554.6535424	-554.6533864	-554.653612	-554.65352	-554.6532429	-554.6531549	-554.65348		
AA	-606.82504	-551.01198	-550.9863	-284.38915	-548.68782	-554.68228	-401.06848	-401.0052648	-398.8890869	-398.8720764	-438.17155	-686.2513962	-629.9141045	-629.89562		
Total	-1161.4989	-1105.6714	-1105.6559	-839.04967	-1103.3632	-1109.3357	-955.7234	-955.6620531	-953.5518031	-953.5414202	-992.83937	-1240.905216	-1184.569085	-1184.5586		Total
IE	-12.973744	-3.9611361	-10.264286	-4.4647634	-13.243584	-0.042684	-1.0460182	-2.03676406	-5.867115306	-9.871853325	-8.9760797	-0.362061686	-1.145923963	-5.9427497		-80.218963

MAOB-3MT	Cysteine	Glutamine	Isoleucine	Isoleucine 2	leucine	Leucine 2	(iso)Leucine	Phenylalanine	Phenylalanine 2	Phenylalanine 3	Tyrosine	Tyrosine 2	Tyrosine 3	Tyrosine 4	Proline	Proline 2	Tryptophan	FAD		
Ligand	-556.23417	-556.23445	-556.23406	-556.23388	-556.23376	-556.23377	-556.23455	-556.2340236	-556.2337846	-556.2337652	-556.23396	-556.2341135	-556.2357016	-556.23491	-556.23377	-556.23377	-556.23377	-556.23566		
AA	-721.84922	-531.68236	-441.58364	-441.57658	-441.5959	-441.58684	-441.58772	-554.6980705	-554.6894861	-554.6786364	-629.91559	-629.9125838	-629.91674	-629.91165	-401.06824	-401.08281	-686.25109	-3351.3141		
Total	-1276.0861	-1087.9191	-997.82458	-997.81139	-997.8294	-997.82192	-997.85367	-1110.937311	-1110.927895	-1110.911793	-1186.1516	-1186.148746	-1186.16333	-1186.1674	-957.30169	-957.31791	-1242.4855	-3907.6289		Total
IE	-1.6791477	-1.4701406	-4.3158616	-0.5852436	0.164742	-0.8179241	-19.703244	-3.273648663	-2.914263305	0.382014606	-1.2640319	-1.285536042	-6.832601423	-13.069996	0.2037385	-0.8347263	-0.4265395	-49.676742		-107.39915

COMT-DOPAC	Asparagine	Asparagine 2	Aspartic acid	spartic acid	Glutamic acid	Leucine	Lysine	Magnesium	Methionine	Methionine 2	Proline	Tryptophan	Tryptophan 2	Valine	Valine 2		
Ligand	-609.26806	-609.26731	-609.26756	-609.26775	-609.26753	-609.26738	-609.26915	-609.2682055	-609.2675185	-609.2682132	-609.26766	-609.2676901	-609.2676506	-609.26732	-609.26732		
AA	-492.35068	-492.35141	-511.69288	-511.69035	-551.00179	-441.59518	-497.28485	-199.2090352	-800.4701883	-800.4547723	-401.08824	-686.2504013	-686.2520173	-402.29394	-402.27933		
Total	-1101.6697	-1101.6198	-1120.7943	-1120.7804	-1160.1129	-1050.8675	-1106.8528	-809.2427308	-1409.752306	-1409.724097	-1010.3668	-1295.524922	-1295.5162	-1011.5628	-1011.5476		Total
IE	-31.961883	-0.684621	104.22433	111.52745	98.182404	-3.1322645	-187.52958	-480.3522911	-9.161420954	-0.697294831	-6.8403474	-4.285989659	2.176412534	-0.991411	-0.591146		-410.11765

COMT-3HP	Asparagine	Asparagine 2	Aspartic acid	spartic acid	Glutamic acid	Leucine	Lysine	Magnesium	Methionine	Methionine 2	Proline	Tryptophan	Tryptophan 2	Valine	Valine 2		
Ligand	-589.31869	-589.3186	-589.31914	-589.31869	-589.31949	-589.32144	-589.31984	-589.3200908	-589.3186902	-589.3198641	-589.32055	-589.3197392	-589.3186902	-589.31961	-589.32078		
AA	-492.35153	-492.35078	-511.69112	-511.69154	-551.52347	-441.59575	-497.27433	-199.209084	-800.4700163	-800.4559224	-401.08523	-686.2505997	-686.2522268	-402.29401	-402.26957		
Total	-1081.7265	-1081.6762	-1100.8004	-1100.8011	-1140.9455	-1030.9233	-1086.9256	-789.4900998	-1389.796515	-1389.779811	-990.41727	-1275.586415	-1275.577118	-991.6201	-991.59314		Total
IE	-35.303073	-4.3070941	131.71085	131.21907	-64.35394	-3.8275061	-207.99822	-602.9895537	-4.900118146	-2.52573515	-7.2101212	-10.0881935	-3.891303126	-4.0694744	-1.7517348		-690.28614

TyOH-Tyr	Arginine	Glutamic acid	Glutamic acid	Glutamic acid	Glycine	Glycine 2	Histidine	Histidine 2	Iron	Leucine	Leucine 2	Leucine 3	Phenylalanine	Pheylalanine	Proline	Serine	Serine 2	Serine 3	Threonine	Tryptophan	Tyrosine	Tyrosine 2	Valine		
Ligand	-629.87127	-629.87269	-629.87129	-629.87168	-629.87155	-629.87128	-629.87163	-629.8720719	-629.8730322	-629.8712687	-629.87179	-629.8718473	-629.8722189	-629.87127	-629.87188	-629.87129	-629.87129	-629.87127	-629.87174	-629.87136	-629.87129	-629.87231	-629.87201		
AA	-606.81704	-551.52764	-550.97414	-550.97842	-284.36525	-284.38255	-548.66212	-548.6843126	-1262.55424	-441.5774406	-441.57202	-441.5582681	-554.6691328	-554.68255	-401.06795	-398.87157	-398.87626	-398.88715	-438.20529	-686.23632	-629.91397	-629.893	-402.29323		
Total	-1236.6984	-1181.433	-1180.8381	-1180.8451	-914.24609	-914.25346	-1178.5292	-1178.548557	-1892.676825	-1071.448447	-1071.4469	-1071.441827	-1184.544348	-1184.5538	-1030.9435	-1028.7443	-1028.7468	-1028.7587	-1068.0848	-1316.1077	-1259.7833	-1259.7746	-1032.1651		Total
IE	-6.3463958	-20.518166	4.6077357	3.1068642	-5.8271524	0.2371384	2.8444679	4.911681264	-156.5963843	0.176963954	-1.9570678	-7.349267645	-1.880123256	0.0023971	-2.3156676	-0.8827559	0.4665966	-0.148694	-4.8768137	-0.0319396	1.2116361	-5.846255	0.0801361		-197.01118

Table S6. Counterpoise corrected MP2 and M062X/6-311+g* energies for Dopamine with each amino acid in the active site of the Sult1a3 enzyme, as well as the calculated interaction energy. In each table, the first row is the BSSE-corrected energy for Dopamine, the second row is the BSSE-corrected energy for the amino acid residue, the third row is the energy for the complex of Dopamine and amino acid residue, and the fourth row is the difference between the complex and the 2 components, converted into kcal/mol. At the end of each set of rows is the total interaction energy.

Sult1a3-DA	Alanine	Aspartic acid	Glutamic acid	Histidine	Histidine 2	Lysine	Phenylalanine	Phenylalanine 2	Phenylalanine 3	Proline			
Ligand	-5.16E+02	-5.16E+02	-5.16E+02	-5.16E+02	-5.16E+02	-5.16E+02	-5.16E+02	-5.16E+02	-5.16E+02	-5.16E+02			
AA	-3.23E+02	-5.11E+02	-5.50E+02	-5.47E+02	-5.48E+02	-4.96E+02	-5.53E+02	-5.53E+02	-5.53E+02	-4.00E+02	MP2		
Total	-8.38E+02	-1.03E+03	-1.07E+03	-1.06E+03	-1.06E+03	-1.01E+03	-1.07E+03	-1.07E+03	-1.07E+03	-9.16E+02	Total		
IE	-0.66	-119.99	-85.27	-15.47	45.33	23.41	-8.96	-3.75	-1.82	-0.16	-167.34		
Sult1a3-DA	Alanine	Aspartic acid	Glutamic acid	Histidine	Histidine 2	Lysine	Phenylalanine	Phenylalanine 2	Phenylalanine 3	Proline			
Ligand	-516.899949	-516.9004383	-516.9003756	-516.9008979	-516.900207	-516.9017938	-516.9006528	-516.9012512	-516.9003807	-516.8999021			
AA	-323.6647081	-511.7142749	-550.9937416	-548.6723738	-549.0644311	-497.2946941	-554.6964375	-554.6974734	-554.6975171	-401.0842831	M062X		
Total	-840.565391	-1028.813901	-1068.035196	-1065.6016	-1065.891136	-1014.164068	-1071.613866	-1071.60521	-1071.599653	-917.9839418	Total		
IE	-0.460502239	-124.9920699	-88.52802495	-17.77618261	46.12311605	20.34397533	-10.52685778	-4.069357692	-1.101580377	0.152742715	-180.8347414	-13.50	Diff from MP2

Table S7. Counterpoise corrected MP2 and M062X/6-311+g* energies for PCM with each amino acid in the active site of the Sult1a3 enzyme, as well as the calculated interaction energy. In each table, the first row is the BSSE-corrected energy for PCM, the second row is the BSSE-corrected energy for the amino acid residue, the third row is the energy for the complex of PCM and amino acid residue, and the fourth row is the difference between the complex and the 2 components, converted into kcal/mol. At the end of each set of rows is the total interaction energy.

Sult1a3-PCM	Alanine	Aspartic acid	Glutamic acid	Histidine	Histidine 2	Lysine	Phenylalanine	Phenylalanine 2	Phenylalanine 3	Proline				
Ligand	-5.14E+02	-5.14E+02	-5.14E+02	-5.14E+02	-5.14E+02	-5.14E+02	-5.53E+02	-5.14E+02	-5.14E+02	-5.14E+02				
AA	-3.23E+02	-5.11E+02	-5.50E+02	-5.48E+02	-5.47E+02	-4.96E+02	-5.14E+02	-5.53E+02	-5.53E+02	-4.00E+02		MP2		
Total	-8.36E+02	-1.02E+03	-1.06E+03	-1.06E+03	-1.06E+03	-1.01E+03	-1.07E+03	-1.07E+03	-1.07E+03	-9.14E+02		Total		
IE	-0.56	40.70	51.00	-110.12	-1.52	-125.97	-4.93	-4.96	-2.64	-1.28		-160.28		

Sult1a3-PCM	Alanine	Aspartic acid	Glutamic acid	Histidine	Histidine 2	Lysine	Phenylalanine	Phenylalanine 2	Phenylalanine 3	Proline				
Ligand	-514.8285107	-514.8286972	-514.8289917	-514.829066	-514.8285464	-514.8290116	-514.8292282	-514.829588	-514.8288885	-514.8285138				
AA	-323.6628728	-511.7179036	-550.9873594	-549.0360433	-548.6800546	-497.2843592	-554.6965116	-554.697447	-554.6972446	-401.0842667		M062X		
Total	-838.4923214	-1026.482886	-1065.738006	-1064.047414	-1063.51065	-1012.324592	-1069.536625	-1069.535721	-1069.529154	-915.9148359		Total		
IE	-0.59	39.98	49.16	-114.40	-1.29	-132.54	-6.83	-5.45	-1.90	-1.29		-175.14	-14.85	Diff from MP2
